# Remus: A Web Application for Prioritization of Regulatory Regions and Variants in Monogenic Diseases

**DOI:** 10.3389/fgene.2021.638960

**Published:** 2021-03-05

**Authors:** Paweł Sztromwasser, Damian Skrzypczak, Arkadiusz Michalak, Wojciech Fendler

**Affiliations:** ^1^Department of Biostatistics and Translational Medicine, Medical University of Lodz, Łódź, Poland; ^2^Biostatistics Group, Department of Genetics, Wrocław University of Environmental and Life Sciences, Wrocław, Poland; ^3^Department of Pediatrics, Diabetology, Endocrinology and Nephrology, Medical University of Lodz, Łódź, Poland; ^4^Department of Radiation Oncology, Dana-Farber Cancer Institute, Harvard Medical School, Boston, MA, United States

**Keywords:** regulatory variants, whole-genome sequencing, monogenic disorder, variant prioritization, web application, regulatory regions

## Abstract

**Background:**

Analysis of variants in distant regulatory elements could improve the current 25–50% yield of genetic testing for monogenic diseases. However, the vast size of the regulome, great number of variants, and the difficulty in predicting their phenotypic impact make searching for pathogenic variants in the regulatory genome challenging. New tools for the identification of regulatory variants based on their relevance to the phenotype are needed.

**Methods:**

We used tissue-specific regulatory *loci* mapped by ENCODE and FANTOM, together with miRNA–gene interactions from miRTarBase and miRWalk, to develop Remus, a web application for the identification of tissue-specific regulatory regions. Remus searches for regulatory features linked to the known disease-associated genes and filters them using activity status in the target tissues relevant for the studied disorder. For user convenience, Remus provides a web interface and facilitates in-browser filtering of variant files suitable for sensitive patient data.

**Results:**

To evaluate our approach, we used a set of 146 regulatory mutations reported causative for 68 distinct monogenic disorders and a manually curated a list of tissues affected by these disorders. In 89.7% of cases, Remus identified the regulator containing the pathogenic mutation. The tissue-specific search limited the number of considered variants by 82.5% as compared to a tissue-agnostic search.

**Conclusion:**

Remus facilitates the identification of regulatory regions potentially associated with a monogenic disease and can supplement classical analysis of coding variations with the aim of improving the diagnostic yield in whole-genome sequencing experiments.

## Introduction

Over the past decade, the generation and analysis of sequencing data in the clinical setting focused mainly on the protein-coding genes. Gene-targeted approaches are economically and computationally justified (Escaramís et al., 2015) and yield interpretable data. Coding variant analysis is supported by rich functional annotation, making it easier to shortlist relevant variants and link candidate hits to a biological process and, eventually, to the disease phenotype. However, the plunging costs of sequencing enable a shift from targeted assays to whole-genome sequencing (WGS), a technique that offers unparalleled diagnostic rates for monogenic disorders (Gilissen et al., 2014; Lionel et al., 2018). WGS produces data with the least technical bias (Meienberg et al., 2016), enables detection of structural variants (Escaramís et al., 2015), and provides an opportunity to interrogate non-coding regions of the genome (Smedley et al., 2016). The increase in conclusive diagnoses, as compared to the targeted approaches, stems most often from structural variants, deep intronic variants, and mutations previously missed (Lionel et al., 2018). Causative regulatory variants are rarely found, despite a number of already known pathogenic mutations identified in the regulome (Smedley et al., 2016).

The scarcity of pathogenic regulatory mutations stems partly from the difficulty in sifting through the variants in the regulatory genome. It remains challenging, due both to the sheer number of variants and the difficulty of interpretation (Gilissen et al., 2014). According to a conservative estimation, the human regulome comprises 8.5% of the genome, making it over four times larger than the protein-coding sequence (The Encode Project Consortium, 2012) typically interrogated for pathogenic variants. Population frequency databases such as gnomAD (Karczewski et al., 2019) and whole-genome pathogenicity scores [e.g., CADD (Kircher et al., 2014), DANN (Quang et al., 2015), and ReMM (Smedley et al., 2016)] help in excluding benign polymorphisms and prioritization of variants with a likely functional effect. Still, the regulatory regions, being under a weaker evolutionary constraint than the coding regions (The Encode Project Consortium, 2012), make shortlisting variants solely based on these factors insufficient.

Repositories of known regulatory elements [e.g., ORegAnno (Lesurf et al., 2016) and VISTA Enhancer Browser (Visel et al., 2007)] or enhancers implicated in pathogenic processes (DiseaseEnhancer database) (Zhang et al., 2018) can help in the identification of variants in known regulators. Another invaluable resource for regulatory variant analysis are repositories of regulatory regions, which have been comprehensively mapped by initiatives such as ENCODE (The Encode Project Consortium, 2012), FANTOM (Forrest et al., 2014), or BLUEPRINT (Adams et al., 2012). In these databases, regulatory regions have been linked to tissue-specific activity status, which could potentially help in the prioritization of individual regulatory elements based on the disease phenotype. Access to these regulatory datasets is provided through dedicated browsers, e.g., SlideBase (Ienasescu et al., 2016) for FANTOM enhancers and promoters, and the ENCODE portal (Davis et al., 2018). However, these tools are targeted mainly at researchers studying the regulome and are not easy to access or navigate for clinicians searching for regulatory variants in a disease.

Tissue-specific regulatory data, in the form of genome tracks, are also available in major genome browsers (Kent et al., 2002; Robinson et al., 2011; Zerbino et al., 2018), allowing visual inspection of the regulatory landscapes in the genomic *loci* of interest. Subsets of these tracks are provided in variant annotation software such as ENSEMBL VEP (McLaren et al., 2016) and ANNOVAR (Wang et al., 2010). Other resources integrate this genome-wide regulatory information in various ways: to support studying the role of enhancers in complex diseases (Human Enhancer Disease Database, HEDD) (Wang et al., 2018), to enrich GeneCards suite with enhancer information (GeneHancer) (Fishilevich et al., 2017), or to support variant annotation and prioritization (Genomiser) (Smedley et al., 2016).

Despite the long list of resources with a focus on regulatory elements, we found no software that would integrate the wide spectrum of the available regulatory data for easy querying and facilitate the interrogation of patient genomes in the search for candidate variants. In a scenario where a patient’s variants had been scrutinized for coding changes with no conclusive diagnosis, we envisioned a tool that would facilitate a straightforward identification of candidate variants in the regulatory regions relevant for the patient’s phenotype. The main requirements set forth included a wide choice of regulatory elements, ease of use for shortlisting candidate variants, and a possibility of analysis of sensitive data.

## Materials and Methods

Remus is a web server that integrates regulatory data collected from several sources. The application enables searching regulatory elements based on target genes and tissues, and the identified regulators can be used to filter genetic variants. The regulatory data aggregated by Remus are available for both the hg19 and hg38 genome builds and are described in detail in the next sections. Remus was developed in Python 3 using the Flask framework. Users interact with it *via* a web application interface implemented with Bootstrap^1^ or *via* an application programming interface (API). The variant filtering functionality was implemented in JavaScript.

### Datasets

Remus queries regulatory regions for a set of target genes. The list of genes and their transcript coordinates were downloaded from the UCSC Table Browser (Karolchik, 2004) (*ncbiRefSeq* table accessed December 2019) for both supported genome builds (hg19 and hg38) and stored in an SQLite database. The regulatory datasets aggregated in Remus can be divided into two categories: microRNA–gene interactions and tissue-specific regulatory regions. MicroRNA–transcript interactions were downloaded from miRTarBase (Chou et al., 2018) (accessed Oct. 2018) and miRWalk (Sticht et al., 2018) (accessed Oct. 2019) and stored in an SQLite database. In the case of miRWalk, only interactions with the 3′-UTR of the genes were used (Supplementary Table 1).

The tissue-specific regulatory regions are genome-wide tracks of regulatory elements active in single tissues or primary cells. These were downloaded from ENCODE and FANTOM repositories and processed as described in the next paragraphs. The regulatory elements were functionally categorized into promoters, DNA-binding sites (enhancers, repressors, and insulators), and accessible chromatin regions and stored in compressed and indexed BED files.

### Data Preparation

Coordinates of the ENCODE (The Encode Project Consortium, 2012) regulatory features were downloaded from the ENCODE portal (Davis et al., 2018) (accessed Sept. 2019) as compressed BED files (Supplementary Table 1). Downloaded files came from experiments with status “*released*,” only on human samples classified as *tissue* or *primary cell*. Experiments with error flags from internal ENCODE audits were excluded. For DNA-binding tracks, we used *narrowPeak* BED files with status *released*, containing *optimal idr thresholded peaks* or *pseudoreplicated idr thresholded peaks*, and originating from transcription factor (TF) chromatin immunoprecipitation (ChIP) sequencing experiments. Experiments for transcription-repressing TFs (CTCF) and transcription-promoting TFs were not separated, as variants in the binding sites of both types of TFs can have functional implications for the regulated genes. These tracks are also referred to as transcription factor binding site (TFBS) tracks. Accessible chromatin regions were extracted from results of sequencing assays based on formaldehyde-assisted isolation of regulatory elements (FAIRE-seq) and deoxyribonuclease footprinting (DNase-seq). We used *narrowPeak* BED files with status *released*, containing *peaks* and originating from DNase-seq and FAIRE-seq experiments. To provide equal tissue and cell type coverage for both genome builds, liftOver tool (Hinrichs, 2006) was used to convert BED files in the hg19 coordinate system to hg38, and *vice versa* (Supplementary Figure 1). Next, coordinates from biological replicates of the same tissue or cell type, including lifted-over data, were collapsed (merged) using BEDtools (Quinlan and Hall, 2010).

Coordinates of SCREEN *cis* candidate regulatory elements (ccREs) (ENCODE Project Consortium et al., 2020) were downloaded from the ENCODE portal (accessed September 2019) as compressed BED files (Supplementary Table 1). The ccREs were divided into promoters, enhancers, insulators, and accessible chromatin according to the annotations provided in the BED files. If multiple files for a tissue or cell type were present, collapsing was done as described above for the ENCODE data. As all SCREEN tracks were in the hg19 coordinate system, hg38 data were produced using the liftOver tool (Hinrichs, 2006).

Coordinates of FANTOM (Forrest et al., 2014) enhancers were downloaded from SlideBase (Ienasescu et al., 2016) (accessed October 2019) as tissue and organ facet-specific BED files. FANTOM promoters were generated from a normalized (relative log expression, RLE) cap analysis of gene expression (CAGE) peak expression table for human samples, downloaded from the FANTOM5 repository (accessed October 2019). The table was split into tissue and primary cell type tracks using the FANTOM ontology. CAGE peaks with normalized tags per million (TPM) expression values below 10 were filtered out. The peaks, indicating transcription start sites, were expanded 200 bp upstream to include the promoter region. As all FANTOM tracks were in the hg19 coordinate system, hg38 data were produced using the liftOver tool (Hinrichs, 2006).

Data download and processing was automated in a build script available in Remus source code repository^2^. This facilitates data provenance tracking and deployment of a private Remus instance on one’s infrastructure. Comparisons between processed datasets were made using BEDtools (Quinlan and Hall, 2010) and R packages. The R Markdown script documenting this analysis can be found in a dedicated repository^3^.

### Searching Regulatory Elements

**FIGURE 1:**
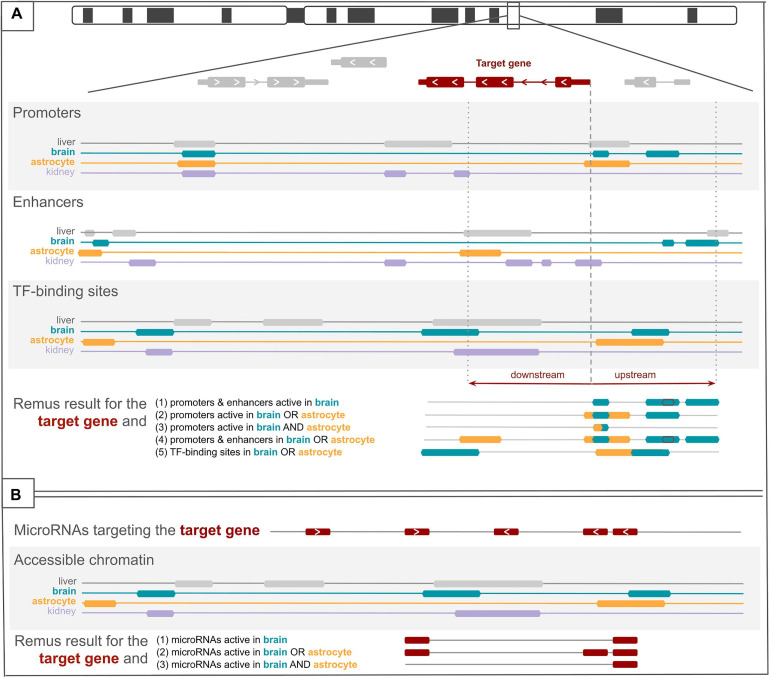
Visualization of the search protocol employed in Remus. In **(A)**, three datasets with *cis*-regulatory elements are depicted, each with four tissue-specific regulator tracks. For a selected target gene, regulatory features are identified based on the overlap with a window around a gene’s transcription start sites (the figure presents only one transcript). Upstream and downstream distances from the transcript start site can be set for each dataset individually. On the *bottom*, five sets of identified regulators are presented, depending on the example search criteria (1–5). In **(B)**, a track with microRNAs (miRNAs) targeting the target gene is shown, and four tracks of accessible chromatin regions. Remus includes in the result only those miRNAs that overlap an accessible chromatin region in the selected tissues (1–3).

For a given genome build (hg19 or hg38), a set of HGNC (HUGO Gene Nomenclature Committee) gene symbols (target genes), and one or more tissues or cell types (target tissues), regulatory regions are identified using the following protocol (Figure 1). A set of transcription start site (TSS) intervals overlapping the target genes’ transcription start sites is created using all transcripts of the target genes. The first base of a transcript is used to calculate a TSS interval, and the upstream and downstream spans can be tuned by the user, independently for each dataset. A regulatory region from a *cis*-regulatory dataset (e.g., SCREEN promoters and FANTOM enhancers) is included in the result if: (1) it overlaps the TSS interval and (2a) *active-in-any* mode is selected for the dataset and the region is present in any of the target tissue tracks of the dataset or (2b) *active-in-all* mode is selected for the dataset and the region is present in every available target tissue track of the dataset. MicroRNA (miRNA) transcript coordinates are included in the result if (1) a miRNA–target gene interaction is present in a selected dataset (i.e., miRTarBase and miRWalk) and meets the user-specified search criteria (e.g., confidence) and (2) the miRNA transcript overlaps any of the ENCODE accessible chromatin tracks for the target tissues. In the final result, coordinates of the regulatory regions and coordinates of the miRNA transcripts, selected as described above, are merged and collapsed. The result, in a form of a BED file or a table, contains information about the origin of each interval allowing to trace back the dataset and tissue. All operations on genomic coordinates were implemented using pybedtools (Dale et al., 2011).

### Variant Filtering

For user convenience, we added a variant filtering functionality to the application. It is implemented in JavaScript, works entirely in a user’s browser, and does not require sending the data over the Internet, which makes it suitable for sensitive data analysis. The feature is currently limited by the memory available to the browser, but our tests have shown that a single-sample whole-genome variant call format (VCF) file can be processed on a regular laptop within a few seconds. VCF files filtered by Remus can be further scrutinized for pathogenic variants using other means, such as population frequency data or pathogenicity scores.

### Evaluation

A subset of the pathogenic regulatory variants collected by Smedley et al. (2016) was used to evaluate Remus. We selected promoters and enhancers located up to 1 Mb away from the closest transcription start site of the affected gene. The variants were divided into three groups: (1) promoter variants, located 1–100 bp upstream of a transcript start of the affected gene; (2) intragenic, regulatory variants within the transcript of the gene, but not classified as promoter variants; and (3) distal, located more than 100 bp away from the closest transcript start of the gene. Using the original publication for each variant, we chose one tissue or one cell type affected by the disorder that best matched a track available in one of the Remus datasets (Supplementary Table 2). In addition, a list of related tissues/cell types representing a wider choice of tracks was created for each case. Coordinates of the tested variants, affected genes, and selected tissues are listed in Supplementary Table 2. Remus API was queried using this list of genes and tissues, against all *cis*-regulatory datasets with the default parameters. The number and size of the regulatory regions output by Remus for each queried gene, as well as information whether the pathogenic variant overlapped any of the identified regulators, are provided in Supplementary Table 3.

## Results

Remus^4^ is a web application and API that facilitates the identification of regulatory regions and genetic variants potentially involved in the pathogenicity of monogenic diseases. Remus uses a range of tissue-specific genome-wide datasets to identify regulatory regions active in user-selected target tissues and in proximity of the target genes (Figure 1). Dataset availability for each tissue is provided in the user interface and can suggest tracks with relevant regulatory data. Coordinates of the regulatory regions can be downloaded, viewed in a genome browser, or immediately used for in-browser filtering of variants, making it suitable for analysis of sensitive data.

**FIGURE 2:**
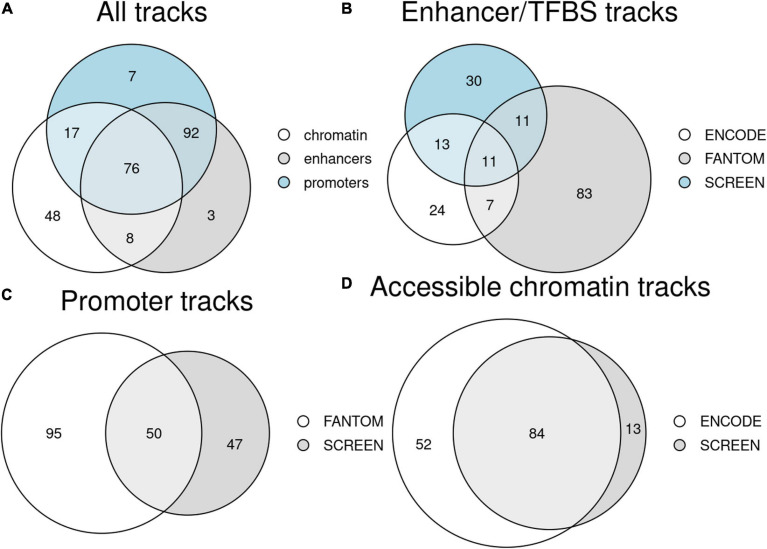
Counts of unique tissue and primary cell type tracks available in Remus. **(A)** Tracks for the three regulatory region classes (enhancers/TFBS, promoters, and accessible chromatin); *numbers in overlaps* indicate tissues/cell types with data for several regulator classes. **(B–D)** Breakdown of the tissue tracks into datasets of origin for the three regulatory region classes: enhancers/TFBS **(B)**, promoters **(C)**, and accessible chromatin **(D)**.

Analysis starts from one or more target genes, for example implicated in the pathogenesis of a disease, and a set of tissues or cell types affected by the disease (target tissues; Figure 1). After setting these, the user can choose the types of regulators to include (promoters, enhancers, repressors, insulators, open chromatin regions, and miRNAs) and a set of particular datasets to query. The datasets vary in the experimental techniques that were used to identify the regulatory elements and in tissue coverage (Figure 2). *Cis*-regulatory datasets available in Remus come from ENCODE (The Encode Project Consortium, 2012), SCREEN (ENCODE Project Consortium et al., 2020), and FANTOM (Forrest et al., 2014) repositories and represent the results of diverse genome-wide experimental assays on human primary cell types and tissues (details in “Materials and Methods” section). MicroRNA–gene interactions are available in two flavors: experimentally validated (MiRTarBase) (Chou et al., 2018) and predicted (MirWalk) (Sticht et al., 2018). Neither is tissue-specific, and to restrict the interactions to the target tissues, localization of the miRNAs interacting with the 3′-UTRs of the target genes is filtered through regions of accessible chromatin in the target tissues. Together, the available datasets represent a diverse set of *cis*- and *trans*-regulators active in the target tissues that, if mutated, could impact the expression of the input genes.

Remus includes regulatory elements in the final result based on genomic proximity to the target genes and activity status in the target tissues. Two modes of search were implemented. A permissive search will include a regulatory region if it is active in *any* of the selected target tissues (Figure 1A, example 2), and a strict search requires a region to be active in every tissue of interest (Figure 1A, example 3). The final result contains genomic coordinates of the selected regulatory regions along with information about the tracks (tissue and dataset) where they were identified.

We have provided three ways of using the result. Firstly, the identified regulatory regions can be downloaded as a BED or Excel file for further processing. Secondly, the user is provided with a direct link to the UCSC Genome Browser, where the regulatory region *loci* can be cross-checked with a number of other datasets, including, for instance, evolutionary conservation, SNP density, or TF-binding motifs. Thirdly, a user-provided variant file (VCF) can be filtered using the discovered regulatory regions to suggest regulatory variant candidates relevant for the studied phenotype.

### Summary of the Datasets

In the data preparation phase, we integrated results from 3,509 genome-wide assays and 565 regulatory tracks (Supplementary Tables 1, 4). It resulted in a total of 179 DNA-binding tracks (enhancers, repressors, and insulators), 192 promoter tracks, and 146 accessible chromatin tracks, each representing a single tissue or a cell type (Figure 2A). For 76 tissues, all three types of regulatory tracks were available, and for 168 both promoters and DNA-binding sites were present. The datasets were largely complementary in terms of the types of regulatory elements they contributed and in terms of the tissues and cell types covered (Figures 2B–D).

Due to the diverse experimental techniques used to identify the regulatory regions, the regulatory datasets differed considerably in terms of feature density and individual regulatory region characteristics. ENCODE TF-binding site and SCREEN enhancer tracks contained, on average, 66 thousands and 64 thousands active regions, while FANTOM enhancer tracks had, on average, 1.8 thousands such regions (Supplementary Figure 2). The sizes of the individual enhancers and TF-binding sites were similar across the datasets, with the majority spanning 250–500 bp. For promoters, we also noted large differences, i.e., SCREEN and FANTOM tracks had, on average, 62 thousands and 12 thousands active promoter regions per tissue, and the average promoter sizes were 582 bp and 275 bp, respectively. Accessible chromatin regions differed in average count per track (333 thousands for ENCODE and 123 thousands for SCREEN) and average sizes of individual regions (176 bp and 539 bp; Supplementary Figures 2E,F), but the average overall span was similar (60 and 66 Mb).

Only a fraction of the regulatory tracks included in Remus was originally available in both genome builds. The FANTOM and SCREEN datasets were only available in the hg19 coordinate system, and many ENCODE tracks were provided for a single genome build. Coordinate liftover of tracks available only for a single genome build (hg19 or hg38) yielded extra 36 kb–11.6 Mb regions (0.3–46% increase; median = 14%) for the hg38 TF-binding site tracks and additional 466 kb–61.6 Mb (0.6–263%; median = 4.4%) in the case of the hg38 open chromatin tracks (Supplementary Figure 3). A small fraction of the regions failed to map between the genome builds, and these were discarded from the destination genome, but not the source genome. For the hg19 tracks lifted over to hg38, 0–1.15% base pairs (median = 0.026%) failed to map, and for the hg38 to hg19 liftover, between 0.09 and 1.02% base pairs (median = 0.3%) were not lifted over.

The miRNA–gene interactions available in Remus include 380,640 miRTarBase (Chou et al., 2018) records with experimental evidence, mapped for 2,600 human miRNAs and their 15,065 target genes. On average, each miRNA targeted 146 genes (1–2,627; median = 94) and a single gene was targeted by 25 miRNAs (1–359; median = 14). A database of computationally predicted miRNA–target interactions from miRWalk (Sticht et al., 2018) was also included. It comprised over 13.5 million interactions involving 2,588 miRNAs and 18,448 genes. In this dataset, a miRNA interacted with a 3′-UTR of, on average, 5,231 genes (8–10,516; median = 5,706), and a gene was targeted by 1–2,321 miRNAs (mean = 732, median = 656).

### Tissue-Specific Analysis

**FIGURE 3:**
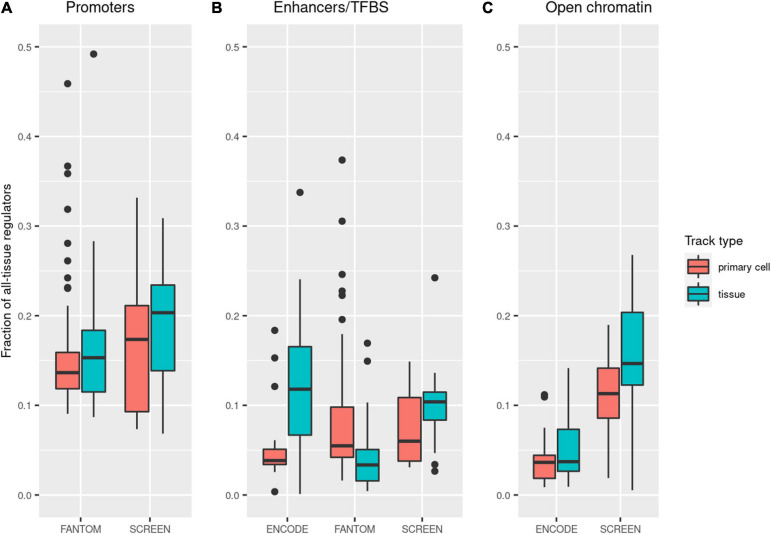
Decrease in the genomic span of the regulatory regions after limiting the analysis to a single track. The *Y*-axis presents the fraction of all regulatory regions present in a single tissue or cell type track (based on the numbers of base pairs). *Points in the figure* represent individual tissue/cell type tracks. The *boxes* show the interquartile range (IQR) of the distribution, the *middle bar* represents the median, and the *whiskers* extend to the min and max points within 1.5*IQR from quartiles Q1 and Q3, respectively.

Our main aim of using tissue-specific data for regulatory variant selection was restricting the vast regulome to the phenotype-relevant regions only. We calculated the rate in which a single tissue/cell type track limited the span of the considered regulatory regions. The rates varied between tissues and cell types and across datasets (Figure 3). As expected, reduction in the size of the regulatory regions was greater for primary cell tracks, which was the case for all datasets except FANTOM enhancers. A single tissue promoter track contained medians of 17 and 14% (SCREEN and FANTOM, respectively) of all promoter regions (Supplementary Table 5). In the case of enhancers, the rates were lower and ranged between 4.9% (FANTOM) and 8.7% (SCREEN). Selecting a single cell type enhancer track from the FANTOM, SCREEN, or ENCODE dataset allowed limiting the span of the considered regulatory elements to below 6% of the total. Open chromatin regions differed for ENCODE and SCREEN, with the former being more specific and constraining analysis to medians of 3.6 and 3.7% for primary cell and tissue tracks, respectively. The median open chromatin track from SCREEN contained 14.6% (11.7 and 17.1% for primary cells and tissues) of the total open chromatin regions in this dataset.

### Evaluation

To evaluate Remus in searching pathogenic mutations, we used a set of 146 regulatory variants reported causative for 68 distinct monogenic disorders (Smedley et al., 2016) and a manually curated list of tissues affected by these disorders (Supplementary Table 2). The search was performed in two steps: firstly, for a single tissue or cell type that best matched the phenotype and, secondly, for a wider set of tissues related to the phenotype (e.g., single-tissue search for regulatory elements in the brain was expanded to include the midbrain and astrocyte tracks as well). In the control test, regulators in all tissues were queried (unselected search). The results were broken down into promoters, intragenic, and distal regulators (details in the “Materials and Methods” section). Coordinates of the regulatory variants, genes, and tissues selected for the test were listed in Supplementary Table 2.

Overall, the set of regulators identified by Remus for a single tissue comprised 1–909 regions (median = 98) spanning 110 bp–175.7 kb (median = 44.2 kb) and included the pathogenic variant in 63 of 78 cases (80.7% recall; Table 1). In the multi-tissue test, 5–1,010 (median = 254) regulators were found covering 3.2 kb–296.5 kb (median = 98.4 kb) of the genome sequence. The causative variant was among the identified regulators in 70 of 78 cases (89.7%). In the control, tissue-unselected test, the pathogenic variant was identified in four more cases (94.9% recall). Specificity of the search was measured as the fraction of the tissue-unselected result excluded by performing a tissue-specific query. For single- and multiple-tissue searches, it equaled to 92.7 and 82.5%, respectively. The identified regulatory regions and their sizes are available in Supplementary Table 3.

**TABLE 1:** Sensitivity and specificity tested on 146 regulatory mutations in 68 monogenic disorders.

		**Single tissue**				**Multiple tissues**					**Unselected**	
		
		**Count**	**Size (kb)**	**Specificity (%)**	**Sensitivity**	**Count**	**Size (kb)**	**Specificity (%)**	**Identified**	**Count**	**Size (kb)**	**Identified**
		
	Min	1	0.1	63.4		33	17.7	56.6		645	370.7	
							
Distal regulators	Mean	135	48.2	91.6	18/26	334	105.6	82.1	20/26	1237	554.7	23/26
							
	Median	97	42.7	93.3	(69.2%)	272	93.1	80.9	(76.9%)	1268	561.6	(88.5%)
							
	Max	909	175.7	100.0		1010	228.7	96.7		1676	801.8	

	Min	2	0.4	75.7		5	3.2	56.9		645	370.7	
							
Intragenic regulators	Mean	156	64.7	89.9	15/21	374	143.1	77.2	19/21	1,237	554.7	20/21
							
	Median	88	40.6	93.8	(71.4%)	406	148.2	75.9	(90.5%)	1,268	561.6	(95.2%)
							
	Max	796	170.3	99.9		874	296.5	99.5		1,676	801.8	

	Min	2	0.7	63.4		26	17.4	56.6		748	373.6	
							
Promoters	Mean	138	56.9	89.8	30/31	269	99.8	82.8	31/31	1,267	554.6	31/31
							
	Median	98	47.0	91.6	(96.8%)	210	92.8	86.8	(100%)	1,268	534.7	(100%)
							
	Max	909	175.7	99.9		1,010	240.9	97.9		1,676	826.3	

Overall	Median	98	44.2	92.73	63/78 (80.7%)	254	98.4	82.49	70/78 (89.7%)	1,265	561.6	74/78 (94.9%)

*Analyses for single target tissue and multiple target tissues are contrasted with the control (tissue-unselected) search. Mutation categories are broken down into mutations of promoters, intragenic regulators, and distal regulators (see “Materials and Methods” section). Specificity of the search was measured as the fraction of the tissue-unselected result excluded by performing a tissue-specific query.*

Out of four regulatory mutations missing from Remus tissue-specific results, and identified by the control tissue-unselected search, two were in enhancers of *TBX5* and *HBA2* (139 kb and 13 kb away from the nearest TSS, respectively), one in a distal promoter of ACTN4 located 445 bp away from the gene start, and one in the intron of KCNJ11. They were found in tracks unrelated or vaguely related to the phenotype of the disorder caused by the mutation, e.g., *HBA2* enhancer overlapped only the stomach accessible chromatin track from ENCODE, and the *ACTN4* promoter causing focal segmental glomerulosclerosis was found in ENCODE TF-binding site tracks of erythroblasts, liver, gastroesophageal sphincter, and accessible chromatin in uterus.

Breakdown of the regulatory categories showed a markable difference in sensitivity between the regulators proximal to transcription start site (promoters; recall of 96.8% in single-tissue tests) and those that were further away, such as distal and intragenic regulators (recall of 69.2 and 71.4%, respectively). In contrast, the difference in the median specificity for the single-tissue analysis was marginal (specificity = 91.6–93.8%).

To present the use of Remus in a specific case, we chose an example of monogenic diabetes of the young (MODY), a rare congenital type of diabetes responsible for 1–4% of the diabetes worldwide (Sztromwasser et al., 2019). MODY is caused by malfunctioning beta cells of the pancreas, and several genes have been involved in the pathogenesis of the disease. One of the genes is *BLK*, for which an enhancer located 19.7 kb upstream of the gene has also been implicated in the pathogenesis (Borowiec et al., 2009). To contrast Remus’ tissue-specific search with an unspecific screen for regulatory elements, we performed two analyses. Firstly, we queried regulators active in all tissues and located up to 50 kb upstream and downstream of *BLK*’s transcription start site. In this analysis, Remus identified 89 regulatory regions covering a total of 87.2 kb. In the second analysis, we tried to use the cell types affected by MODY, and since pancreatic beta cell tracks were not available, we used pancreas as the target tissue. An equally parameterized search restricted to regulators active in the pancreas resulted in nine regulatory regions covering 4.7 kb, which corresponded to 5.3% of the regions resulting from the tissue-unselected search. Reduction in the span of the considered regulatory region should translate directly into a lower number of regulatory variant candidates. To confirm, this we looked at a set of 10 whole-genome samples, where we observed a drop in the number of candidate regulatory single nucleotide variant (SNV) from 204–292 to 10–14 per sample, illustrating that the decrease in the number of regulatory variants is proportional to the reduction in the span of regulatory regions.

## Discussion

In this work we present Remus, a new web server that integrates multiple regulatory datasets from several sources to facilitate search for regulatory variants with a putative role in monogenic disorder pathogenesis. Using a simple user interface, the tool enables tissue-specific queries across a range of regulatory element types: promoters, enhancers, transcription factor binding sites, as well as miRNA–gene interactions. Aggregation and processing of regulatory data allowed us to compare the datasets and identify differences stemming from the experimental techniques used to obtain them. For instance, the FANTOM datasets are based on CAGE-seq data, where a sharp signal spanning a few tens of base pairs represents a transcription initiation event (Kodzius et al., 2006). Promoter regions obtained from CAGE peaks are thus narrow and located precisely at transcription start sites. In contrast, SCREEN promoters were mapped by overlapping open chromatin regions (DNase-seq footprinting) located upstream of annotated genes, with histone modifications promoting transcription (ChIP-seq) (The Encode Project Consortium, 2012). DNase footprints are wider than CAGE-seq peaks and, despite being methylated, may not necessarily be actively transcribed, which explains the modest overlap between the two datasets (Supplementary Figure 4).

Similar differences stemming from experimental techniques can be observed for the enhancer and TF-binding datasets (Supplementary Figure 3). FANTOM enhancers mapped by CAGE-seq are transcribed enhancers—a subset of all active enhancers in a cell (Andersson et al., 2014). This explains their small count when compared to the other two enhancer datasets. SCREEN enhancers, similarly to SCREEN promoters, are based on open chromatin regions with methylation signal-promoting activity. In contrast, the ENCODE TF-binding tracks obtained in ChIP-seq experiments represent genome-wide footprints of TFs (including *CTCF* repressor), a wide set of regulators that both promote and repress gene activity (Supplementary Figure 4).

The diversity of the experimental techniques represented in the regulatory datasets available in Remus facilitates tuning the sensitivity and specificity of the search. By using only the FANTOM data, the user gets more sparse regulatory regions with clear evidence of RNA polymerase binding. Including enhancer, TF-binding datasets, and open chromatin regions gradually expands the search area, likely increasing the rate of regulatory elements with weaker impacts on gene expression. Further sensitivity and specificity adjustment can be made on the tissue level—the search protocol allows querying regulators by activity in any or all tissues in the user-specified set of tissues relevant for the phenotype.

The option of tuning sensitivity by modifying the set of target tissues was illustrated in the experiment that evaluated the efficacy of Remus. In a strict single-tissue search, Remus identified over 80% of regulators with a role in the disease, while the span of considered *loci* was decreased over 10-fold (92% specificity). A permissive multi-tissue query resulted in, on average, less than 20% of all the regulators in the proximity of the target genes and was able to identify causative regulators in all but four cases present in the datasets. This demonstrated that a targeted search for regulatory variants can be sensitive and at the same time yield comprehensible numbers of candidate mutations for evaluation. Noteworthy is that variants in the core promoter regions were identified with considerably higher sensitivity than distal regulators, especially when a single tissue was used for searching. This is explained by the fact that the core promoters are more likely to be active and detected by Remus in several tissues, while distal regulators act frequently in a tissue-specific manner. In consequence, the search for promoter variants is less dependent on the choice of tissue of interest.

The regulators missed by Remus’ tissue-focused search were either not present in the regulatory datasets we aggregated or were present in tracks not related to the tissues affected by the mutation of the regulator. This highlights the dependence of the results on the quality and extent of the underlying data and indicates that, in isolated cases, known regulatory regions can be missing from the genome-wide regulatory tracks. Similarly, regulatory data may not be available for specific tissues and developmental stages, as exemplified by the *PTF1A* enhancer active in pancreatic progenitor cells (Weedon et al., 2014) that was not present in any of the tracks included in Remus.

Access to the regulatory data aggregated in Remus is provided through a programmatic interface and a web interface, where a user can easily query the regulatory elements and filter variants. The latter, a seemingly minor feature, due to the way it was implemented, does not require transferring variant files over the Internet and can be safely used to filter sensitive data in a user’s own browser. In a setting where data privacy is a priority or a legal requirement (Shabani and Borry, 2018), this lifts the access barriers for using the public Remus instance. Although the functionality is limited by a computer’s memory available to a web browser, typical whole-genome variant files (VCF) for a single patient are well within these limits.

As a web application, Remus lifts the access barriers for its users—it does not require installation, downloading of large datasets, or complex configuration. We found one other software that offers a web-based identification of regulatory variants with a putative role in disease. IPEV (Zhang et al., 2019) classifies user-uploaded variants using a random forest classifier trained on the DiseaseEnhancer (Zhang et al., 2018) database. The tool is limited to enhancers and the classification procedure requires 20 min to analyze 100 variants, making it unsuitable for whole-genome analysis. Additionally, the analysis involves uploading the variants, which may be undesirable in case of sensitive patient data. Among other web applications that support regulatory variant analysis, the HEDD database offers rich functionality targeted mainly at studying complex diseases and gene enhancer networks (Wang et al., 2018). The database offers a subset of the regulatory datasets available in Remus, but coordinates of the regulators for target genes can be queried in individual datasets and downloaded for local filtering of variants. Other resources, such as GeneHancer (Fishilevich et al., 2017), SlideBase (Ienasescu et al., 2016), and generic genome browsers (Kent et al., 2002; Zerbino et al., 2018), are limited in the scope of regulatory features and datasets provided and, offer only manual browsing of individual regulators or bulk data download for programmatic use.

The approach implemented in Remus has several limitations. One is certainly the availability of the regulatory data for specific cell types and developmental stages. This shortcoming may be, however, compensated in the future as more tissues and cell types are profiled for regulatory regions. Another limitation is the identification of the regulators only by proximity to the TSS of the gene and, implicitly, tissue co-activity. It should be expected that a portion of the identified regulatory regions have no influence on the expression of the gene in question, but regulate other genes in the region. This is a minor issue for shortlisting candidate variants for a rare disease as these need to be scrutinized with the help of other means, such as population frequency or pathogenicity scores. As Remus is currently not able to annotate or filter variants using these annotations, we recommend pre-filtering the variants using other tools (Wang et al., 2010; McLaren et al., 2016) in advance.

However, it is worth noting that the rate of such false-positive hits could be reduced by linking distal regulators to genes by tissue co-expression or expression quantitative trait loci (eQTLs). Ideally, relevant regulators should be identified using tissue-specific topologically associated domain (TAD) regions, but to our knowledge, no large public datasets of this kind exist.

Application of the Remus methodology to studying the pathogenesis of rare disorders could suggest that an alteration in an actively used regulator of a disease-causing gene could be equally deleterious as a mutation of the gene itself. As this can be the case for dosage-sensitive genes, in many situations, other (alternative) regulatory elements could be sufficient to maintain gene expression on high-enough levels. Also, the assumption that a phenotype affecting a particular organ is caused by a mutation in a gene or a regulatory element active in that part of the body fails to accommodate disorders where the effect of the mutation is indirect, e.g., hyperammonemia, caused by mutations in liver enzymes, manifests in urea accumulation in the blood (Williams et al., 2018). And finally, only mutations of existing regulatory elements can be identified by Remus. Gain-of-function mutations resulting in new transcription factor binding sites or promoters would be missed, unless they fall within an existing regulatory region.

## Conclusion

We developed Remus, a web server that facilitates targeted screening for candidate regulatory variants in whole-genome sequencing data. It integrates regulatory features from multiple sources, allows tissue-specific queries in a simple web interface, and provides a filtering functionality suited for sensitive data. As shown on a set of known pathogenic regulatory variants, a tissue-specific search reduced the number of candidate variants by an order of magnitude while missing only a small fraction of the causative variants. We expect that Remus will prove useful when revisiting undiagnosed cases with available WGS data, as well as in analyses of new patients without findings in protein-coding genes. Expanding the variant search beyond the coding sequence will likely yield candidate variants and may improve the diagnostic rate of whole-genome sequencing in monogenic disorders.

## Data Availability Statement

The original contributions presented in the study are included in the article/Supplementary Material, further inquiries can be directed to the corresponding author/s.

## Author Contributions

PS and WF designed the project. PS and DS implemented the software. PS aggregated and analyzed the regulatory data and wrote the manuscript. PS and AM performed the evaluation experiments. PS, AM, and WF tested the final version of the software. All authors accepted the final version of the manuscript.

## Conflict of Interest

The authors declare that the research was conducted in the absence of any commercial or financial relationships that could be construed as a potential conflict of interest.

## Footnotes

1 https://getbootstrap.com

2 https://github.com/seru71/Remus

3 https://github.com/seru71/remus_manus

4 https://remus.btm.umed.pl

## Supplementary Material

Supplementary Figure 1 Illustration of track collapsing and liftover. Original tracks for the same tissue (biological replicates) and in the same genome build were merged. Next, coordinates were lifted over to the other genome build (i.e., hg19 to hg38, and *vice versa*), and merging on the same tissues was performed again.

Supplementary Figure 2 Numbers and sizes of the enhancer/TFBS **(A,B)**, promoter **(C,D)**, and accessible chromatin regions **(E,F)** originating from the different datasets. In the figure the numbers are divided between tissue and celltype tracks. When considered together, ENCODE TF-binding site and SCREEN enhancer tracks contained on average 66 thousands and 64 thousands active regions, while FANTOM enhancer tracks had on average 1.8 thousands such regions. Sizes of individual enhancers and TF-binding sites were similar across the datasets with majority spanning 250–500 bp. Promoter tracks from SCREEN and FANTOM had on average 62 thousands and 12 thousands regions per tissue, and average promoter sizes were 582 bp and 275 bp, respectively. Accessible chromatin regions differed in average count per track (333 thousands for ENCODE and 123 thousands for SCREEN) and average sizes of individual regions (176 bp and 539 bp), but the average overall span was similar (60 and 66 Mb; not shown in the figure). Points in the figure represent individual tissue/celltype tracks. The boxes show the interquartile range (IQR) of the distribution, the middle bar represents the median, and the whiskers extend to the min and max points within 1.5^∗^IQR from quartiles Q1 and Q3, respectively.

Supplementary Figure 3 Percentage gain in genomic span of the ENCODE TFBS and accessible chromatin regions as a result of merging hg38 tracks with the tracks available only in hg19 coordinate system (after liftover to hg38). Points in the figure represent individual tissue/celltype tracks. The boxes show the interquartile range (IQR) of the distribution, the middle bar represents the median, and the whiskers extend to the min and max points within 1.5^∗^IQR from quartiles Q1 and Q3, respectively.

Supplementary Figure 4 Overlap between promoter tracks **(A)** and accessible chromatin tracks **(B)** for the same tissue or celltype obtained using different assays. FANTOM promoters originate from CAGE-seq, ENCODE accessible chromatin from TF-ChIP seq, and SCREEN promoters and open chromatin from DNase-seq experiments. The overlap for a single track was calculated as a fraction of basepairs overlapping the same tissue/celltype track in the compared dataset, e.g., a median of 83.4% of a FANTOM tissue promoter track overlaps its equivalent in the SCREEN dataset, while a median of 8.7% of a SCREEN tissue track is present in equivalent track from the FANTOM dataset.

Supplementary Table 1 The list of files used to create Remus database.

Supplementary Table 2 Manually curated list of gene-tissue pairs.

Supplementary Table 3 Results of the evaluation experiment.

Supplementary Table 4 Numbers of tissue and celltype tracks available in Remus.

Supplementary Table 5 Genomic span and specificity of tracks in the available datasets.

## Abbreviations

API: application programming interface
CAGE: cap analysis of gene expression
ChIP: chromatin immunoprecipitation
DNase: deoxyribonuclease
FAIRE: formaldehyde-assisted isolation of regulatory elements
HGNC: HUGO Gene Nomenclature Committee
RLE: relative log expression (normalization method)
TF: transcription factor
TFBS: transcription factor binding site
TSS: transcription start site
WGS: whole-genome sequencing.
