# Supplementary material for: Remus: A Web Application for Prioritization of Regulatory Regions and Variants in Monogenic Diseases
Source: Front Genet. 2021 Mar 5;12:638960. doi: 10.3389/fgene.2021.638960 (PMC7978111; doi:10.3389/fgene.2021.638960)
Supplement: Supplementary Figure 1 — Illustration of track collapsing and liftover. Original tracks for the same tissue (biological replicates) and in the same genome build were merged. Next, coordinates were lifted over to the other genome build (i.e., hg19 to hg38, and vice versa), and merging on the same tissues was performed again. [file Data_Sheet_1.zip › Supplementary Material/Data Sheet 1.pdf]

Supplementary Table 2

Category	Chr	Position	Gene	First_choice_tissue	Wide_choice_tissues	Distance_from_TSS [bp]
distal	chr7	156061506	SHH	brain	brain;midbrain;astrocyte of the hippocampus;astrocyte of the cerebellum;astrocyte of the spinal cord;astrocyte	456538
distal	chr11	31685945	PAX6	retina	eye;retina;retinal pigment epithelial cell	139841
distal	chr12	114704515	TBX5	heart	heart;heart right ventricle;heart left ventricle	139454
distal	chrX	105251968	SERPINA7	liver	liver;right lobe of liver;hepatocyte;hepatic stellate cell	30751
distal	chr10	23508446	PTF1A	pancreas	pancreas;body of pancreas;endocrine pancreas	26987
distal	chr10	23508437	PTF1A	pancreas	pancreas;body of pancreas;endocrine pancreas	26978
distal	chr10	23508365	PTF1A	pancreas	pancreas;body of pancreas;endocrine pancreas	26906
distal	chr10	23508363	PTF1A	pancreas	pancreas;body of pancreas;endocrine pancreas	26904
distal	chr10	23508305	PTF1A	pancreas	pancreas;body of pancreas;endocrine pancreas	26846
distal	chr8	11331747	BLK	pancreas	pancreas;body of pancreas;endocrine pancreas	19753
distal	chr16	209709	HBA2	erythroblast	stromal cell of bone marrow;erythroblast	13136
distal	chr1	209989478	IRF6	kidney	kidney;kidney epithelial cell;epithelial cell of proximal tubule;kidney capillary endothelial cell;left kidney;right kidney;kidney tubule cell;renal pelvis;left renal pelvis;right renal pelvis;renal cortical epithelial cell;renal cortex interstitium;left renal cortex interstitium;right renal cortex interstitium; glomerular endothelial cell	9957
distal	chr17	42078968	NAGS	liver	liver;right lobe of liver;hepatocyte;hepatic stellate cell;small intestine;intestinal epithelial cell	3063
distal	chr7	117119337	CFTR	lung	lung;respiratory epithelial cell;tracheal epithelial cell;pancreas;liver;colonic mucosa;intestinal epithelial cell	679
distal	chr19	39137810	ACTN4	glomerular endothelial cell	kidney;kidney epithelial cell;epithelial cell of proximal tubule;kidney capillary endothelial cell;left kidney;right kidney;kidney tubule cell;renal pelvis;left renal pelvis;right renal pelvis;renal cortical epithelial cell;renal cortex interstitium;left renal cortex interstitium;right renal cortex interstitium; glomerular endothelial cell	445

distal	chrX	153237261	HCFC1	astrocyte	neuron;neuronal stem cell;neurosphere;brain;midbrain;astrocyte of the hippocampus;astrocyte of the cerebellum;astrocyte of the spinal cord;astrocyte	441
distal	chr19	49468350	FTL	erythroblast	blood;peripheral blood mononuclear cell;erythroblast;granulocyte	215
distal	chr11	5276213	HBG2	reticulocyte	blood;peripheral blood mononuclear cell;erythroblast;granulocyte;reticulocyte	201
distal	chr11	5271283	HBG1	blood	blood;peripheral blood mononuclear cell;erythroblast;granulocyte	195
distal	chr3	48632780	COL7A1	skin fibroblast	foreskin keratinocyte;foreskin fibroblast;fibroblast of skin of abdomen;foreskin melanocyte;fibroblast of skin of left biceps;fibroblast of skin of left quadriceps;fibroblast of skin of right quadriceps;fibroblast of skin of scalp;fibroblast of skin of right biceps;fibroblast of skin of upper back;lower leg skin;fibroblast of skin of back;skin fibroblast embryonic;skin fibroblast;fibroblast of skin of abdomen embryonic;skin of body embryonic;skin of body;suprapubic skin	186
distal	chr20	42984264	HNF4A	pancreas	pancreas;body of pancreas;endocrine pancreas	176
distal	chr11	5276186	HBG2	reticulocyte	blood;peripheral blood mononuclear cell;erythroblast;granulocyte;reticulocyte	174
distal	chr20	42984276	HNF4A	pancreas	pancreas;body of pancreas;endocrine pancreas	164
distal	chrX	38211584	OTC	liver	liver;right lobe of liver;hepatocyte;hepatic stellate cell	151
distal	chr20	42984299	HNF4A	pancreas	pancreas;body of pancreas;endocrine pancreas	141
distal	chr11	67250359	AIP	brain	brain;midbrain;cerebellar cortex;cerebellum;astrocyte of the hippocampus;astrocyte of the cerebellum;astrocyte of the spinal cord;astrocyte	140
distal	chr9	104198194	ALDOB	liver	liver;right lobe of liver;hepatocyte;hepatic stellate cell	131
distal	chr20	42984309	HNF4A	pancreas	pancreas;body of pancreas;endocrine pancreas	131
distal	chr11	17498513	ABCC8	pancreas	pancreas;body of pancreas;endocrine pancreas	120
distal	chrX	55057617	ALAS2	reticulocyte	stromal cell of bone marrow;erythroblast	119

distal	chrX	135730217	CD40LG	naive thymus-derived CD4-positive, alpha-beta T cell	CD4-positive, alpha-beta T cell;naive thymus-derived CD4-positive, alpha-beta T cell;CD4-positive helper T cell;CD4-positive, CD25-positive, alpha-beta regulatory T cell;CD4-positive, alpha-beta memory T cell	118
distal	chr11	5271204	HBG1	blood	blood;peripheral blood mononuclear cell;erythroblast;granulocyte	116
distal	chr11	5276125	HBG2	reticulocyte	blood;peripheral blood mononuclear cell;erythroblast;granulocyte;reticulocyte	113
distal	chr11	5271201	HBG1	blood	blood;peripheral blood mononuclear cell;erythroblast;granulocyte	113
distal	chr1	171621877	MYOC	eye	retina;retina embryonic;retinal pigment epithelial cell;eye	103
distal	chr11	2182543	INS	pancreas	pancreas;body of pancreas;endocrine pancreas	103
distal	chrX	146993366	FMR1	astrocyte	neuron;neuronal stem cell;neurosphere;brain;midbrain;astrocyte of the hippocampus;astrocyte of the cerebellum;astrocyte of the spinal cord;astrocyte	102
intragenic	chr1	21890663	ALPL	osteoblast	liver;right lobe of liver;kidney;left arm bone;left leg bone;right arm bone;right leg bone;osteoblast	54813
intragenic	chr3	12421189	PPARG	adipose tissue	adipose tissue;subcutaneous adipose tissue;subcutaneous abdominal adipose tissue;intestinal epithelial cell;large intestine;small intestine	28189
intragenic	chr10	127505271	UROS	erythroblast	erythroblast	6567
intragenic	chr10	127505287	UROS	erythroblast	erythroblast	6551
intragenic	chr10	127505291	UROS	erythroblast	erythroblast	6547
intragenic	chrX	55054635	ALAS2	reticulocyte	stromal cell of bone marrow;erythroblast;reticulocyte	2863
intragenic	chr8	11560787	GATA4	heart	cardiac muscle cell;left cardiac atrium;right cardiac atrium;heart;heart left ventricle;heart right ventricle	872
intragenic	chr8	11560864	GATA4	heart	cardiac muscle cell;left cardiac atrium;right cardiac atrium;heart;heart left ventricle;heart right ventricle	795
intragenic	chr5	112073008	APC	colonic mucosa	colonic mucosa;sigmoid colon;transverse colon	547
intragenic	chr2	219524871	BCS1L	skin fibroblast	skin fibroblast;fibroblast of dermis;skeletal muscle tissue;skeletal muscle cell;skeletal muscle myoblast;	493
intragenic	chr11	17409772	KCNJ11	pancreas	pancreas;body of pancreas;endocrine pancreas;brain;neuron	435

intragenic	chr8	11561283	GATA4	heart	cardiac muscle cell;left cardiac atrium;right cardiac atrium;heart;heart left ventricle;heart right ventricle	376
intragenic	chr8	11561369	GATA4	heart	cardiac muscle cell;left cardiac atrium;right cardiac atrium;heart;heart left ventricle;heart right ventricle	290
intragenic	chr10	89623462	PTEN	breast epithelium	breast epithelium;epithelial cell of prostate;thyroid gland;colonic mucosa;sigmoid colon;transverse colon	268
intragenic	chr8	11561399	GATA4	heart	cardiac muscle cell;left cardiac atrium;right cardiac atrium;heart;heart left ventricle;heart right ventricle	260
intragenic	chr1	8021919	PARK7	brain	brain;caudate nucleus;neuron;liver;right lobe of liver;hepatocyte;skeletal muscle tissue;skeletal muscle cell;kidney	206
intragenic	chr10	89623373	PTEN	breast epithelium	breast epithelium;epithelial cell of prostate;thyroid gland;colonic mucosa;sigmoid colon;transverse colon	179
intragenic	chr10	89623365	PTEN	breast epithelium	breast epithelium;epithelial cell of prostate;thyroid gland;colonic mucosa;sigmoid colon;transverse colon	171
intragenic	chrX	100641044	BTK	lymphocyte of B lineage	lymphocyte of B lineage;common myeloid progenitor, CD34-positive	169
intragenic	chr1	228337561	GJC2	brain	dendritic cell;neuron;brain	147
intragenic	chrX	70443185	GJB1	liver	liver;right lobe of liver;hepatocyte;neuron	130
intragenic	chr8	41655260	ANK1	erythroblast	erythroblast	119
intragenic	chr9	35657917	RMRP	liver	liver;hepatocyte;kidney embryonic	99
intragenic	chr12	121416448	HNF1A	hepatocyte	hepatocyte;liver;right lobe of liver;kidney;left kidney;right kidney;pancreas;body of pancreas;endocrine pancreas	78
intragenic	chr12	121416446	HNF1A	hepatocyte	hepatocyte;liver;right lobe of liver;kidney;left kidney;right kidney;pancreas;body of pancreas;endocrine pancreas	76
intragenic	chr12	121416444	HNF1A	hepatocyte	hepatocyte;liver;right lobe of liver;kidney;left kidney;right kidney;pancreas;body of pancreas;endocrine pancreas	74
intragenic	chr9	35657945	RMRP	liver	liver;hepatocyte;kidney embryonic	71
intragenic	chrY	2655719	SRY	testis	testis;testis embryonic	64
intragenic	chr19	11200089	LDLR	liver	liver;right lobe of liver;hepatocyte;adrenal gland;bronchial epithelial cell	52
intragenic	chr19	11200087	LDLR	liver	liver;right lobe of liver;hepatocyte;adrenal gland;bronchial epithelial cell	50

intragenic	chr13	60738072	DIAPH3	peripheral blood mononuclear cell	peripheral blood mononuclear cell	48
intragenic	chrX	70443099	GJB1	liver	liver;right lobe of liver;hepatocyte;neuron	44
intragenic	chr19	11200073	LDLR	liver	liver;right lobe of liver;hepatocyte;adrenal gland;bronchial epithelial cell	36
intragenic	chr13	113760124	F7	liver	liver;right lobe of liver;hepatocyte	23
intragenic	chr18	77748580	TXNL4A	kidney embryonic	mesenchymal cell;kidney embryonic	13
promoter	chr2	47630106	MSH2	sigmoid colon	colonic mucosa;sigmoid colon;transverse colon;uterine smooth muscle cell;uterus	99
promoter	chr3	169482947	TERC	stromal cell of bone marrow	stromal cell of bone marrow	98
promoter	chr7	117119923	CFTR	lung	lung;respiratory epithelial cell;tracheal epithelial cell;pancreas;liver;colonic mucosa;intestinal epithelial cell	93
promoter	chr11	2182533	INS	pancreas	pancreas;body of pancreas;endocrine pancreas	93
promoter	chr11	2182532	INS	pancreas	pancreas;body of pancreas;endocrine pancreas	92
promoter	chr10	71075518	HK1	erythroblast	brain;neuron;erythroblast; peripheral blood mononuclear cell	91
promoter	chr11	5248391	HBB	erythroblast	stromal cell of bone marrow;erythroblast	89
promoter	chr11	5248389	HBB	erythroblast	stromal cell of bone marrow;erythroblast	87
promoter	chr11	5248389	HBB	erythroblast	stromal cell of bone marrow;erythroblast	87
promoter	chr11	5248388	HBB	erythroblast	stromal cell of bone marrow;erythroblast	86
promoter	chr11	5248388	HBB	erythroblast	stromal cell of bone marrow;erythroblast	86
promoter	chr11	5248388	HBB	erythroblast	stromal cell of bone marrow;erythroblast	86
promoter	chrX	154251084	F8	liver	endothelial cell of hepatic sinusoid;liver;right lobe of liver;hepatocyte;blood vessel endothelial cell	85
promoter	chr17	4806454	CHRNE	skeletal muscle tissue	forelimb muscle;hindlimb muscle;muscle of arm;muscle of back;muscle of leg;muscle of trunk;psoas muscle;skeletal muscle cell;skeletal muscle myoblast;skeletal muscle tissue;smooth muscle tissue	84
promoter	chr17	4806453	CHRNE	skeletal muscle tissue	forelimb muscle;hindlimb muscle;muscle of arm;muscle of back;muscle of leg;muscle of trunk;psoas muscle;skeletal muscle cell;skeletal muscle myoblast;skeletal muscle tissue;smooth muscle tissue	83

promoter	chrX	154251082	F8	liver	endothelial cell of hepatic sinusoid;liver;right lobe of liver;hepatocyte;blood vessel endothelial cell	83
promoter	chr12	121416289	HNF1A	hepatocyte	hepatocyte;liver;right lobe of liver;kidney;left kidney;right kidney;pancreas;body of pancreas;endocrine pancreas	81
promoter	chr7	31003560	GHRHR	brain	brain	75
promoter	chr11	5248374	HBB	erythroblast	stromal cell of bone marrow;erythroblast	72
promoter	chr11	5248372	HBB	erythroblast	stromal cell of bone marrow;erythroblast	70
promoter	chr2	234668851	UGT1A1	liver	liver;righ lobe of liver;hepatocyte	67
promoter	chrX	146993405	FMR1	astrocyte	neuron;neuronal stem cell;neurosphere;brain;midbrain;astrocyte of the hippocampus;astrocyte of the cerebellum;astrocyte of the spinal cord;astrocyte	63
promoter	chr11	116708365	APOA1	liver	liver;right lobe of liver;hepatocyte;small intestine	62
promoter	chr14	73603081	PSEN1	heart	heart;heart left ventricle;heart right ventricle;heart embryonic;brain;neuron	61
promoter	chr3	169482906	TERC	stromal cell of bone marrow	stromal cell of bone marrow	57
promoter	chr13	52585683	ATP7B	liver	liver;right lobe of liver;kidney;left kidney;right kidney;brain	52
promoter	chr1	173886568	SERPINC1	liver	liver;right lobe of liver;hepatocyte	51
promoter	chr11	2193087	TH	brain	brain;substantia nigra	51
promoter	chr11	2193086	TH	brain	brain;substantia nigra	50
promoter	chr11	2193085	TH	brain	brain;substantia nigra	49
promoter	chr17	3539712	CTNS	kidney	kidney;thyroid gland;pancreasendocrine pancreas;brain	49
promoter	chr17	3539712	CTNS	kidney	kidney;thyroid gland;pancreasendocrine pancreas;brain	49
promoter	chrX	154251048	F8	liver	endothelial cell of hepatic sinusoid;liver;right lobe of liver;hepatocyte;blood vessel endothelial cell	49
promoter	chrX	154251045	F8	liver	endothelial cell of hepatic sinusoid;liver;right lobe of liver;hepatocyte;blood vessel endothelial cell	46
promoter	chrX	154251045	F8	liver	endothelial cell of hepatic sinusoid;liver;right lobe of liver;hepatocyte;blood vessel endothelial cell	46
promoter	chr1	155271269	PKLR	erythroblast	erythroblast;liver;right lobe of liver;hepatocyte	43
promoter	chr6	118869382	PLN	heart	heart;heart left ventricle;heart right ventricle;skeletal muscle tissue;skeletal muscle cell	43

promoter	chr13	20767158	GJB2	mammary epithelial cell	mammary epithelial cell	43
promoter	chr17	3539720	CTNS	kidney	kidney;thyroid gland;pancreasendocrine pancreas;brain	41
promoter	chr13	113760062	F7	liver	liver;right lobe of liver;hepatocyte	39
promoter	chr16	56995796	CETP	liver	liver;right lobe of liver;hepatocyte	38
promoter	chr1	155271258	PKLR	erythroblast	erythroblast;liver;right lobe of liver;hepatocyte	32
promoter	chr7	117119984	CFTR	lung	lung;respiratory epithelial cell;tracheal epithelial cell;pancreas;liver;colonic mucosa;intestinal epithelial cell	32
promoter	chr11	5248333	HBB	erythroblast	stromal cell of bone marrow;erythroblast	31
promoter	chr13	48877851	RB1	retina	retina;retina embryonic;retinal pigment epithelial cell	31
promoter	chr11	5248332	HBB	erythroblast	stromal cell of bone marrow;erythroblast	30
promoter	chr11	5248331	HBB	erythroblast	stromal cell of bone marrow;erythroblast	29
promoter	chr11	5248331	HBB	erythroblast	stromal cell of bone marrow;erythroblast	29
promoter	chr11	5248330	HBB	erythroblast	stromal cell of bone marrow;erythroblast	28
promoter	chr11	5248329	HBB	erythroblast	stromal cell of bone marrow;erythroblast	27
promoter	chr11	5248329	HBB	erythroblast	stromal cell of bone marrow;erythroblast	27
promoter	chr13	48877856	RB1	retina	retina;retina embryonic;retinal pigment epithelial cell	26
promoter	chr13	48877856	RB1	retina	retina;retina embryonic;retinal pigment epithelial cell	26
promoter	chrX	70443029	GJB1	liver	liver;right lobe of liver;hepatocyte;neuron	26
promoter	chrX	70443029	GJB1	liver	liver;right lobe of liver;hepatocyte;neuron	26
promoter	chrX	138612869	F9	liver	liver;right lobe of liver;hepatocyte	25
promoter	chrX	138612869	F9	liver	liver;right lobe of liver;hepatocyte	25
promoter	chrX	138612869	F9	liver	liver;right lobe of liver;hepatocyte	25
promoter	chr11	5248326	HBB	erythroblast	stromal cell of bone marrow;erythroblast	24
promoter	chrX	70443031	GJB1	liver	liver;right lobe of liver;hepatocyte;neuron	24
promoter	chrX	146993444	FMR1	astrocyte	neuron;neuronal stem cell;neurosphere;brain;midbrain;astrocyte of the hippocampus;astrocyte of the cerebellum;astrocyte of the spinal cord;astrocyte	24
promoter	chr8	41655164	ANK1	erythroblast	erythroblast	23
promoter	chrX	138612871	F9	liver	liver;right lobe of liver;hepatocyte	23
promoter	chr13	48877860	RB1	retina	retina;retina embryonic;retinal pigment epithelial cell	22
promoter	chrX	138612872	F9	liver	liver;right lobe of liver;hepatocyte	22
promoter	chrX	138612872	F9	liver	liver;right lobe of liver;hepatocyte	22

promoter	chr19	45449218	APOC2	liver	liver;right lobe of liver;hepatocyte;hepatic stellate cell	20
promoter	chrX	138612874	F9	liver	liver;right lobe of liver;hepatocyte	20
promoter	chrX	138612875	F9	liver	liver;right lobe of liver;hepatocyte	19
promoter	chrX	138612875	F9	liver	liver;right lobe of liver;hepatocyte	19
promoter	chrX	138612876	F9	liver	liver;right lobe of liver;hepatocyte	18
promoter	chr9	35658032	RMRP	liver	liver;hepatocyte;kidney embryonic	16
promoter	chr12	121416354	HNF1A	hepatocyte	hepatocyte;liver;right lobe of liver;kidney;left kidney;right kidney;pancreas;body of pancreas;endocrine pancreas	16
promoter	chr1	160001799	PIGM	granulocyte	granulocyte;skin fibroblast	15
