# Supplementary material for: Remus: A Web Application for Prioritization of Regulatory Regions and Variants in Monogenic Diseases
Source: Front Genet. 2021 Mar 5;12:638960. doi: 10.3389/fgene.2021.638960 (PMC7978111; doi:10.3389/fgene.2021.638960)
Supplement: Supplementary Figure 1 — Illustration of track collapsing and liftover. Original tracks for the same tissue (biological replicates) and in the same genome build were merged. Next, coordinates were lifted over to the other genome build (i.e., hg19 to hg38, and vice versa), and merging on the same tissues was performed again. [file Data_Sheet_1.zip › Supplementary Material/Data Sheet 2.pdf]

Supplementary Table 5

Regulator	Source	Track_type	Median_track_size [bp]	All_tissues_size [bp]	Fraction	Median_track_size_joint	Fraction_joint
chromatin	encode	primary_cell	49478870	1361271278	0.0363	49724351	0.0365
chromatin	encode	tissue	50565329	1361271278	0.0371		
chromatin	screen	primary_cell	55522362	474960506	0.1169	69574398	0.1465
chromatin	screen	tissue	81122328	474960506	0.1708		
enhancers	encode	primary_cell	12290477	320135082	0.0384	19579021	0.0612
enhancers	encode	tissue	37777567	320135082	0.1180		
enhancers	fantom5	primary_cell	547980	9988039	0.0549	491536	0.0492
enhancers	fantom5	tissue	334676	9988039	0.0335		
enhancers	screen	primary_cell	20865305	351104599	0.0594	30620251	0.0872
enhancers	screen	tissue	32681834	351104599	0.0931		
promoters	fantom5	primary_cell	2872249	21047960	0.1365	2931685	0.1393
promoters	fantom5	tissue	3222865	21047960	0.1531		
promoters	screen	primary_cell	32051540	188688724	0.1699	32728042	0.1734
promoters	screen	tissue	34577624	188688724	0.1833		
