# Supplementary material for: Remus: A Web Application for Prioritization of Regulatory Regions and Variants in Monogenic Diseases
Source: Front Genet. 2021 Mar 5;12:638960. doi: 10.3389/fgene.2021.638960 (PMC7978111; doi:10.3389/fgene.2021.638960)
Supplement: Supplementary Figure 1 — Illustration of track collapsing and liftover. Original tracks for the same tissue (biological replicates) and in the same genome build were merged. Next, coordinates were lifted over to the other genome build (i.e., hg19 to hg38, and vice versa), and merging on the same tissues was performed again. [file Data_Sheet_1.zip › Supplementary Material/Data Sheet 3.pdf]

ENCODE Accessible chromatin

https://www.encodeproject.org/metadata/type%3DExperiment%26status%3Dreleased%26replicates.library.biosample.donor.organism.scientific_name%3DHomo%2Bsapiens
%26files.file_type%3Dbed%2BnarrowPeak%26biosample_ontology.classification%3Dprimary%2Bcell%26biosample_ontology.classification%3Dtissue%26audit.ERROR.category
%2521%3Dextremely%2Blow%2Bspot%2Bscore%26audit.ERROR.category%2521%3Dextremely%2Blow%2Bread%2Bdepth%26assay_slims%3DDNA%2Baccessibility
%26replicates.library.biosample.treatments.treatment_term_name%2521%3Dcisplatin%26replicates.library.biosample.treatments.treatment_term_name%2521%3Dretinoic%2Bacid
%26advancedQuery%3D%2540type%253AExperiment%2Bdate_released%253A%255B2009-01-01%2BTO%2B2019-09-30%255D/metadata.tsv

https://www.encodeproject.org/files/ENCFF876ORT/@@download/ENCFF876ORT.bed.gz

https://www.encodeproject.org/files/ENCFF804SPK/@@download/ENCFF804SPK.bed.gz

https://www.encodeproject.org/files/ENCFF279CTV/@@download/ENCFF279CTV.bed.gz

https://www.encodeproject.org/files/ENCFF186BIW/@@download/ENCFF186BIW.bed.gz

https://www.encodeproject.org/files/ENCFF327BRB/@@download/ENCFF327BRB.bed.gz

https://www.encodeproject.org/files/ENCFF379ZXE/@@download/ENCFF379ZXE.bed.gz

https://www.encodeproject.org/files/ENCFF326LBO/@@download/ENCFF326LBO.bed.gz

https://www.encodeproject.org/files/ENCFF405FGA/@@download/ENCFF405FGA.bed.gz

https://www.encodeproject.org/files/ENCFF936LFC/@@download/ENCFF936LFC.bed.gz

https://www.encodeproject.org/files/ENCFF481BII/@@download/ENCFF481BII.bed.gz

https://www.encodeproject.org/files/ENCFF472DJP/@@download/ENCFF472DJP.bed.gz

https://www.encodeproject.org/files/ENCFF196VQQ/@@download/ENCFF196VQQ.bed.gz

https://www.encodeproject.org/files/ENCFF047SSX/@@download/ENCFF047SSX.bed.gz

https://www.encodeproject.org/files/ENCFF250JHL/@@download/ENCFF250JHL.bed.gz

https://www.encodeproject.org/files/ENCFF683PCM/@@download/ENCFF683PCM.bed.gz

https://www.encodeproject.org/files/ENCFF292QIX/@@download/ENCFF292QIX.bed.gz

https://www.encodeproject.org/files/ENCFF367ZGO/@@download/ENCFF367ZGO.bed.gz

https://www.encodeproject.org/files/ENCFF805JKJ/@@download/ENCFF805JKJ.bed.gz

https://www.encodeproject.org/files/ENCFF024LKC/@@download/ENCFF024LKC.bed.gz

https://www.encodeproject.org/files/ENCFF872DCV/@@download/ENCFF872DCV.bed.gz

https://www.encodeproject.org/files/ENCFF402DJK/@@download/ENCFF402DJK.bed.gz

https://www.encodeproject.org/files/ENCFF318YXS/@@download/ENCFF318YXS.bed.gz

https://www.encodeproject.org/files/ENCFF138YRO/@@download/ENCFF138YRO.bed.gz

https://www.encodeproject.org/files/ENCFF133ILP/@@download/ENCFF133ILP.bed.gz

https://www.encodeproject.org/files/ENCFF668TER/@@download/ENCFF668TER.bed.gz

https://www.encodeproject.org/files/ENCFF304TBE/@@download/ENCFF304TBE.bed.gz

https://www.encodeproject.org/files/ENCFF839YTP/@@download/ENCFF839YTP.bed.gz

https://www.encodeproject.org/files/ENCFF991UKF/@@download/ENCFF991UKF.bed.gz

https://www.encodeproject.org/files/ENCFF808EVW/@@download/ENCFF808EVW.bed.gz

https://www.encodeproject.org/files/ENCFF710ARV/@@download/ENCFF710ARV.bed.gz

https://www.encodeproject.org/files/ENCFF399QLP/@@download/ENCFF399QLP.bed.gz

https://www.encodeproject.org/files/ENCFF906IMQ/@@download/ENCFF906IMQ.bed.gz

https://www.encodeproject.org/files/ENCFF259KDP/@@download/ENCFF259KDP.bed.gz

https://www.encodeproject.org/files/ENCFF814UZW/@@download/ENCFF814UZW.bed.gz

https://www.encodeproject.org/files/ENCFF568EZF/@@download/ENCFF568EZF.bed.gz

https://www.encodeproject.org/files/ENCFF332ZJY/@@download/ENCFF332ZJY.bed.gz

https://www.encodeproject.org/files/ENCFF224KRB/@@download/ENCFF224KRB.bed.gz

https://www.encodeproject.org/files/ENCFF440NVK/@@download/ENCFF440NVK.bed.gz

https://www.encodeproject.org/files/ENCFF299AHY/@@download/ENCFF299AHY.bed.gz

https://www.encodeproject.org/files/ENCFF241MIW/@@download/ENCFF241MIW.bed.gz

https://www.encodeproject.org/files/ENCFF127HJI/@@download/ENCFF127HJI.bed.gz

https://www.encodeproject.org/files/ENCFF380DUQ/@@download/ENCFF380DUQ.bed.gz

https://www.encodeproject.org/files/ENCFF507JIF/@@download/ENCFF507JIF.bed.gz

https://www.encodeproject.org/files/ENCFF159HJV/@@download/ENCFF159HJV.bed.gz

https://www.encodeproject.org/files/ENCFF444ZRC/@@download/ENCFF444ZRC.bed.gz

https://www.encodeproject.org/files/ENCFF210RAG/@@download/ENCFF210RAG.bed.gz

https://www.encodeproject.org/files/ENCFF805PHJ/@@download/ENCFF805PHJ.bed.gz

https://www.encodeproject.org/files/ENCFF083NJI/@@download/ENCFF083NJI.bed.gz

https://www.encodeproject.org/files/ENCFF403YHM/@@download/ENCFF403YHM.bed.gz
https://www.encodeproject.org/files/ENCFF148PHO/@@download/ENCFF148PHO.bed.gz
https://www.encodeproject.org/files/ENCFF115HTH/@@download/ENCFF115HTH.bed.gz
https://www.encodeproject.org/files/ENCFF625XNX/@@download/ENCFF625XNX.bed.gz
https://www.encodeproject.org/files/ENCFF326SOJ/@@download/ENCFF326SOJ.bed.gz
https://www.encodeproject.org/files/ENCFF536SRV/@@download/ENCFF536SRV.bed.gz
https://www.encodeproject.org/files/ENCFF354SWE/@@download/ENCFF354SWE.bed.gz
https://www.encodeproject.org/files/ENCFF470JVG/@@download/ENCFF470JVG.bed.gz
https://www.encodeproject.org/files/ENCFF675ZGP/@@download/ENCFF675ZGP.bed.gz
https://www.encodeproject.org/files/ENCFF378WSR/@@download/ENCFF378WSR.bed.gz
https://www.encodeproject.org/files/ENCFF341RBP/@@download/ENCFF341RBP.bed.gz
https://www.encodeproject.org/files/ENCFF948PWG/@@download/ENCFF948PWG.bed.gz
https://www.encodeproject.org/files/ENCFF310YLD/@@download/ENCFF310YLD.bed.gz
https://www.encodeproject.org/files/ENCFF468ODG/@@download/ENCFF468ODG.bed.gz
https://www.encodeproject.org/files/ENCFF773JYF/@@download/ENCFF773JYF.bed.gz
https://www.encodeproject.org/files/ENCFF703UHS/@@download/ENCFF703UHS.bed.gz
https://www.encodeproject.org/files/ENCFF458IQB/@@download/ENCFF458IQB.bed.gz
https://www.encodeproject.org/files/ENCFF495XDQ/@@download/ENCFF495XDQ.bed.gz
https://www.encodeproject.org/files/ENCFF001UUI/@@download/ENCFF001UUI.bed.gz
https://www.encodeproject.org/files/ENCFF732MQW/@@download/ENCFF732MQW.bed.gz
https://www.encodeproject.org/files/ENCFF966DRW/@@download/ENCFF966DRW.bed.gz
https://www.encodeproject.org/files/ENCFF952ETG/@@download/ENCFF952ETG.bed.gz
https://www.encodeproject.org/files/ENCFF455ZGC/@@download/ENCFF455ZGC.bed.gz
https://www.encodeproject.org/files/ENCFF447UOZ/@@download/ENCFF447UOZ.bed.gz
https://www.encodeproject.org/files/ENCFF292CDE/@@download/ENCFF292CDE.bed.gz
https://www.encodeproject.org/files/ENCFF115WLO/@@download/ENCFF115WLO.bed.gz
https://www.encodeproject.org/files/ENCFF665JVG/@@download/ENCFF665JVG.bed.gz
https://www.encodeproject.org/files/ENCFF843EEX/@@download/ENCFF843EEX.bed.gz
https://www.encodeproject.org/files/ENCFF336MQK/@@download/ENCFF336MQK.bed.gz
https://www.encodeproject.org/files/ENCFF968IOH/@@download/ENCFF968IOH.bed.gz
https://www.encodeproject.org/files/ENCFF159MJS/@@download/ENCFF159MJS.bed.gz
https://www.encodeproject.org/files/ENCFF958ENE/@@download/ENCFF958ENE.bed.gz
https://www.encodeproject.org/files/ENCFF733SKD/@@download/ENCFF733SKD.bed.gz
https://www.encodeproject.org/files/ENCFF001UXO/@@download/ENCFF001UXO.bed.gz
https://www.encodeproject.org/files/ENCFF606KOP/@@download/ENCFF606KOP.bed.gz
https://www.encodeproject.org/files/ENCFF217FCM/@@download/ENCFF217FCM.bed.gz
https://www.encodeproject.org/files/ENCFF654CVF/@@download/ENCFF654CVF.bed.gz
https://www.encodeproject.org/files/ENCFF445UFF/@@download/ENCFF445UFF.bed.gz
https://www.encodeproject.org/files/ENCFF243TMO/@@download/ENCFF243TMO.bed.gz
https://www.encodeproject.org/files/ENCFF019TMK/@@download/ENCFF019TMK.bed.gz
https://www.encodeproject.org/files/ENCFF292MEN/@@download/ENCFF292MEN.bed.gz
https://www.encodeproject.org/files/ENCFF700RJO/@@download/ENCFF700RJO.bed.gz
https://www.encodeproject.org/files/ENCFF897BWP/@@download/ENCFF897BWP.bed.gz
https://www.encodeproject.org/files/ENCFF840ZTK/@@download/ENCFF840ZTK.bed.gz
https://www.encodeproject.org/files/ENCFF224LJW/@@download/ENCFF224LJW.bed.gz
https://www.encodeproject.org/files/ENCFF675RVL/@@download/ENCFF675RVL.bed.gz
https://www.encodeproject.org/files/ENCFF727MYL/@@download/ENCFF727MYL.bed.gz
https://www.encodeproject.org/files/ENCFF177AKO/@@download/ENCFF177AKO.bed.gz
https://www.encodeproject.org/files/ENCFF523ZNN/@@download/ENCFF523ZNN.bed.gz
https://www.encodeproject.org/files/ENCFF566SXQ/@@download/ENCFF566SXQ.bed.gz
https://www.encodeproject.org/files/ENCFF614QQR/@@download/ENCFF614QQR.bed.gz
https://www.encodeproject.org/files/ENCFF071RMN/@@download/ENCFF071RMN.bed.gz
https://www.encodeproject.org/files/ENCFF922MQL/@@download/ENCFF922MQL.bed.gz

https://www.encodeproject.org/files/ENCFF468VWC/@@download/ENCFF468VWC.bed.gz
https://www.encodeproject.org/files/ENCFF907GJF/@@download/ENCFF907GJF.bed.gz
https://www.encodeproject.org/files/ENCFF168YQF/@@download/ENCFF168YQF.bed.gz
https://www.encodeproject.org/files/ENCFF923EVD/@@download/ENCFF923EVD.bed.gz
https://www.encodeproject.org/files/ENCFF144HBQ/@@download/ENCFF144HBQ.bed.gz
https://www.encodeproject.org/files/ENCFF757GAE/@@download/ENCFF757GAE.bed.gz
https://www.encodeproject.org/files/ENCFF337UCF/@@download/ENCFF337UCF.bed.gz
https://www.encodeproject.org/files/ENCFF207HFD/@@download/ENCFF207HFD.bed.gz
https://www.encodeproject.org/files/ENCFF469AJE/@@download/ENCFF469AJE.bed.gz
https://www.encodeproject.org/files/ENCFF706OKJ/@@download/ENCFF706OKJ.bed.gz
https://www.encodeproject.org/files/ENCFF894QXT/@@download/ENCFF894QXT.bed.gz
https://www.encodeproject.org/files/ENCFF217JVU/@@download/ENCFF217JVU.bed.gz
https://www.encodeproject.org/files/ENCFF265RXK/@@download/ENCFF265RXK.bed.gz
https://www.encodeproject.org/files/ENCFF860NPB/@@download/ENCFF860NPB.bed.gz
https://www.encodeproject.org/files/ENCFF815GDP/@@download/ENCFF815GDP.bed.gz
https://www.encodeproject.org/files/ENCFF247IVB/@@download/ENCFF247IVB.bed.gz
https://www.encodeproject.org/files/ENCFF007TSW/@@download/ENCFF007TSW.bed.gz
https://www.encodeproject.org/files/ENCFF335JED/@@download/ENCFF335JED.bed.gz
https://www.encodeproject.org/files/ENCFF198RKA/@@download/ENCFF198RKA.bed.gz
https://www.encodeproject.org/files/ENCFF001UZF/@@download/ENCFF001UZF.bed.gz
https://www.encodeproject.org/files/ENCFF277ILM/@@download/ENCFF277ILM.bed.gz
https://www.encodeproject.org/files/ENCFF416JUZ/@@download/ENCFF416JUZ.bed.gz
https://www.encodeproject.org/files/ENCFF974GLD/@@download/ENCFF974GLD.bed.gz
https://www.encodeproject.org/files/ENCFF501IFI/@@download/ENCFF501IFI.bed.gz
https://www.encodeproject.org/files/ENCFF648KXM/@@download/ENCFF648KXM.bed.gz
https://www.encodeproject.org/files/ENCFF857RHW/@@download/ENCFF857RHW.bed.gz
https://www.encodeproject.org/files/ENCFF001UZE/@@download/ENCFF001UZE.bed.gz
https://www.encodeproject.org/files/ENCFF001UZD/@@download/ENCFF001UZD.bed.gz
https://www.encodeproject.org/files/ENCFF874PJV/@@download/ENCFF874PJV.bed.gz
https://www.encodeproject.org/files/ENCFF856BCY/@@download/ENCFF856BCY.bed.gz
https://www.encodeproject.org/files/ENCFF037XOG/@@download/ENCFF037XOG.bed.gz
https://www.encodeproject.org/files/ENCFF787OCX/@@download/ENCFF787OCX.bed.gz
https://www.encodeproject.org/files/ENCFF725UKA/@@download/ENCFF725UKA.bed.gz
https://www.encodeproject.org/files/ENCFF148NZN/@@download/ENCFF148NZN.bed.gz
https://www.encodeproject.org/files/ENCFF576YYS/@@download/ENCFF576YYS.bed.gz
https://www.encodeproject.org/files/ENCFF471KBD/@@download/ENCFF471KBD.bed.gz
https://www.encodeproject.org/files/ENCFF301HUB/@@download/ENCFF301HUB.bed.gz
https://www.encodeproject.org/files/ENCFF828VUZ/@@download/ENCFF828VUZ.bed.gz
https://www.encodeproject.org/files/ENCFF679MSO/@@download/ENCFF679MSO.bed.gz
https://www.encodeproject.org/files/ENCFF265ZNN/@@download/ENCFF265ZNN.bed.gz
https://www.encodeproject.org/files/ENCFF254VDZ/@@download/ENCFF254VDZ.bed.gz
https://www.encodeproject.org/files/ENCFF541WEX/@@download/ENCFF541WEX.bed.gz
https://www.encodeproject.org/files/ENCFF603LUK/@@download/ENCFF603LUK.bed.gz
https://www.encodeproject.org/files/ENCFF148HZW/@@download/ENCFF148HZW.bed.gz
https://www.encodeproject.org/files/ENCFF591NRB/@@download/ENCFF591NRB.bed.gz
https://www.encodeproject.org/files/ENCFF401BCF/@@download/ENCFF401BCF.bed.gz
https://www.encodeproject.org/files/ENCFF911LSM/@@download/ENCFF911LSM.bed.gz
https://www.encodeproject.org/files/ENCFF380LAI/@@download/ENCFF380LAI.bed.gz
https://www.encodeproject.org/files/ENCFF015ULH/@@download/ENCFF015ULH.bed.gz
https://www.encodeproject.org/files/ENCFF402DMO/@@download/ENCFF402DMO.bed.gz
https://www.encodeproject.org/files/ENCFF569TTJ/@@download/ENCFF569TTJ.bed.gz
https://www.encodeproject.org/files/ENCFF309NRH/@@download/ENCFF309NRH.bed.gz
https://www.encodeproject.org/files/ENCFF145LFA/@@download/ENCFF145LFA.bed.gz

https://www.encodeproject.org/files/ENCFF201DHP/@@download/ENCFF201DHP.bed.gz
https://www.encodeproject.org/files/ENCFF287TFK/@@download/ENCFF287TFK.bed.gz
https://www.encodeproject.org/files/ENCFF474QAJ/@@download/ENCFF474QAJ.bed.gz
https://www.encodeproject.org/files/ENCFF969YOA/@@download/ENCFF969YOA.bed.gz
https://www.encodeproject.org/files/ENCFF982VBQ/@@download/ENCFF982VBQ.bed.gz
https://www.encodeproject.org/files/ENCFF974WUR/@@download/ENCFF974WUR.bed.gz
https://www.encodeproject.org/files/ENCFF443SFQ/@@download/ENCFF443SFQ.bed.gz
https://www.encodeproject.org/files/ENCFF099SSL/@@download/ENCFF099SSL.bed.gz
https://www.encodeproject.org/files/ENCFF024DAR/@@download/ENCFF024DAR.bed.gz
https://www.encodeproject.org/files/ENCFF600EJV/@@download/ENCFF600EJV.bed.gz
https://www.encodeproject.org/files/ENCFF521ZLX/@@download/ENCFF521ZLX.bed.gz
https://www.encodeproject.org/files/ENCFF058SNC/@@download/ENCFF058SNC.bed.gz
https://www.encodeproject.org/files/ENCFF363VBT/@@download/ENCFF363VBT.bed.gz
https://www.encodeproject.org/files/ENCFF395XDV/@@download/ENCFF395XDV.bed.gz
https://www.encodeproject.org/files/ENCFF109VIJ/@@download/ENCFF109VIJ.bed.gz
https://www.encodeproject.org/files/ENCFF062FOP/@@download/ENCFF062FOP.bed.gz
https://www.encodeproject.org/files/ENCFF422HNI/@@download/ENCFF422HNI.bed.gz
https://www.encodeproject.org/files/ENCFF519DSD/@@download/ENCFF519DSD.bed.gz
https://www.encodeproject.org/files/ENCFF487ZAH/@@download/ENCFF487ZAH.bed.gz
https://www.encodeproject.org/files/ENCFF612FUV/@@download/ENCFF612FUV.bed.gz
https://www.encodeproject.org/files/ENCFF450BGX/@@download/ENCFF450BGX.bed.gz
https://www.encodeproject.org/files/ENCFF387SIU/@@download/ENCFF387SIU.bed.gz
https://www.encodeproject.org/files/ENCFF378KGO/@@download/ENCFF378KGO.bed.gz
https://www.encodeproject.org/files/ENCFF285IVA/@@download/ENCFF285IVA.bed.gz
https://www.encodeproject.org/files/ENCFF061JQU/@@download/ENCFF061JQU.bed.gz
https://www.encodeproject.org/files/ENCFF001SPJ/@@download/ENCFF001SPJ.bed.gz
https://www.encodeproject.org/files/ENCFF001UXJ/@@download/ENCFF001UXJ.bed.gz
https://www.encodeproject.org/files/ENCFF012SEI/@@download/ENCFF012SEI.bed.gz
https://www.encodeproject.org/files/ENCFF573CUG/@@download/ENCFF573CUG.bed.gz
https://www.encodeproject.org/files/ENCFF510LHV/@@download/ENCFF510LHV.bed.gz
https://www.encodeproject.org/files/ENCFF071KIB/@@download/ENCFF071KIB.bed.gz
https://www.encodeproject.org/files/ENCFF993MCD/@@download/ENCFF993MCD.bed.gz
https://www.encodeproject.org/files/ENCFF837VPM/@@download/ENCFF837VPM.bed.gz
https://www.encodeproject.org/files/ENCFF399OBI/@@download/ENCFF399OBI.bed.gz
https://www.encodeproject.org/files/ENCFF601NRR/@@download/ENCFF601NRR.bed.gz
https://www.encodeproject.org/files/ENCFF300IHF/@@download/ENCFF300IHF.bed.gz
https://www.encodeproject.org/files/ENCFF581FDW/@@download/ENCFF581FDW.bed.gz
https://www.encodeproject.org/files/ENCFF542CPB/@@download/ENCFF542CPB.bed.gz
https://www.encodeproject.org/files/ENCFF053LUK/@@download/ENCFF053LUK.bed.gz
https://www.encodeproject.org/files/ENCFF333XKC/@@download/ENCFF333XKC.bed.gz
https://www.encodeproject.org/files/ENCFF171LSI/@@download/ENCFF171LSI.bed.gz
https://www.encodeproject.org/files/ENCFF918ICP/@@download/ENCFF918ICP.bed.gz
https://www.encodeproject.org/files/ENCFF559VIU/@@download/ENCFF559VIU.bed.gz
https://www.encodeproject.org/files/ENCFF182JTX/@@download/ENCFF182JTX.bed.gz
https://www.encodeproject.org/files/ENCFF971VBF/@@download/ENCFF971VBF.bed.gz
https://www.encodeproject.org/files/ENCFF408CBX/@@download/ENCFF408CBX.bed.gz
https://www.encodeproject.org/files/ENCFF491YQL/@@download/ENCFF491YQL.bed.gz
https://www.encodeproject.org/files/ENCFF418BQS/@@download/ENCFF418BQS.bed.gz
https://www.encodeproject.org/files/ENCFF311ACF/@@download/ENCFF311ACF.bed.gz
https://www.encodeproject.org/files/ENCFF348DBB/@@download/ENCFF348DBB.bed.gz
https://www.encodeproject.org/files/ENCFF662OET/@@download/ENCFF662OET.bed.gz
https://www.encodeproject.org/files/ENCFF007BRU/@@download/ENCFF007BRU.bed.gz
https://www.encodeproject.org/files/ENCFF779HDH/@@download/ENCFF779HDH.bed.gz

https://www.encodeproject.org/files/ENCFF335ZUW/@@download/ENCFF335ZUW.bed.gz
https://www.encodeproject.org/files/ENCFF594OFO/@@download/ENCFF594OFO.bed.gz
https://www.encodeproject.org/files/ENCFF419STH/@@download/ENCFF419STH.bed.gz
https://www.encodeproject.org/files/ENCFF786BNQ/@@download/ENCFF786BNQ.bed.gz
https://www.encodeproject.org/files/ENCFF799TYV/@@download/ENCFF799TYV.bed.gz
https://www.encodeproject.org/files/ENCFF862JLT/@@download/ENCFF862JLT.bed.gz
https://www.encodeproject.org/files/ENCFF333MTL/@@download/ENCFF333MTL.bed.gz
https://www.encodeproject.org/files/ENCFF751OEX/@@download/ENCFF751OEX.bed.gz
https://www.encodeproject.org/files/ENCFF172HGE/@@download/ENCFF172HGE.bed.gz
https://www.encodeproject.org/files/ENCFF640MSE/@@download/ENCFF640MSE.bed.gz
https://www.encodeproject.org/files/ENCFF695LNO/@@download/ENCFF695LNO.bed.gz
https://www.encodeproject.org/files/ENCFF289LRM/@@download/ENCFF289LRM.bed.gz
https://www.encodeproject.org/files/ENCFF898GQW/@@download/ENCFF898GQW.bed.gz
https://www.encodeproject.org/files/ENCFF663DTX/@@download/ENCFF663DTX.bed.gz
https://www.encodeproject.org/files/ENCFF118AXD/@@download/ENCFF118AXD.bed.gz
https://www.encodeproject.org/files/ENCFF270OXY/@@download/ENCFF270OXY.bed.gz
https://www.encodeproject.org/files/ENCFF077NBT/@@download/ENCFF077NBT.bed.gz
https://www.encodeproject.org/files/ENCFF601ZWO/@@download/ENCFF601ZWO.bed.gz
https://www.encodeproject.org/files/ENCFF017TAW/@@download/ENCFF017TAW.bed.gz
https://www.encodeproject.org/files/ENCFF622UTM/@@download/ENCFF622UTM.bed.gz
https://www.encodeproject.org/files/ENCFF236UMJ/@@download/ENCFF236UMJ.bed.gz
https://www.encodeproject.org/files/ENCFF431UVI/@@download/ENCFF431UVI.bed.gz
https://www.encodeproject.org/files/ENCFF544CQR/@@download/ENCFF544CQR.bed.gz
https://www.encodeproject.org/files/ENCFF484XHW/@@download/ENCFF484XHW.bed.gz
https://www.encodeproject.org/files/ENCFF330CNE/@@download/ENCFF330CNE.bed.gz
https://www.encodeproject.org/files/ENCFF821OUM/@@download/ENCFF821OUM.bed.gz
https://www.encodeproject.org/files/ENCFF848SAN/@@download/ENCFF848SAN.bed.gz
https://www.encodeproject.org/files/ENCFF561ENC/@@download/ENCFF561ENC.bed.gz
https://www.encodeproject.org/files/ENCFF275FNU/@@download/ENCFF275FNU.bed.gz
https://www.encodeproject.org/files/ENCFF042LWW/@@download/ENCFF042LWW.bed.gz
https://www.encodeproject.org/files/ENCFF679HWR/@@download/ENCFF679HWR.bed.gz
https://www.encodeproject.org/files/ENCFF137UKU/@@download/ENCFF137UKU.bed.gz
https://www.encodeproject.org/files/ENCFF537THG/@@download/ENCFF537THG.bed.gz
https://www.encodeproject.org/files/ENCFF284JYN/@@download/ENCFF284JYN.bed.gz
https://www.encodeproject.org/files/ENCFF877KLX/@@download/ENCFF877KLX.bed.gz
https://www.encodeproject.org/files/ENCFF227HYU/@@download/ENCFF227HYU.bed.gz
https://www.encodeproject.org/files/ENCFF360CCE/@@download/ENCFF360CCE.bed.gz
https://www.encodeproject.org/files/ENCFF312ZGX/@@download/ENCFF312ZGX.bed.gz
https://www.encodeproject.org/files/ENCFF069HXE/@@download/ENCFF069HXE.bed.gz
https://www.encodeproject.org/files/ENCFF783SLY/@@download/ENCFF783SLY.bed.gz
https://www.encodeproject.org/files/ENCFF369YZL/@@download/ENCFF369YZL.bed.gz
https://www.encodeproject.org/files/ENCFF214NKM/@@download/ENCFF214NKM.bed.gz
https://www.encodeproject.org/files/ENCFF880QTT/@@download/ENCFF880QTT.bed.gz
https://www.encodeproject.org/files/ENCFF683CID/@@download/ENCFF683CID.bed.gz
https://www.encodeproject.org/files/ENCFF901III/@@download/ENCFF901III.bed.gz
https://www.encodeproject.org/files/ENCFF589FTC/@@download/ENCFF589FTC.bed.gz
https://www.encodeproject.org/files/ENCFF433RET/@@download/ENCFF433RET.bed.gz
https://www.encodeproject.org/files/ENCFF717QSC/@@download/ENCFF717QSC.bed.gz
https://www.encodeproject.org/files/ENCFF286LLG/@@download/ENCFF286LLG.bed.gz
https://www.encodeproject.org/files/ENCFF337KZM/@@download/ENCFF337KZM.bed.gz
https://www.encodeproject.org/files/ENCFF073OXD/@@download/ENCFF073OXD.bed.gz
https://www.encodeproject.org/files/ENCFF125FSL/@@download/ENCFF125FSL.bed.gz
https://www.encodeproject.org/files/ENCFF662ZSK/@@download/ENCFF662ZSK.bed.gz

https://www.encodeproject.org/files/ENCFF016PCJ/@@download/ENCFF016PCJ.bed.gz
https://www.encodeproject.org/files/ENCFF284QST/@@download/ENCFF284QST.bed.gz
https://www.encodeproject.org/files/ENCFF908SUH/@@download/ENCFF908SUH.bed.gz
https://www.encodeproject.org/files/ENCFF243YZS/@@download/ENCFF243YZS.bed.gz
https://www.encodeproject.org/files/ENCFF504DAE/@@download/ENCFF504DAE.bed.gz
https://www.encodeproject.org/files/ENCFF706NZB/@@download/ENCFF706NZB.bed.gz
https://www.encodeproject.org/files/ENCFF246POE/@@download/ENCFF246POE.bed.gz
https://www.encodeproject.org/files/ENCFF468OWC/@@download/ENCFF468OWC.bed.gz
https://www.encodeproject.org/files/ENCFF614FJM/@@download/ENCFF614FJM.bed.gz
https://www.encodeproject.org/files/ENCFF529IQV/@@download/ENCFF529IQV.bed.gz
https://www.encodeproject.org/files/ENCFF907KYP/@@download/ENCFF907KYP.bed.gz
https://www.encodeproject.org/files/ENCFF904UQR/@@download/ENCFF904UQR.bed.gz
https://www.encodeproject.org/files/ENCFF047CHH/@@download/ENCFF047CHH.bed.gz
https://www.encodeproject.org/files/ENCFF254ICV/@@download/ENCFF254ICV.bed.gz
https://www.encodeproject.org/files/ENCFF144ERX/@@download/ENCFF144ERX.bed.gz
https://www.encodeproject.org/files/ENCFF003GKO/@@download/ENCFF003GKO.bed.gz
https://www.encodeproject.org/files/ENCFF700QDA/@@download/ENCFF700QDA.bed.gz
https://www.encodeproject.org/files/ENCFF368AAH/@@download/ENCFF368AAH.bed.gz
https://www.encodeproject.org/files/ENCFF815XOR/@@download/ENCFF815XOR.bed.gz
https://www.encodeproject.org/files/ENCFF652KUN/@@download/ENCFF652KUN.bed.gz
https://www.encodeproject.org/files/ENCFF242ABW/@@download/ENCFF242ABW.bed.gz
https://www.encodeproject.org/files/ENCFF846UED/@@download/ENCFF846UED.bed.gz
https://www.encodeproject.org/files/ENCFF255HLT/@@download/ENCFF255HLT.bed.gz
https://www.encodeproject.org/files/ENCFF573KCL/@@download/ENCFF573KCL.bed.gz
https://www.encodeproject.org/files/ENCFF137EPX/@@download/ENCFF137EPX.bed.gz
https://www.encodeproject.org/files/ENCFF119HGJ/@@download/ENCFF119HGJ.bed.gz
https://www.encodeproject.org/files/ENCFF433PDK/@@download/ENCFF433PDK.bed.gz
https://www.encodeproject.org/files/ENCFF593JZH/@@download/ENCFF593JZH.bed.gz
https://www.encodeproject.org/files/ENCFF470LXJ/@@download/ENCFF470LXJ.bed.gz
https://www.encodeproject.org/files/ENCFF955SRI/@@download/ENCFF955SRI.bed.gz
https://www.encodeproject.org/files/ENCFF925OKW/@@download/ENCFF925OKW.bed.gz
https://www.encodeproject.org/files/ENCFF066XNH/@@download/ENCFF066XNH.bed.gz
https://www.encodeproject.org/files/ENCFF725QIG/@@download/ENCFF725QIG.bed.gz
https://www.encodeproject.org/files/ENCFF743UIM/@@download/ENCFF743UIM.bed.gz
https://www.encodeproject.org/files/ENCFF770BVB/@@download/ENCFF770BVB.bed.gz
https://www.encodeproject.org/files/ENCFF927MCZ/@@download/ENCFF927MCZ.bed.gz
https://www.encodeproject.org/files/ENCFF900JRB/@@download/ENCFF900JRB.bed.gz
https://www.encodeproject.org/files/ENCFF740XLE/@@download/ENCFF740XLE.bed.gz
https://www.encodeproject.org/files/ENCFF212EPW/@@download/ENCFF212EPW.bed.gz
https://www.encodeproject.org/files/ENCFF434VXT/@@download/ENCFF434VXT.bed.gz
https://www.encodeproject.org/files/ENCFF479XZN/@@download/ENCFF479XZN.bed.gz
https://www.encodeproject.org/files/ENCFF333PLF/@@download/ENCFF333PLF.bed.gz
https://www.encodeproject.org/files/ENCFF729ATE/@@download/ENCFF729ATE.bed.gz
https://www.encodeproject.org/files/ENCFF227WDB/@@download/ENCFF227WDB.bed.gz
https://www.encodeproject.org/files/ENCFF278ROU/@@download/ENCFF278ROU.bed.gz
https://www.encodeproject.org/files/ENCFF447UPF/@@download/ENCFF447UPF.bed.gz
https://www.encodeproject.org/files/ENCFF187NZJ/@@download/ENCFF187NZJ.bed.gz
https://www.encodeproject.org/files/ENCFF682OQR/@@download/ENCFF682OQR.bed.gz
https://www.encodeproject.org/files/ENCFF362WET/@@download/ENCFF362WET.bed.gz
https://www.encodeproject.org/files/ENCFF849IJR/@@download/ENCFF849IJR.bed.gz
https://www.encodeproject.org/files/ENCFF217MXO/@@download/ENCFF217MXO.bed.gz
https://www.encodeproject.org/files/ENCFF319ZSH/@@download/ENCFF319ZSH.bed.gz
https://www.encodeproject.org/files/ENCFF008XKZ/@@download/ENCFF008XKZ.bed.gz

https://www.encodeproject.org/files/ENCFF526VCU/@@download/ENCFF526VCU.bed.gz
https://www.encodeproject.org/files/ENCFF030FXE/@@download/ENCFF030FXE.bed.gz
https://www.encodeproject.org/files/ENCFF817MBU/@@download/ENCFF817MBU.bed.gz
https://www.encodeproject.org/files/ENCFF886DDL/@@download/ENCFF886DDL.bed.gz
https://www.encodeproject.org/files/ENCFF837KHA/@@download/ENCFF837KHA.bed.gz
https://www.encodeproject.org/files/ENCFF762SWA/@@download/ENCFF762SWA.bed.gz
https://www.encodeproject.org/files/ENCFF852IEJ/@@download/ENCFF852IEJ.bed.gz
https://www.encodeproject.org/files/ENCFF282BUD/@@download/ENCFF282BUD.bed.gz
https://www.encodeproject.org/files/ENCFF512LDK/@@download/ENCFF512LDK.bed.gz
https://www.encodeproject.org/files/ENCFF011UBS/@@download/ENCFF011UBS.bed.gz
https://www.encodeproject.org/files/ENCFF674ZTX/@@download/ENCFF674ZTX.bed.gz
https://www.encodeproject.org/files/ENCFF726MBT/@@download/ENCFF726MBT.bed.gz
https://www.encodeproject.org/files/ENCFF809SXS/@@download/ENCFF809SXS.bed.gz
https://www.encodeproject.org/files/ENCFF091BAW/@@download/ENCFF091BAW.bed.gz
https://www.encodeproject.org/files/ENCFF867PRM/@@download/ENCFF867PRM.bed.gz
https://www.encodeproject.org/files/ENCFF796WDQ/@@download/ENCFF796WDQ.bed.gz
https://www.encodeproject.org/files/ENCFF428OZI/@@download/ENCFF428OZI.bed.gz
https://www.encodeproject.org/files/ENCFF171NVI/@@download/ENCFF171NVI.bed.gz
https://www.encodeproject.org/files/ENCFF852EZT/@@download/ENCFF852EZT.bed.gz
https://www.encodeproject.org/files/ENCFF070VRK/@@download/ENCFF070VRK.bed.gz
https://www.encodeproject.org/files/ENCFF982BYK/@@download/ENCFF982BYK.bed.gz
https://www.encodeproject.org/files/ENCFF295VYO/@@download/ENCFF295VYO.bed.gz
https://www.encodeproject.org/files/ENCFF959FMP/@@download/ENCFF959FMP.bed.gz
https://www.encodeproject.org/files/ENCFF757CMC/@@download/ENCFF757CMC.bed.gz
https://www.encodeproject.org/files/ENCFF409POH/@@download/ENCFF409POH.bed.gz
https://www.encodeproject.org/files/ENCFF248TFS/@@download/ENCFF248TFS.bed.gz
https://www.encodeproject.org/files/ENCFF834IMZ/@@download/ENCFF834IMZ.bed.gz
https://www.encodeproject.org/files/ENCFF870ASQ/@@download/ENCFF870ASQ.bed.gz
https://www.encodeproject.org/files/ENCFF216UBC/@@download/ENCFF216UBC.bed.gz
https://www.encodeproject.org/files/ENCFF522CKK/@@download/ENCFF522CKK.bed.gz
https://www.encodeproject.org/files/ENCFF414RJJ/@@download/ENCFF414RJJ.bed.gz
https://www.encodeproject.org/files/ENCFF885OTL/@@download/ENCFF885OTL.bed.gz
https://www.encodeproject.org/files/ENCFF790MLW/@@download/ENCFF790MLW.bed.gz
https://www.encodeproject.org/files/ENCFF885IBS/@@download/ENCFF885IBS.bed.gz
https://www.encodeproject.org/files/ENCFF063OCF/@@download/ENCFF063OCF.bed.gz
https://www.encodeproject.org/files/ENCFF716KMR/@@download/ENCFF716KMR.bed.gz
https://www.encodeproject.org/files/ENCFF866PBJ/@@download/ENCFF866PBJ.bed.gz
https://www.encodeproject.org/files/ENCFF756JNG/@@download/ENCFF756JNG.bed.gz
https://www.encodeproject.org/files/ENCFF114NLK/@@download/ENCFF114NLK.bed.gz
https://www.encodeproject.org/files/ENCFF711TUV/@@download/ENCFF711TUV.bed.gz
https://www.encodeproject.org/files/ENCFF428WYR/@@download/ENCFF428WYR.bed.gz
https://www.encodeproject.org/files/ENCFF001SRI/@@download/ENCFF001SRI.bed.gz
https://www.encodeproject.org/files/ENCFF001WAO/@@download/ENCFF001WAO.bed.gz
https://www.encodeproject.org/files/ENCFF001WLA/@@download/ENCFF001WLA.bed.gz
https://www.encodeproject.org/files/ENCFF001WKZ/@@download/ENCFF001WKZ.bed.gz
https://www.encodeproject.org/files/ENCFF762YND/@@download/ENCFF762YND.bed.gz
https://www.encodeproject.org/files/ENCFF811FPE/@@download/ENCFF811FPE.bed.gz
https://www.encodeproject.org/files/ENCFF446FTN/@@download/ENCFF446FTN.bed.gz
https://www.encodeproject.org/files/ENCFF957JQC/@@download/ENCFF957JQC.bed.gz
https://www.encodeproject.org/files/ENCFF173DAV/@@download/ENCFF173DAV.bed.gz
https://www.encodeproject.org/files/ENCFF732RPT/@@download/ENCFF732RPT.bed.gz
https://www.encodeproject.org/files/ENCFF120FAB/@@download/ENCFF120FAB.bed.gz
https://www.encodeproject.org/files/ENCFF366BWN/@@download/ENCFF366BWN.bed.gz

Supplementary Table 1

https://www.encodeproject.org/files/ENCFF015SFI/@@download/ENCFF015SFI.bed.gz
https://www.encodeproject.org/files/ENCFF838RWH/@@download/ENCFF838RWH.bed.gz
https://www.encodeproject.org/files/ENCFF736AMQ/@@download/ENCFF736AMQ.bed.gz
https://www.encodeproject.org/files/ENCFF414VZK/@@download/ENCFF414VZK.bed.gz
https://www.encodeproject.org/files/ENCFF787ZAY/@@download/ENCFF787ZAY.bed.gz
https://www.encodeproject.org/files/ENCFF153LET/@@download/ENCFF153LET.bed.gz
https://www.encodeproject.org/files/ENCFF910JLQ/@@download/ENCFF910JLQ.bed.gz
https://www.encodeproject.org/files/ENCFF001SQS/@@download/ENCFF001SQS.bed.gz
https://www.encodeproject.org/files/ENCFF001WIB/@@download/ENCFF001WIB.bed.gz
https://www.encodeproject.org/files/ENCFF001WIC/@@download/ENCFF001WIC.bed.gz
https://www.encodeproject.org/files/ENCFF716BEY/@@download/ENCFF716BEY.bed.gz
https://www.encodeproject.org/files/ENCFF115KOG/@@download/ENCFF115KOG.bed.gz
https://www.encodeproject.org/files/ENCFF272AFT/@@download/ENCFF272AFT.bed.gz
https://www.encodeproject.org/files/ENCFF764ODA/@@download/ENCFF764ODA.bed.gz
https://www.encodeproject.org/files/ENCFF460PHQ/@@download/ENCFF460PHQ.bed.gz
https://www.encodeproject.org/files/ENCFF503RUE/@@download/ENCFF503RUE.bed.gz
https://www.encodeproject.org/files/ENCFF674UST/@@download/ENCFF674UST.bed.gz
https://www.encodeproject.org/files/ENCFF524ACB/@@download/ENCFF524ACB.bed.gz
https://www.encodeproject.org/files/ENCFF749YMK/@@download/ENCFF749YMK.bed.gz
https://www.encodeproject.org/files/ENCFF119HDI/@@download/ENCFF119HDI.bed.gz
https://www.encodeproject.org/files/ENCFF262INI/@@download/ENCFF262INI.bed.gz
https://www.encodeproject.org/files/ENCFF870FYH/@@download/ENCFF870FYH.bed.gz
https://www.encodeproject.org/files/ENCFF848RML/@@download/ENCFF848RML.bed.gz
https://www.encodeproject.org/files/ENCFF413NVW/@@download/ENCFF413NVW.bed.gz
https://www.encodeproject.org/files/ENCFF806JNR/@@download/ENCFF806JNR.bed.gz
https://www.encodeproject.org/files/ENCFF216PTJ/@@download/ENCFF216PTJ.bed.gz
https://www.encodeproject.org/files/ENCFF586ZED/@@download/ENCFF586ZED.bed.gz
https://www.encodeproject.org/files/ENCFF066WIR/@@download/ENCFF066WIR.bed.gz
https://www.encodeproject.org/files/ENCFF221AJA/@@download/ENCFF221AJA.bed.gz
https://www.encodeproject.org/files/ENCFF984RKH/@@download/ENCFF984RKH.bed.gz
https://www.encodeproject.org/files/ENCFF178AYH/@@download/ENCFF178AYH.bed.gz
https://www.encodeproject.org/files/ENCFF459DTS/@@download/ENCFF459DTS.bed.gz
https://www.encodeproject.org/files/ENCFF652JYR/@@download/ENCFF652JYR.bed.gz
https://www.encodeproject.org/files/ENCFF402SZW/@@download/ENCFF402SZW.bed.gz
https://www.encodeproject.org/files/ENCFF898HGX/@@download/ENCFF898HGX.bed.gz
https://www.encodeproject.org/files/ENCFF109RSW/@@download/ENCFF109RSW.bed.gz
https://www.encodeproject.org/files/ENCFF276EBZ/@@download/ENCFF276EBZ.bed.gz
https://www.encodeproject.org/files/ENCFF988HSM/@@download/ENCFF988HSM.bed.gz
https://www.encodeproject.org/files/ENCFF947JNC/@@download/ENCFF947JNC.bed.gz
https://www.encodeproject.org/files/ENCFF813RJG/@@download/ENCFF813RJG.bed.gz
https://www.encodeproject.org/files/ENCFF628EFJ/@@download/ENCFF628EFJ.bed.gz
https://www.encodeproject.org/files/ENCFF133YNT/@@download/ENCFF133YNT.bed.gz
https://www.encodeproject.org/files/ENCFF671FOX/@@download/ENCFF671FOX.bed.gz
https://www.encodeproject.org/files/ENCFF767VXO/@@download/ENCFF767VXO.bed.gz
https://www.encodeproject.org/files/ENCFF941EJJ/@@download/ENCFF941EJJ.bed.gz
https://www.encodeproject.org/files/ENCFF036PGY/@@download/ENCFF036PGY.bed.gz
https://www.encodeproject.org/files/ENCFF828HED/@@download/ENCFF828HED.bed.gz
https://www.encodeproject.org/files/ENCFF828TWL/@@download/ENCFF828TWL.bed.gz
https://www.encodeproject.org/files/ENCFF259XYI/@@download/ENCFF259XYI.bed.gz
https://www.encodeproject.org/files/ENCFF661AIO/@@download/ENCFF661AIO.bed.gz
https://www.encodeproject.org/files/ENCFF001UZH/@@download/ENCFF001UZH.bed.gz
https://www.encodeproject.org/files/ENCFF416QZR/@@download/ENCFF416QZR.bed.gz
https://www.encodeproject.org/files/ENCFF653DQL/@@download/ENCFF653DQL.bed.gz

https://www.encodeproject.org/files/ENCFF523IIS/@@download/ENCFF523IIS.bed.gz
https://www.encodeproject.org/files/ENCFF360KYS/@@download/ENCFF360KYS.bed.gz
https://www.encodeproject.org/files/ENCFF312YEI/@@download/ENCFF312YEI.bed.gz
https://www.encodeproject.org/files/ENCFF982JMW/@@download/ENCFF982JMW.bed.gz
https://www.encodeproject.org/files/ENCFF642HTL/@@download/ENCFF642HTL.bed.gz
https://www.encodeproject.org/files/ENCFF289XNF/@@download/ENCFF289XNF.bed.gz
https://www.encodeproject.org/files/ENCFF355SRP/@@download/ENCFF355SRP.bed.gz
https://www.encodeproject.org/files/ENCFF053GCY/@@download/ENCFF053GCY.bed.gz
https://www.encodeproject.org/files/ENCFF718BDX/@@download/ENCFF718BDX.bed.gz
https://www.encodeproject.org/files/ENCFF100SUV/@@download/ENCFF100SUV.bed.gz
https://www.encodeproject.org/files/ENCFF698AUI/@@download/ENCFF698AUI.bed.gz
https://www.encodeproject.org/files/ENCFF663INK/@@download/ENCFF663INK.bed.gz
https://www.encodeproject.org/files/ENCFF170HDL/@@download/ENCFF170HDL.bed.gz
https://www.encodeproject.org/files/ENCFF134WKG/@@download/ENCFF134WKG.bed.gz
https://www.encodeproject.org/files/ENCFF182YXK/@@download/ENCFF182YXK.bed.gz
https://www.encodeproject.org/files/ENCFF474MZJ/@@download/ENCFF474MZJ.bed.gz
https://www.encodeproject.org/files/ENCFF357RAP/@@download/ENCFF357RAP.bed.gz
https://www.encodeproject.org/files/ENCFF266ADI/@@download/ENCFF266ADI.bed.gz
https://www.encodeproject.org/files/ENCFF375DHF/@@download/ENCFF375DHF.bed.gz
https://www.encodeproject.org/files/ENCFF346DVN/@@download/ENCFF346DVN.bed.gz
https://www.encodeproject.org/files/ENCFF994ALS/@@download/ENCFF994ALS.bed.gz
https://www.encodeproject.org/files/ENCFF397QTB/@@download/ENCFF397QTB.bed.gz
https://www.encodeproject.org/files/ENCFF342IEP/@@download/ENCFF342IEP.bed.gz
https://www.encodeproject.org/files/ENCFF878HUC/@@download/ENCFF878HUC.bed.gz
https://www.encodeproject.org/files/ENCFF753HBM/@@download/ENCFF753HBM.bed.gz
https://www.encodeproject.org/files/ENCFF454NTN/@@download/ENCFF454NTN.bed.gz
https://www.encodeproject.org/files/ENCFF001SRQ/@@download/ENCFF001SRQ.bed.gz
https://www.encodeproject.org/files/ENCFF001WMD/@@download/ENCFF001WMD.bed.gz
https://www.encodeproject.org/files/ENCFF001WME/@@download/ENCFF001WME.bed.gz
https://www.encodeproject.org/files/ENCFF654ASP/@@download/ENCFF654ASP.bed.gz
https://www.encodeproject.org/files/ENCFF548ZXW/@@download/ENCFF548ZXW.bed.gz
https://www.encodeproject.org/files/ENCFF752VHK/@@download/ENCFF752VHK.bed.gz
https://www.encodeproject.org/files/ENCFF358CBP/@@download/ENCFF358CBP.bed.gz
https://www.encodeproject.org/files/ENCFF641BJK/@@download/ENCFF641BJK.bed.gz
https://www.encodeproject.org/files/ENCFF997TBX/@@download/ENCFF997TBX.bed.gz
https://www.encodeproject.org/files/ENCFF948IZD/@@download/ENCFF948IZD.bed.gz
https://www.encodeproject.org/files/ENCFF895EXH/@@download/ENCFF895EXH.bed.gz
https://www.encodeproject.org/files/ENCFF269ORH/@@download/ENCFF269ORH.bed.gz
https://www.encodeproject.org/files/ENCFF750YPR/@@download/ENCFF750YPR.bed.gz
https://www.encodeproject.org/files/ENCFF423OSL/@@download/ENCFF423OSL.bed.gz
https://www.encodeproject.org/files/ENCFF239LDT/@@download/ENCFF239LDT.bed.gz
https://www.encodeproject.org/files/ENCFF376YIY/@@download/ENCFF376YIY.bed.gz
https://www.encodeproject.org/files/ENCFF587YNA/@@download/ENCFF587YNA.bed.gz
https://www.encodeproject.org/files/ENCFF180AEX/@@download/ENCFF180AEX.bed.gz
https://www.encodeproject.org/files/ENCFF382VKO/@@download/ENCFF382VKO.bed.gz
https://www.encodeproject.org/files/ENCFF210YXP/@@download/ENCFF210YXP.bed.gz
https://www.encodeproject.org/files/ENCFF080UTT/@@download/ENCFF080UTT.bed.gz
https://www.encodeproject.org/files/ENCFF966HTZ/@@download/ENCFF966HTZ.bed.gz
https://www.encodeproject.org/files/ENCFF447KGU/@@download/ENCFF447KGU.bed.gz
https://www.encodeproject.org/files/ENCFF047JNI/@@download/ENCFF047JNI.bed.gz
https://www.encodeproject.org/files/ENCFF658VUR/@@download/ENCFF658VUR.bed.gz
https://www.encodeproject.org/files/ENCFF987DJP/@@download/ENCFF987DJP.bed.gz
https://www.encodeproject.org/files/ENCFF435GTP/@@download/ENCFF435GTP.bed.gz

Supplementary Table 1

https://www.encodeproject.org/files/ENCFF059GGU/@@download/ENCFF059GGU.bed.gz
https://www.encodeproject.org/files/ENCFF612ZBD/@@download/ENCFF612ZBD.bed.gz
https://www.encodeproject.org/files/ENCFF036JUB/@@download/ENCFF036JUB.bed.gz
https://www.encodeproject.org/files/ENCFF313UXI/@@download/ENCFF313UXI.bed.gz
https://www.encodeproject.org/files/ENCFF509FBR/@@download/ENCFF509FBR.bed.gz
https://www.encodeproject.org/files/ENCFF195YWM/@@download/ENCFF195YWM.bed.gz
https://www.encodeproject.org/files/ENCFF378QVC/@@download/ENCFF378QVC.bed.gz
https://www.encodeproject.org/files/ENCFF311IIU/@@download/ENCFF311IIU.bed.gz
https://www.encodeproject.org/files/ENCFF387LWE/@@download/ENCFF387LWE.bed.gz
https://www.encodeproject.org/files/ENCFF494EIC/@@download/ENCFF494EIC.bed.gz
https://www.encodeproject.org/files/ENCFF033UGB/@@download/ENCFF033UGB.bed.gz
https://www.encodeproject.org/files/ENCFF120DNW/@@download/ENCFF120DNW.bed.gz
https://www.encodeproject.org/files/ENCFF813JWJ/@@download/ENCFF813JWJ.bed.gz
https://www.encodeproject.org/files/ENCFF986VDP/@@download/ENCFF986VDP.bed.gz
https://www.encodeproject.org/files/ENCFF716YVE/@@download/ENCFF716YVE.bed.gz
https://www.encodeproject.org/files/ENCFF087QAV/@@download/ENCFF087QAV.bed.gz
https://www.encodeproject.org/files/ENCFF456LZZ/@@download/ENCFF456LZZ.bed.gz
https://www.encodeproject.org/files/ENCFF459XGI/@@download/ENCFF459XGI.bed.gz
https://www.encodeproject.org/files/ENCFF115CZG/@@download/ENCFF115CZG.bed.gz
https://www.encodeproject.org/files/ENCFF173ZUV/@@download/ENCFF173ZUV.bed.gz
https://www.encodeproject.org/files/ENCFF393ARF/@@download/ENCFF393ARF.bed.gz
https://www.encodeproject.org/files/ENCFF023ZMS/@@download/ENCFF023ZMS.bed.gz
https://www.encodeproject.org/files/ENCFF678GPZ/@@download/ENCFF678GPZ.bed.gz
https://www.encodeproject.org/files/ENCFF260LTK/@@download/ENCFF260LTK.bed.gz
https://www.encodeproject.org/files/ENCFF198MLE/@@download/ENCFF198MLE.bed.gz
https://www.encodeproject.org/files/ENCFF407GRH/@@download/ENCFF407GRH.bed.gz
https://www.encodeproject.org/files/ENCFF155DZV/@@download/ENCFF155DZV.bed.gz
https://www.encodeproject.org/files/ENCFF667TQB/@@download/ENCFF667TQB.bed.gz
https://www.encodeproject.org/files/ENCFF614KFD/@@download/ENCFF614KFD.bed.gz
https://www.encodeproject.org/files/ENCFF082ISN/@@download/ENCFF082ISN.bed.gz
https://www.encodeproject.org/files/ENCFF347ARK/@@download/ENCFF347ARK.bed.gz
https://www.encodeproject.org/files/ENCFF966PVO/@@download/ENCFF966PVO.bed.gz
https://www.encodeproject.org/files/ENCFF391CGC/@@download/ENCFF391CGC.bed.gz
https://www.encodeproject.org/files/ENCFF543QXW/@@download/ENCFF543QXW.bed.gz
https://www.encodeproject.org/files/ENCFF957LQJ/@@download/ENCFF957LQJ.bed.gz
https://www.encodeproject.org/files/ENCFF358SYQ/@@download/ENCFF358SYQ.bed.gz
https://www.encodeproject.org/files/ENCFF024SOP/@@download/ENCFF024SOP.bed.gz
https://www.encodeproject.org/files/ENCFF744LRT/@@download/ENCFF744LRT.bed.gz
https://www.encodeproject.org/files/ENCFF564OAG/@@download/ENCFF564OAG.bed.gz
https://www.encodeproject.org/files/ENCFF645FNZ/@@download/ENCFF645FNZ.bed.gz
https://www.encodeproject.org/files/ENCFF172GSX/@@download/ENCFF172GSX.bed.gz
https://www.encodeproject.org/files/ENCFF917VNK/@@download/ENCFF917VNK.bed.gz
https://www.encodeproject.org/files/ENCFF412EJZ/@@download/ENCFF412EJZ.bed.gz
https://www.encodeproject.org/files/ENCFF338UFC/@@download/ENCFF338UFC.bed.gz
https://www.encodeproject.org/files/ENCFF569GSL/@@download/ENCFF569GSL.bed.gz
https://www.encodeproject.org/files/ENCFF167XAJ/@@download/ENCFF167XAJ.bed.gz
https://www.encodeproject.org/files/ENCFF360LKQ/@@download/ENCFF360LKQ.bed.gz
https://www.encodeproject.org/files/ENCFF662JOI/@@download/ENCFF662JOI.bed.gz
https://www.encodeproject.org/files/ENCFF262FPE/@@download/ENCFF262FPE.bed.gz
https://www.encodeproject.org/files/ENCFF193PBQ/@@download/ENCFF193PBQ.bed.gz
https://www.encodeproject.org/files/ENCFF179NOL/@@download/ENCFF179NOL.bed.gz
https://www.encodeproject.org/files/ENCFF868AXL/@@download/ENCFF868AXL.bed.gz
https://www.encodeproject.org/files/ENCFF082YXC/@@download/ENCFF082YXC.bed.gz

https://www.encodeproject.org/files/ENCFF324JDA/@@download/ENCFF324JDA.bed.gz
https://www.encodeproject.org/files/ENCFF827CEJ/@@download/ENCFF827CEJ.bed.gz
https://www.encodeproject.org/files/ENCFF360OCL/@@download/ENCFF360OCL.bed.gz
https://www.encodeproject.org/files/ENCFF949YFS/@@download/ENCFF949YFS.bed.gz
https://www.encodeproject.org/files/ENCFF717OZX/@@download/ENCFF717OZX.bed.gz
https://www.encodeproject.org/files/ENCFF355WMJ/@@download/ENCFF355WMJ.bed.gz
https://www.encodeproject.org/files/ENCFF433EYA/@@download/ENCFF433EYA.bed.gz
https://www.encodeproject.org/files/ENCFF929WJR/@@download/ENCFF929WJR.bed.gz
https://www.encodeproject.org/files/ENCFF186VEE/@@download/ENCFF186VEE.bed.gz
https://www.encodeproject.org/files/ENCFF442VET/@@download/ENCFF442VET.bed.gz
https://www.encodeproject.org/files/ENCFF816CNZ/@@download/ENCFF816CNZ.bed.gz
https://www.encodeproject.org/files/ENCFF807UWE/@@download/ENCFF807UWE.bed.gz
https://www.encodeproject.org/files/ENCFF999MRB/@@download/ENCFF999MRB.bed.gz
https://www.encodeproject.org/files/ENCFF688ZWO/@@download/ENCFF688ZWO.bed.gz
https://www.encodeproject.org/files/ENCFF215UXZ/@@download/ENCFF215UXZ.bed.gz
https://www.encodeproject.org/files/ENCFF641CNI/@@download/ENCFF641CNI.bed.gz
https://www.encodeproject.org/files/ENCFF896PXG/@@download/ENCFF896PXG.bed.gz
https://www.encodeproject.org/files/ENCFF745WLV/@@download/ENCFF745WLV.bed.gz
https://www.encodeproject.org/files/ENCFF592FVN/@@download/ENCFF592FVN.bed.gz
https://www.encodeproject.org/files/ENCFF638BPG/@@download/ENCFF638BPG.bed.gz
https://www.encodeproject.org/files/ENCFF588APX/@@download/ENCFF588APX.bed.gz
https://www.encodeproject.org/files/ENCFF067CKQ/@@download/ENCFF067CKQ.bed.gz
https://www.encodeproject.org/files/ENCFF241RJJ/@@download/ENCFF241RJJ.bed.gz
https://www.encodeproject.org/files/ENCFF595YXV/@@download/ENCFF595YXV.bed.gz
https://www.encodeproject.org/files/ENCFF981HPR/@@download/ENCFF981HPR.bed.gz
https://www.encodeproject.org/files/ENCFF709SFY/@@download/ENCFF709SFY.bed.gz
https://www.encodeproject.org/files/ENCFF727JJT/@@download/ENCFF727JJT.bed.gz
https://www.encodeproject.org/files/ENCFF431OVX/@@download/ENCFF431OVX.bed.gz
https://www.encodeproject.org/files/ENCFF001UZC/@@download/ENCFF001UZC.bed.gz
https://www.encodeproject.org/files/ENCFF443WZT/@@download/ENCFF443WZT.bed.gz
https://www.encodeproject.org/files/ENCFF838FGZ/@@download/ENCFF838FGZ.bed.gz
https://www.encodeproject.org/files/ENCFF525QGN/@@download/ENCFF525QGN.bed.gz
https://www.encodeproject.org/files/ENCFF470FFR/@@download/ENCFF470FFR.bed.gz
https://www.encodeproject.org/files/ENCFF001WCO/@@download/ENCFF001WCO.bed.gz
https://www.encodeproject.org/files/ENCFF001WTE/@@download/ENCFF001WTE.bed.gz
https://www.encodeproject.org/files/ENCFF001WCQ/@@download/ENCFF001WCQ.bed.gz
https://www.encodeproject.org/files/ENCFF001WTF/@@download/ENCFF001WTF.bed.gz
https://www.encodeproject.org/files/ENCFF920ELS/@@download/ENCFF920ELS.bed.gz
https://www.encodeproject.org/files/ENCFF280ZRI/@@download/ENCFF280ZRI.bed.gz
https://www.encodeproject.org/files/ENCFF350MDQ/@@download/ENCFF350MDQ.bed.gz
https://www.encodeproject.org/files/ENCFF344XVX/@@download/ENCFF344XVX.bed.gz
https://www.encodeproject.org/files/ENCFF909VZN/@@download/ENCFF909VZN.bed.gz
https://www.encodeproject.org/files/ENCFF415CSY/@@download/ENCFF415CSY.bed.gz
https://www.encodeproject.org/files/ENCFF387TJX/@@download/ENCFF387TJX.bed.gz
https://www.encodeproject.org/files/ENCFF159CHE/@@download/ENCFF159CHE.bed.gz
https://www.encodeproject.org/files/ENCFF618EIJ/@@download/ENCFF618EIJ.bed.gz
https://www.encodeproject.org/files/ENCFF843ZSC/@@download/ENCFF843ZSC.bed.gz
https://www.encodeproject.org/files/ENCFF010UMG/@@download/ENCFF010UMG.bed.gz
https://www.encodeproject.org/files/ENCFF180AQM/@@download/ENCFF180AQM.bed.gz
https://www.encodeproject.org/files/ENCFF916PTD/@@download/ENCFF916PTD.bed.gz
https://www.encodeproject.org/files/ENCFF890SKU/@@download/ENCFF890SKU.bed.gz
https://www.encodeproject.org/files/ENCFF523INF/@@download/ENCFF523INF.bed.gz
https://www.encodeproject.org/files/ENCFF122TCQ/@@download/ENCFF122TCQ.bed.gz

Supplementary Table 1

https://www.encodeproject.org/files/ENCFF200HLO/@@download/ENCFF200HLO.bed.gz
https://www.encodeproject.org/files/ENCFF670JBM/@@download/ENCFF670JBM.bed.gz
https://www.encodeproject.org/files/ENCFF737MBR/@@download/ENCFF737MBR.bed.gz
https://www.encodeproject.org/files/ENCFF080NAO/@@download/ENCFF080NAO.bed.gz
https://www.encodeproject.org/files/ENCFF846OZD/@@download/ENCFF846OZD.bed.gz
https://www.encodeproject.org/files/ENCFF059VUS/@@download/ENCFF059VUS.bed.gz
https://www.encodeproject.org/files/ENCFF318ASA/@@download/ENCFF318ASA.bed.gz
https://www.encodeproject.org/files/ENCFF212WWF/@@download/ENCFF212WWF.bed.gz
https://www.encodeproject.org/files/ENCFF111XGH/@@download/ENCFF111XGH.bed.gz
https://www.encodeproject.org/files/ENCFF110CYC/@@download/ENCFF110CYC.bed.gz
https://www.encodeproject.org/files/ENCFF457SNT/@@download/ENCFF457SNT.bed.gz
https://www.encodeproject.org/files/ENCFF617MXO/@@download/ENCFF617MXO.bed.gz
https://www.encodeproject.org/files/ENCFF733HEF/@@download/ENCFF733HEF.bed.gz
https://www.encodeproject.org/files/ENCFF314KLM/@@download/ENCFF314KLM.bed.gz
https://www.encodeproject.org/files/ENCFF274NTF/@@download/ENCFF274NTF.bed.gz
https://www.encodeproject.org/files/ENCFF917YWY/@@download/ENCFF917YWY.bed.gz
https://www.encodeproject.org/files/ENCFF227RVA/@@download/ENCFF227RVA.bed.gz
https://www.encodeproject.org/files/ENCFF453ZDK/@@download/ENCFF453ZDK.bed.gz
https://www.encodeproject.org/files/ENCFF240WZG/@@download/ENCFF240WZG.bed.gz
https://www.encodeproject.org/files/ENCFF193WYH/@@download/ENCFF193WYH.bed.gz
https://www.encodeproject.org/files/ENCFF505DCA/@@download/ENCFF505DCA.bed.gz
https://www.encodeproject.org/files/ENCFF191WFG/@@download/ENCFF191WFG.bed.gz
https://www.encodeproject.org/files/ENCFF235WMX/@@download/ENCFF235WMX.bed.gz
https://www.encodeproject.org/files/ENCFF907KBL/@@download/ENCFF907KBL.bed.gz
https://www.encodeproject.org/files/ENCFF603QML/@@download/ENCFF603QML.bed.gz
https://www.encodeproject.org/files/ENCFF440GUP/@@download/ENCFF440GUP.bed.gz
https://www.encodeproject.org/files/ENCFF579CGP/@@download/ENCFF579CGP.bed.gz
https://www.encodeproject.org/files/ENCFF304OAZ/@@download/ENCFF304OAZ.bed.gz
https://www.encodeproject.org/files/ENCFF209MOG/@@download/ENCFF209MOG.bed.gz
https://www.encodeproject.org/files/ENCFF040ZVN/@@download/ENCFF040ZVN.bed.gz
https://www.encodeproject.org/files/ENCFF691XOS/@@download/ENCFF691XOS.bed.gz
https://www.encodeproject.org/files/ENCFF358YMC/@@download/ENCFF358YMC.bed.gz
https://www.encodeproject.org/files/ENCFF599BMS/@@download/ENCFF599BMS.bed.gz
https://www.encodeproject.org/files/ENCFF446VBC/@@download/ENCFF446VBC.bed.gz
https://www.encodeproject.org/files/ENCFF668VKT/@@download/ENCFF668VKT.bed.gz
https://www.encodeproject.org/files/ENCFF191HPU/@@download/ENCFF191HPU.bed.gz
https://www.encodeproject.org/files/ENCFF684HBU/@@download/ENCFF684HBU.bed.gz
https://www.encodeproject.org/files/ENCFF415XZW/@@download/ENCFF415XZW.bed.gz
https://www.encodeproject.org/files/ENCFF438EZP/@@download/ENCFF438EZP.bed.gz
https://www.encodeproject.org/files/ENCFF063IUG/@@download/ENCFF063IUG.bed.gz
https://www.encodeproject.org/files/ENCFF228QBR/@@download/ENCFF228QBR.bed.gz
https://www.encodeproject.org/files/ENCFF564MST/@@download/ENCFF564MST.bed.gz
https://www.encodeproject.org/files/ENCFF915XQC/@@download/ENCFF915XQC.bed.gz
https://www.encodeproject.org/files/ENCFF708WKP/@@download/ENCFF708WKP.bed.gz
https://www.encodeproject.org/files/ENCFF615LEO/@@download/ENCFF615LEO.bed.gz
https://www.encodeproject.org/files/ENCFF326SSU/@@download/ENCFF326SSU.bed.gz
https://www.encodeproject.org/files/ENCFF103ZMI/@@download/ENCFF103ZMI.bed.gz
https://www.encodeproject.org/files/ENCFF206QUY/@@download/ENCFF206QUY.bed.gz
https://www.encodeproject.org/files/ENCFF809PKZ/@@download/ENCFF809PKZ.bed.gz
https://www.encodeproject.org/files/ENCFF355MRH/@@download/ENCFF355MRH.bed.gz
https://www.encodeproject.org/files/ENCFF950EWN/@@download/ENCFF950EWN.bed.gz
https://www.encodeproject.org/files/ENCFF026XWM/@@download/ENCFF026XWM.bed.gz
https://www.encodeproject.org/files/ENCFF796BQH/@@download/ENCFF796BQH.bed.gz

Supplementary Table 1

https://www.encodeproject.org/files/ENCFF874BCK/@@download/ENCFF874BCK.bed.gz
https://www.encodeproject.org/files/ENCFF983MXB/@@download/ENCFF983MXB.bed.gz
https://www.encodeproject.org/files/ENCFF718DFS/@@download/ENCFF718DFS.bed.gz
https://www.encodeproject.org/files/ENCFF727YVM/@@download/ENCFF727YVM.bed.gz
https://www.encodeproject.org/files/ENCFF877COU/@@download/ENCFF877COU.bed.gz
https://www.encodeproject.org/files/ENCFF402XGX/@@download/ENCFF402XGX.bed.gz
https://www.encodeproject.org/files/ENCFF144MJE/@@download/ENCFF144MJE.bed.gz
https://www.encodeproject.org/files/ENCFF795QUJ/@@download/ENCFF795QUJ.bed.gz
https://www.encodeproject.org/files/ENCFF295UQG/@@download/ENCFF295UQG.bed.gz
https://www.encodeproject.org/files/ENCFF354YEA/@@download/ENCFF354YEA.bed.gz
https://www.encodeproject.org/files/ENCFF914YLY/@@download/ENCFF914YLY.bed.gz
https://www.encodeproject.org/files/ENCFF762YAX/@@download/ENCFF762YAX.bed.gz
https://www.encodeproject.org/files/ENCFF264MZZ/@@download/ENCFF264MZZ.bed.gz
https://www.encodeproject.org/files/ENCFF617LBQ/@@download/ENCFF617LBQ.bed.gz
https://www.encodeproject.org/files/ENCFF053SED/@@download/ENCFF053SED.bed.gz
https://www.encodeproject.org/files/ENCFF537FMS/@@download/ENCFF537FMS.bed.gz
https://www.encodeproject.org/files/ENCFF197CRC/@@download/ENCFF197CRC.bed.gz
https://www.encodeproject.org/files/ENCFF096VYH/@@download/ENCFF096VYH.bed.gz
https://www.encodeproject.org/files/ENCFF622ITZ/@@download/ENCFF622ITZ.bed.gz
https://www.encodeproject.org/files/ENCFF629UPF/@@download/ENCFF629UPF.bed.gz
https://www.encodeproject.org/files/ENCFF051UGM/@@download/ENCFF051UGM.bed.gz
https://www.encodeproject.org/files/ENCFF924NKC/@@download/ENCFF924NKC.bed.gz
https://www.encodeproject.org/files/ENCFF607HYD/@@download/ENCFF607HYD.bed.gz
https://www.encodeproject.org/files/ENCFF554QRP/@@download/ENCFF554QRP.bed.gz
https://www.encodeproject.org/files/ENCFF390KQH/@@download/ENCFF390KQH.bed.gz
https://www.encodeproject.org/files/ENCFF120MDN/@@download/ENCFF120MDN.bed.gz
https://www.encodeproject.org/files/ENCFF436EPI/@@download/ENCFF436EPI.bed.gz
https://www.encodeproject.org/files/ENCFF014YBK/@@download/ENCFF014YBK.bed.gz
https://www.encodeproject.org/files/ENCFF630TFU/@@download/ENCFF630TFU.bed.gz
https://www.encodeproject.org/files/ENCFF949WQH/@@download/ENCFF949WQH.bed.gz
https://www.encodeproject.org/files/ENCFF182ZYZ/@@download/ENCFF182ZYZ.bed.gz
https://www.encodeproject.org/files/ENCFF056XBS/@@download/ENCFF056XBS.bed.gz
https://www.encodeproject.org/files/ENCFF012SAA/@@download/ENCFF012SAA.bed.gz
https://www.encodeproject.org/files/ENCFF982PAZ/@@download/ENCFF982PAZ.bed.gz
https://www.encodeproject.org/files/ENCFF970PWO/@@download/ENCFF970PWO.bed.gz
https://www.encodeproject.org/files/ENCFF105SNJ/@@download/ENCFF105SNJ.bed.gz
https://www.encodeproject.org/files/ENCFF047DWA/@@download/ENCFF047DWA.bed.gz
https://www.encodeproject.org/files/ENCFF371AFC/@@download/ENCFF371AFC.bed.gz
https://www.encodeproject.org/files/ENCFF531SWI/@@download/ENCFF531SWI.bed.gz
https://www.encodeproject.org/files/ENCFF602DIZ/@@download/ENCFF602DIZ.bed.gz
https://www.encodeproject.org/files/ENCFF986ZJA/@@download/ENCFF986ZJA.bed.gz
https://www.encodeproject.org/files/ENCFF514DWJ/@@download/ENCFF514DWJ.bed.gz
https://www.encodeproject.org/files/ENCFF354ROB/@@download/ENCFF354ROB.bed.gz
https://www.encodeproject.org/files/ENCFF115PGQ/@@download/ENCFF115PGQ.bed.gz
https://www.encodeproject.org/files/ENCFF805BEX/@@download/ENCFF805BEX.bed.gz
https://www.encodeproject.org/files/ENCFF105ADZ/@@download/ENCFF105ADZ.bed.gz
https://www.encodeproject.org/files/ENCFF802GPU/@@download/ENCFF802GPU.bed.gz
https://www.encodeproject.org/files/ENCFF096TTO/@@download/ENCFF096TTO.bed.gz
https://www.encodeproject.org/files/ENCFF462QBZ/@@download/ENCFF462QBZ.bed.gz
https://www.encodeproject.org/files/ENCFF937NUZ/@@download/ENCFF937NUZ.bed.gz
https://www.encodeproject.org/files/ENCFF070NZJ/@@download/ENCFF070NZJ.bed.gz
https://www.encodeproject.org/files/ENCFF009WLN/@@download/ENCFF009WLN.bed.gz
https://www.encodeproject.org/files/ENCFF249FGP/@@download/ENCFF249FGP.bed.gz

Supplementary Table 1

https://www.encodeproject.org/files/ENCFF031MPR/@@download/ENCFF031MPR.bed.gz
https://www.encodeproject.org/files/ENCFF798QOA/@@download/ENCFF798QOA.bed.gz
https://www.encodeproject.org/files/ENCFF264PJZ/@@download/ENCFF264PJZ.bed.gz
https://www.encodeproject.org/files/ENCFF175BCP/@@download/ENCFF175BCP.bed.gz
https://www.encodeproject.org/files/ENCFF401SKP/@@download/ENCFF401SKP.bed.gz
https://www.encodeproject.org/files/ENCFF543KDP/@@download/ENCFF543KDP.bed.gz
https://www.encodeproject.org/files/ENCFF222ZZI/@@download/ENCFF222ZZI.bed.gz
https://www.encodeproject.org/files/ENCFF502FQS/@@download/ENCFF502FQS.bed.gz
https://www.encodeproject.org/files/ENCFF116WYF/@@download/ENCFF116WYF.bed.gz
https://www.encodeproject.org/files/ENCFF749DUT/@@download/ENCFF749DUT.bed.gz
https://www.encodeproject.org/files/ENCFF637ZID/@@download/ENCFF637ZID.bed.gz
https://www.encodeproject.org/files/ENCFF734PPZ/@@download/ENCFF734PPZ.bed.gz
https://www.encodeproject.org/files/ENCFF191JBX/@@download/ENCFF191JBX.bed.gz
https://www.encodeproject.org/files/ENCFF460YVL/@@download/ENCFF460YVL.bed.gz
https://www.encodeproject.org/files/ENCFF819MDU/@@download/ENCFF819MDU.bed.gz
https://www.encodeproject.org/files/ENCFF041NEG/@@download/ENCFF041NEG.bed.gz
https://www.encodeproject.org/files/ENCFF888NOP/@@download/ENCFF888NOP.bed.gz
https://www.encodeproject.org/files/ENCFF265KNN/@@download/ENCFF265KNN.bed.gz
https://www.encodeproject.org/files/ENCFF134EOH/@@download/ENCFF134EOH.bed.gz
https://www.encodeproject.org/files/ENCFF148PCY/@@download/ENCFF148PCY.bed.gz
https://www.encodeproject.org/files/ENCFF802PVJ/@@download/ENCFF802PVJ.bed.gz
https://www.encodeproject.org/files/ENCFF994YDK/@@download/ENCFF994YDK.bed.gz
https://www.encodeproject.org/files/ENCFF996VLV/@@download/ENCFF996VLV.bed.gz
https://www.encodeproject.org/files/ENCFF901XEJ/@@download/ENCFF901XEJ.bed.gz
https://www.encodeproject.org/files/ENCFF485NAD/@@download/ENCFF485NAD.bed.gz
https://www.encodeproject.org/files/ENCFF158IRO/@@download/ENCFF158IRO.bed.gz
https://www.encodeproject.org/files/ENCFF696ZBX/@@download/ENCFF696ZBX.bed.gz
https://www.encodeproject.org/files/ENCFF246TUN/@@download/ENCFF246TUN.bed.gz
https://www.encodeproject.org/files/ENCFF588SBI/@@download/ENCFF588SBI.bed.gz
https://www.encodeproject.org/files/ENCFF508UQC/@@download/ENCFF508UQC.bed.gz
https://www.encodeproject.org/files/ENCFF464PRA/@@download/ENCFF464PRA.bed.gz
https://www.encodeproject.org/files/ENCFF473LEG/@@download/ENCFF473LEG.bed.gz
https://www.encodeproject.org/files/ENCFF948QHW/@@download/ENCFF948QHW.bed.gz
https://www.encodeproject.org/files/ENCFF549EGS/@@download/ENCFF549EGS.bed.gz
https://www.encodeproject.org/files/ENCFF560YBU/@@download/ENCFF560YBU.bed.gz
https://www.encodeproject.org/files/ENCFF384RMC/@@download/ENCFF384RMC.bed.gz
https://www.encodeproject.org/files/ENCFF589KFW/@@download/ENCFF589KFW.bed.gz
https://www.encodeproject.org/files/ENCFF839FSF/@@download/ENCFF839FSF.bed.gz
https://www.encodeproject.org/files/ENCFF128KCC/@@download/ENCFF128KCC.bed.gz
https://www.encodeproject.org/files/ENCFF098XHW/@@download/ENCFF098XHW.bed.gz
https://www.encodeproject.org/files/ENCFF375XEP/@@download/ENCFF375XEP.bed.gz
https://www.encodeproject.org/files/ENCFF472VBK/@@download/ENCFF472VBK.bed.gz
https://www.encodeproject.org/files/ENCFF875AFU/@@download/ENCFF875AFU.bed.gz
https://www.encodeproject.org/files/ENCFF396OCF/@@download/ENCFF396OCF.bed.gz
https://www.encodeproject.org/files/ENCFF219PSV/@@download/ENCFF219PSV.bed.gz
https://www.encodeproject.org/files/ENCFF945ILS/@@download/ENCFF945ILS.bed.gz
https://www.encodeproject.org/files/ENCFF711IJR/@@download/ENCFF711IJR.bed.gz
https://www.encodeproject.org/files/ENCFF510AYV/@@download/ENCFF510AYV.bed.gz
https://www.encodeproject.org/files/ENCFF602DCV/@@download/ENCFF602DCV.bed.gz
https://www.encodeproject.org/files/ENCFF290OTF/@@download/ENCFF290OTF.bed.gz
https://www.encodeproject.org/files/ENCFF773MYG/@@download/ENCFF773MYG.bed.gz
https://www.encodeproject.org/files/ENCFF631GAR/@@download/ENCFF631GAR.bed.gz
https://www.encodeproject.org/files/ENCFF602XCK/@@download/ENCFF602XCK.bed.gz

Supplementary Table 1

https://www.encodeproject.org/files/ENCFF168SXR/@@download/ENCFF168SXR.bed.gz
https://www.encodeproject.org/files/ENCFF181OFF/@@download/ENCFF181OFF.bed.gz
https://www.encodeproject.org/files/ENCFF138LZU/@@download/ENCFF138LZU.bed.gz
https://www.encodeproject.org/files/ENCFF579QTP/@@download/ENCFF579QTP.bed.gz
https://www.encodeproject.org/files/ENCFF857PAY/@@download/ENCFF857PAY.bed.gz
https://www.encodeproject.org/files/ENCFF213XLZ/@@download/ENCFF213XLZ.bed.gz
https://www.encodeproject.org/files/ENCFF915INZ/@@download/ENCFF915INZ.bed.gz
https://www.encodeproject.org/files/ENCFF971TRF/@@download/ENCFF971TRF.bed.gz
https://www.encodeproject.org/files/ENCFF040WPR/@@download/ENCFF040WPR.bed.gz
https://www.encodeproject.org/files/ENCFF711VFJ/@@download/ENCFF711VFJ.bed.gz
https://www.encodeproject.org/files/ENCFF934EAN/@@download/ENCFF934EAN.bed.gz
https://www.encodeproject.org/files/ENCFF110SZL/@@download/ENCFF110SZL.bed.gz
https://www.encodeproject.org/files/ENCFF188ZFG/@@download/ENCFF188ZFG.bed.gz
https://www.encodeproject.org/files/ENCFF227CMU/@@download/ENCFF227CMU.bed.gz
https://www.encodeproject.org/files/ENCFF639XZY/@@download/ENCFF639XZY.bed.gz
https://www.encodeproject.org/files/ENCFF753RBC/@@download/ENCFF753RBC.bed.gz
https://www.encodeproject.org/files/ENCFF203IXI/@@download/ENCFF203IXI.bed.gz
https://www.encodeproject.org/files/ENCFF795ISF/@@download/ENCFF795ISF.bed.gz
https://www.encodeproject.org/files/ENCFF662ROY/@@download/ENCFF662ROY.bed.gz
https://www.encodeproject.org/files/ENCFF725LKV/@@download/ENCFF725LKV.bed.gz
https://www.encodeproject.org/files/ENCFF359WHG/@@download/ENCFF359WHG.bed.gz
https://www.encodeproject.org/files/ENCFF547UCK/@@download/ENCFF547UCK.bed.gz
https://www.encodeproject.org/files/ENCFF131SCF/@@download/ENCFF131SCF.bed.gz
https://www.encodeproject.org/files/ENCFF026SFK/@@download/ENCFF026SFK.bed.gz
https://www.encodeproject.org/files/ENCFF126JXB/@@download/ENCFF126JXB.bed.gz
https://www.encodeproject.org/files/ENCFF376CNB/@@download/ENCFF376CNB.bed.gz
https://www.encodeproject.org/files/ENCFF758VXS/@@download/ENCFF758VXS.bed.gz
https://www.encodeproject.org/files/ENCFF722CYJ/@@download/ENCFF722CYJ.bed.gz
https://www.encodeproject.org/files/ENCFF425HGL/@@download/ENCFF425HGL.bed.gz
https://www.encodeproject.org/files/ENCFF137KRV/@@download/ENCFF137KRV.bed.gz
https://www.encodeproject.org/files/ENCFF879VBG/@@download/ENCFF879VBG.bed.gz
https://www.encodeproject.org/files/ENCFF273EIR/@@download/ENCFF273EIR.bed.gz
https://www.encodeproject.org/files/ENCFF676RFS/@@download/ENCFF676RFS.bed.gz
https://www.encodeproject.org/files/ENCFF192AJH/@@download/ENCFF192AJH.bed.gz
https://www.encodeproject.org/files/ENCFF667CVN/@@download/ENCFF667CVN.bed.gz
https://www.encodeproject.org/files/ENCFF597GVZ/@@download/ENCFF597GVZ.bed.gz
https://www.encodeproject.org/files/ENCFF722RMF/@@download/ENCFF722RMF.bed.gz
https://www.encodeproject.org/files/ENCFF474JWV/@@download/ENCFF474JWV.bed.gz
https://www.encodeproject.org/files/ENCFF625OKQ/@@download/ENCFF625OKQ.bed.gz
https://www.encodeproject.org/files/ENCFF518QQC/@@download/ENCFF518QQC.bed.gz
https://www.encodeproject.org/files/ENCFF686BLY/@@download/ENCFF686BLY.bed.gz
https://www.encodeproject.org/files/ENCFF382NHI/@@download/ENCFF382NHI.bed.gz
https://www.encodeproject.org/files/ENCFF243MBB/@@download/ENCFF243MBB.bed.gz
https://www.encodeproject.org/files/ENCFF036IFB/@@download/ENCFF036IFB.bed.gz
https://www.encodeproject.org/files/ENCFF296CLY/@@download/ENCFF296CLY.bed.gz
https://www.encodeproject.org/files/ENCFF903ULR/@@download/ENCFF903ULR.bed.gz
https://www.encodeproject.org/files/ENCFF856UQI/@@download/ENCFF856UQI.bed.gz
https://www.encodeproject.org/files/ENCFF745LBF/@@download/ENCFF745LBF.bed.gz
https://www.encodeproject.org/files/ENCFF738IOC/@@download/ENCFF738IOC.bed.gz
https://www.encodeproject.org/files/ENCFF810MDP/@@download/ENCFF810MDP.bed.gz
https://www.encodeproject.org/files/ENCFF001UYC/@@download/ENCFF001UYC.bed.gz
https://www.encodeproject.org/files/ENCFF708SRP/@@download/ENCFF708SRP.bed.gz
https://www.encodeproject.org/files/ENCFF162HDT/@@download/ENCFF162HDT.bed.gz

Supplementary Table 1

https://www.encodeproject.org/files/ENCFF385RNI/@@download/ENCFF385RNI.bed.gz

https://www.encodeproject.org/files/ENCFF866THD/@@download/ENCFF866THD.bed.gz

https://www.encodeproject.org/files/ENCFF406RTA/@@download/ENCFF406RTA.bed.gz

https://www.encodeproject.org/files/ENCFF940SCA/@@download/ENCFF940SCA.bed.gz

https://www.encodeproject.org/files/ENCFF001WBA/@@download/ENCFF001WBA.bed.gz

https://www.encodeproject.org/files/ENCFF001WNC/@@download/ENCFF001WNC.bed.gz

https://www.encodeproject.org/files/ENCFF001WNB/@@download/ENCFF001WNB.bed.gz

https://www.encodeproject.org/files/ENCFF136MYL/@@download/ENCFF136MYL.bed.gz

https://www.encodeproject.org/files/ENCFF406AWN/@@download/ENCFF406AWN.bed.gz

https://www.encodeproject.org/files/ENCFF330HEK/@@download/ENCFF330HEK.bed.gz

https://www.encodeproject.org/files/ENCFF097KBE/@@download/ENCFF097KBE.bed.gz

https://www.encodeproject.org/files/ENCFF967LJZ/@@download/ENCFF967LJZ.bed.gz

https://www.encodeproject.org/files/ENCFF490TJL/@@download/ENCFF490TJL.bed.gz

https://www.encodeproject.org/files/ENCFF645VRE/@@download/ENCFF645VRE.bed.gz

https://www.encodeproject.org/files/ENCFF881SHA/@@download/ENCFF881SHA.bed.gz

https://www.encodeproject.org/files/ENCFF861EYO/@@download/ENCFF861EYO.bed.gz

https://www.encodeproject.org/files/ENCFF820KNU/@@download/ENCFF820KNU.bed.gz

https://www.encodeproject.org/files/ENCFF556WLI/@@download/ENCFF556WLI.bed.gz

https://www.encodeproject.org/files/ENCFF640UDN/@@download/ENCFF640UDN.bed.gz

https://www.encodeproject.org/files/ENCFF634NQJ/@@download/ENCFF634NQJ.bed.gz

https://www.encodeproject.org/files/ENCFF842IEY/@@download/ENCFF842IEY.bed.gz

https://www.encodeproject.org/files/ENCFF953HYB/@@download/ENCFF953HYB.bed.gz

https://www.encodeproject.org/files/ENCFF909LBR/@@download/ENCFF909LBR.bed.gz

https://www.encodeproject.org/files/ENCFF765BJR/@@download/ENCFF765BJR.bed.gz

https://www.encodeproject.org/files/ENCFF744IAP/@@download/ENCFF744IAP.bed.gz

https://www.encodeproject.org/files/ENCFF577ZMC/@@download/ENCFF577ZMC.bed.gz

https://www.encodeproject.org/files/ENCFF475AOQ/@@download/ENCFF475AOQ.bed.gz

https://www.encodeproject.org/files/ENCFF948CVH/@@download/ENCFF948CVH.bed.gz

https://www.encodeproject.org/files/ENCFF111HEY/@@download/ENCFF111HEY.bed.gz

https://www.encodeproject.org/files/ENCFF679QGU/@@download/ENCFF679QGU.bed.gz

https://www.encodeproject.org/files/ENCFF243LFH/@@download/ENCFF243LFH.bed.gz

https://www.encodeproject.org/files/ENCFF291MQR/@@download/ENCFF291MQR.bed.gz

https://www.encodeproject.org/files/ENCFF574LXH/@@download/ENCFF574LXH.bed.gz

https://www.encodeproject.org/files/ENCFF227WZR/@@download/ENCFF227WZR.bed.gz

https://www.encodeproject.org/files/ENCFF236JVB/@@download/ENCFF236JVB.bed.gz

https://www.encodeproject.org/files/ENCFF389WGU/@@download/ENCFF389WGU.bed.gz

https://www.encodeproject.org/files/ENCFF555ZGP/@@download/ENCFF555ZGP.bed.gz

https://www.encodeproject.org/files/ENCFF317VBE/@@download/ENCFF317VBE.bed.gz

https://www.encodeproject.org/files/ENCFF852TKL/@@download/ENCFF852TKL.bed.gz

https://www.encodeproject.org/files/ENCFF991KFV/@@download/ENCFF991KFV.bed.gz

https://www.encodeproject.org/files/ENCFF500AZS/@@download/ENCFF500AZS.bed.gz

https://www.encodeproject.org/files/ENCFF342PZX/@@download/ENCFF342PZX.bed.gz

https://www.encodeproject.org/files/ENCFF498QOJ/@@download/ENCFF498QOJ.bed.gz

https://www.encodeproject.org/files/ENCFF960YSU/@@download/ENCFF960YSU.bed.gz

https://www.encodeproject.org/files/ENCFF916EFR/@@download/ENCFF916EFR.bed.gz

https://www.encodeproject.org/files/ENCFF342TOK/@@download/ENCFF342TOK.bed.gz

https://www.encodeproject.org/files/ENCFF284CPF/@@download/ENCFF284CPF.bed.gz

https://www.encodeproject.org/files/ENCFF417APA/@@download/ENCFF417APA.bed.gz

https://www.encodeproject.org/files/ENCFF189LKP/@@download/ENCFF189LKP.bed.gz

https://www.encodeproject.org/files/ENCFF196JFB/@@download/ENCFF196JFB.bed.gz

https://www.encodeproject.org/files/ENCFF448GNP/@@download/ENCFF448GNP.bed.gz

https://www.encodeproject.org/files/ENCFF190MLU/@@download/ENCFF190MLU.bed.gz

https://www.encodeproject.org/files/ENCFF778TVZ/@@download/ENCFF778TVZ.bed.gz

Supplementary Table 1

https://www.encodeproject.org/files/ENCFF297IRX/@@download/ENCFF297IRX.bed.gz
https://www.encodeproject.org/files/ENCFF871GML/@@download/ENCFF871GML.bed.gz
https://www.encodeproject.org/files/ENCFF320DNQ/@@download/ENCFF320DNQ.bed.gz
https://www.encodeproject.org/files/ENCFF152JIP/@@download/ENCFF152JIP.bed.gz
https://www.encodeproject.org/files/ENCFF390EKL/@@download/ENCFF390EKL.bed.gz
https://www.encodeproject.org/files/ENCFF566KSS/@@download/ENCFF566KSS.bed.gz
https://www.encodeproject.org/files/ENCFF968JTF/@@download/ENCFF968JTF.bed.gz
https://www.encodeproject.org/files/ENCFF322ZNH/@@download/ENCFF322ZNH.bed.gz
https://www.encodeproject.org/files/ENCFF465FWP/@@download/ENCFF465FWP.bed.gz
https://www.encodeproject.org/files/ENCFF371RXZ/@@download/ENCFF371RXZ.bed.gz
https://www.encodeproject.org/files/ENCFF422SQQ/@@download/ENCFF422SQQ.bed.gz
https://www.encodeproject.org/files/ENCFF201INC/@@download/ENCFF201INC.bed.gz
https://www.encodeproject.org/files/ENCFF031SHA/@@download/ENCFF031SHA.bed.gz
https://www.encodeproject.org/files/ENCFF926JFU/@@download/ENCFF926JFU.bed.gz
https://www.encodeproject.org/files/ENCFF466TCI/@@download/ENCFF466TCI.bed.gz
https://www.encodeproject.org/files/ENCFF981YWC/@@download/ENCFF981YWC.bed.gz
https://www.encodeproject.org/files/ENCFF472TGW/@@download/ENCFF472TGW.bed.gz
https://www.encodeproject.org/files/ENCFF741CZJ/@@download/ENCFF741CZJ.bed.gz
https://www.encodeproject.org/files/ENCFF850WOE/@@download/ENCFF850WOE.bed.gz
https://www.encodeproject.org/files/ENCFF327HDP/@@download/ENCFF327HDP.bed.gz
https://www.encodeproject.org/files/ENCFF630USR/@@download/ENCFF630USR.bed.gz
https://www.encodeproject.org/files/ENCFF822VOH/@@download/ENCFF822VOH.bed.gz
https://www.encodeproject.org/files/ENCFF047VNP/@@download/ENCFF047VNP.bed.gz
https://www.encodeproject.org/files/ENCFF960GTK/@@download/ENCFF960GTK.bed.gz
https://www.encodeproject.org/files/ENCFF457SNJ/@@download/ENCFF457SNJ.bed.gz
https://www.encodeproject.org/files/ENCFF208QNH/@@download/ENCFF208QNH.bed.gz
https://www.encodeproject.org/files/ENCFF929JWE/@@download/ENCFF929JWE.bed.gz
https://www.encodeproject.org/files/ENCFF097UAJ/@@download/ENCFF097UAJ.bed.gz
https://www.encodeproject.org/files/ENCFF152RON/@@download/ENCFF152RON.bed.gz
https://www.encodeproject.org/files/ENCFF731WEV/@@download/ENCFF731WEV.bed.gz
https://www.encodeproject.org/files/ENCFF765ZIA/@@download/ENCFF765ZIA.bed.gz
https://www.encodeproject.org/files/ENCFF033GXT/@@download/ENCFF033GXT.bed.gz
https://www.encodeproject.org/files/ENCFF832UZR/@@download/ENCFF832UZR.bed.gz
https://www.encodeproject.org/files/ENCFF799OWA/@@download/ENCFF799OWA.bed.gz
https://www.encodeproject.org/files/ENCFF362PZC/@@download/ENCFF362PZC.bed.gz
https://www.encodeproject.org/files/ENCFF243ZBV/@@download/ENCFF243ZBV.bed.gz
https://www.encodeproject.org/files/ENCFF176BHG/@@download/ENCFF176BHG.bed.gz
https://www.encodeproject.org/files/ENCFF874KUM/@@download/ENCFF874KUM.bed.gz
https://www.encodeproject.org/files/ENCFF165LSE/@@download/ENCFF165LSE.bed.gz
https://www.encodeproject.org/files/ENCFF826HLM/@@download/ENCFF826HLM.bed.gz
https://www.encodeproject.org/files/ENCFF358CVP/@@download/ENCFF358CVP.bed.gz
https://www.encodeproject.org/files/ENCFF251OQX/@@download/ENCFF251OQX.bed.gz
https://www.encodeproject.org/files/ENCFF992VNB/@@download/ENCFF992VNB.bed.gz
https://www.encodeproject.org/files/ENCFF274FLT/@@download/ENCFF274FLT.bed.gz
https://www.encodeproject.org/files/ENCFF238ZFI/@@download/ENCFF238ZFI.bed.gz
https://www.encodeproject.org/files/ENCFF290EPY/@@download/ENCFF290EPY.bed.gz
https://www.encodeproject.org/files/ENCFF216SYK/@@download/ENCFF216SYK.bed.gz
https://www.encodeproject.org/files/ENCFF461MVJ/@@download/ENCFF461MVJ.bed.gz
https://www.encodeproject.org/files/ENCFF376EBF/@@download/ENCFF376EBF.bed.gz
https://www.encodeproject.org/files/ENCFF941HUI/@@download/ENCFF941HUI.bed.gz
https://www.encodeproject.org/files/ENCFF336JBS/@@download/ENCFF336JBS.bed.gz
https://www.encodeproject.org/files/ENCFF701AFB/@@download/ENCFF701AFB.bed.gz
https://www.encodeproject.org/files/ENCFF196JPA/@@download/ENCFF196JPA.bed.gz

https://www.encodeproject.org/files/ENCFF922JEK/@@download/ENCFF922JEK.bed.gz
https://www.encodeproject.org/files/ENCFF699XQF/@@download/ENCFF699XQF.bed.gz
https://www.encodeproject.org/files/ENCFF230WVU/@@download/ENCFF230WVU.bed.gz
https://www.encodeproject.org/files/ENCFF419TZO/@@download/ENCFF419TZO.bed.gz
https://www.encodeproject.org/files/ENCFF227CPA/@@download/ENCFF227CPA.bed.gz
https://www.encodeproject.org/files/ENCFF727DDM/@@download/ENCFF727DDM.bed.gz
https://www.encodeproject.org/files/ENCFF914OII/@@download/ENCFF914OII.bed.gz
https://www.encodeproject.org/files/ENCFF323KSA/@@download/ENCFF323KSA.bed.gz
https://www.encodeproject.org/files/ENCFF449PAL/@@download/ENCFF449PAL.bed.gz
https://www.encodeproject.org/files/ENCFF064JZR/@@download/ENCFF064JZR.bed.gz
https://www.encodeproject.org/files/ENCFF598HGO/@@download/ENCFF598HGO.bed.gz
https://www.encodeproject.org/files/ENCFF772JBK/@@download/ENCFF772JBK.bed.gz
https://www.encodeproject.org/files/ENCFF452SZV/@@download/ENCFF452SZV.bed.gz
https://www.encodeproject.org/files/ENCFF491PPP/@@download/ENCFF491PPP.bed.gz
https://www.encodeproject.org/files/ENCFF230WCV/@@download/ENCFF230WCV.bed.gz
https://www.encodeproject.org/files/ENCFF079ELS/@@download/ENCFF079ELS.bed.gz
https://www.encodeproject.org/files/ENCFF804DUL/@@download/ENCFF804DUL.bed.gz
https://www.encodeproject.org/files/ENCFF524EGE/@@download/ENCFF524EGE.bed.gz
https://www.encodeproject.org/files/ENCFF312NAH/@@download/ENCFF312NAH.bed.gz
https://www.encodeproject.org/files/ENCFF292LZY/@@download/ENCFF292LZY.bed.gz
https://www.encodeproject.org/files/ENCFF476LDM/@@download/ENCFF476LDM.bed.gz
https://www.encodeproject.org/files/ENCFF116PRN/@@download/ENCFF116PRN.bed.gz
https://www.encodeproject.org/files/ENCFF246DEW/@@download/ENCFF246DEW.bed.gz
https://www.encodeproject.org/files/ENCFF567HDQ/@@download/ENCFF567HDQ.bed.gz
https://www.encodeproject.org/files/ENCFF988BMI/@@download/ENCFF988BMI.bed.gz
https://www.encodeproject.org/files/ENCFF001SQK/@@download/ENCFF001SQK.bed.gz
https://www.encodeproject.org/files/ENCFF001WGP/@@download/ENCFF001WGP.bed.gz
https://www.encodeproject.org/files/ENCFF001WGQ/@@download/ENCFF001WGQ.bed.gz
https://www.encodeproject.org/files/ENCFF173MXX/@@download/ENCFF173MXX.bed.gz
https://www.encodeproject.org/files/ENCFF714QOR/@@download/ENCFF714QOR.bed.gz
https://www.encodeproject.org/files/ENCFF819ZIM/@@download/ENCFF819ZIM.bed.gz
https://www.encodeproject.org/files/ENCFF057PEY/@@download/ENCFF057PEY.bed.gz
https://www.encodeproject.org/files/ENCFF892TZU/@@download/ENCFF892TZU.bed.gz
https://www.encodeproject.org/files/ENCFF588QXP/@@download/ENCFF588QXP.bed.gz
https://www.encodeproject.org/files/ENCFF702JBC/@@download/ENCFF702JBC.bed.gz
https://www.encodeproject.org/files/ENCFF901ATC/@@download/ENCFF901ATC.bed.gz
https://www.encodeproject.org/files/ENCFF886GXC/@@download/ENCFF886GXC.bed.gz
https://www.encodeproject.org/files/ENCFF938IDZ/@@download/ENCFF938IDZ.bed.gz
https://www.encodeproject.org/files/ENCFF915YFO/@@download/ENCFF915YFO.bed.gz
https://www.encodeproject.org/files/ENCFF347MBD/@@download/ENCFF347MBD.bed.gz
https://www.encodeproject.org/files/ENCFF249KIJ/@@download/ENCFF249KIJ.bed.gz
https://www.encodeproject.org/files/ENCFF637CAJ/@@download/ENCFF637CAJ.bed.gz
https://www.encodeproject.org/files/ENCFF751PUA/@@download/ENCFF751PUA.bed.gz
https://www.encodeproject.org/files/ENCFF199RXV/@@download/ENCFF199RXV.bed.gz
https://www.encodeproject.org/files/ENCFF908KXP/@@download/ENCFF908KXP.bed.gz
https://www.encodeproject.org/files/ENCFF921FAL/@@download/ENCFF921FAL.bed.gz
https://www.encodeproject.org/files/ENCFF783TQZ/@@download/ENCFF783TQZ.bed.gz
https://www.encodeproject.org/files/ENCFF690IVT/@@download/ENCFF690IVT.bed.gz
https://www.encodeproject.org/files/ENCFF829UTZ/@@download/ENCFF829UTZ.bed.gz
https://www.encodeproject.org/files/ENCFF403PJS/@@download/ENCFF403PJS.bed.gz
https://www.encodeproject.org/files/ENCFF355LDD/@@download/ENCFF355LDD.bed.gz
https://www.encodeproject.org/files/ENCFF654IWG/@@download/ENCFF654IWG.bed.gz
https://www.encodeproject.org/files/ENCFF238LUI/@@download/ENCFF238LUI.bed.gz

https://www.encodeproject.org/files/ENCFF957KRB/@@download/ENCFF957KRB.bed.gz
https://www.encodeproject.org/files/ENCFF109ERC/@@download/ENCFF109ERC.bed.gz
https://www.encodeproject.org/files/ENCFF817OHQ/@@download/ENCFF817OHQ.bed.gz
https://www.encodeproject.org/files/ENCFF640MFC/@@download/ENCFF640MFC.bed.gz
https://www.encodeproject.org/files/ENCFF106PMZ/@@download/ENCFF106PMZ.bed.gz
https://www.encodeproject.org/files/ENCFF619DCX/@@download/ENCFF619DCX.bed.gz
https://www.encodeproject.org/files/ENCFF382MCS/@@download/ENCFF382MCS.bed.gz
https://www.encodeproject.org/files/ENCFF532OGD/@@download/ENCFF532OGD.bed.gz
https://www.encodeproject.org/files/ENCFF833JBU/@@download/ENCFF833JBU.bed.gz
https://www.encodeproject.org/files/ENCFF747BVJ/@@download/ENCFF747BVJ.bed.gz
https://www.encodeproject.org/files/ENCFF031EZZ/@@download/ENCFF031EZZ.bed.gz
https://www.encodeproject.org/files/ENCFF581CUG/@@download/ENCFF581CUG.bed.gz
https://www.encodeproject.org/files/ENCFF825WDW/@@download/ENCFF825WDW.bed.gz
https://www.encodeproject.org/files/ENCFF812GJU/@@download/ENCFF812GJU.bed.gz
https://www.encodeproject.org/files/ENCFF515FKC/@@download/ENCFF515FKC.bed.gz
https://www.encodeproject.org/files/ENCFF132JBP/@@download/ENCFF132JBP.bed.gz
https://www.encodeproject.org/files/ENCFF711ASQ/@@download/ENCFF711ASQ.bed.gz
https://www.encodeproject.org/files/ENCFF896ZUQ/@@download/ENCFF896ZUQ.bed.gz
https://www.encodeproject.org/files/ENCFF659BCP/@@download/ENCFF659BCP.bed.gz
https://www.encodeproject.org/files/ENCFF372CNG/@@download/ENCFF372CNG.bed.gz
https://www.encodeproject.org/files/ENCFF158UAN/@@download/ENCFF158UAN.bed.gz
https://www.encodeproject.org/files/ENCFF028NJF/@@download/ENCFF028NJF.bed.gz
https://www.encodeproject.org/files/ENCFF603EXE/@@download/ENCFF603EXE.bed.gz
https://www.encodeproject.org/files/ENCFF003GCY/@@download/ENCFF003GCY.bed.gz
https://www.encodeproject.org/files/ENCFF602BHS/@@download/ENCFF602BHS.bed.gz
https://www.encodeproject.org/files/ENCFF232BIG/@@download/ENCFF232BIG.bed.gz
https://www.encodeproject.org/files/ENCFF944DGI/@@download/ENCFF944DGI.bed.gz
https://www.encodeproject.org/files/ENCFF779CFZ/@@download/ENCFF779CFZ.bed.gz
https://www.encodeproject.org/files/ENCFF134ZVJ/@@download/ENCFF134ZVJ.bed.gz
https://www.encodeproject.org/files/ENCFF028CVN/@@download/ENCFF028CVN.bed.gz
https://www.encodeproject.org/files/ENCFF523TKA/@@download/ENCFF523TKA.bed.gz
https://www.encodeproject.org/files/ENCFF611KWJ/@@download/ENCFF611KWJ.bed.gz
https://www.encodeproject.org/files/ENCFF016XGY/@@download/ENCFF016XGY.bed.gz
https://www.encodeproject.org/files/ENCFF828JRB/@@download/ENCFF828JRB.bed.gz
https://www.encodeproject.org/files/ENCFF495XMA/@@download/ENCFF495XMA.bed.gz
https://www.encodeproject.org/files/ENCFF901XRC/@@download/ENCFF901XRC.bed.gz
https://www.encodeproject.org/files/ENCFF854EJY/@@download/ENCFF854EJY.bed.gz
https://www.encodeproject.org/files/ENCFF727NEX/@@download/ENCFF727NEX.bed.gz
https://www.encodeproject.org/files/ENCFF371GGV/@@download/ENCFF371GGV.bed.gz
https://www.encodeproject.org/files/ENCFF264UIE/@@download/ENCFF264UIE.bed.gz
https://www.encodeproject.org/files/ENCFF995DGS/@@download/ENCFF995DGS.bed.gz
https://www.encodeproject.org/files/ENCFF209TED/@@download/ENCFF209TED.bed.gz
https://www.encodeproject.org/files/ENCFF382WUE/@@download/ENCFF382WUE.bed.gz
https://www.encodeproject.org/files/ENCFF083GBY/@@download/ENCFF083GBY.bed.gz
https://www.encodeproject.org/files/ENCFF682RBA/@@download/ENCFF682RBA.bed.gz
https://www.encodeproject.org/files/ENCFF230IMJ/@@download/ENCFF230IMJ.bed.gz
https://www.encodeproject.org/files/ENCFF199IMR/@@download/ENCFF199IMR.bed.gz
https://www.encodeproject.org/files/ENCFF334VOK/@@download/ENCFF334VOK.bed.gz
https://www.encodeproject.org/files/ENCFF171PUD/@@download/ENCFF171PUD.bed.gz
https://www.encodeproject.org/files/ENCFF852PNR/@@download/ENCFF852PNR.bed.gz
https://www.encodeproject.org/files/ENCFF475YRW/@@download/ENCFF475YRW.bed.gz
https://www.encodeproject.org/files/ENCFF307VFD/@@download/ENCFF307VFD.bed.gz
https://www.encodeproject.org/files/ENCFF786QZY/@@download/ENCFF786QZY.bed.gz

Supplementary Table 1

https://www.encodeproject.org/files/ENCFF489OIN/@@download/ENCFF489OIN.bed.gz
https://www.encodeproject.org/files/ENCFF613TMO/@@download/ENCFF613TMO.bed.gz
https://www.encodeproject.org/files/ENCFF434BQJ/@@download/ENCFF434BQJ.bed.gz
https://www.encodeproject.org/files/ENCFF681SCC/@@download/ENCFF681SCC.bed.gz
https://www.encodeproject.org/files/ENCFF372NZP/@@download/ENCFF372NZP.bed.gz
https://www.encodeproject.org/files/ENCFF327DJR/@@download/ENCFF327DJR.bed.gz
https://www.encodeproject.org/files/ENCFF836HJU/@@download/ENCFF836HJU.bed.gz
https://www.encodeproject.org/files/ENCFF074KEY/@@download/ENCFF074KEY.bed.gz
https://www.encodeproject.org/files/ENCFF320XBU/@@download/ENCFF320XBU.bed.gz
https://www.encodeproject.org/files/ENCFF349CIP/@@download/ENCFF349CIP.bed.gz
https://www.encodeproject.org/files/ENCFF991WDM/@@download/ENCFF991WDM.bed.gz
https://www.encodeproject.org/files/ENCFF810WAZ/@@download/ENCFF810WAZ.bed.gz
https://www.encodeproject.org/files/ENCFF187FTS/@@download/ENCFF187FTS.bed.gz
https://www.encodeproject.org/files/ENCFF321NTP/@@download/ENCFF321NTP.bed.gz
https://www.encodeproject.org/files/ENCFF315TWS/@@download/ENCFF315TWS.bed.gz
https://www.encodeproject.org/files/ENCFF009YJE/@@download/ENCFF009YJE.bed.gz
https://www.encodeproject.org/files/ENCFF933ABR/@@download/ENCFF933ABR.bed.gz
https://www.encodeproject.org/files/ENCFF613IXQ/@@download/ENCFF613IXQ.bed.gz
https://www.encodeproject.org/files/ENCFF858JQG/@@download/ENCFF858JQG.bed.gz
https://www.encodeproject.org/files/ENCFF157IYR/@@download/ENCFF157IYR.bed.gz
https://www.encodeproject.org/files/ENCFF916VOC/@@download/ENCFF916VOC.bed.gz
https://www.encodeproject.org/files/ENCFF586UYY/@@download/ENCFF586UYY.bed.gz
https://www.encodeproject.org/files/ENCFF134ZJK/@@download/ENCFF134ZJK.bed.gz
https://www.encodeproject.org/files/ENCFF465GKU/@@download/ENCFF465GKU.bed.gz
https://www.encodeproject.org/files/ENCFF016DSD/@@download/ENCFF016DSD.bed.gz
https://www.encodeproject.org/files/ENCFF994DZN/@@download/ENCFF994DZN.bed.gz
https://www.encodeproject.org/files/ENCFF304MPW/@@download/ENCFF304MPW.bed.gz
https://www.encodeproject.org/files/ENCFF022UVJ/@@download/ENCFF022UVJ.bed.gz
https://www.encodeproject.org/files/ENCFF836ETB/@@download/ENCFF836ETB.bed.gz
https://www.encodeproject.org/files/ENCFF682ALU/@@download/ENCFF682ALU.bed.gz
https://www.encodeproject.org/files/ENCFF979SJD/@@download/ENCFF979SJD.bed.gz
https://www.encodeproject.org/files/ENCFF689FDS/@@download/ENCFF689FDS.bed.gz
https://www.encodeproject.org/files/ENCFF963VXI/@@download/ENCFF963VXI.bed.gz
https://www.encodeproject.org/files/ENCFF785AIA/@@download/ENCFF785AIA.bed.gz
https://www.encodeproject.org/files/ENCFF962TZR/@@download/ENCFF962TZR.bed.gz
https://www.encodeproject.org/files/ENCFF553KUB/@@download/ENCFF553KUB.bed.gz
https://www.encodeproject.org/files/ENCFF971SYQ/@@download/ENCFF971SYQ.bed.gz
https://www.encodeproject.org/files/ENCFF281DKC/@@download/ENCFF281DKC.bed.gz
https://www.encodeproject.org/files/ENCFF543NMS/@@download/ENCFF543NMS.bed.gz
https://www.encodeproject.org/files/ENCFF470XXM/@@download/ENCFF470XXM.bed.gz
https://www.encodeproject.org/files/ENCFF609KNI/@@download/ENCFF609KNI.bed.gz
https://www.encodeproject.org/files/ENCFF382AEJ/@@download/ENCFF382AEJ.bed.gz
https://www.encodeproject.org/files/ENCFF814TBU/@@download/ENCFF814TBU.bed.gz
https://www.encodeproject.org/files/ENCFF327JLK/@@download/ENCFF327JLK.bed.gz
https://www.encodeproject.org/files/ENCFF696DDE/@@download/ENCFF696DDE.bed.gz
https://www.encodeproject.org/files/ENCFF058UNN/@@download/ENCFF058UNN.bed.gz
https://www.encodeproject.org/files/ENCFF572RTJ/@@download/ENCFF572RTJ.bed.gz
https://www.encodeproject.org/files/ENCFF173JRC/@@download/ENCFF173JRC.bed.gz
https://www.encodeproject.org/files/ENCFF186QCQ/@@download/ENCFF186QCQ.bed.gz
https://www.encodeproject.org/files/ENCFF409FEK/@@download/ENCFF409FEK.bed.gz
https://www.encodeproject.org/files/ENCFF197BPE/@@download/ENCFF197BPE.bed.gz
https://www.encodeproject.org/files/ENCFF018TWY/@@download/ENCFF018TWY.bed.gz
https://www.encodeproject.org/files/ENCFF012QTD/@@download/ENCFF012QTD.bed.gz

https://www.encodeproject.org/files/ENCFF518XTM/@@download/ENCFF518XTM.bed.gz
https://www.encodeproject.org/files/ENCFF502RSX/@@download/ENCFF502RSX.bed.gz
https://www.encodeproject.org/files/ENCFF446CFP/@@download/ENCFF446CFP.bed.gz
https://www.encodeproject.org/files/ENCFF510VFD/@@download/ENCFF510VFD.bed.gz
https://www.encodeproject.org/files/ENCFF010WLQ/@@download/ENCFF010WLQ.bed.gz
https://www.encodeproject.org/files/ENCFF352EVQ/@@download/ENCFF352EVQ.bed.gz
https://www.encodeproject.org/files/ENCFF714RWU/@@download/ENCFF714RWU.bed.gz
https://www.encodeproject.org/files/ENCFF208EAG/@@download/ENCFF208EAG.bed.gz
https://www.encodeproject.org/files/ENCFF519BRW/@@download/ENCFF519BRW.bed.gz
https://www.encodeproject.org/files/ENCFF666AHJ/@@download/ENCFF666AHJ.bed.gz
https://www.encodeproject.org/files/ENCFF618BFP/@@download/ENCFF618BFP.bed.gz
https://www.encodeproject.org/files/ENCFF929VDK/@@download/ENCFF929VDK.bed.gz
https://www.encodeproject.org/files/ENCFF400IOO/@@download/ENCFF400IOO.bed.gz
https://www.encodeproject.org/files/ENCFF686ZXP/@@download/ENCFF686ZXP.bed.gz
https://www.encodeproject.org/files/ENCFF289AWO/@@download/ENCFF289AWO.bed.gz
https://www.encodeproject.org/files/ENCFF936IDJ/@@download/ENCFF936IDJ.bed.gz
https://www.encodeproject.org/files/ENCFF748AYU/@@download/ENCFF748AYU.bed.gz
https://www.encodeproject.org/files/ENCFF880KWE/@@download/ENCFF880KWE.bed.gz
https://www.encodeproject.org/files/ENCFF671GMS/@@download/ENCFF671GMS.bed.gz
https://www.encodeproject.org/files/ENCFF933TIH/@@download/ENCFF933TIH.bed.gz
https://www.encodeproject.org/files/ENCFF774PWU/@@download/ENCFF774PWU.bed.gz
https://www.encodeproject.org/files/ENCFF877USZ/@@download/ENCFF877USZ.bed.gz
https://www.encodeproject.org/files/ENCFF694LGV/@@download/ENCFF694LGV.bed.gz
https://www.encodeproject.org/files/ENCFF516KID/@@download/ENCFF516KID.bed.gz
https://www.encodeproject.org/files/ENCFF488ZLH/@@download/ENCFF488ZLH.bed.gz
https://www.encodeproject.org/files/ENCFF761TYQ/@@download/ENCFF761TYQ.bed.gz
https://www.encodeproject.org/files/ENCFF167STH/@@download/ENCFF167STH.bed.gz
https://www.encodeproject.org/files/ENCFF761ZIK/@@download/ENCFF761ZIK.bed.gz
https://www.encodeproject.org/files/ENCFF413NZU/@@download/ENCFF413NZU.bed.gz
https://www.encodeproject.org/files/ENCFF248VSR/@@download/ENCFF248VSR.bed.gz
https://www.encodeproject.org/files/ENCFF347SKO/@@download/ENCFF347SKO.bed.gz
https://www.encodeproject.org/files/ENCFF998DYY/@@download/ENCFF998DYY.bed.gz
https://www.encodeproject.org/files/ENCFF539ZGV/@@download/ENCFF539ZGV.bed.gz
https://www.encodeproject.org/files/ENCFF615KNN/@@download/ENCFF615KNN.bed.gz
https://www.encodeproject.org/files/ENCFF238ATT/@@download/ENCFF238ATT.bed.gz
https://www.encodeproject.org/files/ENCFF719FAT/@@download/ENCFF719FAT.bed.gz
https://www.encodeproject.org/files/ENCFF833JFM/@@download/ENCFF833JFM.bed.gz
https://www.encodeproject.org/files/ENCFF050DXR/@@download/ENCFF050DXR.bed.gz
https://www.encodeproject.org/files/ENCFF945HWH/@@download/ENCFF945HWH.bed.gz
https://www.encodeproject.org/files/ENCFF817YEL/@@download/ENCFF817YEL.bed.gz
https://www.encodeproject.org/files/ENCFF811CIQ/@@download/ENCFF811CIQ.bed.gz
https://www.encodeproject.org/files/ENCFF240SMG/@@download/ENCFF240SMG.bed.gz
https://www.encodeproject.org/files/ENCFF855BFG/@@download/ENCFF855BFG.bed.gz
https://www.encodeproject.org/files/ENCFF207IYJ/@@download/ENCFF207IYJ.bed.gz
https://www.encodeproject.org/files/ENCFF415JJI/@@download/ENCFF415JJI.bed.gz
https://www.encodeproject.org/files/ENCFF266QFC/@@download/ENCFF266QFC.bed.gz
https://www.encodeproject.org/files/ENCFF755QDM/@@download/ENCFF755QDM.bed.gz
https://www.encodeproject.org/files/ENCFF128TOW/@@download/ENCFF128TOW.bed.gz
https://www.encodeproject.org/files/ENCFF929FIK/@@download/ENCFF929FIK.bed.gz
https://www.encodeproject.org/files/ENCFF986ZJB/@@download/ENCFF986ZJB.bed.gz
https://www.encodeproject.org/files/ENCFF182CAL/@@download/ENCFF182CAL.bed.gz
https://www.encodeproject.org/files/ENCFF910QCI/@@download/ENCFF910QCI.bed.gz
https://www.encodeproject.org/files/ENCFF296XIB/@@download/ENCFF296XIB.bed.gz

Supplementary Table 1

https://www.encodeproject.org/files/ENCFF338RTX/@@download/ENCFF338RTX.bed.gz
https://www.encodeproject.org/files/ENCFF040UAE/@@download/ENCFF040UAE.bed.gz
https://www.encodeproject.org/files/ENCFF251NTZ/@@download/ENCFF251NTZ.bed.gz
https://www.encodeproject.org/files/ENCFF898PPG/@@download/ENCFF898PPG.bed.gz
https://www.encodeproject.org/files/ENCFF408KGV/@@download/ENCFF408KGV.bed.gz
https://www.encodeproject.org/files/ENCFF663PTZ/@@download/ENCFF663PTZ.bed.gz
https://www.encodeproject.org/files/ENCFF568IPS/@@download/ENCFF568IPS.bed.gz
https://www.encodeproject.org/files/ENCFF693LCL/@@download/ENCFF693LCL.bed.gz
https://www.encodeproject.org/files/ENCFF530CGM/@@download/ENCFF530CGM.bed.gz
https://www.encodeproject.org/files/ENCFF205BHS/@@download/ENCFF205BHS.bed.gz
https://www.encodeproject.org/files/ENCFF820TAI/@@download/ENCFF820TAI.bed.gz
https://www.encodeproject.org/files/ENCFF464BPU/@@download/ENCFF464BPU.bed.gz
https://www.encodeproject.org/files/ENCFF585TGK/@@download/ENCFF585TGK.bed.gz
https://www.encodeproject.org/files/ENCFF676GRC/@@download/ENCFF676GRC.bed.gz
https://www.encodeproject.org/files/ENCFF986LLS/@@download/ENCFF986LLS.bed.gz
https://www.encodeproject.org/files/ENCFF180TWQ/@@download/ENCFF180TWQ.bed.gz
https://www.encodeproject.org/files/ENCFF425FYS/@@download/ENCFF425FYS.bed.gz
https://www.encodeproject.org/files/ENCFF375UBL/@@download/ENCFF375UBL.bed.gz
https://www.encodeproject.org/files/ENCFF037EEA/@@download/ENCFF037EEA.bed.gz
https://www.encodeproject.org/files/ENCFF089OHV/@@download/ENCFF089OHV.bed.gz
https://www.encodeproject.org/files/ENCFF794IVB/@@download/ENCFF794IVB.bed.gz
https://www.encodeproject.org/files/ENCFF483JXP/@@download/ENCFF483JXP.bed.gz
https://www.encodeproject.org/files/ENCFF539JKS/@@download/ENCFF539JKS.bed.gz
https://www.encodeproject.org/files/ENCFF528QQS/@@download/ENCFF528QQS.bed.gz
https://www.encodeproject.org/files/ENCFF203JLT/@@download/ENCFF203JLT.bed.gz
https://www.encodeproject.org/files/ENCFF194RWJ/@@download/ENCFF194RWJ.bed.gz
https://www.encodeproject.org/files/ENCFF276FKY/@@download/ENCFF276FKY.bed.gz
https://www.encodeproject.org/files/ENCFF807DWK/@@download/ENCFF807DWK.bed.gz
https://www.encodeproject.org/files/ENCFF316VYS/@@download/ENCFF316VYS.bed.gz
https://www.encodeproject.org/files/ENCFF305IAL/@@download/ENCFF305IAL.bed.gz
https://www.encodeproject.org/files/ENCFF054IJX/@@download/ENCFF054IJX.bed.gz
https://www.encodeproject.org/files/ENCFF286XIL/@@download/ENCFF286XIL.bed.gz
https://www.encodeproject.org/files/ENCFF528GDM/@@download/ENCFF528GDM.bed.gz
https://www.encodeproject.org/files/ENCFF437AEG/@@download/ENCFF437AEG.bed.gz
https://www.encodeproject.org/files/ENCFF418IIT/@@download/ENCFF418IIT.bed.gz
https://www.encodeproject.org/files/ENCFF135UVJ/@@download/ENCFF135UVJ.bed.gz
https://www.encodeproject.org/files/ENCFF770SAC/@@download/ENCFF770SAC.bed.gz
https://www.encodeproject.org/files/ENCFF258KAT/@@download/ENCFF258KAT.bed.gz
https://www.encodeproject.org/files/ENCFF082PWS/@@download/ENCFF082PWS.bed.gz
https://www.encodeproject.org/files/ENCFF001WAY/@@download/ENCFF001WAY.bed.gz
https://www.encodeproject.org/files/ENCFF001WMU/@@download/ENCFF001WMU.bed.gz
https://www.encodeproject.org/files/ENCFF001WMT/@@download/ENCFF001WMT.bed.gz
https://www.encodeproject.org/files/ENCFF216SNT/@@download/ENCFF216SNT.bed.gz
https://www.encodeproject.org/files/ENCFF352KQN/@@download/ENCFF352KQN.bed.gz
https://www.encodeproject.org/files/ENCFF962IGI/@@download/ENCFF962IGI.bed.gz
https://www.encodeproject.org/files/ENCFF223UWR/@@download/ENCFF223UWR.bed.gz
https://www.encodeproject.org/files/ENCFF200WGF/@@download/ENCFF200WGF.bed.gz
https://www.encodeproject.org/files/ENCFF710XCN/@@download/ENCFF710XCN.bed.gz
https://www.encodeproject.org/files/ENCFF547OAH/@@download/ENCFF547OAH.bed.gz
https://www.encodeproject.org/files/ENCFF422JUT/@@download/ENCFF422JUT.bed.gz
https://www.encodeproject.org/files/ENCFF666HLW/@@download/ENCFF666HLW.bed.gz
https://www.encodeproject.org/files/ENCFF103UPI/@@download/ENCFF103UPI.bed.gz
https://www.encodeproject.org/files/ENCFF678IKQ/@@download/ENCFF678IKQ.bed.gz

Supplementary Table 1

https://www.encodeproject.org/files/ENCFF251UGS/@@download/ENCFF251UGS.bed.gz
https://www.encodeproject.org/files/ENCFF065PFK/@@download/ENCFF065PFK.bed.gz
https://www.encodeproject.org/files/ENCFF858CNH/@@download/ENCFF858CNH.bed.gz
https://www.encodeproject.org/files/ENCFF001SQY/@@download/ENCFF001SQY.bed.gz
https://www.encodeproject.org/files/ENCFF001WAA/@@download/ENCFF001WAA.bed.gz
https://www.encodeproject.org/files/ENCFF001WJI/@@download/ENCFF001WJI.bed.gz
https://www.encodeproject.org/files/ENCFF001WJH/@@download/ENCFF001WJH.bed.gz
https://www.encodeproject.org/files/ENCFF086HEP/@@download/ENCFF086HEP.bed.gz
https://www.encodeproject.org/files/ENCFF102DHF/@@download/ENCFF102DHF.bed.gz
https://www.encodeproject.org/files/ENCFF063MTX/@@download/ENCFF063MTX.bed.gz
https://www.encodeproject.org/files/ENCFF741BMK/@@download/ENCFF741BMK.bed.gz
https://www.encodeproject.org/files/ENCFF318TAF/@@download/ENCFF318TAF.bed.gz
https://www.encodeproject.org/files/ENCFF792BQE/@@download/ENCFF792BQE.bed.gz
https://www.encodeproject.org/files/ENCFF700NCC/@@download/ENCFF700NCC.bed.gz
https://www.encodeproject.org/files/ENCFF920DLC/@@download/ENCFF920DLC.bed.gz
https://www.encodeproject.org/files/ENCFF229KSL/@@download/ENCFF229KSL.bed.gz
https://www.encodeproject.org/files/ENCFF611CTG/@@download/ENCFF611CTG.bed.gz
https://www.encodeproject.org/files/ENCFF094EMV/@@download/ENCFF094EMV.bed.gz
https://www.encodeproject.org/files/ENCFF617SLR/@@download/ENCFF617SLR.bed.gz
https://www.encodeproject.org/files/ENCFF900LAT/@@download/ENCFF900LAT.bed.gz
https://www.encodeproject.org/files/ENCFF831AAQ/@@download/ENCFF831AAQ.bed.gz
https://www.encodeproject.org/files/ENCFF516FXF/@@download/ENCFF516FXF.bed.gz
https://www.encodeproject.org/files/ENCFF104JWY/@@download/ENCFF104JWY.bed.gz
https://www.encodeproject.org/files/ENCFF655VLV/@@download/ENCFF655VLV.bed.gz
https://www.encodeproject.org/files/ENCFF777SVA/@@download/ENCFF777SVA.bed.gz
https://www.encodeproject.org/files/ENCFF448ZKW/@@download/ENCFF448ZKW.bed.gz
https://www.encodeproject.org/files/ENCFF650YFS/@@download/ENCFF650YFS.bed.gz
https://www.encodeproject.org/files/ENCFF166HST/@@download/ENCFF166HST.bed.gz
https://www.encodeproject.org/files/ENCFF797TTK/@@download/ENCFF797TTK.bed.gz
https://www.encodeproject.org/files/ENCFF203FLV/@@download/ENCFF203FLV.bed.gz
https://www.encodeproject.org/files/ENCFF054UBW/@@download/ENCFF054UBW.bed.gz
https://www.encodeproject.org/files/ENCFF572YUX/@@download/ENCFF572YUX.bed.gz
https://www.encodeproject.org/files/ENCFF781ZDV/@@download/ENCFF781ZDV.bed.gz
https://www.encodeproject.org/files/ENCFF062LJL/@@download/ENCFF062LJL.bed.gz
https://www.encodeproject.org/files/ENCFF171JLD/@@download/ENCFF171JLD.bed.gz
https://www.encodeproject.org/files/ENCFF066LTB/@@download/ENCFF066LTB.bed.gz
https://www.encodeproject.org/files/ENCFF373OQQ/@@download/ENCFF373OQQ.bed.gz
https://www.encodeproject.org/files/ENCFF365MBB/@@download/ENCFF365MBB.bed.gz
https://www.encodeproject.org/files/ENCFF454ASD/@@download/ENCFF454ASD.bed.gz
https://www.encodeproject.org/files/ENCFF831RQP/@@download/ENCFF831RQP.bed.gz
https://www.encodeproject.org/files/ENCFF191JLB/@@download/ENCFF191JLB.bed.gz
https://www.encodeproject.org/files/ENCFF975DXH/@@download/ENCFF975DXH.bed.gz
https://www.encodeproject.org/files/ENCFF241TEF/@@download/ENCFF241TEF.bed.gz
https://www.encodeproject.org/files/ENCFF340HXV/@@download/ENCFF340HXV.bed.gz
https://www.encodeproject.org/files/ENCFF274QOY/@@download/ENCFF274QOY.bed.gz
https://www.encodeproject.org/files/ENCFF334ENU/@@download/ENCFF334ENU.bed.gz
https://www.encodeproject.org/files/ENCFF332PPZ/@@download/ENCFF332PPZ.bed.gz
https://www.encodeproject.org/files/ENCFF411ADY/@@download/ENCFF411ADY.bed.gz
https://www.encodeproject.org/files/ENCFF248MCU/@@download/ENCFF248MCU.bed.gz
https://www.encodeproject.org/files/ENCFF630LEF/@@download/ENCFF630LEF.bed.gz
https://www.encodeproject.org/files/ENCFF130PYY/@@download/ENCFF130PYY.bed.gz
https://www.encodeproject.org/files/ENCFF970QZI/@@download/ENCFF970QZI.bed.gz
https://www.encodeproject.org/files/ENCFF470QNA/@@download/ENCFF470QNA.bed.gz

Supplementary Table 1

https://www.encodeproject.org/files/ENCFF047EML/@@download/ENCFF047EML.bed.gz
https://www.encodeproject.org/files/ENCFF911AZF/@@download/ENCFF911AZF.bed.gz
https://www.encodeproject.org/files/ENCFF500XRA/@@download/ENCFF500XRA.bed.gz
https://www.encodeproject.org/files/ENCFF405FRU/@@download/ENCFF405FRU.bed.gz
https://www.encodeproject.org/files/ENCFF157KGS/@@download/ENCFF157KGS.bed.gz
https://www.encodeproject.org/files/ENCFF857LHK/@@download/ENCFF857LHK.bed.gz
https://www.encodeproject.org/files/ENCFF678OAV/@@download/ENCFF678OAV.bed.gz
https://www.encodeproject.org/files/ENCFF420ADF/@@download/ENCFF420ADF.bed.gz
https://www.encodeproject.org/files/ENCFF422BPW/@@download/ENCFF422BPW.bed.gz
https://www.encodeproject.org/files/ENCFF616EQW/@@download/ENCFF616EQW.bed.gz
https://www.encodeproject.org/files/ENCFF108XQG/@@download/ENCFF108XQG.bed.gz
https://www.encodeproject.org/files/ENCFF233ORQ/@@download/ENCFF233ORQ.bed.gz
https://www.encodeproject.org/files/ENCFF488DIV/@@download/ENCFF488DIV.bed.gz
https://www.encodeproject.org/files/ENCFF796YHU/@@download/ENCFF796YHU.bed.gz
https://www.encodeproject.org/files/ENCFF225YGW/@@download/ENCFF225YGW.bed.gz
https://www.encodeproject.org/files/ENCFF044SPL/@@download/ENCFF044SPL.bed.gz
https://www.encodeproject.org/files/ENCFF976NRJ/@@download/ENCFF976NRJ.bed.gz
https://www.encodeproject.org/files/ENCFF412ATV/@@download/ENCFF412ATV.bed.gz
https://www.encodeproject.org/files/ENCFF599BMR/@@download/ENCFF599BMR.bed.gz
https://www.encodeproject.org/files/ENCFF514SRC/@@download/ENCFF514SRC.bed.gz
https://www.encodeproject.org/files/ENCFF133ICX/@@download/ENCFF133ICX.bed.gz
https://www.encodeproject.org/files/ENCFF927SPG/@@download/ENCFF927SPG.bed.gz
https://www.encodeproject.org/files/ENCFF298ZZV/@@download/ENCFF298ZZV.bed.gz
https://www.encodeproject.org/files/ENCFF001SRX/@@download/ENCFF001SRX.bed.gz
https://www.encodeproject.org/files/ENCFF001WBU/@@download/ENCFF001WBU.bed.gz
https://www.encodeproject.org/files/ENCFF001WQS/@@download/ENCFF001WQS.bed.gz
https://www.encodeproject.org/files/ENCFF001WQR/@@download/ENCFF001WQR.bed.gz
https://www.encodeproject.org/files/ENCFF558EUY/@@download/ENCFF558EUY.bed.gz
https://www.encodeproject.org/files/ENCFF619FYY/@@download/ENCFF619FYY.bed.gz
https://www.encodeproject.org/files/ENCFF399UZY/@@download/ENCFF399UZY.bed.gz
https://www.encodeproject.org/files/ENCFF803JHO/@@download/ENCFF803JHO.bed.gz
https://www.encodeproject.org/files/ENCFF293TIJ/@@download/ENCFF293TIJ.bed.gz
https://www.encodeproject.org/files/ENCFF204SXQ/@@download/ENCFF204SXQ.bed.gz
https://www.encodeproject.org/files/ENCFF789WQN/@@download/ENCFF789WQN.bed.gz
https://www.encodeproject.org/files/ENCFF538VZH/@@download/ENCFF538VZH.bed.gz
https://www.encodeproject.org/files/ENCFF903SKN/@@download/ENCFF903SKN.bed.gz
https://www.encodeproject.org/files/ENCFF716RGP/@@download/ENCFF716RGP.bed.gz
https://www.encodeproject.org/files/ENCFF966MFW/@@download/ENCFF966MFW.bed.gz
https://www.encodeproject.org/files/ENCFF655XBN/@@download/ENCFF655XBN.bed.gz
https://www.encodeproject.org/files/ENCFF993GBL/@@download/ENCFF993GBL.bed.gz
https://www.encodeproject.org/files/ENCFF512IML/@@download/ENCFF512IML.bed.gz
https://www.encodeproject.org/files/ENCFF312YPK/@@download/ENCFF312YPK.bed.gz
https://www.encodeproject.org/files/ENCFF782ITT/@@download/ENCFF782ITT.bed.gz
https://www.encodeproject.org/files/ENCFF433VTN/@@download/ENCFF433VTN.bed.gz
https://www.encodeproject.org/files/ENCFF797QID/@@download/ENCFF797QID.bed.gz
https://www.encodeproject.org/files/ENCFF753IAY/@@download/ENCFF753IAY.bed.gz
https://www.encodeproject.org/files/ENCFF516TPQ/@@download/ENCFF516TPQ.bed.gz
https://www.encodeproject.org/files/ENCFF561QZT/@@download/ENCFF561QZT.bed.gz
https://www.encodeproject.org/files/ENCFF699QSY/@@download/ENCFF699QSY.bed.gz
https://www.encodeproject.org/files/ENCFF854JUR/@@download/ENCFF854JUR.bed.gz
https://www.encodeproject.org/files/ENCFF163TAJ/@@download/ENCFF163TAJ.bed.gz
https://www.encodeproject.org/files/ENCFF688LNA/@@download/ENCFF688LNA.bed.gz
https://www.encodeproject.org/files/ENCFF689DDJ/@@download/ENCFF689DDJ.bed.gz

Supplementary Table 1

https://www.encodeproject.org/files/ENCFF269TPT/@@download/ENCFF269TPT.bed.gz
https://www.encodeproject.org/files/ENCFF601PAY/@@download/ENCFF601PAY.bed.gz
https://www.encodeproject.org/files/ENCFF745OVI/@@download/ENCFF745OVI.bed.gz
https://www.encodeproject.org/files/ENCFF131LUK/@@download/ENCFF131LUK.bed.gz
https://www.encodeproject.org/files/ENCFF831JPV/@@download/ENCFF831JPV.bed.gz
https://www.encodeproject.org/files/ENCFF755CWG/@@download/ENCFF755CWG.bed.gz
https://www.encodeproject.org/files/ENCFF911WDJ/@@download/ENCFF911WDJ.bed.gz
https://www.encodeproject.org/files/ENCFF674AGU/@@download/ENCFF674AGU.bed.gz
https://www.encodeproject.org/files/ENCFF191MBC/@@download/ENCFF191MBC.bed.gz
https://www.encodeproject.org/files/ENCFF355JBS/@@download/ENCFF355JBS.bed.gz
https://www.encodeproject.org/files/ENCFF757IKL/@@download/ENCFF757IKL.bed.gz
https://www.encodeproject.org/files/ENCFF194JND/@@download/ENCFF194JND.bed.gz
https://www.encodeproject.org/files/ENCFF374WTZ/@@download/ENCFF374WTZ.bed.gz
https://www.encodeproject.org/files/ENCFF079FQJ/@@download/ENCFF079FQJ.bed.gz
https://www.encodeproject.org/files/ENCFF160NEE/@@download/ENCFF160NEE.bed.gz
https://www.encodeproject.org/files/ENCFF585XKV/@@download/ENCFF585XKV.bed.gz
https://www.encodeproject.org/files/ENCFF056FRC/@@download/ENCFF056FRC.bed.gz
https://www.encodeproject.org/files/ENCFF817MGC/@@download/ENCFF817MGC.bed.gz
https://www.encodeproject.org/files/ENCFF594XCR/@@download/ENCFF594XCR.bed.gz
https://www.encodeproject.org/files/ENCFF043UST/@@download/ENCFF043UST.bed.gz
https://www.encodeproject.org/files/ENCFF439ZRL/@@download/ENCFF439ZRL.bed.gz
https://www.encodeproject.org/files/ENCFF827RCC/@@download/ENCFF827RCC.bed.gz
https://www.encodeproject.org/files/ENCFF434IPQ/@@download/ENCFF434IPQ.bed.gz
https://www.encodeproject.org/files/ENCFF800AAI/@@download/ENCFF800AAI.bed.gz
https://www.encodeproject.org/files/ENCFF882DPD/@@download/ENCFF882DPD.bed.gz
https://www.encodeproject.org/files/ENCFF227OWL/@@download/ENCFF227OWL.bed.gz
https://www.encodeproject.org/files/ENCFF845VOI/@@download/ENCFF845VOI.bed.gz
https://www.encodeproject.org/files/ENCFF743APB/@@download/ENCFF743APB.bed.gz
https://www.encodeproject.org/files/ENCFF979NIV/@@download/ENCFF979NIV.bed.gz
https://www.encodeproject.org/files/ENCFF067OMU/@@download/ENCFF067OMU.bed.gz
https://www.encodeproject.org/files/ENCFF020FWO/@@download/ENCFF020FWO.bed.gz
https://www.encodeproject.org/files/ENCFF241JMO/@@download/ENCFF241JMO.bed.gz
https://www.encodeproject.org/files/ENCFF716SOS/@@download/ENCFF716SOS.bed.gz
https://www.encodeproject.org/files/ENCFF871EKB/@@download/ENCFF871EKB.bed.gz
https://www.encodeproject.org/files/ENCFF384SLZ/@@download/ENCFF384SLZ.bed.gz
https://www.encodeproject.org/files/ENCFF116GQA/@@download/ENCFF116GQA.bed.gz
https://www.encodeproject.org/files/ENCFF846BDG/@@download/ENCFF846BDG.bed.gz
https://www.encodeproject.org/files/ENCFF635WCC/@@download/ENCFF635WCC.bed.gz
https://www.encodeproject.org/files/ENCFF008DLV/@@download/ENCFF008DLV.bed.gz
https://www.encodeproject.org/files/ENCFF601TZC/@@download/ENCFF601TZC.bed.gz
https://www.encodeproject.org/files/ENCFF233TRB/@@download/ENCFF233TRB.bed.gz
https://www.encodeproject.org/files/ENCFF473MAD/@@download/ENCFF473MAD.bed.gz
https://www.encodeproject.org/files/ENCFF905NQU/@@download/ENCFF905NQU.bed.gz
https://www.encodeproject.org/files/ENCFF661OPZ/@@download/ENCFF661OPZ.bed.gz
https://www.encodeproject.org/files/ENCFF010EEN/@@download/ENCFF010EEN.bed.gz
https://www.encodeproject.org/files/ENCFF444VNW/@@download/ENCFF444VNW.bed.gz
https://www.encodeproject.org/files/ENCFF005DMK/@@download/ENCFF005DMK.bed.gz
https://www.encodeproject.org/files/ENCFF808FRN/@@download/ENCFF808FRN.bed.gz
https://www.encodeproject.org/files/ENCFF450XZG/@@download/ENCFF450XZG.bed.gz
https://www.encodeproject.org/files/ENCFF174VRM/@@download/ENCFF174VRM.bed.gz
https://www.encodeproject.org/files/ENCFF401FZM/@@download/ENCFF401FZM.bed.gz
https://www.encodeproject.org/files/ENCFF146VYU/@@download/ENCFF146VYU.bed.gz
https://www.encodeproject.org/files/ENCFF939RCS/@@download/ENCFF939RCS.bed.gz

Supplementary Table 1

https://www.encodeproject.org/files/ENCFF147KNW/@@download/ENCFF147KNW.bed.gz
https://www.encodeproject.org/files/ENCFF647GWK/@@download/ENCFF647GWK.bed.gz
https://www.encodeproject.org/files/ENCFF883JEC/@@download/ENCFF883JEC.bed.gz
https://www.encodeproject.org/files/ENCFF108GEI/@@download/ENCFF108GEI.bed.gz
https://www.encodeproject.org/files/ENCFF198WIN/@@download/ENCFF198WIN.bed.gz
https://www.encodeproject.org/files/ENCFF952DIQ/@@download/ENCFF952DIQ.bed.gz
https://www.encodeproject.org/files/ENCFF613FIB/@@download/ENCFF613FIB.bed.gz
https://www.encodeproject.org/files/ENCFF059TKF/@@download/ENCFF059TKF.bed.gz
https://www.encodeproject.org/files/ENCFF911WSI/@@download/ENCFF911WSI.bed.gz
https://www.encodeproject.org/files/ENCFF358VDV/@@download/ENCFF358VDV.bed.gz
https://www.encodeproject.org/files/ENCFF719SHZ/@@download/ENCFF719SHZ.bed.gz
https://www.encodeproject.org/files/ENCFF559XLA/@@download/ENCFF559XLA.bed.gz
https://www.encodeproject.org/files/ENCFF007GQK/@@download/ENCFF007GQK.bed.gz
https://www.encodeproject.org/files/ENCFF045HVB/@@download/ENCFF045HVB.bed.gz
https://www.encodeproject.org/files/ENCFF001SRD/@@download/ENCFF001SRD.bed.gz
https://www.encodeproject.org/files/ENCFF001WAK/@@download/ENCFF001WAK.bed.gz
https://www.encodeproject.org/files/ENCFF001WKG/@@download/ENCFF001WKG.bed.gz
https://www.encodeproject.org/files/ENCFF001WKF/@@download/ENCFF001WKF.bed.gz
https://www.encodeproject.org/files/ENCFF894ZLD/@@download/ENCFF894ZLD.bed.gz
https://www.encodeproject.org/files/ENCFF090IVS/@@download/ENCFF090IVS.bed.gz
https://www.encodeproject.org/files/ENCFF419NYS/@@download/ENCFF419NYS.bed.gz
https://www.encodeproject.org/files/ENCFF045UNP/@@download/ENCFF045UNP.bed.gz
https://www.encodeproject.org/files/ENCFF296UGM/@@download/ENCFF296UGM.bed.gz
https://www.encodeproject.org/files/ENCFF919ZKZ/@@download/ENCFF919ZKZ.bed.gz
https://www.encodeproject.org/files/ENCFF810AVG/@@download/ENCFF810AVG.bed.gz
https://www.encodeproject.org/files/ENCFF605XYH/@@download/ENCFF605XYH.bed.gz
https://www.encodeproject.org/files/ENCFF118YWY/@@download/ENCFF118YWY.bed.gz
https://www.encodeproject.org/files/ENCFF132AES/@@download/ENCFF132AES.bed.gz
https://www.encodeproject.org/files/ENCFF888YPD/@@download/ENCFF888YPD.bed.gz
https://www.encodeproject.org/files/ENCFF194OUN/@@download/ENCFF194OUN.bed.gz
https://www.encodeproject.org/files/ENCFF233MJO/@@download/ENCFF233MJO.bed.gz
https://www.encodeproject.org/files/ENCFF645BDW/@@download/ENCFF645BDW.bed.gz
https://www.encodeproject.org/files/ENCFF338PYM/@@download/ENCFF338PYM.bed.gz
https://www.encodeproject.org/files/ENCFF628IJD/@@download/ENCFF628IJD.bed.gz
https://www.encodeproject.org/files/ENCFF205MKH/@@download/ENCFF205MKH.bed.gz
https://www.encodeproject.org/files/ENCFF675VDC/@@download/ENCFF675VDC.bed.gz
https://www.encodeproject.org/files/ENCFF906SBI/@@download/ENCFF906SBI.bed.gz
https://www.encodeproject.org/files/ENCFF027SVR/@@download/ENCFF027SVR.bed.gz
https://www.encodeproject.org/files/ENCFF837AYQ/@@download/ENCFF837AYQ.bed.gz
https://www.encodeproject.org/files/ENCFF898XAX/@@download/ENCFF898XAX.bed.gz
https://www.encodeproject.org/files/ENCFF259VVH/@@download/ENCFF259VVH.bed.gz
https://www.encodeproject.org/files/ENCFF274EFD/@@download/ENCFF274EFD.bed.gz
https://www.encodeproject.org/files/ENCFF323NRZ/@@download/ENCFF323NRZ.bed.gz
https://www.encodeproject.org/files/ENCFF307XDO/@@download/ENCFF307XDO.bed.gz
https://www.encodeproject.org/files/ENCFF052IIY/@@download/ENCFF052IIY.bed.gz
https://www.encodeproject.org/files/ENCFF863CNC/@@download/ENCFF863CNC.bed.gz
https://www.encodeproject.org/files/ENCFF087TBH/@@download/ENCFF087TBH.bed.gz
https://www.encodeproject.org/files/ENCFF965XXV/@@download/ENCFF965XXV.bed.gz
https://www.encodeproject.org/files/ENCFF001SRY/@@download/ENCFF001SRY.bed.gz
https://www.encodeproject.org/files/ENCFF001WBW/@@download/ENCFF001WBW.bed.gz
https://www.encodeproject.org/files/ENCFF001WRA/@@download/ENCFF001WRA.bed.gz
https://www.encodeproject.org/files/ENCFF001WQZ/@@download/ENCFF001WQZ.bed.gz
https://www.encodeproject.org/files/ENCFF828LVR/@@download/ENCFF828LVR.bed.gz

Supplementary Table 1

https://www.encodeproject.org/files/ENCFF596SKV/@@download/ENCFF596SKV.bed.gz
https://www.encodeproject.org/files/ENCFF445GCV/@@download/ENCFF445GCV.bed.gz
https://www.encodeproject.org/files/ENCFF178BEG/@@download/ENCFF178BEG.bed.gz
https://www.encodeproject.org/files/ENCFF494VHO/@@download/ENCFF494VHO.bed.gz
https://www.encodeproject.org/files/ENCFF020LBO/@@download/ENCFF020LBO.bed.gz
https://www.encodeproject.org/files/ENCFF273CHB/@@download/ENCFF273CHB.bed.gz
https://www.encodeproject.org/files/ENCFF986PAK/@@download/ENCFF986PAK.bed.gz
https://www.encodeproject.org/files/ENCFF748XDV/@@download/ENCFF748XDV.bed.gz
https://www.encodeproject.org/files/ENCFF620RQG/@@download/ENCFF620RQG.bed.gz
https://www.encodeproject.org/files/ENCFF383ASH/@@download/ENCFF383ASH.bed.gz
https://www.encodeproject.org/files/ENCFF225DFA/@@download/ENCFF225DFA.bed.gz
https://www.encodeproject.org/files/ENCFF578BEO/@@download/ENCFF578BEO.bed.gz
https://www.encodeproject.org/files/ENCFF136JFN/@@download/ENCFF136JFN.bed.gz
https://www.encodeproject.org/files/ENCFF863TZJ/@@download/ENCFF863TZJ.bed.gz
https://www.encodeproject.org/files/ENCFF963COX/@@download/ENCFF963COX.bed.gz
https://www.encodeproject.org/files/ENCFF132MDE/@@download/ENCFF132MDE.bed.gz
https://www.encodeproject.org/files/ENCFF040GTO/@@download/ENCFF040GTO.bed.gz
https://www.encodeproject.org/files/ENCFF709GPG/@@download/ENCFF709GPG.bed.gz
https://www.encodeproject.org/files/ENCFF885SOK/@@download/ENCFF885SOK.bed.gz
https://www.encodeproject.org/files/ENCFF799AAK/@@download/ENCFF799AAK.bed.gz
https://www.encodeproject.org/files/ENCFF267VKD/@@download/ENCFF267VKD.bed.gz
https://www.encodeproject.org/files/ENCFF096NVD/@@download/ENCFF096NVD.bed.gz
https://www.encodeproject.org/files/ENCFF419OGO/@@download/ENCFF419OGO.bed.gz
https://www.encodeproject.org/files/ENCFF308HMF/@@download/ENCFF308HMF.bed.gz
https://www.encodeproject.org/files/ENCFF939NZB/@@download/ENCFF939NZB.bed.gz
https://www.encodeproject.org/files/ENCFF030CCM/@@download/ENCFF030CCM.bed.gz
https://www.encodeproject.org/files/ENCFF199PXG/@@download/ENCFF199PXG.bed.gz
https://www.encodeproject.org/files/ENCFF738DSM/@@download/ENCFF738DSM.bed.gz
https://www.encodeproject.org/files/ENCFF006LOY/@@download/ENCFF006LOY.bed.gz
https://www.encodeproject.org/files/ENCFF567KDR/@@download/ENCFF567KDR.bed.gz
https://www.encodeproject.org/files/ENCFF225NJA/@@download/ENCFF225NJA.bed.gz
https://www.encodeproject.org/files/ENCFF334EUI/@@download/ENCFF334EUI.bed.gz
https://www.encodeproject.org/files/ENCFF571UWP/@@download/ENCFF571UWP.bed.gz
https://www.encodeproject.org/files/ENCFF321LTV/@@download/ENCFF321LTV.bed.gz
https://www.encodeproject.org/files/ENCFF928MTS/@@download/ENCFF928MTS.bed.gz
https://www.encodeproject.org/files/ENCFF198ALI/@@download/ENCFF198ALI.bed.gz
https://www.encodeproject.org/files/ENCFF756EVG/@@download/ENCFF756EVG.bed.gz
https://www.encodeproject.org/files/ENCFF787RYE/@@download/ENCFF787RYE.bed.gz
https://www.encodeproject.org/files/ENCFF662SZI/@@download/ENCFF662SZI.bed.gz
https://www.encodeproject.org/files/ENCFF121NZP/@@download/ENCFF121NZP.bed.gz
https://www.encodeproject.org/files/ENCFF013FRI/@@download/ENCFF013FRI.bed.gz
https://www.encodeproject.org/files/ENCFF261WWC/@@download/ENCFF261WWC.bed.gz
https://www.encodeproject.org/files/ENCFF766NHL/@@download/ENCFF766NHL.bed.gz
https://www.encodeproject.org/files/ENCFF825DDS/@@download/ENCFF825DDS.bed.gz
https://www.encodeproject.org/files/ENCFF231HAY/@@download/ENCFF231HAY.bed.gz
https://www.encodeproject.org/files/ENCFF835NPW/@@download/ENCFF835NPW.bed.gz
https://www.encodeproject.org/files/ENCFF169THH/@@download/ENCFF169THH.bed.gz
https://www.encodeproject.org/files/ENCFF348CJE/@@download/ENCFF348CJE.bed.gz
https://www.encodeproject.org/files/ENCFF391BRB/@@download/ENCFF391BRB.bed.gz
https://www.encodeproject.org/files/ENCFF159AMT/@@download/ENCFF159AMT.bed.gz
https://www.encodeproject.org/files/ENCFF617DDH/@@download/ENCFF617DDH.bed.gz
https://www.encodeproject.org/files/ENCFF680GPQ/@@download/ENCFF680GPQ.bed.gz
https://www.encodeproject.org/files/ENCFF988IWO/@@download/ENCFF988IWO.bed.gz

https://www.encodeproject.org/files/ENCFF265DAD/@@download/ENCFF265DAD.bed.gz
https://www.encodeproject.org/files/ENCFF790KEZ/@@download/ENCFF790KEZ.bed.gz
https://www.encodeproject.org/files/ENCFF830NSD/@@download/ENCFF830NSD.bed.gz
https://www.encodeproject.org/files/ENCFF422GYH/@@download/ENCFF422GYH.bed.gz
https://www.encodeproject.org/files/ENCFF195HDR/@@download/ENCFF195HDR.bed.gz
https://www.encodeproject.org/files/ENCFF776TBD/@@download/ENCFF776TBD.bed.gz
https://www.encodeproject.org/files/ENCFF004KSX/@@download/ENCFF004KSX.bed.gz
https://www.encodeproject.org/files/ENCFF437CGV/@@download/ENCFF437CGV.bed.gz
https://www.encodeproject.org/files/ENCFF705KHG/@@download/ENCFF705KHG.bed.gz
https://www.encodeproject.org/files/ENCFF616ZRX/@@download/ENCFF616ZRX.bed.gz
https://www.encodeproject.org/files/ENCFF828BFV/@@download/ENCFF828BFV.bed.gz
https://www.encodeproject.org/files/ENCFF261IPB/@@download/ENCFF261IPB.bed.gz
https://www.encodeproject.org/files/ENCFF400XZZ/@@download/ENCFF400XZZ.bed.gz
https://www.encodeproject.org/files/ENCFF683SWS/@@download/ENCFF683SWS.bed.gz
https://www.encodeproject.org/files/ENCFF690UKD/@@download/ENCFF690UKD.bed.gz
https://www.encodeproject.org/files/ENCFF690DRM/@@download/ENCFF690DRM.bed.gz
https://www.encodeproject.org/files/ENCFF254WWG/@@download/ENCFF254WWG.bed.gz
https://www.encodeproject.org/files/ENCFF881BUS/@@download/ENCFF881BUS.bed.gz
https://www.encodeproject.org/files/ENCFF898WTK/@@download/ENCFF898WTK.bed.gz
https://www.encodeproject.org/files/ENCFF012XVV/@@download/ENCFF012XVV.bed.gz
https://www.encodeproject.org/files/ENCFF277WMS/@@download/ENCFF277WMS.bed.gz
https://www.encodeproject.org/files/ENCFF664QUB/@@download/ENCFF664QUB.bed.gz
https://www.encodeproject.org/files/ENCFF288VSL/@@download/ENCFF288VSL.bed.gz
https://www.encodeproject.org/files/ENCFF143DJJ/@@download/ENCFF143DJJ.bed.gz
https://www.encodeproject.org/files/ENCFF742RPQ/@@download/ENCFF742RPQ.bed.gz
https://www.encodeproject.org/files/ENCFF052SVM/@@download/ENCFF052SVM.bed.gz
https://www.encodeproject.org/files/ENCFF642WZB/@@download/ENCFF642WZB.bed.gz
https://www.encodeproject.org/files/ENCFF086KUU/@@download/ENCFF086KUU.bed.gz
https://www.encodeproject.org/files/ENCFF851CRN/@@download/ENCFF851CRN.bed.gz
https://www.encodeproject.org/files/ENCFF245PZC/@@download/ENCFF245PZC.bed.gz
https://www.encodeproject.org/files/ENCFF921EBT/@@download/ENCFF921EBT.bed.gz
https://www.encodeproject.org/files/ENCFF619GCQ/@@download/ENCFF619GCQ.bed.gz
https://www.encodeproject.org/files/ENCFF926GXK/@@download/ENCFF926GXK.bed.gz
https://www.encodeproject.org/files/ENCFF488SRU/@@download/ENCFF488SRU.bed.gz
https://www.encodeproject.org/files/ENCFF725WHX/@@download/ENCFF725WHX.bed.gz
https://www.encodeproject.org/files/ENCFF414HEV/@@download/ENCFF414HEV.bed.gz
https://www.encodeproject.org/files/ENCFF084INH/@@download/ENCFF084INH.bed.gz
https://www.encodeproject.org/files/ENCFF416PMR/@@download/ENCFF416PMR.bed.gz
https://www.encodeproject.org/files/ENCFF897TZA/@@download/ENCFF897TZA.bed.gz
https://www.encodeproject.org/files/ENCFF847WTW/@@download/ENCFF847WTW.bed.gz
https://www.encodeproject.org/files/ENCFF808AWD/@@download/ENCFF808AWD.bed.gz
https://www.encodeproject.org/files/ENCFF213TVS/@@download/ENCFF213TVS.bed.gz
https://www.encodeproject.org/files/ENCFF551DHI/@@download/ENCFF551DHI.bed.gz
https://www.encodeproject.org/files/ENCFF019PZF/@@download/ENCFF019PZF.bed.gz
https://www.encodeproject.org/files/ENCFF992AXG/@@download/ENCFF992AXG.bed.gz
https://www.encodeproject.org/files/ENCFF961JXL/@@download/ENCFF961JXL.bed.gz
https://www.encodeproject.org/files/ENCFF917MLF/@@download/ENCFF917MLF.bed.gz
https://www.encodeproject.org/files/ENCFF278XGA/@@download/ENCFF278XGA.bed.gz
https://www.encodeproject.org/files/ENCFF485PWM/@@download/ENCFF485PWM.bed.gz
https://www.encodeproject.org/files/ENCFF687COE/@@download/ENCFF687COE.bed.gz
https://www.encodeproject.org/files/ENCFF001SRL/@@download/ENCFF001SRL.bed.gz
https://www.encodeproject.org/files/ENCFF001WLK/@@download/ENCFF001WLK.bed.gz
https://www.encodeproject.org/files/ENCFF201RNY/@@download/ENCFF201RNY.bed.gz

Supplementary Table 1

https://www.encodeproject.org/files/ENCFF897JBO/@@download/ENCFF897JBO.bed.gz
https://www.encodeproject.org/files/ENCFF265EUS/@@download/ENCFF265EUS.bed.gz
https://www.encodeproject.org/files/ENCFF506IBG/@@download/ENCFF506IBG.bed.gz
https://www.encodeproject.org/files/ENCFF811KVP/@@download/ENCFF811KVP.bed.gz
https://www.encodeproject.org/files/ENCFF084PQH/@@download/ENCFF084PQH.bed.gz
https://www.encodeproject.org/files/ENCFF010ACH/@@download/ENCFF010ACH.bed.gz
https://www.encodeproject.org/files/ENCFF811FJV/@@download/ENCFF811FJV.bed.gz
https://www.encodeproject.org/files/ENCFF058TRU/@@download/ENCFF058TRU.bed.gz
https://www.encodeproject.org/files/ENCFF484DDR/@@download/ENCFF484DDR.bed.gz
https://www.encodeproject.org/files/ENCFF116DHO/@@download/ENCFF116DHO.bed.gz
https://www.encodeproject.org/files/ENCFF459SPU/@@download/ENCFF459SPU.bed.gz
https://www.encodeproject.org/files/ENCFF577FOX/@@download/ENCFF577FOX.bed.gz
https://www.encodeproject.org/files/ENCFF073TWU/@@download/ENCFF073TWU.bed.gz
https://www.encodeproject.org/files/ENCFF426OFK/@@download/ENCFF426OFK.bed.gz
https://www.encodeproject.org/files/ENCFF569RJR/@@download/ENCFF569RJR.bed.gz
https://www.encodeproject.org/files/ENCFF863LIV/@@download/ENCFF863LIV.bed.gz
https://www.encodeproject.org/files/ENCFF341ZYC/@@download/ENCFF341ZYC.bed.gz
https://www.encodeproject.org/files/ENCFF143QFM/@@download/ENCFF143QFM.bed.gz
https://www.encodeproject.org/files/ENCFF183EXC/@@download/ENCFF183EXC.bed.gz
https://www.encodeproject.org/files/ENCFF985BXZ/@@download/ENCFF985BXZ.bed.gz
https://www.encodeproject.org/files/ENCFF832GVN/@@download/ENCFF832GVN.bed.gz
https://www.encodeproject.org/files/ENCFF820RVX/@@download/ENCFF820RVX.bed.gz
https://www.encodeproject.org/files/ENCFF590FZJ/@@download/ENCFF590FZJ.bed.gz
https://www.encodeproject.org/files/ENCFF360ADQ/@@download/ENCFF360ADQ.bed.gz
https://www.encodeproject.org/files/ENCFF247ZAZ/@@download/ENCFF247ZAZ.bed.gz
https://www.encodeproject.org/files/ENCFF536KAC/@@download/ENCFF536KAC.bed.gz
https://www.encodeproject.org/files/ENCFF391DGU/@@download/ENCFF391DGU.bed.gz
https://www.encodeproject.org/files/ENCFF274FIO/@@download/ENCFF274FIO.bed.gz
https://www.encodeproject.org/files/ENCFF811USD/@@download/ENCFF811USD.bed.gz
https://www.encodeproject.org/files/ENCFF173MJQ/@@download/ENCFF173MJQ.bed.gz
https://www.encodeproject.org/files/ENCFF318JGR/@@download/ENCFF318JGR.bed.gz
https://www.encodeproject.org/files/ENCFF762HSP/@@download/ENCFF762HSP.bed.gz
https://www.encodeproject.org/files/ENCFF803XNU/@@download/ENCFF803XNU.bed.gz
https://www.encodeproject.org/files/ENCFF272WHU/@@download/ENCFF272WHU.bed.gz
https://www.encodeproject.org/files/ENCFF701IYS/@@download/ENCFF701IYS.bed.gz
https://www.encodeproject.org/files/ENCFF064SMJ/@@download/ENCFF064SMJ.bed.gz
https://www.encodeproject.org/files/ENCFF580VSJ/@@download/ENCFF580VSJ.bed.gz
https://www.encodeproject.org/files/ENCFF949RAR/@@download/ENCFF949RAR.bed.gz
https://www.encodeproject.org/files/ENCFF163AYI/@@download/ENCFF163AYI.bed.gz
https://www.encodeproject.org/files/ENCFF001WCU/@@download/ENCFF001WCU.bed.gz
https://www.encodeproject.org/files/ENCFF001WTO/@@download/ENCFF001WTO.bed.gz
https://www.encodeproject.org/files/ENCFF587LEX/@@download/ENCFF587LEX.bed.gz
https://www.encodeproject.org/files/ENCFF837QHQ/@@download/ENCFF837QHQ.bed.gz
https://www.encodeproject.org/files/ENCFF420UQF/@@download/ENCFF420UQF.bed.gz
https://www.encodeproject.org/files/ENCFF144XMY/@@download/ENCFF144XMY.bed.gz
https://www.encodeproject.org/files/ENCFF395ABD/@@download/ENCFF395ABD.bed.gz
https://www.encodeproject.org/files/ENCFF891DND/@@download/ENCFF891DND.bed.gz
https://www.encodeproject.org/files/ENCFF948LTX/@@download/ENCFF948LTX.bed.gz
https://www.encodeproject.org/files/ENCFF579IWV/@@download/ENCFF579IWV.bed.gz
https://www.encodeproject.org/files/ENCFF936ENC/@@download/ENCFF936ENC.bed.gz
https://www.encodeproject.org/files/ENCFF883WWT/@@download/ENCFF883WWT.bed.gz
https://www.encodeproject.org/files/ENCFF279CHX/@@download/ENCFF279CHX.bed.gz
https://www.encodeproject.org/files/ENCFF119HQB/@@download/ENCFF119HQB.bed.gz

https://www.encodeproject.org/files/ENCFF037QQA/@@download/ENCFF037QQA.bed.gz
https://www.encodeproject.org/files/ENCFF009EMB/@@download/ENCFF009EMB.bed.gz
https://www.encodeproject.org/files/ENCFF315CSH/@@download/ENCFF315CSH.bed.gz
https://www.encodeproject.org/files/ENCFF829ZEZ/@@download/ENCFF829ZEZ.bed.gz
https://www.encodeproject.org/files/ENCFF551HRI/@@download/ENCFF551HRI.bed.gz
https://www.encodeproject.org/files/ENCFF930SLU/@@download/ENCFF930SLU.bed.gz
https://www.encodeproject.org/files/ENCFF618FRQ/@@download/ENCFF618FRQ.bed.gz
https://www.encodeproject.org/files/ENCFF649BXV/@@download/ENCFF649BXV.bed.gz
https://www.encodeproject.org/files/ENCFF001SQM/@@download/ENCFF001SQM.bed.gz
https://www.encodeproject.org/files/ENCFF001VZI/@@download/ENCFF001VZI.bed.gz
https://www.encodeproject.org/files/ENCFF001WGY/@@download/ENCFF001WGY.bed.gz
https://www.encodeproject.org/files/ENCFF001WGX/@@download/ENCFF001WGX.bed.gz
https://www.encodeproject.org/files/ENCFF111AGF/@@download/ENCFF111AGF.bed.gz
https://www.encodeproject.org/files/ENCFF583FVY/@@download/ENCFF583FVY.bed.gz
https://www.encodeproject.org/files/ENCFF698ZFJ/@@download/ENCFF698ZFJ.bed.gz
https://www.encodeproject.org/files/ENCFF662JPI/@@download/ENCFF662JPI.bed.gz
https://www.encodeproject.org/files/ENCFF001SRV/@@download/ENCFF001SRV.bed.gz
https://www.encodeproject.org/files/ENCFF001WQG/@@download/ENCFF001WQG.bed.gz
https://www.encodeproject.org/files/ENCFF581KXE/@@download/ENCFF581KXE.bed.gz
https://www.encodeproject.org/files/ENCFF289XSM/@@download/ENCFF289XSM.bed.gz
https://www.encodeproject.org/files/ENCFF669OTR/@@download/ENCFF669OTR.bed.gz
https://www.encodeproject.org/files/ENCFF038AUV/@@download/ENCFF038AUV.bed.gz
https://www.encodeproject.org/files/ENCFF110FXW/@@download/ENCFF110FXW.bed.gz
https://www.encodeproject.org/files/ENCFF937WLI/@@download/ENCFF937WLI.bed.gz
https://www.encodeproject.org/files/ENCFF045KGY/@@download/ENCFF045KGY.bed.gz
https://www.encodeproject.org/files/ENCFF696SPY/@@download/ENCFF696SPY.bed.gz
https://www.encodeproject.org/files/ENCFF341TLD/@@download/ENCFF341TLD.bed.gz
https://www.encodeproject.org/files/ENCFF687CLH/@@download/ENCFF687CLH.bed.gz
https://www.encodeproject.org/files/ENCFF590UPO/@@download/ENCFF590UPO.bed.gz
https://www.encodeproject.org/files/ENCFF154TFX/@@download/ENCFF154TFX.bed.gz
https://www.encodeproject.org/files/ENCFF424GPY/@@download/ENCFF424GPY.bed.gz
https://www.encodeproject.org/files/ENCFF859JIA/@@download/ENCFF859JIA.bed.gz
https://www.encodeproject.org/files/ENCFF095EZR/@@download/ENCFF095EZR.bed.gz
https://www.encodeproject.org/files/ENCFF631XSP/@@download/ENCFF631XSP.bed.gz
https://www.encodeproject.org/files/ENCFF738QCO/@@download/ENCFF738QCO.bed.gz
https://www.encodeproject.org/files/ENCFF194DCA/@@download/ENCFF194DCA.bed.gz
https://www.encodeproject.org/files/ENCFF636QEY/@@download/ENCFF636QEY.bed.gz
https://www.encodeproject.org/files/ENCFF725NMZ/@@download/ENCFF725NMZ.bed.gz
https://www.encodeproject.org/files/ENCFF326IQU/@@download/ENCFF326IQU.bed.gz
https://www.encodeproject.org/files/ENCFF709TJW/@@download/ENCFF709TJW.bed.gz
https://www.encodeproject.org/files/ENCFF877XCF/@@download/ENCFF877XCF.bed.gz
https://www.encodeproject.org/files/ENCFF803IZB/@@download/ENCFF803IZB.bed.gz
https://www.encodeproject.org/files/ENCFF847JTV/@@download/ENCFF847JTV.bed.gz
https://www.encodeproject.org/files/ENCFF666CHE/@@download/ENCFF666CHE.bed.gz
https://www.encodeproject.org/files/ENCFF857MFE/@@download/ENCFF857MFE.bed.gz
https://www.encodeproject.org/files/ENCFF398ENA/@@download/ENCFF398ENA.bed.gz
https://www.encodeproject.org/files/ENCFF897PRD/@@download/ENCFF897PRD.bed.gz
https://www.encodeproject.org/files/ENCFF032FWK/@@download/ENCFF032FWK.bed.gz
https://www.encodeproject.org/files/ENCFF890KUA/@@download/ENCFF890KUA.bed.gz
https://www.encodeproject.org/files/ENCFF403SUB/@@download/ENCFF403SUB.bed.gz
https://www.encodeproject.org/files/ENCFF966FIA/@@download/ENCFF966FIA.bed.gz
https://www.encodeproject.org/files/ENCFF251ZGK/@@download/ENCFF251ZGK.bed.gz
https://www.encodeproject.org/files/ENCFF600DTC/@@download/ENCFF600DTC.bed.gz

https://www.encodeproject.org/files/ENCFF001WJT/@@download/ENCFF001WJT.bed.gz
https://www.encodeproject.org/files/ENCFF001WJU/@@download/ENCFF001WJU.bed.gz
https://www.encodeproject.org/files/ENCFF710XFX/@@download/ENCFF710XFX.bed.gz
https://www.encodeproject.org/files/ENCFF109MGN/@@download/ENCFF109MGN.bed.gz
https://www.encodeproject.org/files/ENCFF672BMB/@@download/ENCFF672BMB.bed.gz
https://www.encodeproject.org/files/ENCFF757LPN/@@download/ENCFF757LPN.bed.gz
https://www.encodeproject.org/files/ENCFF516NFM/@@download/ENCFF516NFM.bed.gz
https://www.encodeproject.org/files/ENCFF915VUW/@@download/ENCFF915VUW.bed.gz
https://www.encodeproject.org/files/ENCFF568PXP/@@download/ENCFF568PXP.bed.gz
https://www.encodeproject.org/files/ENCFF405PWW/@@download/ENCFF405PWW.bed.gz
https://www.encodeproject.org/files/ENCFF964EQB/@@download/ENCFF964EQB.bed.gz
https://www.encodeproject.org/files/ENCFF584REQ/@@download/ENCFF584REQ.bed.gz
https://www.encodeproject.org/files/ENCFF112EXY/@@download/ENCFF112EXY.bed.gz
https://www.encodeproject.org/files/ENCFF388STW/@@download/ENCFF388STW.bed.gz
https://www.encodeproject.org/files/ENCFF488OAJ/@@download/ENCFF488OAJ.bed.gz
https://www.encodeproject.org/files/ENCFF523BCF/@@download/ENCFF523BCF.bed.gz
https://www.encodeproject.org/files/ENCFF534JLS/@@download/ENCFF534JLS.bed.gz
https://www.encodeproject.org/files/ENCFF921KRC/@@download/ENCFF921KRC.bed.gz
https://www.encodeproject.org/files/ENCFF579PHD/@@download/ENCFF579PHD.bed.gz
https://www.encodeproject.org/files/ENCFF360YZY/@@download/ENCFF360YZY.bed.gz
https://www.encodeproject.org/files/ENCFF242XKH/@@download/ENCFF242XKH.bed.gz
https://www.encodeproject.org/files/ENCFF009MPE/@@download/ENCFF009MPE.bed.gz
https://www.encodeproject.org/files/ENCFF925SKQ/@@download/ENCFF925SKQ.bed.gz
https://www.encodeproject.org/files/ENCFF905VNX/@@download/ENCFF905VNX.bed.gz
https://www.encodeproject.org/files/ENCFF001UUJ/@@download/ENCFF001UUJ.bed.gz
https://www.encodeproject.org/files/ENCFF855HES/@@download/ENCFF855HES.bed.gz
https://www.encodeproject.org/files/ENCFF255NTQ/@@download/ENCFF255NTQ.bed.gz
https://www.encodeproject.org/files/ENCFF144EGT/@@download/ENCFF144EGT.bed.gz
https://www.encodeproject.org/files/ENCFF281ASO/@@download/ENCFF281ASO.bed.gz
https://www.encodeproject.org/files/ENCFF301AOO/@@download/ENCFF301AOO.bed.gz
https://www.encodeproject.org/files/ENCFF246RTJ/@@download/ENCFF246RTJ.bed.gz
https://www.encodeproject.org/files/ENCFF839BDR/@@download/ENCFF839BDR.bed.gz
https://www.encodeproject.org/files/ENCFF175SSY/@@download/ENCFF175SSY.bed.gz
https://www.encodeproject.org/files/ENCFF111IXG/@@download/ENCFF111IXG.bed.gz
https://www.encodeproject.org/files/ENCFF173CIO/@@download/ENCFF173CIO.bed.gz
https://www.encodeproject.org/files/ENCFF237GDG/@@download/ENCFF237GDG.bed.gz
https://www.encodeproject.org/files/ENCFF115NGF/@@download/ENCFF115NGF.bed.gz
https://www.encodeproject.org/files/ENCFF001SRB/@@download/ENCFF001SRB.bed.gz
https://www.encodeproject.org/files/ENCFF001WAE/@@download/ENCFF001WAE.bed.gz
https://www.encodeproject.org/files/ENCFF001WJX/@@download/ENCFF001WJX.bed.gz
https://www.encodeproject.org/files/ENCFF001WJY/@@download/ENCFF001WJY.bed.gz
https://www.encodeproject.org/files/ENCFF066PEB/@@download/ENCFF066PEB.bed.gz
https://www.encodeproject.org/files/ENCFF772YRU/@@download/ENCFF772YRU.bed.gz
https://www.encodeproject.org/files/ENCFF387KMX/@@download/ENCFF387KMX.bed.gz
https://www.encodeproject.org/files/ENCFF042ALK/@@download/ENCFF042ALK.bed.gz
https://www.encodeproject.org/files/ENCFF654MTB/@@download/ENCFF654MTB.bed.gz
https://www.encodeproject.org/files/ENCFF014POT/@@download/ENCFF014POT.bed.gz
https://www.encodeproject.org/files/ENCFF801FQN/@@download/ENCFF801FQN.bed.gz
https://www.encodeproject.org/files/ENCFF826OER/@@download/ENCFF826OER.bed.gz
https://www.encodeproject.org/files/ENCFF040SYZ/@@download/ENCFF040SYZ.bed.gz
https://www.encodeproject.org/files/ENCFF836ZPU/@@download/ENCFF836ZPU.bed.gz
https://www.encodeproject.org/files/ENCFF851EIO/@@download/ENCFF851EIO.bed.gz
https://www.encodeproject.org/files/ENCFF349OBE/@@download/ENCFF349OBE.bed.gz

https://www.encodeproject.org/files/ENCFF281XUZ/@@download/ENCFF281XUZ.bed.gz
https://www.encodeproject.org/files/ENCFF531XHH/@@download/ENCFF531XHH.bed.gz
https://www.encodeproject.org/files/ENCFF623POM/@@download/ENCFF623POM.bed.gz
https://www.encodeproject.org/files/ENCFF653SUH/@@download/ENCFF653SUH.bed.gz
https://www.encodeproject.org/files/ENCFF841AYF/@@download/ENCFF841AYF.bed.gz
https://www.encodeproject.org/files/ENCFF095QAG/@@download/ENCFF095QAG.bed.gz
https://www.encodeproject.org/files/ENCFF879ARW/@@download/ENCFF879ARW.bed.gz
https://www.encodeproject.org/files/ENCFF058ACJ/@@download/ENCFF058ACJ.bed.gz
https://www.encodeproject.org/files/ENCFF532WPU/@@download/ENCFF532WPU.bed.gz
https://www.encodeproject.org/files/ENCFF983IBW/@@download/ENCFF983IBW.bed.gz
https://www.encodeproject.org/files/ENCFF027EJI/@@download/ENCFF027EJI.bed.gz
https://www.encodeproject.org/files/ENCFF104FUO/@@download/ENCFF104FUO.bed.gz
https://www.encodeproject.org/files/ENCFF447LDJ/@@download/ENCFF447LDJ.bed.gz
https://www.encodeproject.org/files/ENCFF920DDN/@@download/ENCFF920DDN.bed.gz
https://www.encodeproject.org/files/ENCFF118DRT/@@download/ENCFF118DRT.bed.gz
https://www.encodeproject.org/files/ENCFF989JGF/@@download/ENCFF989JGF.bed.gz
https://www.encodeproject.org/files/ENCFF472SZR/@@download/ENCFF472SZR.bed.gz
https://www.encodeproject.org/files/ENCFF605QDV/@@download/ENCFF605QDV.bed.gz
https://www.encodeproject.org/files/ENCFF087XDG/@@download/ENCFF087XDG.bed.gz
https://www.encodeproject.org/files/ENCFF692AJW/@@download/ENCFF692AJW.bed.gz
https://www.encodeproject.org/files/ENCFF711OGS/@@download/ENCFF711OGS.bed.gz
https://www.encodeproject.org/files/ENCFF989NHM/@@download/ENCFF989NHM.bed.gz
https://www.encodeproject.org/files/ENCFF102IYI/@@download/ENCFF102IYI.bed.gz
https://www.encodeproject.org/files/ENCFF050MNJ/@@download/ENCFF050MNJ.bed.gz
https://www.encodeproject.org/files/ENCFF186QHO/@@download/ENCFF186QHO.bed.gz
https://www.encodeproject.org/files/ENCFF061UBG/@@download/ENCFF061UBG.bed.gz
https://www.encodeproject.org/files/ENCFF285FXR/@@download/ENCFF285FXR.bed.gz
https://www.encodeproject.org/files/ENCFF790SST/@@download/ENCFF790SST.bed.gz
https://www.encodeproject.org/files/ENCFF526QUD/@@download/ENCFF526QUD.bed.gz
https://www.encodeproject.org/files/ENCFF627ACQ/@@download/ENCFF627ACQ.bed.gz
https://www.encodeproject.org/files/ENCFF424PWV/@@download/ENCFF424PWV.bed.gz
https://www.encodeproject.org/files/ENCFF941FIK/@@download/ENCFF941FIK.bed.gz
https://www.encodeproject.org/files/ENCFF057CCO/@@download/ENCFF057CCO.bed.gz
https://www.encodeproject.org/files/ENCFF523KLV/@@download/ENCFF523KLV.bed.gz
https://www.encodeproject.org/files/ENCFF552TQZ/@@download/ENCFF552TQZ.bed.gz
https://www.encodeproject.org/files/ENCFF072PQR/@@download/ENCFF072PQR.bed.gz
https://www.encodeproject.org/files/ENCFF603QCZ/@@download/ENCFF603QCZ.bed.gz
https://www.encodeproject.org/files/ENCFF629LQR/@@download/ENCFF629LQR.bed.gz
https://www.encodeproject.org/files/ENCFF456NFP/@@download/ENCFF456NFP.bed.gz
https://www.encodeproject.org/files/ENCFF200RYP/@@download/ENCFF200RYP.bed.gz
https://www.encodeproject.org/files/ENCFF453AAS/@@download/ENCFF453AAS.bed.gz
https://www.encodeproject.org/files/ENCFF825CIW/@@download/ENCFF825CIW.bed.gz
https://www.encodeproject.org/files/ENCFF301ARM/@@download/ENCFF301ARM.bed.gz
https://www.encodeproject.org/files/ENCFF722OVO/@@download/ENCFF722OVO.bed.gz
https://www.encodeproject.org/files/ENCFF746BBB/@@download/ENCFF746BBB.bed.gz
https://www.encodeproject.org/files/ENCFF698LRC/@@download/ENCFF698LRC.bed.gz
https://www.encodeproject.org/files/ENCFF037SOW/@@download/ENCFF037SOW.bed.gz
https://www.encodeproject.org/files/ENCFF900TMY/@@download/ENCFF900TMY.bed.gz
https://www.encodeproject.org/files/ENCFF426FAC/@@download/ENCFF426FAC.bed.gz
https://www.encodeproject.org/files/ENCFF276TVG/@@download/ENCFF276TVG.bed.gz
https://www.encodeproject.org/files/ENCFF143MFN/@@download/ENCFF143MFN.bed.gz
https://www.encodeproject.org/files/ENCFF077UJK/@@download/ENCFF077UJK.bed.gz
https://www.encodeproject.org/files/ENCFF853GQY/@@download/ENCFF853GQY.bed.gz

https://www.encodeproject.org/files/ENCFF365FGD/@@download/ENCFF365FGD.bed.gz
https://www.encodeproject.org/files/ENCFF051EYR/@@download/ENCFF051EYR.bed.gz
https://www.encodeproject.org/files/ENCFF374BCD/@@download/ENCFF374BCD.bed.gz
https://www.encodeproject.org/files/ENCFF841MMD/@@download/ENCFF841MMD.bed.gz
https://www.encodeproject.org/files/ENCFF346ZSZ/@@download/ENCFF346ZSZ.bed.gz
https://www.encodeproject.org/files/ENCFF293TWN/@@download/ENCFF293TWN.bed.gz
https://www.encodeproject.org/files/ENCFF897GBH/@@download/ENCFF897GBH.bed.gz
https://www.encodeproject.org/files/ENCFF876IKZ/@@download/ENCFF876IKZ.bed.gz
https://www.encodeproject.org/files/ENCFF535KCZ/@@download/ENCFF535KCZ.bed.gz
https://www.encodeproject.org/files/ENCFF306IHB/@@download/ENCFF306IHB.bed.gz
https://www.encodeproject.org/files/ENCFF132DVF/@@download/ENCFF132DVF.bed.gz
https://www.encodeproject.org/files/ENCFF037JUZ/@@download/ENCFF037JUZ.bed.gz
https://www.encodeproject.org/files/ENCFF095GEI/@@download/ENCFF095GEI.bed.gz
https://www.encodeproject.org/files/ENCFF316XWU/@@download/ENCFF316XWU.bed.gz
https://www.encodeproject.org/files/ENCFF166VSZ/@@download/ENCFF166VSZ.bed.gz
https://www.encodeproject.org/files/ENCFF067JJG/@@download/ENCFF067JJG.bed.gz
https://www.encodeproject.org/files/ENCFF444DXW/@@download/ENCFF444DXW.bed.gz
https://www.encodeproject.org/files/ENCFF978OUM/@@download/ENCFF978OUM.bed.gz
https://www.encodeproject.org/files/ENCFF357EZI/@@download/ENCFF357EZI.bed.gz
https://www.encodeproject.org/files/ENCFF438GJD/@@download/ENCFF438GJD.bed.gz
https://www.encodeproject.org/files/ENCFF210WFD/@@download/ENCFF210WFD.bed.gz
https://www.encodeproject.org/files/ENCFF205UIO/@@download/ENCFF205UIO.bed.gz
https://www.encodeproject.org/files/ENCFF745FDQ/@@download/ENCFF745FDQ.bed.gz
https://www.encodeproject.org/files/ENCFF766PCO/@@download/ENCFF766PCO.bed.gz
https://www.encodeproject.org/files/ENCFF081UUE/@@download/ENCFF081UUE.bed.gz
https://www.encodeproject.org/files/ENCFF064KOE/@@download/ENCFF064KOE.bed.gz
https://www.encodeproject.org/files/ENCFF346XDJ/@@download/ENCFF346XDJ.bed.gz
https://www.encodeproject.org/files/ENCFF675NAH/@@download/ENCFF675NAH.bed.gz
https://www.encodeproject.org/files/ENCFF457HPK/@@download/ENCFF457HPK.bed.gz
https://www.encodeproject.org/files/ENCFF001SSE/@@download/ENCFF001SSE.bed.gz
https://www.encodeproject.org/files/ENCFF001WSF/@@download/ENCFF001WSF.bed.gz
https://www.encodeproject.org/files/ENCFF001WSG/@@download/ENCFF001WSG.bed.gz
https://www.encodeproject.org/files/ENCFF171BNY/@@download/ENCFF171BNY.bed.gz
https://www.encodeproject.org/files/ENCFF162CFI/@@download/ENCFF162CFI.bed.gz
https://www.encodeproject.org/files/ENCFF218MZJ/@@download/ENCFF218MZJ.bed.gz
https://www.encodeproject.org/files/ENCFF231GLQ/@@download/ENCFF231GLQ.bed.gz
https://www.encodeproject.org/files/ENCFF926BER/@@download/ENCFF926BER.bed.gz
https://www.encodeproject.org/files/ENCFF113GMS/@@download/ENCFF113GMS.bed.gz
https://www.encodeproject.org/files/ENCFF954NAG/@@download/ENCFF954NAG.bed.gz
https://www.encodeproject.org/files/ENCFF474DSN/@@download/ENCFF474DSN.bed.gz
https://www.encodeproject.org/files/ENCFF543CWJ/@@download/ENCFF543CWJ.bed.gz
https://www.encodeproject.org/files/ENCFF960GYE/@@download/ENCFF960GYE.bed.gz
https://www.encodeproject.org/files/ENCFF196OHE/@@download/ENCFF196OHE.bed.gz
https://www.encodeproject.org/files/ENCFF524AJJ/@@download/ENCFF524AJJ.bed.gz
https://www.encodeproject.org/files/ENCFF241QSX/@@download/ENCFF241QSX.bed.gz
https://www.encodeproject.org/files/ENCFF347YCV/@@download/ENCFF347YCV.bed.gz
https://www.encodeproject.org/files/ENCFF894PDJ/@@download/ENCFF894PDJ.bed.gz
https://www.encodeproject.org/files/ENCFF493IHC/@@download/ENCFF493IHC.bed.gz
https://www.encodeproject.org/files/ENCFF164FDZ/@@download/ENCFF164FDZ.bed.gz
https://www.encodeproject.org/files/ENCFF134XVA/@@download/ENCFF134XVA.bed.gz
https://www.encodeproject.org/files/ENCFF352RNR/@@download/ENCFF352RNR.bed.gz
https://www.encodeproject.org/files/ENCFF245TMF/@@download/ENCFF245TMF.bed.gz
https://www.encodeproject.org/files/ENCFF373HMJ/@@download/ENCFF373HMJ.bed.gz

Supplementary Table 1

https://www.encodeproject.org/files/ENCFF361FJF/@@download/ENCFF361FJF.bed.gz
https://www.encodeproject.org/files/ENCFF501PXO/@@download/ENCFF501PXO.bed.gz
https://www.encodeproject.org/files/ENCFF863EHC/@@download/ENCFF863EHC.bed.gz
https://www.encodeproject.org/files/ENCFF556AXZ/@@download/ENCFF556AXZ.bed.gz
https://www.encodeproject.org/files/ENCFF926OPP/@@download/ENCFF926OPP.bed.gz
https://www.encodeproject.org/files/ENCFF662NTN/@@download/ENCFF662NTN.bed.gz
https://www.encodeproject.org/files/ENCFF738JDE/@@download/ENCFF738JDE.bed.gz
https://www.encodeproject.org/files/ENCFF004GBA/@@download/ENCFF004GBA.bed.gz
https://www.encodeproject.org/files/ENCFF878OAD/@@download/ENCFF878OAD.bed.gz
https://www.encodeproject.org/files/ENCFF720DUQ/@@download/ENCFF720DUQ.bed.gz
https://www.encodeproject.org/files/ENCFF559HGO/@@download/ENCFF559HGO.bed.gz
https://www.encodeproject.org/files/ENCFF875NRF/@@download/ENCFF875NRF.bed.gz
https://www.encodeproject.org/files/ENCFF070ZJT/@@download/ENCFF070ZJT.bed.gz
https://www.encodeproject.org/files/ENCFF199ETV/@@download/ENCFF199ETV.bed.gz
https://www.encodeproject.org/files/ENCFF102CQZ/@@download/ENCFF102CQZ.bed.gz
https://www.encodeproject.org/files/ENCFF001WQJ/@@download/ENCFF001WQJ.bed.gz
https://www.encodeproject.org/files/ENCFF001WQK/@@download/ENCFF001WQK.bed.gz
https://www.encodeproject.org/files/ENCFF376LWA/@@download/ENCFF376LWA.bed.gz
https://www.encodeproject.org/files/ENCFF386FTD/@@download/ENCFF386FTD.bed.gz
https://www.encodeproject.org/files/ENCFF254WCU/@@download/ENCFF254WCU.bed.gz
https://www.encodeproject.org/files/ENCFF547MHR/@@download/ENCFF547MHR.bed.gz
https://www.encodeproject.org/files/ENCFF454SFD/@@download/ENCFF454SFD.bed.gz
https://www.encodeproject.org/files/ENCFF419LXY/@@download/ENCFF419LXY.bed.gz
https://www.encodeproject.org/files/ENCFF772WEQ/@@download/ENCFF772WEQ.bed.gz
https://www.encodeproject.org/files/ENCFF735WAG/@@download/ENCFF735WAG.bed.gz
https://www.encodeproject.org/files/ENCFF409URA/@@download/ENCFF409URA.bed.gz
https://www.encodeproject.org/files/ENCFF728VWK/@@download/ENCFF728VWK.bed.gz
https://www.encodeproject.org/files/ENCFF973XMT/@@download/ENCFF973XMT.bed.gz
https://www.encodeproject.org/files/ENCFF382NKO/@@download/ENCFF382NKO.bed.gz
https://www.encodeproject.org/files/ENCFF308QRZ/@@download/ENCFF308QRZ.bed.gz
https://www.encodeproject.org/files/ENCFF172LKJ/@@download/ENCFF172LKJ.bed.gz
https://www.encodeproject.org/files/ENCFF492MCV/@@download/ENCFF492MCV.bed.gz
https://www.encodeproject.org/files/ENCFF515EMX/@@download/ENCFF515EMX.bed.gz
https://www.encodeproject.org/files/ENCFF221FFD/@@download/ENCFF221FFD.bed.gz
https://www.encodeproject.org/files/ENCFF798GBT/@@download/ENCFF798GBT.bed.gz
https://www.encodeproject.org/files/ENCFF835SMB/@@download/ENCFF835SMB.bed.gz
https://www.encodeproject.org/files/ENCFF216NXR/@@download/ENCFF216NXR.bed.gz
https://www.encodeproject.org/files/ENCFF410MHQ/@@download/ENCFF410MHQ.bed.gz
https://www.encodeproject.org/files/ENCFF286UIJ/@@download/ENCFF286UIJ.bed.gz
https://www.encodeproject.org/files/ENCFF046KTX/@@download/ENCFF046KTX.bed.gz
https://www.encodeproject.org/files/ENCFF537AWN/@@download/ENCFF537AWN.bed.gz
https://www.encodeproject.org/files/ENCFF353SVP/@@download/ENCFF353SVP.bed.gz
https://www.encodeproject.org/files/ENCFF130KKE/@@download/ENCFF130KKE.bed.gz
https://www.encodeproject.org/files/ENCFF173WZS/@@download/ENCFF173WZS.bed.gz
https://www.encodeproject.org/files/ENCFF220ATL/@@download/ENCFF220ATL.bed.gz
https://www.encodeproject.org/files/ENCFF299STS/@@download/ENCFF299STS.bed.gz
https://www.encodeproject.org/files/ENCFF364FFR/@@download/ENCFF364FFR.bed.gz
https://www.encodeproject.org/files/ENCFF134ZDK/@@download/ENCFF134ZDK.bed.gz
https://www.encodeproject.org/files/ENCFF128BFZ/@@download/ENCFF128BFZ.bed.gz
https://www.encodeproject.org/files/ENCFF682LFV/@@download/ENCFF682LFV.bed.gz
https://www.encodeproject.org/files/ENCFF729VSK/@@download/ENCFF729VSK.bed.gz
https://www.encodeproject.org/files/ENCFF025BJB/@@download/ENCFF025BJB.bed.gz
https://www.encodeproject.org/files/ENCFF406AAD/@@download/ENCFF406AAD.bed.gz

Supplementary Table 1

https://www.encodeproject.org/files/ENCFF363XQF/@@download/ENCFF363XQF.bed.gz
https://www.encodeproject.org/files/ENCFF818WPF/@@download/ENCFF818WPF.bed.gz
https://www.encodeproject.org/files/ENCFF971WVS/@@download/ENCFF971WVS.bed.gz
https://www.encodeproject.org/files/ENCFF370OYQ/@@download/ENCFF370OYQ.bed.gz
https://www.encodeproject.org/files/ENCFF833SIZ/@@download/ENCFF833SIZ.bed.gz
https://www.encodeproject.org/files/ENCFF800TEG/@@download/ENCFF800TEG.bed.gz
https://www.encodeproject.org/files/ENCFF909JGU/@@download/ENCFF909JGU.bed.gz
https://www.encodeproject.org/files/ENCFF735APJ/@@download/ENCFF735APJ.bed.gz
https://www.encodeproject.org/files/ENCFF390FMQ/@@download/ENCFF390FMQ.bed.gz
https://www.encodeproject.org/files/ENCFF819NTW/@@download/ENCFF819NTW.bed.gz
https://www.encodeproject.org/files/ENCFF843QJV/@@download/ENCFF843QJV.bed.gz
https://www.encodeproject.org/files/ENCFF024JBX/@@download/ENCFF024JBX.bed.gz
https://www.encodeproject.org/files/ENCFF588WQL/@@download/ENCFF588WQL.bed.gz
https://www.encodeproject.org/files/ENCFF059HII/@@download/ENCFF059HII.bed.gz
https://www.encodeproject.org/files/ENCFF429OVG/@@download/ENCFF429OVG.bed.gz
https://www.encodeproject.org/files/ENCFF807HKO/@@download/ENCFF807HKO.bed.gz
https://www.encodeproject.org/files/ENCFF684QFQ/@@download/ENCFF684QFQ.bed.gz
https://www.encodeproject.org/files/ENCFF011FIW/@@download/ENCFF011FIW.bed.gz
https://www.encodeproject.org/files/ENCFF755PMB/@@download/ENCFF755PMB.bed.gz
https://www.encodeproject.org/files/ENCFF338NUC/@@download/ENCFF338NUC.bed.gz
https://www.encodeproject.org/files/ENCFF908BGP/@@download/ENCFF908BGP.bed.gz
https://www.encodeproject.org/files/ENCFF583SXE/@@download/ENCFF583SXE.bed.gz
https://www.encodeproject.org/files/ENCFF167SDL/@@download/ENCFF167SDL.bed.gz
https://www.encodeproject.org/files/ENCFF513LFB/@@download/ENCFF513LFB.bed.gz
https://www.encodeproject.org/files/ENCFF367BEU/@@download/ENCFF367BEU.bed.gz
https://www.encodeproject.org/files/ENCFF352HSZ/@@download/ENCFF352HSZ.bed.gz
https://www.encodeproject.org/files/ENCFF050NEM/@@download/ENCFF050NEM.bed.gz
https://www.encodeproject.org/files/ENCFF189FNX/@@download/ENCFF189FNX.bed.gz
https://www.encodeproject.org/files/ENCFF819JMJ/@@download/ENCFF819JMJ.bed.gz
https://www.encodeproject.org/files/ENCFF740MJY/@@download/ENCFF740MJY.bed.gz
https://www.encodeproject.org/files/ENCFF001UYA/@@download/ENCFF001UYA.bed.gz
https://www.encodeproject.org/files/ENCFF827CXU/@@download/ENCFF827CXU.bed.gz
https://www.encodeproject.org/files/ENCFF504EOC/@@download/ENCFF504EOC.bed.gz
https://www.encodeproject.org/files/ENCFF995OBT/@@download/ENCFF995OBT.bed.gz
https://www.encodeproject.org/files/ENCFF036XTH/@@download/ENCFF036XTH.bed.gz
https://www.encodeproject.org/files/ENCFF283GEW/@@download/ENCFF283GEW.bed.gz
https://www.encodeproject.org/files/ENCFF271ZJR/@@download/ENCFF271ZJR.bed.gz
https://www.encodeproject.org/files/ENCFF283UJD/@@download/ENCFF283UJD.bed.gz
https://www.encodeproject.org/files/ENCFF020LII/@@download/ENCFF020LII.bed.gz
https://www.encodeproject.org/files/ENCFF924FPB/@@download/ENCFF924FPB.bed.gz
https://www.encodeproject.org/files/ENCFF021TGH/@@download/ENCFF021TGH.bed.gz
https://www.encodeproject.org/files/ENCFF762WIQ/@@download/ENCFF762WIQ.bed.gz
https://www.encodeproject.org/files/ENCFF839MGO/@@download/ENCFF839MGO.bed.gz
https://www.encodeproject.org/files/ENCFF270GNM/@@download/ENCFF270GNM.bed.gz
https://www.encodeproject.org/files/ENCFF871HXB/@@download/ENCFF871HXB.bed.gz
https://www.encodeproject.org/files/ENCFF498MDZ/@@download/ENCFF498MDZ.bed.gz
https://www.encodeproject.org/files/ENCFF145QKC/@@download/ENCFF145QKC.bed.gz
https://www.encodeproject.org/files/ENCFF642UFQ/@@download/ENCFF642UFQ.bed.gz
https://www.encodeproject.org/files/ENCFF205VMB/@@download/ENCFF205VMB.bed.gz
https://www.encodeproject.org/files/ENCFF468TOZ/@@download/ENCFF468TOZ.bed.gz
https://www.encodeproject.org/files/ENCFF414BFO/@@download/ENCFF414BFO.bed.gz
https://www.encodeproject.org/files/ENCFF504INS/@@download/ENCFF504INS.bed.gz
https://www.encodeproject.org/files/ENCFF306UIG/@@download/ENCFF306UIG.bed.gz

https://www.encodeproject.org/files/ENCFF587FIW/@@download/ENCFF587FIW.bed.gz
https://www.encodeproject.org/files/ENCFF728VKA/@@download/ENCFF728VKA.bed.gz
https://www.encodeproject.org/files/ENCFF962JWU/@@download/ENCFF962JWU.bed.gz
https://www.encodeproject.org/files/ENCFF251ZXX/@@download/ENCFF251ZXX.bed.gz
https://www.encodeproject.org/files/ENCFF409JTM/@@download/ENCFF409JTM.bed.gz
https://www.encodeproject.org/files/ENCFF782KJY/@@download/ENCFF782KJY.bed.gz
https://www.encodeproject.org/files/ENCFF914SZL/@@download/ENCFF914SZL.bed.gz
https://www.encodeproject.org/files/ENCFF203UET/@@download/ENCFF203UET.bed.gz
https://www.encodeproject.org/files/ENCFF281HKU/@@download/ENCFF281HKU.bed.gz
https://www.encodeproject.org/files/ENCFF975JLM/@@download/ENCFF975JLM.bed.gz
https://www.encodeproject.org/files/ENCFF140VUL/@@download/ENCFF140VUL.bed.gz
https://www.encodeproject.org/files/ENCFF803THQ/@@download/ENCFF803THQ.bed.gz
https://www.encodeproject.org/files/ENCFF058TUX/@@download/ENCFF058TUX.bed.gz
https://www.encodeproject.org/files/ENCFF126IZH/@@download/ENCFF126IZH.bed.gz
https://www.encodeproject.org/files/ENCFF001WTS/@@download/ENCFF001WTS.bed.gz
https://www.encodeproject.org/files/ENCFF345HQB/@@download/ENCFF345HQB.bed.gz
https://www.encodeproject.org/files/ENCFF481KKG/@@download/ENCFF481KKG.bed.gz
https://www.encodeproject.org/files/ENCFF570MGY/@@download/ENCFF570MGY.bed.gz
https://www.encodeproject.org/files/ENCFF619RAY/@@download/ENCFF619RAY.bed.gz
https://www.encodeproject.org/files/ENCFF772NXP/@@download/ENCFF772NXP.bed.gz
https://www.encodeproject.org/files/ENCFF231NXS/@@download/ENCFF231NXS.bed.gz
https://www.encodeproject.org/files/ENCFF874GGX/@@download/ENCFF874GGX.bed.gz
https://www.encodeproject.org/files/ENCFF542RWT/@@download/ENCFF542RWT.bed.gz
https://www.encodeproject.org/files/ENCFF684MAZ/@@download/ENCFF684MAZ.bed.gz
https://www.encodeproject.org/files/ENCFF332HJI/@@download/ENCFF332HJI.bed.gz
https://www.encodeproject.org/files/ENCFF415CEW/@@download/ENCFF415CEW.bed.gz
https://www.encodeproject.org/files/ENCFF168UIB/@@download/ENCFF168UIB.bed.gz
https://www.encodeproject.org/files/ENCFF785ORF/@@download/ENCFF785ORF.bed.gz
https://www.encodeproject.org/files/ENCFF136FZU/@@download/ENCFF136FZU.bed.gz
https://www.encodeproject.org/files/ENCFF278QPW/@@download/ENCFF278QPW.bed.gz
https://www.encodeproject.org/files/ENCFF930MUK/@@download/ENCFF930MUK.bed.gz
https://www.encodeproject.org/files/ENCFF295FPK/@@download/ENCFF295FPK.bed.gz
https://www.encodeproject.org/files/ENCFF475QCQ/@@download/ENCFF475QCQ.bed.gz
https://www.encodeproject.org/files/ENCFF213IAV/@@download/ENCFF213IAV.bed.gz
https://www.encodeproject.org/files/ENCFF311LCN/@@download/ENCFF311LCN.bed.gz
https://www.encodeproject.org/files/ENCFF721HLN/@@download/ENCFF721HLN.bed.gz
https://www.encodeproject.org/files/ENCFF970CEY/@@download/ENCFF970CEY.bed.gz
https://www.encodeproject.org/files/ENCFF265CKF/@@download/ENCFF265CKF.bed.gz
https://www.encodeproject.org/files/ENCFF388DEY/@@download/ENCFF388DEY.bed.gz
https://www.encodeproject.org/files/ENCFF001UXH/@@download/ENCFF001UXH.bed.gz
https://www.encodeproject.org/files/ENCFF659FKS/@@download/ENCFF659FKS.bed.gz
https://www.encodeproject.org/files/ENCFF933LWC/@@download/ENCFF933LWC.bed.gz
https://www.encodeproject.org/files/ENCFF299PZE/@@download/ENCFF299PZE.bed.gz
https://www.encodeproject.org/files/ENCFF350XYE/@@download/ENCFF350XYE.bed.gz
https://www.encodeproject.org/files/ENCFF001UYB/@@download/ENCFF001UYB.bed.gz
https://www.encodeproject.org/files/ENCFF449XXS/@@download/ENCFF449XXS.bed.gz
https://www.encodeproject.org/files/ENCFF121XJN/@@download/ENCFF121XJN.bed.gz
https://www.encodeproject.org/files/ENCFF990ZGF/@@download/ENCFF990ZGF.bed.gz
https://www.encodeproject.org/files/ENCFF229BEY/@@download/ENCFF229BEY.bed.gz
https://www.encodeproject.org/files/ENCFF454SUH/@@download/ENCFF454SUH.bed.gz
https://www.encodeproject.org/files/ENCFF331ODX/@@download/ENCFF331ODX.bed.gz
https://www.encodeproject.org/files/ENCFF049XKN/@@download/ENCFF049XKN.bed.gz
https://www.encodeproject.org/files/ENCFF477GLZ/@@download/ENCFF477GLZ.bed.gz

Supplementary Table 1

https://www.encodeproject.org/files/ENCFF356OSE/@@download/ENCFF356OSE.bed.gz
https://www.encodeproject.org/files/ENCFF747IZU/@@download/ENCFF747IZU.bed.gz
https://www.encodeproject.org/files/ENCFF262FHU/@@download/ENCFF262FHU.bed.gz
https://www.encodeproject.org/files/ENCFF732RRW/@@download/ENCFF732RRW.bed.gz
https://www.encodeproject.org/files/ENCFF874CCT/@@download/ENCFF874CCT.bed.gz
https://www.encodeproject.org/files/ENCFF024TPY/@@download/ENCFF024TPY.bed.gz
https://www.encodeproject.org/files/ENCFF102GUG/@@download/ENCFF102GUG.bed.gz
https://www.encodeproject.org/files/ENCFF096PKD/@@download/ENCFF096PKD.bed.gz
https://www.encodeproject.org/files/ENCFF001WTL/@@download/ENCFF001WTL.bed.gz
https://www.encodeproject.org/files/ENCFF001WTM/@@download/ENCFF001WTM.bed.gz
https://www.encodeproject.org/files/ENCFF742OKP/@@download/ENCFF742OKP.bed.gz
https://www.encodeproject.org/files/ENCFF549EOZ/@@download/ENCFF549EOZ.bed.gz
https://www.encodeproject.org/files/ENCFF654MRJ/@@download/ENCFF654MRJ.bed.gz
https://www.encodeproject.org/files/ENCFF012QDX/@@download/ENCFF012QDX.bed.gz
https://www.encodeproject.org/files/ENCFF434LIX/@@download/ENCFF434LIX.bed.gz
https://www.encodeproject.org/files/ENCFF773IYG/@@download/ENCFF773IYG.bed.gz
https://www.encodeproject.org/files/ENCFF349JWY/@@download/ENCFF349JWY.bed.gz
https://www.encodeproject.org/files/ENCFF926FFA/@@download/ENCFF926FFA.bed.gz
https://www.encodeproject.org/files/ENCFF949OGH/@@download/ENCFF949OGH.bed.gz
https://www.encodeproject.org/files/ENCFF412ONU/@@download/ENCFF412ONU.bed.gz
https://www.encodeproject.org/files/ENCFF072BNW/@@download/ENCFF072BNW.bed.gz
https://www.encodeproject.org/files/ENCFF492UJW/@@download/ENCFF492UJW.bed.gz
https://www.encodeproject.org/files/ENCFF944FSO/@@download/ENCFF944FSO.bed.gz
https://www.encodeproject.org/files/ENCFF794AMB/@@download/ENCFF794AMB.bed.gz
https://www.encodeproject.org/files/ENCFF213RJV/@@download/ENCFF213RJV.bed.gz
https://www.encodeproject.org/files/ENCFF681WSK/@@download/ENCFF681WSK.bed.gz
https://www.encodeproject.org/files/ENCFF371HNS/@@download/ENCFF371HNS.bed.gz
https://www.encodeproject.org/files/ENCFF341KCP/@@download/ENCFF341KCP.bed.gz
https://www.encodeproject.org/files/ENCFF001SQQ/@@download/ENCFF001SQQ.bed.gz
https://www.encodeproject.org/files/ENCFF001VZO/@@download/ENCFF001VZO.bed.gz
https://www.encodeproject.org/files/ENCFF001WHU/@@download/ENCFF001WHU.bed.gz
https://www.encodeproject.org/files/ENCFF001WHT/@@download/ENCFF001WHT.bed.gz
https://www.encodeproject.org/files/ENCFF185RSW/@@download/ENCFF185RSW.bed.gz
https://www.encodeproject.org/files/ENCFF690GBE/@@download/ENCFF690GBE.bed.gz
https://www.encodeproject.org/files/ENCFF653KYK/@@download/ENCFF653KYK.bed.gz
https://www.encodeproject.org/files/ENCFF528ZJQ/@@download/ENCFF528ZJQ.bed.gz
https://www.encodeproject.org/files/ENCFF339WEE/@@download/ENCFF339WEE.bed.gz
https://www.encodeproject.org/files/ENCFF233SJW/@@download/ENCFF233SJW.bed.gz
https://www.encodeproject.org/files/ENCFF300CAD/@@download/ENCFF300CAD.bed.gz
https://www.encodeproject.org/files/ENCFF866FQI/@@download/ENCFF866FQI.bed.gz
https://www.encodeproject.org/files/ENCFF609QJR/@@download/ENCFF609QJR.bed.gz
https://www.encodeproject.org/files/ENCFF850STV/@@download/ENCFF850STV.bed.gz
https://www.encodeproject.org/files/ENCFF268PYO/@@download/ENCFF268PYO.bed.gz
https://www.encodeproject.org/files/ENCFF060JZA/@@download/ENCFF060JZA.bed.gz
https://www.encodeproject.org/files/ENCFF349YPC/@@download/ENCFF349YPC.bed.gz
https://www.encodeproject.org/files/ENCFF715SDA/@@download/ENCFF715SDA.bed.gz
https://www.encodeproject.org/files/ENCFF025GJZ/@@download/ENCFF025GJZ.bed.gz
https://www.encodeproject.org/files/ENCFF007RSQ/@@download/ENCFF007RSQ.bed.gz
https://www.encodeproject.org/files/ENCFF574UTF/@@download/ENCFF574UTF.bed.gz
https://www.encodeproject.org/files/ENCFF671KDX/@@download/ENCFF671KDX.bed.gz
https://www.encodeproject.org/files/ENCFF897YVS/@@download/ENCFF897YVS.bed.gz
https://www.encodeproject.org/files/ENCFF459ZCG/@@download/ENCFF459ZCG.bed.gz
https://www.encodeproject.org/files/ENCFF819SKB/@@download/ENCFF819SKB.bed.gz

https://www.encodeproject.org/files/ENCFF457XYZ/@@download/ENCFF457XYZ.bed.gz
https://www.encodeproject.org/files/ENCFF890VPG/@@download/ENCFF890VPG.bed.gz
https://www.encodeproject.org/files/ENCFF800DOC/@@download/ENCFF800DOC.bed.gz
https://www.encodeproject.org/files/ENCFF225QPG/@@download/ENCFF225QPG.bed.gz
https://www.encodeproject.org/files/ENCFF723PRK/@@download/ENCFF723PRK.bed.gz
https://www.encodeproject.org/files/ENCFF633GQX/@@download/ENCFF633GQX.bed.gz
https://www.encodeproject.org/files/ENCFF947RDC/@@download/ENCFF947RDC.bed.gz
https://www.encodeproject.org/files/ENCFF104VLJ/@@download/ENCFF104VLJ.bed.gz
https://www.encodeproject.org/files/ENCFF145OUR/@@download/ENCFF145OUR.bed.gz
https://www.encodeproject.org/files/ENCFF977UYY/@@download/ENCFF977UYY.bed.gz
https://www.encodeproject.org/files/ENCFF687RNV/@@download/ENCFF687RNV.bed.gz
https://www.encodeproject.org/files/ENCFF675UKK/@@download/ENCFF675UKK.bed.gz
https://www.encodeproject.org/files/ENCFF094AUJ/@@download/ENCFF094AUJ.bed.gz
https://www.encodeproject.org/files/ENCFF593LQN/@@download/ENCFF593LQN.bed.gz
https://www.encodeproject.org/files/ENCFF148VKA/@@download/ENCFF148VKA.bed.gz
https://www.encodeproject.org/files/ENCFF374WRP/@@download/ENCFF374WRP.bed.gz
https://www.encodeproject.org/files/ENCFF803WAE/@@download/ENCFF803WAE.bed.gz
https://www.encodeproject.org/files/ENCFF768BAQ/@@download/ENCFF768BAQ.bed.gz
https://www.encodeproject.org/files/ENCFF216LLC/@@download/ENCFF216LLC.bed.gz
https://www.encodeproject.org/files/ENCFF020KUI/@@download/ENCFF020KUI.bed.gz
https://www.encodeproject.org/files/ENCFF929ZRA/@@download/ENCFF929ZRA.bed.gz
https://www.encodeproject.org/files/ENCFF648GGB/@@download/ENCFF648GGB.bed.gz
https://www.encodeproject.org/files/ENCFF203WKJ/@@download/ENCFF203WKJ.bed.gz
https://www.encodeproject.org/files/ENCFF238SPA/@@download/ENCFF238SPA.bed.gz
https://www.encodeproject.org/files/ENCFF699IYG/@@download/ENCFF699IYG.bed.gz
https://www.encodeproject.org/files/ENCFF218POQ/@@download/ENCFF218POQ.bed.gz
https://www.encodeproject.org/files/ENCFF632JSA/@@download/ENCFF632JSA.bed.gz
https://www.encodeproject.org/files/ENCFF343JUT/@@download/ENCFF343JUT.bed.gz
https://www.encodeproject.org/files/ENCFF523ZGF/@@download/ENCFF523ZGF.bed.gz
https://www.encodeproject.org/files/ENCFF881OJT/@@download/ENCFF881OJT.bed.gz
https://www.encodeproject.org/files/ENCFF207QQA/@@download/ENCFF207QQA.bed.gz
https://www.encodeproject.org/files/ENCFF386QCU/@@download/ENCFF386QCU.bed.gz
https://www.encodeproject.org/files/ENCFF423BJZ/@@download/ENCFF423BJZ.bed.gz
https://www.encodeproject.org/files/ENCFF709TMH/@@download/ENCFF709TMH.bed.gz
https://www.encodeproject.org/files/ENCFF564VUG/@@download/ENCFF564VUG.bed.gz
https://www.encodeproject.org/files/ENCFF012LUH/@@download/ENCFF012LUH.bed.gz
https://www.encodeproject.org/files/ENCFF249GZW/@@download/ENCFF249GZW.bed.gz
https://www.encodeproject.org/files/ENCFF085SZC/@@download/ENCFF085SZC.bed.gz
https://www.encodeproject.org/files/ENCFF935IUZ/@@download/ENCFF935IUZ.bed.gz
https://www.encodeproject.org/files/ENCFF627DYO/@@download/ENCFF627DYO.bed.gz
https://www.encodeproject.org/files/ENCFF770PXI/@@download/ENCFF770PXI.bed.gz
https://www.encodeproject.org/files/ENCFF001SRS/@@download/ENCFF001SRS.bed.gz
https://www.encodeproject.org/files/ENCFF001WML/@@download/ENCFF001WML.bed.gz
https://www.encodeproject.org/files/ENCFF001WMM/@@download/ENCFF001WMM.bed.gz
https://www.encodeproject.org/files/ENCFF535GNR/@@download/ENCFF535GNR.bed.gz
https://www.encodeproject.org/files/ENCFF749ZBH/@@download/ENCFF749ZBH.bed.gz
https://www.encodeproject.org/files/ENCFF248RWN/@@download/ENCFF248RWN.bed.gz
https://www.encodeproject.org/files/ENCFF915CHV/@@download/ENCFF915CHV.bed.gz
https://www.encodeproject.org/files/ENCFF072PDN/@@download/ENCFF072PDN.bed.gz
https://www.encodeproject.org/files/ENCFF198QHH/@@download/ENCFF198QHH.bed.gz
https://www.encodeproject.org/files/ENCFF774CBZ/@@download/ENCFF774CBZ.bed.gz
https://www.encodeproject.org/files/ENCFF914SEH/@@download/ENCFF914SEH.bed.gz
https://www.encodeproject.org/files/ENCFF968ISS/@@download/ENCFF968ISS.bed.gz

Supplementary Table 1

https://www.encodeproject.org/files/ENCFF458PCJ/@@download/ENCFF458PCJ.bed.gz
https://www.encodeproject.org/files/ENCFF249VMA/@@download/ENCFF249VMA.bed.gz
https://www.encodeproject.org/files/ENCFF248PIL/@@download/ENCFF248PIL.bed.gz
https://www.encodeproject.org/files/ENCFF521DDI/@@download/ENCFF521DDI.bed.gz
https://www.encodeproject.org/files/ENCFF038VGE/@@download/ENCFF038VGE.bed.gz
https://www.encodeproject.org/files/ENCFF665BXP/@@download/ENCFF665BXP.bed.gz
https://www.encodeproject.org/files/ENCFF196ENU/@@download/ENCFF196ENU.bed.gz
https://www.encodeproject.org/files/ENCFF875CDR/@@download/ENCFF875CDR.bed.gz
https://www.encodeproject.org/files/ENCFF384YXE/@@download/ENCFF384YXE.bed.gz
https://www.encodeproject.org/files/ENCFF001UVP/@@download/ENCFF001UVP.bed.gz
https://www.encodeproject.org/files/ENCFF225UJM/@@download/ENCFF225UJM.bed.gz
https://www.encodeproject.org/files/ENCFF892RWC/@@download/ENCFF892RWC.bed.gz
https://www.encodeproject.org/files/ENCFF998JKA/@@download/ENCFF998JKA.bed.gz
https://www.encodeproject.org/files/ENCFF366TAR/@@download/ENCFF366TAR.bed.gz
https://www.encodeproject.org/files/ENCFF724PVV/@@download/ENCFF724PVV.bed.gz
https://www.encodeproject.org/files/ENCFF360ZAH/@@download/ENCFF360ZAH.bed.gz
https://www.encodeproject.org/files/ENCFF876KEA/@@download/ENCFF876KEA.bed.gz
https://www.encodeproject.org/files/ENCFF597KNJ/@@download/ENCFF597KNJ.bed.gz
https://www.encodeproject.org/files/ENCFF770BXK/@@download/ENCFF770BXK.bed.gz
https://www.encodeproject.org/files/ENCFF354AEP/@@download/ENCFF354AEP.bed.gz
https://www.encodeproject.org/files/ENCFF133XTG/@@download/ENCFF133XTG.bed.gz
https://www.encodeproject.org/files/ENCFF330NPA/@@download/ENCFF330NPA.bed.gz
https://www.encodeproject.org/files/ENCFF931DUC/@@download/ENCFF931DUC.bed.gz
https://www.encodeproject.org/files/ENCFF808QZI/@@download/ENCFF808QZI.bed.gz
https://www.encodeproject.org/files/ENCFF417IJL/@@download/ENCFF417IJL.bed.gz
https://www.encodeproject.org/files/ENCFF785PSO/@@download/ENCFF785PSO.bed.gz
https://www.encodeproject.org/files/ENCFF122HLI/@@download/ENCFF122HLI.bed.gz
https://www.encodeproject.org/files/ENCFF478XDH/@@download/ENCFF478XDH.bed.gz
https://www.encodeproject.org/files/ENCFF072SZT/@@download/ENCFF072SZT.bed.gz
https://www.encodeproject.org/files/ENCFF258LMH/@@download/ENCFF258LMH.bed.gz
https://www.encodeproject.org/files/ENCFF731WZI/@@download/ENCFF731WZI.bed.gz
https://www.encodeproject.org/files/ENCFF617UHG/@@download/ENCFF617UHG.bed.gz
https://www.encodeproject.org/files/ENCFF604AQG/@@download/ENCFF604AQG.bed.gz
https://www.encodeproject.org/files/ENCFF046QJY/@@download/ENCFF046QJY.bed.gz
https://www.encodeproject.org/files/ENCFF868NGL/@@download/ENCFF868NGL.bed.gz
https://www.encodeproject.org/files/ENCFF299FPS/@@download/ENCFF299FPS.bed.gz
https://www.encodeproject.org/files/ENCFF065SSZ/@@download/ENCFF065SSZ.bed.gz
https://www.encodeproject.org/files/ENCFF584LZP/@@download/ENCFF584LZP.bed.gz
https://www.encodeproject.org/files/ENCFF614SOO/@@download/ENCFF614SOO.bed.gz
https://www.encodeproject.org/files/ENCFF694IEM/@@download/ENCFF694IEM.bed.gz
https://www.encodeproject.org/files/ENCFF667KDW/@@download/ENCFF667KDW.bed.gz
https://www.encodeproject.org/files/ENCFF460ZAB/@@download/ENCFF460ZAB.bed.gz
https://www.encodeproject.org/files/ENCFF735DNU/@@download/ENCFF735DNU.bed.gz
https://www.encodeproject.org/files/ENCFF284MUO/@@download/ENCFF284MUO.bed.gz
https://www.encodeproject.org/files/ENCFF106EDT/@@download/ENCFF106EDT.bed.gz
https://www.encodeproject.org/files/ENCFF113VXK/@@download/ENCFF113VXK.bed.gz
https://www.encodeproject.org/files/ENCFF407RMN/@@download/ENCFF407RMN.bed.gz
https://www.encodeproject.org/files/ENCFF261CWP/@@download/ENCFF261CWP.bed.gz
https://www.encodeproject.org/files/ENCFF001SRN/@@download/ENCFF001SRN.bed.gz
https://www.encodeproject.org/files/ENCFF001WAS/@@download/ENCFF001WAS.bed.gz
https://www.encodeproject.org/files/ENCFF001WLS/@@download/ENCFF001WLS.bed.gz
https://www.encodeproject.org/files/ENCFF001WLR/@@download/ENCFF001WLR.bed.gz
https://www.encodeproject.org/files/ENCFF444HFG/@@download/ENCFF444HFG.bed.gz

Supplementary Table 1

https://www.encodeproject.org/files/ENCFF367GII/@@download/ENCFF367GII.bed.gz
https://www.encodeproject.org/files/ENCFF620HKT/@@download/ENCFF620HKT.bed.gz
https://www.encodeproject.org/files/ENCFF089IWT/@@download/ENCFF089IWT.bed.gz
https://www.encodeproject.org/files/ENCFF460FCG/@@download/ENCFF460FCG.bed.gz
https://www.encodeproject.org/files/ENCFF722GNI/@@download/ENCFF722GNI.bed.gz
https://www.encodeproject.org/files/ENCFF164UXI/@@download/ENCFF164UXI.bed.gz
https://www.encodeproject.org/files/ENCFF410GYO/@@download/ENCFF410GYO.bed.gz
https://www.encodeproject.org/files/ENCFF482JEI/@@download/ENCFF482JEI.bed.gz
https://www.encodeproject.org/files/ENCFF718RIM/@@download/ENCFF718RIM.bed.gz
https://www.encodeproject.org/files/ENCFF577XLP/@@download/ENCFF577XLP.bed.gz
https://www.encodeproject.org/files/ENCFF876SNH/@@download/ENCFF876SNH.bed.gz
https://www.encodeproject.org/files/ENCFF617UGK/@@download/ENCFF617UGK.bed.gz
https://www.encodeproject.org/files/ENCFF205KSG/@@download/ENCFF205KSG.bed.gz
https://www.encodeproject.org/files/ENCFF085NOG/@@download/ENCFF085NOG.bed.gz
https://www.encodeproject.org/files/ENCFF582VIH/@@download/ENCFF582VIH.bed.gz
https://www.encodeproject.org/files/ENCFF566XHO/@@download/ENCFF566XHO.bed.gz
https://www.encodeproject.org/files/ENCFF532RQO/@@download/ENCFF532RQO.bed.gz
https://www.encodeproject.org/files/ENCFF126VBD/@@download/ENCFF126VBD.bed.gz
https://www.encodeproject.org/files/ENCFF690BPH/@@download/ENCFF690BPH.bed.gz
https://www.encodeproject.org/files/ENCFF001UYT/@@download/ENCFF001UYT.bed.gz
https://www.encodeproject.org/files/ENCFF001WHH/@@download/ENCFF001WHH.bed.gz
https://www.encodeproject.org/files/ENCFF867BKX/@@download/ENCFF867BKX.bed.gz
https://www.encodeproject.org/files/ENCFF972QQK/@@download/ENCFF972QQK.bed.gz
https://www.encodeproject.org/files/ENCFF687ZRR/@@download/ENCFF687ZRR.bed.gz
https://www.encodeproject.org/files/ENCFF702ROS/@@download/ENCFF702ROS.bed.gz
https://www.encodeproject.org/files/ENCFF630OEM/@@download/ENCFF630OEM.bed.gz
https://www.encodeproject.org/files/ENCFF300ZIA/@@download/ENCFF300ZIA.bed.gz
https://www.encodeproject.org/files/ENCFF001SRH/@@download/ENCFF001SRH.bed.gz
https://www.encodeproject.org/files/ENCFF001WKV/@@download/ENCFF001WKV.bed.gz
https://www.encodeproject.org/files/ENCFF001WKW/@@download/ENCFF001WKW.bed.gz
https://www.encodeproject.org/files/ENCFF238TWJ/@@download/ENCFF238TWJ.bed.gz
https://www.encodeproject.org/files/ENCFF025EDR/@@download/ENCFF025EDR.bed.gz
https://www.encodeproject.org/files/ENCFF823QRP/@@download/ENCFF823QRP.bed.gz
https://www.encodeproject.org/files/ENCFF084LGH/@@download/ENCFF084LGH.bed.gz
https://www.encodeproject.org/files/ENCFF123PWM/@@download/ENCFF123PWM.bed.gz
https://www.encodeproject.org/files/ENCFF468SYR/@@download/ENCFF468SYR.bed.gz
https://www.encodeproject.org/files/ENCFF350ZQV/@@download/ENCFF350ZQV.bed.gz
https://www.encodeproject.org/files/ENCFF469VYN/@@download/ENCFF469VYN.bed.gz
https://www.encodeproject.org/files/ENCFF876KVN/@@download/ENCFF876KVN.bed.gz
https://www.encodeproject.org/files/ENCFF025ZQZ/@@download/ENCFF025ZQZ.bed.gz
https://www.encodeproject.org/files/ENCFF335TGM/@@download/ENCFF335TGM.bed.gz
https://www.encodeproject.org/files/ENCFF558GRZ/@@download/ENCFF558GRZ.bed.gz
https://www.encodeproject.org/files/ENCFF001SPH/@@download/ENCFF001SPH.bed.gz
https://www.encodeproject.org/files/ENCFF001UXD/@@download/ENCFF001UXD.bed.gz
https://www.encodeproject.org/files/ENCFF075MQY/@@download/ENCFF075MQY.bed.gz
https://www.encodeproject.org/files/ENCFF166RZB/@@download/ENCFF166RZB.bed.gz
https://www.encodeproject.org/files/ENCFF233CGZ/@@download/ENCFF233CGZ.bed.gz
https://www.encodeproject.org/files/ENCFF656LEP/@@download/ENCFF656LEP.bed.gz
https://www.encodeproject.org/files/ENCFF977OWF/@@download/ENCFF977OWF.bed.gz
https://www.encodeproject.org/files/ENCFF179WUI/@@download/ENCFF179WUI.bed.gz
https://www.encodeproject.org/files/ENCFF395QJV/@@download/ENCFF395QJV.bed.gz
https://www.encodeproject.org/files/ENCFF057GAH/@@download/ENCFF057GAH.bed.gz
https://www.encodeproject.org/files/ENCFF001UUH/@@download/ENCFF001UUH.bed.gz

https://www.encodeproject.org/files/ENCFF001UUX/@@download/ENCFF001UUX.bed.gz
https://www.encodeproject.org/files/ENCFF631HBT/@@download/ENCFF631HBT.bed.gz
https://www.encodeproject.org/files/ENCFF611EHQ/@@download/ENCFF611EHQ.bed.gz
https://www.encodeproject.org/files/ENCFF906RLV/@@download/ENCFF906RLV.bed.gz
https://www.encodeproject.org/files/ENCFF101NVD/@@download/ENCFF101NVD.bed.gz
https://www.encodeproject.org/files/ENCFF483BPG/@@download/ENCFF483BPG.bed.gz
https://www.encodeproject.org/files/ENCFF840NPU/@@download/ENCFF840NPU.bed.gz
https://www.encodeproject.org/files/ENCFF104NYW/@@download/ENCFF104NYW.bed.gz
https://www.encodeproject.org/files/ENCFF860LAZ/@@download/ENCFF860LAZ.bed.gz
https://www.encodeproject.org/files/ENCFF001SQN/@@download/ENCFF001SQN.bed.gz
https://www.encodeproject.org/files/ENCFF001VZK/@@download/ENCFF001VZK.bed.gz
https://www.encodeproject.org/files/ENCFF001WHC/@@download/ENCFF001WHC.bed.gz
https://www.encodeproject.org/files/ENCFF001WHB/@@download/ENCFF001WHB.bed.gz
https://www.encodeproject.org/files/ENCFF639MUE/@@download/ENCFF639MUE.bed.gz
https://www.encodeproject.org/files/ENCFF607WIZ/@@download/ENCFF607WIZ.bed.gz
https://www.encodeproject.org/files/ENCFF674LBB/@@download/ENCFF674LBB.bed.gz
https://www.encodeproject.org/files/ENCFF452BSD/@@download/ENCFF452BSD.bed.gz
https://www.encodeproject.org/files/ENCFF973CLH/@@download/ENCFF973CLH.bed.gz
https://www.encodeproject.org/files/ENCFF614ZJX/@@download/ENCFF614ZJX.bed.gz
https://www.encodeproject.org/files/ENCFF628TOP/@@download/ENCFF628TOP.bed.gz
https://www.encodeproject.org/files/ENCFF518IUS/@@download/ENCFF518IUS.bed.gz
https://www.encodeproject.org/files/ENCFF531YLQ/@@download/ENCFF531YLQ.bed.gz
https://www.encodeproject.org/files/ENCFF473ELQ/@@download/ENCFF473ELQ.bed.gz
https://www.encodeproject.org/files/ENCFF191VKY/@@download/ENCFF191VKY.bed.gz
https://www.encodeproject.org/files/ENCFF041XBL/@@download/ENCFF041XBL.bed.gz
https://www.encodeproject.org/files/ENCFF351STM/@@download/ENCFF351STM.bed.gz
https://www.encodeproject.org/files/ENCFF734YEQ/@@download/ENCFF734YEQ.bed.gz
https://www.encodeproject.org/files/ENCFF272RMN/@@download/ENCFF272RMN.bed.gz
https://www.encodeproject.org/files/ENCFF428HAF/@@download/ENCFF428HAF.bed.gz
https://www.encodeproject.org/files/ENCFF618KEX/@@download/ENCFF618KEX.bed.gz
https://www.encodeproject.org/files/ENCFF132BIV/@@download/ENCFF132BIV.bed.gz
https://www.encodeproject.org/files/ENCFF648KWJ/@@download/ENCFF648KWJ.bed.gz
https://www.encodeproject.org/files/ENCFF802SNU/@@download/ENCFF802SNU.bed.gz
https://www.encodeproject.org/files/ENCFF691MJO/@@download/ENCFF691MJO.bed.gz
https://www.encodeproject.org/files/ENCFF782KTL/@@download/ENCFF782KTL.bed.gz
https://www.encodeproject.org/files/ENCFF003GPD/@@download/ENCFF003GPD.bed.gz
https://www.encodeproject.org/files/ENCFF755CVA/@@download/ENCFF755CVA.bed.gz
https://www.encodeproject.org/files/ENCFF574WIN/@@download/ENCFF574WIN.bed.gz
https://www.encodeproject.org/files/ENCFF404WEO/@@download/ENCFF404WEO.bed.gz
https://www.encodeproject.org/files/ENCFF763KLI/@@download/ENCFF763KLI.bed.gz
https://www.encodeproject.org/files/ENCFF312KVV/@@download/ENCFF312KVV.bed.gz
https://www.encodeproject.org/files/ENCFF130CZB/@@download/ENCFF130CZB.bed.gz
https://www.encodeproject.org/files/ENCFF564SMU/@@download/ENCFF564SMU.bed.gz
https://www.encodeproject.org/files/ENCFF515IOM/@@download/ENCFF515IOM.bed.gz
https://www.encodeproject.org/files/ENCFF978III/@@download/ENCFF978III.bed.gz
https://www.encodeproject.org/files/ENCFF067OIC/@@download/ENCFF067OIC.bed.gz
https://www.encodeproject.org/files/ENCFF075LFT/@@download/ENCFF075LFT.bed.gz
https://www.encodeproject.org/files/ENCFF001SRK/@@download/ENCFF001SRK.bed.gz
https://www.encodeproject.org/files/ENCFF001WLH/@@download/ENCFF001WLH.bed.gz
https://www.encodeproject.org/files/ENCFF001WLI/@@download/ENCFF001WLI.bed.gz
https://www.encodeproject.org/files/ENCFF240YXY/@@download/ENCFF240YXY.bed.gz
https://www.encodeproject.org/files/ENCFF766JVH/@@download/ENCFF766JVH.bed.gz
https://www.encodeproject.org/files/ENCFF105ETE/@@download/ENCFF105ETE.bed.gz

Supplementary Table 1

https://www.encodeproject.org/files/ENCFF699EKS/@@download/ENCFF699EKS.bed.gz
https://www.encodeproject.org/files/ENCFF833AYH/@@download/ENCFF833AYH.bed.gz
https://www.encodeproject.org/files/ENCFF103UFM/@@download/ENCFF103UFM.bed.gz
https://www.encodeproject.org/files/ENCFF787YHW/@@download/ENCFF787YHW.bed.gz
https://www.encodeproject.org/files/ENCFF491VQA/@@download/ENCFF491VQA.bed.gz
https://www.encodeproject.org/files/ENCFF454ETI/@@download/ENCFF454ETI.bed.gz
https://www.encodeproject.org/files/ENCFF998ZTZ/@@download/ENCFF998ZTZ.bed.gz
https://www.encodeproject.org/files/ENCFF922CTF/@@download/ENCFF922CTF.bed.gz
https://www.encodeproject.org/files/ENCFF583KFI/@@download/ENCFF583KFI.bed.gz
https://www.encodeproject.org/files/ENCFF035RNU/@@download/ENCFF035RNU.bed.gz
https://www.encodeproject.org/files/ENCFF718CUQ/@@download/ENCFF718CUQ.bed.gz
https://www.encodeproject.org/files/ENCFF971VCD/@@download/ENCFF971VCD.bed.gz
https://www.encodeproject.org/files/ENCFF654WPG/@@download/ENCFF654WPG.bed.gz
https://www.encodeproject.org/files/ENCFF114MYB/@@download/ENCFF114MYB.bed.gz
https://www.encodeproject.org/files/ENCFF306COA/@@download/ENCFF306COA.bed.gz
https://www.encodeproject.org/files/ENCFF925JSO/@@download/ENCFF925JSO.bed.gz
https://www.encodeproject.org/files/ENCFF778MGI/@@download/ENCFF778MGI.bed.gz
https://www.encodeproject.org/files/ENCFF405IIV/@@download/ENCFF405IIV.bed.gz
https://www.encodeproject.org/files/ENCFF392JHV/@@download/ENCFF392JHV.bed.gz
https://www.encodeproject.org/files/ENCFF533MFM/@@download/ENCFF533MFM.bed.gz
https://www.encodeproject.org/files/ENCFF855CMV/@@download/ENCFF855CMV.bed.gz
https://www.encodeproject.org/files/ENCFF317XWF/@@download/ENCFF317XWF.bed.gz
https://www.encodeproject.org/files/ENCFF869AYK/@@download/ENCFF869AYK.bed.gz
https://www.encodeproject.org/files/ENCFF680BZR/@@download/ENCFF680BZR.bed.gz
https://www.encodeproject.org/files/ENCFF134EZN/@@download/ENCFF134EZN.bed.gz
https://www.encodeproject.org/files/ENCFF895VHC/@@download/ENCFF895VHC.bed.gz
https://www.encodeproject.org/files/ENCFF205GHG/@@download/ENCFF205GHG.bed.gz
https://www.encodeproject.org/files/ENCFF398PRG/@@download/ENCFF398PRG.bed.gz
https://www.encodeproject.org/files/ENCFF841UKC/@@download/ENCFF841UKC.bed.gz
https://www.encodeproject.org/files/ENCFF019PSW/@@download/ENCFF019PSW.bed.gz
https://www.encodeproject.org/files/ENCFF442AYJ/@@download/ENCFF442AYJ.bed.gz
https://www.encodeproject.org/files/ENCFF596AFS/@@download/ENCFF596AFS.bed.gz
https://www.encodeproject.org/files/ENCFF114CIS/@@download/ENCFF114CIS.bed.gz
https://www.encodeproject.org/files/ENCFF211VKM/@@download/ENCFF211VKM.bed.gz
https://www.encodeproject.org/files/ENCFF305FTY/@@download/ENCFF305FTY.bed.gz
https://www.encodeproject.org/files/ENCFF852OUW/@@download/ENCFF852OUW.bed.gz
https://www.encodeproject.org/files/ENCFF695PHQ/@@download/ENCFF695PHQ.bed.gz
https://www.encodeproject.org/files/ENCFF520MND/@@download/ENCFF520MND.bed.gz
https://www.encodeproject.org/files/ENCFF316AXF/@@download/ENCFF316AXF.bed.gz
https://www.encodeproject.org/files/ENCFF879CGC/@@download/ENCFF879CGC.bed.gz
https://www.encodeproject.org/files/ENCFF401BPQ/@@download/ENCFF401BPQ.bed.gz
https://www.encodeproject.org/files/ENCFF080POQ/@@download/ENCFF080POQ.bed.gz
https://www.encodeproject.org/files/ENCFF671CWO/@@download/ENCFF671CWO.bed.gz
https://www.encodeproject.org/files/ENCFF945DQY/@@download/ENCFF945DQY.bed.gz
https://www.encodeproject.org/files/ENCFF544CVM/@@download/ENCFF544CVM.bed.gz
https://www.encodeproject.org/files/ENCFF341HAK/@@download/ENCFF341HAK.bed.gz
https://www.encodeproject.org/files/ENCFF687BUW/@@download/ENCFF687BUW.bed.gz
https://www.encodeproject.org/files/ENCFF837PDZ/@@download/ENCFF837PDZ.bed.gz
https://www.encodeproject.org/files/ENCFF784HLE/@@download/ENCFF784HLE.bed.gz
https://www.encodeproject.org/files/ENCFF849KDW/@@download/ENCFF849KDW.bed.gz
https://www.encodeproject.org/files/ENCFF609BOM/@@download/ENCFF609BOM.bed.gz
https://www.encodeproject.org/files/ENCFF502POZ/@@download/ENCFF502POZ.bed.gz
https://www.encodeproject.org/files/ENCFF421EQO/@@download/ENCFF421EQO.bed.gz

Supplementary Table 1

https://www.encodeproject.org/files/ENCFF120MQU/@@download/ENCFF120MQU.bed.gz
https://www.encodeproject.org/files/ENCFF484CEH/@@download/ENCFF484CEH.bed.gz
https://www.encodeproject.org/files/ENCFF172LNK/@@download/ENCFF172LNK.bed.gz
https://www.encodeproject.org/files/ENCFF203HVV/@@download/ENCFF203HVV.bed.gz
https://www.encodeproject.org/files/ENCFF919NRH/@@download/ENCFF919NRH.bed.gz
https://www.encodeproject.org/files/ENCFF721LXU/@@download/ENCFF721LXU.bed.gz
https://www.encodeproject.org/files/ENCFF727SUT/@@download/ENCFF727SUT.bed.gz
https://www.encodeproject.org/files/ENCFF284FOE/@@download/ENCFF284FOE.bed.gz
https://www.encodeproject.org/files/ENCFF607XRM/@@download/ENCFF607XRM.bed.gz
https://www.encodeproject.org/files/ENCFF001SQD/@@download/ENCFF001SQD.bed.gz
https://www.encodeproject.org/files/ENCFF001VYW/@@download/ENCFF001VYW.bed.gz
https://www.encodeproject.org/files/ENCFF001WEQ/@@download/ENCFF001WEQ.bed.gz
https://www.encodeproject.org/files/ENCFF001WEP/@@download/ENCFF001WEP.bed.gz
https://www.encodeproject.org/files/ENCFF541VKE/@@download/ENCFF541VKE.bed.gz
https://www.encodeproject.org/files/ENCFF841XRI/@@download/ENCFF841XRI.bed.gz
https://www.encodeproject.org/files/ENCFF581UTE/@@download/ENCFF581UTE.bed.gz
https://www.encodeproject.org/files/ENCFF263YRO/@@download/ENCFF263YRO.bed.gz
https://www.encodeproject.org/files/ENCFF001SRP/@@download/ENCFF001SRP.bed.gz
https://www.encodeproject.org/files/ENCFF001WAW/@@download/ENCFF001WAW.bed.gz
https://www.encodeproject.org/files/ENCFF001WMA/@@download/ENCFF001WMA.bed.gz
https://www.encodeproject.org/files/ENCFF001WLZ/@@download/ENCFF001WLZ.bed.gz
https://www.encodeproject.org/files/ENCFF551MUC/@@download/ENCFF551MUC.bed.gz
https://www.encodeproject.org/files/ENCFF542SLO/@@download/ENCFF542SLO.bed.gz
https://www.encodeproject.org/files/ENCFF659MOZ/@@download/ENCFF659MOZ.bed.gz
https://www.encodeproject.org/files/ENCFF583YED/@@download/ENCFF583YED.bed.gz
https://www.encodeproject.org/files/ENCFF226GFM/@@download/ENCFF226GFM.bed.gz
https://www.encodeproject.org/files/ENCFF185SDR/@@download/ENCFF185SDR.bed.gz
https://www.encodeproject.org/files/ENCFF914XCX/@@download/ENCFF914XCX.bed.gz
https://www.encodeproject.org/files/ENCFF571LZB/@@download/ENCFF571LZB.bed.gz
https://www.encodeproject.org/files/ENCFF008MTK/@@download/ENCFF008MTK.bed.gz
https://www.encodeproject.org/files/ENCFF949MOE/@@download/ENCFF949MOE.bed.gz
https://www.encodeproject.org/files/ENCFF694GPP/@@download/ENCFF694GPP.bed.gz
https://www.encodeproject.org/files/ENCFF613SZK/@@download/ENCFF613SZK.bed.gz
https://www.encodeproject.org/files/ENCFF831GIV/@@download/ENCFF831GIV.bed.gz
https://www.encodeproject.org/files/ENCFF055ZSF/@@download/ENCFF055ZSF.bed.gz
https://www.encodeproject.org/files/ENCFF964WXZ/@@download/ENCFF964WXZ.bed.gz
https://www.encodeproject.org/files/ENCFF885DOB/@@download/ENCFF885DOB.bed.gz
https://www.encodeproject.org/files/ENCFF228ZTQ/@@download/ENCFF228ZTQ.bed.gz
https://www.encodeproject.org/files/ENCFF011JIY/@@download/ENCFF011JIY.bed.gz
https://www.encodeproject.org/files/ENCFF619IZA/@@download/ENCFF619IZA.bed.gz
https://www.encodeproject.org/files/ENCFF139VWG/@@download/ENCFF139VWG.bed.gz
https://www.encodeproject.org/files/ENCFF747RZI/@@download/ENCFF747RZI.bed.gz
https://www.encodeproject.org/files/ENCFF789TOW/@@download/ENCFF789TOW.bed.gz
https://www.encodeproject.org/files/ENCFF982KOJ/@@download/ENCFF982KOJ.bed.gz
https://www.encodeproject.org/files/ENCFF412SKC/@@download/ENCFF412SKC.bed.gz
https://www.encodeproject.org/files/ENCFF661OGW/@@download/ENCFF661OGW.bed.gz
https://www.encodeproject.org/files/ENCFF193AML/@@download/ENCFF193AML.bed.gz
https://www.encodeproject.org/files/ENCFF031RMC/@@download/ENCFF031RMC.bed.gz
https://www.encodeproject.org/files/ENCFF630VNL/@@download/ENCFF630VNL.bed.gz
https://www.encodeproject.org/files/ENCFF664MRC/@@download/ENCFF664MRC.bed.gz
https://www.encodeproject.org/files/ENCFF207RZS/@@download/ENCFF207RZS.bed.gz
https://www.encodeproject.org/files/ENCFF145MNB/@@download/ENCFF145MNB.bed.gz
https://www.encodeproject.org/files/ENCFF039KHZ/@@download/ENCFF039KHZ.bed.gz

https://www.encodeproject.org/files/ENCFF383OJO/@@download/ENCFF383OJO.bed.gz
https://www.encodeproject.org/files/ENCFF562NMD/@@download/ENCFF562NMD.bed.gz
https://www.encodeproject.org/files/ENCFF393RPX/@@download/ENCFF393RPX.bed.gz
https://www.encodeproject.org/files/ENCFF884KLO/@@download/ENCFF884KLO.bed.gz
https://www.encodeproject.org/files/ENCFF867VFF/@@download/ENCFF867VFF.bed.gz
https://www.encodeproject.org/files/ENCFF588RBG/@@download/ENCFF588RBG.bed.gz
https://www.encodeproject.org/files/ENCFF698QNG/@@download/ENCFF698QNG.bed.gz
https://www.encodeproject.org/files/ENCFF497QQN/@@download/ENCFF497QQN.bed.gz
https://www.encodeproject.org/files/ENCFF932ATD/@@download/ENCFF932ATD.bed.gz
https://www.encodeproject.org/files/ENCFF729VHV/@@download/ENCFF729VHV.bed.gz
https://www.encodeproject.org/files/ENCFF880ORV/@@download/ENCFF880ORV.bed.gz
https://www.encodeproject.org/files/ENCFF566SFJ/@@download/ENCFF566SFJ.bed.gz
https://www.encodeproject.org/files/ENCFF967WVU/@@download/ENCFF967WVU.bed.gz
https://www.encodeproject.org/files/ENCFF955EKA/@@download/ENCFF955EKA.bed.gz
https://www.encodeproject.org/files/ENCFF628MPB/@@download/ENCFF628MPB.bed.gz
https://www.encodeproject.org/files/ENCFF344IND/@@download/ENCFF344IND.bed.gz
https://www.encodeproject.org/files/ENCFF929EGO/@@download/ENCFF929EGO.bed.gz
https://www.encodeproject.org/files/ENCFF257MHT/@@download/ENCFF257MHT.bed.gz
https://www.encodeproject.org/files/ENCFF927NNT/@@download/ENCFF927NNT.bed.gz
https://www.encodeproject.org/files/ENCFF079RNM/@@download/ENCFF079RNM.bed.gz
https://www.encodeproject.org/files/ENCFF001SSF/@@download/ENCFF001SSF.bed.gz
https://www.encodeproject.org/files/ENCFF001WCE/@@download/ENCFF001WCE.bed.gz
https://www.encodeproject.org/files/ENCFF001WSK/@@download/ENCFF001WSK.bed.gz
https://www.encodeproject.org/files/ENCFF001WSJ/@@download/ENCFF001WSJ.bed.gz
https://www.encodeproject.org/files/ENCFF587IAH/@@download/ENCFF587IAH.bed.gz
https://www.encodeproject.org/files/ENCFF741URZ/@@download/ENCFF741URZ.bed.gz
https://www.encodeproject.org/files/ENCFF546QUZ/@@download/ENCFF546QUZ.bed.gz
https://www.encodeproject.org/files/ENCFF338GII/@@download/ENCFF338GII.bed.gz
https://www.encodeproject.org/files/ENCFF243PXZ/@@download/ENCFF243PXZ.bed.gz
https://www.encodeproject.org/files/ENCFF061UXT/@@download/ENCFF061UXT.bed.gz
https://www.encodeproject.org/files/ENCFF060OQR/@@download/ENCFF060OQR.bed.gz
https://www.encodeproject.org/files/ENCFF062NOL/@@download/ENCFF062NOL.bed.gz
https://www.encodeproject.org/files/ENCFF953EHW/@@download/ENCFF953EHW.bed.gz
https://www.encodeproject.org/files/ENCFF944CUY/@@download/ENCFF944CUY.bed.gz
https://www.encodeproject.org/files/ENCFF595OHJ/@@download/ENCFF595OHJ.bed.gz
https://www.encodeproject.org/files/ENCFF251CYD/@@download/ENCFF251CYD.bed.gz
https://www.encodeproject.org/files/ENCFF262NZB/@@download/ENCFF262NZB.bed.gz
https://www.encodeproject.org/files/ENCFF715BDS/@@download/ENCFF715BDS.bed.gz
https://www.encodeproject.org/files/ENCFF033JMX/@@download/ENCFF033JMX.bed.gz
https://www.encodeproject.org/files/ENCFF310RJD/@@download/ENCFF310RJD.bed.gz
https://www.encodeproject.org/files/ENCFF485DEP/@@download/ENCFF485DEP.bed.gz
https://www.encodeproject.org/files/ENCFF520FBI/@@download/ENCFF520FBI.bed.gz
https://www.encodeproject.org/files/ENCFF631NNC/@@download/ENCFF631NNC.bed.gz
https://www.encodeproject.org/files/ENCFF197ZNE/@@download/ENCFF197ZNE.bed.gz
https://www.encodeproject.org/files/ENCFF392EHA/@@download/ENCFF392EHA.bed.gz
https://www.encodeproject.org/files/ENCFF271AGL/@@download/ENCFF271AGL.bed.gz
https://www.encodeproject.org/files/ENCFF484YOE/@@download/ENCFF484YOE.bed.gz
https://www.encodeproject.org/files/ENCFF663LIJ/@@download/ENCFF663LIJ.bed.gz
https://www.encodeproject.org/files/ENCFF177TMO/@@download/ENCFF177TMO.bed.gz
https://www.encodeproject.org/files/ENCFF610OWH/@@download/ENCFF610OWH.bed.gz
https://www.encodeproject.org/files/ENCFF543NKS/@@download/ENCFF543NKS.bed.gz
https://www.encodeproject.org/files/ENCFF425BBF/@@download/ENCFF425BBF.bed.gz
https://www.encodeproject.org/files/ENCFF001WCW/@@download/ENCFF001WCW.bed.gz

Supplementary Table 1

https://www.encodeproject.org/files/ENCFF001WTU/@@download/ENCFF001WTU.bed.gz

https://www.encodeproject.org/files/ENCFF027ARL/@@download/ENCFF027ARL.bed.gz

https://www.encodeproject.org/files/ENCFF852NHM/@@download/ENCFF852NHM.bed.gz

https://www.encodeproject.org/files/ENCFF089JTD/@@download/ENCFF089JTD.bed.gz

https://www.encodeproject.org/files/ENCFF744IFV/@@download/ENCFF744IFV.bed.gz

https://www.encodeproject.org/files/ENCFF475HWF/@@download/ENCFF475HWF.bed.gz

https://www.encodeproject.org/files/ENCFF752PFI/@@download/ENCFF752PFI.bed.gz

https://www.encodeproject.org/files/ENCFF907ODQ/@@download/ENCFF907ODQ.bed.gz

https://www.encodeproject.org/files/ENCFF365DQA/@@download/ENCFF365DQA.bed.gz

https://www.encodeproject.org/files/ENCFF067VHZ/@@download/ENCFF067VHZ.bed.gz

https://www.encodeproject.org/files/ENCFF319CPJ/@@download/ENCFF319CPJ.bed.gz

https://www.encodeproject.org/files/ENCFF944HYF/@@download/ENCFF944HYF.bed.gz

https://www.encodeproject.org/files/ENCFF907IIM/@@download/ENCFF907IIM.bed.gz

https://www.encodeproject.org/files/ENCFF698RYJ/@@download/ENCFF698RYJ.bed.gz

https://www.encodeproject.org/files/ENCFF722BUK/@@download/ENCFF722BUK.bed.gz

https://www.encodeproject.org/files/ENCFF771EKC/@@download/ENCFF771EKC.bed.gz

https://www.encodeproject.org/files/ENCFF099MBG/@@download/ENCFF099MBG.bed.gz

https://www.encodeproject.org/files/ENCFF165LZK/@@download/ENCFF165LZK.bed.gz

https://www.encodeproject.org/files/ENCFF054VNO/@@download/ENCFF054VNO.bed.gz

https://www.encodeproject.org/files/ENCFF926KAT/@@download/ENCFF926KAT.bed.gz

https://www.encodeproject.org/files/ENCFF621YFD/@@download/ENCFF621YFD.bed.gz

https://www.encodeproject.org/files/ENCFF510UYU/@@download/ENCFF510UYU.bed.gz

https://www.encodeproject.org/files/ENCFF388FUA/@@download/ENCFF388FUA.bed.gz

https://www.encodeproject.org/files/ENCFF664WUJ/@@download/ENCFF664WUJ.bed.gz

https://www.encodeproject.org/files/ENCFF646LUA/@@download/ENCFF646LUA.bed.gz

https://www.encodeproject.org/files/ENCFF799KWG/@@download/ENCFF799KWG.bed.gz

https://www.encodeproject.org/files/ENCFF206FZJ/@@download/ENCFF206FZJ.bed.gz

https://www.encodeproject.org/files/ENCFF048ZGK/@@download/ENCFF048ZGK.bed.gz

https://www.encodeproject.org/files/ENCFF378BUF/@@download/ENCFF378BUF.bed.gz

https://www.encodeproject.org/files/ENCFF960LRA/@@download/ENCFF960LRA.bed.gz

https://www.encodeproject.org/files/ENCFF343YZV/@@download/ENCFF343YZV.bed.gz

https://www.encodeproject.org/files/ENCFF822UQG/@@download/ENCFF822UQG.bed.gz

https://www.encodeproject.org/files/ENCFF021ZDC/@@download/ENCFF021ZDC.bed.gz

https://www.encodeproject.org/files/ENCFF948DCR/@@download/ENCFF948DCR.bed.gz

https://www.encodeproject.org/files/ENCFF269BZQ/@@download/ENCFF269BZQ.bed.gz

https://www.encodeproject.org/files/ENCFF197UKJ/@@download/ENCFF197UKJ.bed.gz

https://www.encodeproject.org/files/ENCFF732XMH/@@download/ENCFF732XMH.bed.gz

https://www.encodeproject.org/files/ENCFF828CKH/@@download/ENCFF828CKH.bed.gz

https://www.encodeproject.org/files/ENCFF868YHB/@@download/ENCFF868YHB.bed.gz

https://www.encodeproject.org/files/ENCFF919FUV/@@download/ENCFF919FUV.bed.gz

https://www.encodeproject.org/files/ENCFF365IGK/@@download/ENCFF365IGK.bed.gz

https://www.encodeproject.org/files/ENCFF401NSY/@@download/ENCFF401NSY.bed.gz

https://www.encodeproject.org/files/ENCFF701RGI/@@download/ENCFF701RGI.bed.gz

https://www.encodeproject.org/files/ENCFF784TVL/@@download/ENCFF784TVL.bed.gz

https://www.encodeproject.org/files/ENCFF736ACF/@@download/ENCFF736ACF.bed.gz

https://www.encodeproject.org/files/ENCFF365RKF/@@download/ENCFF365RKF.bed.gz

https://www.encodeproject.org/files/ENCFF261BTV/@@download/ENCFF261BTV.bed.gz

https://www.encodeproject.org/files/ENCFF056LVP/@@download/ENCFF056LVP.bed.gz

https://www.encodeproject.org/files/ENCFF006GNP/@@download/ENCFF006GNP.bed.gz

https://www.encodeproject.org/files/ENCFF671KGS/@@download/ENCFF671KGS.bed.gz

https://www.encodeproject.org/files/ENCFF338PHE/@@download/ENCFF338PHE.bed.gz

https://www.encodeproject.org/files/ENCFF001SRR/@@download/ENCFF001SRR.bed.gz

https://www.encodeproject.org/files/ENCFF001WMH/@@download/ENCFF001WMH.bed.gz

Supplementary Table 1

https://www.encodeproject.org/files/ENCFF001WMI/@@download/ENCFF001WMI.bed.gz
https://www.encodeproject.org/files/ENCFF221ETZ/@@download/ENCFF221ETZ.bed.gz
https://www.encodeproject.org/files/ENCFF599YTD/@@download/ENCFF599YTD.bed.gz
https://www.encodeproject.org/files/ENCFF993TCA/@@download/ENCFF993TCA.bed.gz
https://www.encodeproject.org/files/ENCFF927KUT/@@download/ENCFF927KUT.bed.gz
https://www.encodeproject.org/files/ENCFF883FSZ/@@download/ENCFF883FSZ.bed.gz
https://www.encodeproject.org/files/ENCFF984PNP/@@download/ENCFF984PNP.bed.gz
https://www.encodeproject.org/files/ENCFF416APW/@@download/ENCFF416APW.bed.gz
https://www.encodeproject.org/files/ENCFF533QIZ/@@download/ENCFF533QIZ.bed.gz
https://www.encodeproject.org/files/ENCFF960EDC/@@download/ENCFF960EDC.bed.gz
https://www.encodeproject.org/files/ENCFF975RPX/@@download/ENCFF975RPX.bed.gz
https://www.encodeproject.org/files/ENCFF563UCE/@@download/ENCFF563UCE.bed.gz
https://www.encodeproject.org/files/ENCFF714JOW/@@download/ENCFF714JOW.bed.gz
https://www.encodeproject.org/files/ENCFF386GFZ/@@download/ENCFF386GFZ.bed.gz
https://www.encodeproject.org/files/ENCFF947VSW/@@download/ENCFF947VSW.bed.gz
https://www.encodeproject.org/files/ENCFF525ZZG/@@download/ENCFF525ZZG.bed.gz
https://www.encodeproject.org/files/ENCFF590XAP/@@download/ENCFF590XAP.bed.gz
https://www.encodeproject.org/files/ENCFF507PIV/@@download/ENCFF507PIV.bed.gz
https://www.encodeproject.org/files/ENCFF323LQF/@@download/ENCFF323LQF.bed.gz
https://www.encodeproject.org/files/ENCFF339GAX/@@download/ENCFF339GAX.bed.gz
https://www.encodeproject.org/files/ENCFF206SZY/@@download/ENCFF206SZY.bed.gz
https://www.encodeproject.org/files/ENCFF906KRB/@@download/ENCFF906KRB.bed.gz
https://www.encodeproject.org/files/ENCFF622RNC/@@download/ENCFF622RNC.bed.gz
https://www.encodeproject.org/files/ENCFF938LUA/@@download/ENCFF938LUA.bed.gz
https://www.encodeproject.org/files/ENCFF769UXX/@@download/ENCFF769UXX.bed.gz
https://www.encodeproject.org/files/ENCFF280ELK/@@download/ENCFF280ELK.bed.gz
https://www.encodeproject.org/files/ENCFF386RDS/@@download/ENCFF386RDS.bed.gz
https://www.encodeproject.org/files/ENCFF438XUQ/@@download/ENCFF438XUQ.bed.gz
https://www.encodeproject.org/files/ENCFF038OYK/@@download/ENCFF038OYK.bed.gz
https://www.encodeproject.org/files/ENCFF392XYY/@@download/ENCFF392XYY.bed.gz
https://www.encodeproject.org/files/ENCFF940DDM/@@download/ENCFF940DDM.bed.gz
https://www.encodeproject.org/files/ENCFF794SOC/@@download/ENCFF794SOC.bed.gz
https://www.encodeproject.org/files/ENCFF855YGO/@@download/ENCFF855YGO.bed.gz
https://www.encodeproject.org/files/ENCFF409QXF/@@download/ENCFF409QXF.bed.gz
https://www.encodeproject.org/files/ENCFF520HKD/@@download/ENCFF520HKD.bed.gz
https://www.encodeproject.org/files/ENCFF197ENC/@@download/ENCFF197ENC.bed.gz
https://www.encodeproject.org/files/ENCFF177AUZ/@@download/ENCFF177AUZ.bed.gz
https://www.encodeproject.org/files/ENCFF519RTR/@@download/ENCFF519RTR.bed.gz
https://www.encodeproject.org/files/ENCFF616MAA/@@download/ENCFF616MAA.bed.gz
https://www.encodeproject.org/files/ENCFF315TJF/@@download/ENCFF315TJF.bed.gz
https://www.encodeproject.org/files/ENCFF789TZB/@@download/ENCFF789TZB.bed.gz
https://www.encodeproject.org/files/ENCFF345RXP/@@download/ENCFF345RXP.bed.gz
https://www.encodeproject.org/files/ENCFF913NRZ/@@download/ENCFF913NRZ.bed.gz
https://www.encodeproject.org/files/ENCFF230CHA/@@download/ENCFF230CHA.bed.gz
https://www.encodeproject.org/files/ENCFF235NPS/@@download/ENCFF235NPS.bed.gz
https://www.encodeproject.org/files/ENCFF474QSW/@@download/ENCFF474QSW.bed.gz
https://www.encodeproject.org/files/ENCFF490EXW/@@download/ENCFF490EXW.bed.gz
https://www.encodeproject.org/files/ENCFF444TLL/@@download/ENCFF444TLL.bed.gz
https://www.encodeproject.org/files/ENCFF001UWE/@@download/ENCFF001UWE.bed.gz
https://www.encodeproject.org/files/ENCFF437GCZ/@@download/ENCFF437GCZ.bed.gz
https://www.encodeproject.org/files/ENCFF382IRS/@@download/ENCFF382IRS.bed.gz
https://www.encodeproject.org/files/ENCFF018HAH/@@download/ENCFF018HAH.bed.gz
https://www.encodeproject.org/files/ENCFF437RUN/@@download/ENCFF437RUN.bed.gz

https://www.encodeproject.org/files/ENCFF376WVL/@@download/ENCFF376WVL.bed.gz
https://www.encodeproject.org/files/ENCFF583HUF/@@download/ENCFF583HUF.bed.gz
https://www.encodeproject.org/files/ENCFF214ZLL/@@download/ENCFF214ZLL.bed.gz
https://www.encodeproject.org/files/ENCFF317QQX/@@download/ENCFF317QQX.bed.gz
https://www.encodeproject.org/files/ENCFF091CNN/@@download/ENCFF091CNN.bed.gz
https://www.encodeproject.org/files/ENCFF182PTE/@@download/ENCFF182PTE.bed.gz
https://www.encodeproject.org/files/ENCFF758REB/@@download/ENCFF758REB.bed.gz
https://www.encodeproject.org/files/ENCFF540HZS/@@download/ENCFF540HZS.bed.gz
https://www.encodeproject.org/files/ENCFF106VZG/@@download/ENCFF106VZG.bed.gz
https://www.encodeproject.org/files/ENCFF571GTC/@@download/ENCFF571GTC.bed.gz
https://www.encodeproject.org/files/ENCFF816OHA/@@download/ENCFF816OHA.bed.gz
https://www.encodeproject.org/files/ENCFF527JGW/@@download/ENCFF527JGW.bed.gz
https://www.encodeproject.org/files/ENCFF889NTH/@@download/ENCFF889NTH.bed.gz
https://www.encodeproject.org/files/ENCFF712AKP/@@download/ENCFF712AKP.bed.gz
https://www.encodeproject.org/files/ENCFF329GDP/@@download/ENCFF329GDP.bed.gz
https://www.encodeproject.org/files/ENCFF428SDY/@@download/ENCFF428SDY.bed.gz
https://www.encodeproject.org/files/ENCFF861KWC/@@download/ENCFF861KWC.bed.gz
https://www.encodeproject.org/files/ENCFF469UMQ/@@download/ENCFF469UMQ.bed.gz
https://www.encodeproject.org/files/ENCFF001SSD/@@download/ENCFF001SSD.bed.gz
https://www.encodeproject.org/files/ENCFF001WRX/@@download/ENCFF001WRX.bed.gz
https://www.encodeproject.org/files/ENCFF001WRY/@@download/ENCFF001WRY.bed.gz
https://www.encodeproject.org/files/ENCFF052DHL/@@download/ENCFF052DHL.bed.gz
https://www.encodeproject.org/files/ENCFF632UNR/@@download/ENCFF632UNR.bed.gz
https://www.encodeproject.org/files/ENCFF608WCU/@@download/ENCFF608WCU.bed.gz
https://www.encodeproject.org/files/ENCFF238YWY/@@download/ENCFF238YWY.bed.gz
https://www.encodeproject.org/files/ENCFF504FWZ/@@download/ENCFF504FWZ.bed.gz
https://www.encodeproject.org/files/ENCFF577MUT/@@download/ENCFF577MUT.bed.gz
https://www.encodeproject.org/files/ENCFF590KXD/@@download/ENCFF590KXD.bed.gz
https://www.encodeproject.org/files/ENCFF451SZH/@@download/ENCFF451SZH.bed.gz
https://www.encodeproject.org/files/ENCFF545OIN/@@download/ENCFF545OIN.bed.gz
https://www.encodeproject.org/files/ENCFF374QHY/@@download/ENCFF374QHY.bed.gz
https://www.encodeproject.org/files/ENCFF655IBQ/@@download/ENCFF655IBQ.bed.gz
https://www.encodeproject.org/files/ENCFF063KYR/@@download/ENCFF063KYR.bed.gz
https://www.encodeproject.org/files/ENCFF001SQV/@@download/ENCFF001SQV.bed.gz
https://www.encodeproject.org/files/ENCFF001VZU/@@download/ENCFF001VZU.bed.gz
https://www.encodeproject.org/files/ENCFF001WIO/@@download/ENCFF001WIO.bed.gz
https://www.encodeproject.org/files/ENCFF001WIN/@@download/ENCFF001WIN.bed.gz
https://www.encodeproject.org/files/ENCFF284HLK/@@download/ENCFF284HLK.bed.gz
https://www.encodeproject.org/files/ENCFF702GBT/@@download/ENCFF702GBT.bed.gz
https://www.encodeproject.org/files/ENCFF527EJQ/@@download/ENCFF527EJQ.bed.gz
https://www.encodeproject.org/files/ENCFF184TWB/@@download/ENCFF184TWB.bed.gz
https://www.encodeproject.org/files/ENCFF001WRH/@@download/ENCFF001WRH.bed.gz
https://www.encodeproject.org/files/ENCFF001WRI/@@download/ENCFF001WRI.bed.gz
https://www.encodeproject.org/files/ENCFF084GHJ/@@download/ENCFF084GHJ.bed.gz
https://www.encodeproject.org/files/ENCFF578XJU/@@download/ENCFF578XJU.bed.gz
https://www.encodeproject.org/files/ENCFF566JBQ/@@download/ENCFF566JBQ.bed.gz
https://www.encodeproject.org/files/ENCFF894LMB/@@download/ENCFF894LMB.bed.gz
https://www.encodeproject.org/files/ENCFF967SGG/@@download/ENCFF967SGG.bed.gz
https://www.encodeproject.org/files/ENCFF251QHT/@@download/ENCFF251QHT.bed.gz
https://www.encodeproject.org/files/ENCFF342VGE/@@download/ENCFF342VGE.bed.gz
https://www.encodeproject.org/files/ENCFF557DEE/@@download/ENCFF557DEE.bed.gz
https://www.encodeproject.org/files/ENCFF014NOY/@@download/ENCFF014NOY.bed.gz
https://www.encodeproject.org/files/ENCFF400ZBZ/@@download/ENCFF400ZBZ.bed.gz

https://www.encodeproject.org/files/ENCFF654RSS/@@download/ENCFF654RSS.bed.gz
https://www.encodeproject.org/files/ENCFF447XHB/@@download/ENCFF447XHB.bed.gz
https://www.encodeproject.org/files/ENCFF001SRJ/@@download/ENCFF001SRJ.bed.gz
https://www.encodeproject.org/files/ENCFF001WAG/@@download/ENCFF001WAG.bed.gz
https://www.encodeproject.org/files/ENCFF001WLE/@@download/ENCFF001WLE.bed.gz
https://www.encodeproject.org/files/ENCFF001WLD/@@download/ENCFF001WLD.bed.gz
https://www.encodeproject.org/files/ENCFF058QAN/@@download/ENCFF058QAN.bed.gz
https://www.encodeproject.org/files/ENCFF461WYH/@@download/ENCFF461WYH.bed.gz
https://www.encodeproject.org/files/ENCFF165ZIA/@@download/ENCFF165ZIA.bed.gz
https://www.encodeproject.org/files/ENCFF460ZFL/@@download/ENCFF460ZFL.bed.gz
https://www.encodeproject.org/files/ENCFF001UZB/@@download/ENCFF001UZB.bed.gz
https://www.encodeproject.org/files/ENCFF854QBS/@@download/ENCFF854QBS.bed.gz
https://www.encodeproject.org/files/ENCFF760NDX/@@download/ENCFF760NDX.bed.gz
https://www.encodeproject.org/files/ENCFF149ZMK/@@download/ENCFF149ZMK.bed.gz
https://www.encodeproject.org/files/ENCFF620WOM/@@download/ENCFF620WOM.bed.gz
https://www.encodeproject.org/files/ENCFF149IGI/@@download/ENCFF149IGI.bed.gz
https://www.encodeproject.org/files/ENCFF918YIN/@@download/ENCFF918YIN.bed.gz
https://www.encodeproject.org/files/ENCFF295IFW/@@download/ENCFF295IFW.bed.gz
https://www.encodeproject.org/files/ENCFF866PHQ/@@download/ENCFF866PHQ.bed.gz
https://www.encodeproject.org/files/ENCFF001SQP/@@download/ENCFF001SQP.bed.gz
https://www.encodeproject.org/files/ENCFF001VZM/@@download/ENCFF001VZM.bed.gz
https://www.encodeproject.org/files/ENCFF001WHQ/@@download/ENCFF001WHQ.bed.gz
https://www.encodeproject.org/files/ENCFF001WHP/@@download/ENCFF001WHP.bed.gz
https://www.encodeproject.org/files/ENCFF111NEU/@@download/ENCFF111NEU.bed.gz
https://www.encodeproject.org/files/ENCFF362MTG/@@download/ENCFF362MTG.bed.gz
https://www.encodeproject.org/files/ENCFF957GSL/@@download/ENCFF957GSL.bed.gz
https://www.encodeproject.org/files/ENCFF092IXN/@@download/ENCFF092IXN.bed.gz
https://www.encodeproject.org/files/ENCFF385OUX/@@download/ENCFF385OUX.bed.gz
https://www.encodeproject.org/files/ENCFF861PEN/@@download/ENCFF861PEN.bed.gz
https://www.encodeproject.org/files/ENCFF602NRX/@@download/ENCFF602NRX.bed.gz
https://www.encodeproject.org/files/ENCFF799KFN/@@download/ENCFF799KFN.bed.gz
https://www.encodeproject.org/files/ENCFF117NAW/@@download/ENCFF117NAW.bed.gz
https://www.encodeproject.org/files/ENCFF237ZHC/@@download/ENCFF237ZHC.bed.gz
https://www.encodeproject.org/files/ENCFF572NRY/@@download/ENCFF572NRY.bed.gz
https://www.encodeproject.org/files/ENCFF868BPC/@@download/ENCFF868BPC.bed.gz
https://www.encodeproject.org/files/ENCFF987KRZ/@@download/ENCFF987KRZ.bed.gz
https://www.encodeproject.org/files/ENCFF789ZWO/@@download/ENCFF789ZWO.bed.gz
https://www.encodeproject.org/files/ENCFF001SQL/@@download/ENCFF001SQL.bed.gz
https://www.encodeproject.org/files/ENCFF001VZG/@@download/ENCFF001VZG.bed.gz
https://www.encodeproject.org/files/ENCFF001WGU/@@download/ENCFF001WGU.bed.gz
https://www.encodeproject.org/files/ENCFF001WGT/@@download/ENCFF001WGT.bed.gz
https://www.encodeproject.org/files/ENCFF937JBR/@@download/ENCFF937JBR.bed.gz
https://www.encodeproject.org/files/ENCFF963HFG/@@download/ENCFF963HFG.bed.gz
https://www.encodeproject.org/files/ENCFF316LBU/@@download/ENCFF316LBU.bed.gz
https://www.encodeproject.org/files/ENCFF727SDN/@@download/ENCFF727SDN.bed.gz
https://www.encodeproject.org/files/ENCFF856UKF/@@download/ENCFF856UKF.bed.gz
https://www.encodeproject.org/files/ENCFF067IWG/@@download/ENCFF067IWG.bed.gz
https://www.encodeproject.org/files/ENCFF857QWC/@@download/ENCFF857QWC.bed.gz
https://www.encodeproject.org/files/ENCFF685XBT/@@download/ENCFF685XBT.bed.gz
https://www.encodeproject.org/files/ENCFF863BXZ/@@download/ENCFF863BXZ.bed.gz
https://www.encodeproject.org/files/ENCFF160EBT/@@download/ENCFF160EBT.bed.gz
https://www.encodeproject.org/files/ENCFF053HTN/@@download/ENCFF053HTN.bed.gz
https://www.encodeproject.org/files/ENCFF810ZKH/@@download/ENCFF810ZKH.bed.gz

https://www.encodeproject.org/files/ENCFF463JAI/@@download/ENCFF463JAI.bed.gz
https://www.encodeproject.org/files/ENCFF339IPG/@@download/ENCFF339IPG.bed.gz
https://www.encodeproject.org/files/ENCFF001SQZ/@@download/ENCFF001SQZ.bed.gz
https://www.encodeproject.org/files/ENCFF001WAC/@@download/ENCFF001WAC.bed.gz
https://www.encodeproject.org/files/ENCFF001WJM/@@download/ENCFF001WJM.bed.gz
https://www.encodeproject.org/files/ENCFF001WJL/@@download/ENCFF001WJL.bed.gz
https://www.encodeproject.org/files/ENCFF015FQU/@@download/ENCFF015FQU.bed.gz
https://www.encodeproject.org/files/ENCFF702SCH/@@download/ENCFF702SCH.bed.gz
https://www.encodeproject.org/files/ENCFF272LQP/@@download/ENCFF272LQP.bed.gz
https://www.encodeproject.org/files/ENCFF231PPP/@@download/ENCFF231PPP.bed.gz
https://www.encodeproject.org/files/ENCFF916NTG/@@download/ENCFF916NTG.bed.gz
https://www.encodeproject.org/files/ENCFF056MTI/@@download/ENCFF056MTI.bed.gz
https://www.encodeproject.org/files/ENCFF390RAS/@@download/ENCFF390RAS.bed.gz
https://www.encodeproject.org/files/ENCFF936OHE/@@download/ENCFF936OHE.bed.gz
https://www.encodeproject.org/files/ENCFF597TKH/@@download/ENCFF597TKH.bed.gz
https://www.encodeproject.org/files/ENCFF648NLY/@@download/ENCFF648NLY.bed.gz
https://www.encodeproject.org/files/ENCFF172XNI/@@download/ENCFF172XNI.bed.gz
https://www.encodeproject.org/files/ENCFF068ZPM/@@download/ENCFF068ZPM.bed.gz
https://www.encodeproject.org/files/ENCFF191MFV/@@download/ENCFF191MFV.bed.gz
https://www.encodeproject.org/files/ENCFF891XKE/@@download/ENCFF891XKE.bed.gz
https://www.encodeproject.org/files/ENCFF001SQR/@@download/ENCFF001SQR.bed.gz
https://www.encodeproject.org/files/ENCFF001VZQ/@@download/ENCFF001VZQ.bed.gz
https://www.encodeproject.org/files/ENCFF001WHY/@@download/ENCFF001WHY.bed.gz
https://www.encodeproject.org/files/ENCFF001WHX/@@download/ENCFF001WHX.bed.gz
https://www.encodeproject.org/files/ENCFF687CLF/@@download/ENCFF687CLF.bed.gz
https://www.encodeproject.org/files/ENCFF277DAW/@@download/ENCFF277DAW.bed.gz
https://www.encodeproject.org/files/ENCFF156RTG/@@download/ENCFF156RTG.bed.gz
https://www.encodeproject.org/files/ENCFF862ZMZ/@@download/ENCFF862ZMZ.bed.gz
https://www.encodeproject.org/files/ENCFF963BGI/@@download/ENCFF963BGI.bed.gz
https://www.encodeproject.org/files/ENCFF706BCL/@@download/ENCFF706BCL.bed.gz
https://www.encodeproject.org/files/ENCFF884LTQ/@@download/ENCFF884LTQ.bed.gz
https://www.encodeproject.org/files/ENCFF891BPN/@@download/ENCFF891BPN.bed.gz
https://www.encodeproject.org/files/ENCFF443ZJX/@@download/ENCFF443ZJX.bed.gz
https://www.encodeproject.org/files/ENCFF448DSH/@@download/ENCFF448DSH.bed.gz
https://www.encodeproject.org/files/ENCFF677LCJ/@@download/ENCFF677LCJ.bed.gz
https://www.encodeproject.org/files/ENCFF484TJS/@@download/ENCFF484TJS.bed.gz
https://www.encodeproject.org/files/ENCFF585WSJ/@@download/ENCFF585WSJ.bed.gz
https://www.encodeproject.org/files/ENCFF162VAR/@@download/ENCFF162VAR.bed.gz
https://www.encodeproject.org/files/ENCFF001SOH/@@download/ENCFF001SOH.bed.gz
https://www.encodeproject.org/files/ENCFF001UUK/@@download/ENCFF001UUK.bed.gz
https://www.encodeproject.org/files/ENCFF913HCF/@@download/ENCFF913HCF.bed.gz
https://www.encodeproject.org/files/ENCFF772FRH/@@download/ENCFF772FRH.bed.gz
https://www.encodeproject.org/files/ENCFF268ZNF/@@download/ENCFF268ZNF.bed.gz
https://www.encodeproject.org/files/ENCFF113DRH/@@download/ENCFF113DRH.bed.gz
https://www.encodeproject.org/files/ENCFF018IDK/@@download/ENCFF018IDK.bed.gz
https://www.encodeproject.org/files/ENCFF538ASZ/@@download/ENCFF538ASZ.bed.gz
https://www.encodeproject.org/files/ENCFF601WJA/@@download/ENCFF601WJA.bed.gz
https://www.encodeproject.org/files/ENCFF486OBJ/@@download/ENCFF486OBJ.bed.gz
https://www.encodeproject.org/files/ENCFF024HZS/@@download/ENCFF024HZS.bed.gz
https://www.encodeproject.org/files/ENCFF151DGI/@@download/ENCFF151DGI.bed.gz
https://www.encodeproject.org/files/ENCFF661UHC/@@download/ENCFF661UHC.bed.gz
https://www.encodeproject.org/files/ENCFF530CBM/@@download/ENCFF530CBM.bed.gz
https://www.encodeproject.org/files/ENCFF535LEW/@@download/ENCFF535LEW.bed.gz

https://www.encodeproject.org/files/ENCFF735JRV/@@download/ENCFF735JRV.bed.gz
https://www.encodeproject.org/files/ENCFF231QBI/@@download/ENCFF231QBI.bed.gz
https://www.encodeproject.org/files/ENCFF164IJW/@@download/ENCFF164IJW.bed.gz
https://www.encodeproject.org/files/ENCFF068EJC/@@download/ENCFF068EJC.bed.gz
https://www.encodeproject.org/files/ENCFF740UIQ/@@download/ENCFF740UIQ.bed.gz
https://www.encodeproject.org/files/ENCFF778BRJ/@@download/ENCFF778BRJ.bed.gz
https://www.encodeproject.org/files/ENCFF224GQJ/@@download/ENCFF224GQJ.bed.gz
https://www.encodeproject.org/files/ENCFF082AUS/@@download/ENCFF082AUS.bed.gz
https://www.encodeproject.org/files/ENCFF891RPL/@@download/ENCFF891RPL.bed.gz
https://www.encodeproject.org/files/ENCFF121KYV/@@download/ENCFF121KYV.bed.gz
https://www.encodeproject.org/files/ENCFF352VEF/@@download/ENCFF352VEF.bed.gz
https://www.encodeproject.org/files/ENCFF608VFB/@@download/ENCFF608VFB.bed.gz
https://www.encodeproject.org/files/ENCFF418EPC/@@download/ENCFF418EPC.bed.gz
https://www.encodeproject.org/files/ENCFF998JMX/@@download/ENCFF998JMX.bed.gz
https://www.encodeproject.org/files/ENCFF732VLL/@@download/ENCFF732VLL.bed.gz
https://www.encodeproject.org/files/ENCFF940MZC/@@download/ENCFF940MZC.bed.gz
https://www.encodeproject.org/files/ENCFF194KXV/@@download/ENCFF194KXV.bed.gz
https://www.encodeproject.org/files/ENCFF816UDC/@@download/ENCFF816UDC.bed.gz
https://www.encodeproject.org/files/ENCFF985ZYY/@@download/ENCFF985ZYY.bed.gz
https://www.encodeproject.org/files/ENCFF276XHO/@@download/ENCFF276XHO.bed.gz
https://www.encodeproject.org/files/ENCFF651ZLC/@@download/ENCFF651ZLC.bed.gz
https://www.encodeproject.org/files/ENCFF742WRC/@@download/ENCFF742WRC.bed.gz
https://www.encodeproject.org/files/ENCFF241USC/@@download/ENCFF241USC.bed.gz
https://www.encodeproject.org/files/ENCFF332HNX/@@download/ENCFF332HNX.bed.gz
https://www.encodeproject.org/files/ENCFF147XSC/@@download/ENCFF147XSC.bed.gz
https://www.encodeproject.org/files/ENCFF293PGI/@@download/ENCFF293PGI.bed.gz
https://www.encodeproject.org/files/ENCFF786GMY/@@download/ENCFF786GMY.bed.gz
https://www.encodeproject.org/files/ENCFF958VNA/@@download/ENCFF958VNA.bed.gz
https://www.encodeproject.org/files/ENCFF983SVC/@@download/ENCFF983SVC.bed.gz
https://www.encodeproject.org/files/ENCFF724SUH/@@download/ENCFF724SUH.bed.gz
https://www.encodeproject.org/files/ENCFF042VKK/@@download/ENCFF042VKK.bed.gz
https://www.encodeproject.org/files/ENCFF525FRH/@@download/ENCFF525FRH.bed.gz
https://www.encodeproject.org/files/ENCFF355GJG/@@download/ENCFF355GJG.bed.gz
https://www.encodeproject.org/files/ENCFF712HXM/@@download/ENCFF712HXM.bed.gz
https://www.encodeproject.org/files/ENCFF866WQO/@@download/ENCFF866WQO.bed.gz
https://www.encodeproject.org/files/ENCFF597DDU/@@download/ENCFF597DDU.bed.gz
https://www.encodeproject.org/files/ENCFF001WCL/@@download/ENCFF001WCL.bed.gz
https://www.encodeproject.org/files/ENCFF001WTC/@@download/ENCFF001WTC.bed.gz
https://www.encodeproject.org/files/ENCFF358FFI/@@download/ENCFF358FFI.bed.gz
https://www.encodeproject.org/files/ENCFF483RJF/@@download/ENCFF483RJF.bed.gz
https://www.encodeproject.org/files/ENCFF893GNV/@@download/ENCFF893GNV.bed.gz
https://www.encodeproject.org/files/ENCFF497MKO/@@download/ENCFF497MKO.bed.gz
https://www.encodeproject.org/files/ENCFF607HSK/@@download/ENCFF607HSK.bed.gz
https://www.encodeproject.org/files/ENCFF131FBL/@@download/ENCFF131FBL.bed.gz
https://www.encodeproject.org/files/ENCFF149NMR/@@download/ENCFF149NMR.bed.gz
https://www.encodeproject.org/files/ENCFF617JOF/@@download/ENCFF617JOF.bed.gz
https://www.encodeproject.org/files/ENCFF639QXT/@@download/ENCFF639QXT.bed.gz
https://www.encodeproject.org/files/ENCFF347GDH/@@download/ENCFF347GDH.bed.gz
https://www.encodeproject.org/files/ENCFF001SRE/@@download/ENCFF001SRE.bed.gz
https://www.encodeproject.org/files/ENCFF001WAI/@@download/ENCFF001WAI.bed.gz
https://www.encodeproject.org/files/ENCFF001WKK/@@download/ENCFF001WKK.bed.gz
https://www.encodeproject.org/files/ENCFF001WKJ/@@download/ENCFF001WKJ.bed.gz
https://www.encodeproject.org/files/ENCFF863RNS/@@download/ENCFF863RNS.bed.gz

Supplementary Table 1

https://www.encodeproject.org/files/ENCFF212KZW/@@download/ENCFF212KZW.bed.gz
https://www.encodeproject.org/files/ENCFF614FYH/@@download/ENCFF614FYH.bed.gz
https://www.encodeproject.org/files/ENCFF780DOD/@@download/ENCFF780DOD.bed.gz
https://www.encodeproject.org/files/ENCFF804HVH/@@download/ENCFF804HVH.bed.gz
https://www.encodeproject.org/files/ENCFF567LLQ/@@download/ENCFF567LLQ.bed.gz
https://www.encodeproject.org/files/ENCFF562HRV/@@download/ENCFF562HRV.bed.gz
https://www.encodeproject.org/files/ENCFF531MCH/@@download/ENCFF531MCH.bed.gz
https://www.encodeproject.org/files/ENCFF879CGW/@@download/ENCFF879CGW.bed.gz
https://www.encodeproject.org/files/ENCFF156GSY/@@download/ENCFF156GSY.bed.gz
https://www.encodeproject.org/files/ENCFF001SSA/@@download/ENCFF001SSA.bed.gz
https://www.encodeproject.org/files/ENCFF001WCA/@@download/ENCFF001WCA.bed.gz
https://www.encodeproject.org/files/ENCFF001WRM/@@download/ENCFF001WRM.bed.gz
https://www.encodeproject.org/files/ENCFF001WRL/@@download/ENCFF001WRL.bed.gz
https://www.encodeproject.org/files/ENCFF513YXO/@@download/ENCFF513YXO.bed.gz
https://www.encodeproject.org/files/ENCFF785NVB/@@download/ENCFF785NVB.bed.gz
https://www.encodeproject.org/files/ENCFF331SYD/@@download/ENCFF331SYD.bed.gz
https://www.encodeproject.org/files/ENCFF681UOZ/@@download/ENCFF681UOZ.bed.gz
https://www.encodeproject.org/files/ENCFF267DGC/@@download/ENCFF267DGC.bed.gz
https://www.encodeproject.org/files/ENCFF861DSY/@@download/ENCFF861DSY.bed.gz
https://www.encodeproject.org/files/ENCFF631ZRZ/@@download/ENCFF631ZRZ.bed.gz
https://www.encodeproject.org/files/ENCFF956DVZ/@@download/ENCFF956DVZ.bed.gz
https://www.encodeproject.org/files/ENCFF001SPZ/@@download/ENCFF001SPZ.bed.gz
https://www.encodeproject.org/files/ENCFF001VYU/@@download/ENCFF001VYU.bed.gz
https://www.encodeproject.org/files/ENCFF001WDZ/@@download/ENCFF001WDZ.bed.gz
https://www.encodeproject.org/files/ENCFF001WEA/@@download/ENCFF001WEA.bed.gz
https://www.encodeproject.org/files/ENCFF264FXA/@@download/ENCFF264FXA.bed.gz
https://www.encodeproject.org/files/ENCFF166JSD/@@download/ENCFF166JSD.bed.gz
https://www.encodeproject.org/files/ENCFF990HMA/@@download/ENCFF990HMA.bed.gz
https://www.encodeproject.org/files/ENCFF904WZA/@@download/ENCFF904WZA.bed.gz
https://www.encodeproject.org/files/ENCFF838BWI/@@download/ENCFF838BWI.bed.gz
https://www.encodeproject.org/files/ENCFF781DJJ/@@download/ENCFF781DJJ.bed.gz
https://www.encodeproject.org/files/ENCFF354BYJ/@@download/ENCFF354BYJ.bed.gz
https://www.encodeproject.org/files/ENCFF799TQB/@@download/ENCFF799TQB.bed.gz
https://www.encodeproject.org/files/ENCFF649SVT/@@download/ENCFF649SVT.bed.gz
https://www.encodeproject.org/files/ENCFF825ZLT/@@download/ENCFF825ZLT.bed.gz
https://www.encodeproject.org/files/ENCFF770MTY/@@download/ENCFF770MTY.bed.gz
https://www.encodeproject.org/files/ENCFF474TIO/@@download/ENCFF474TIO.bed.gz
https://www.encodeproject.org/files/ENCFF727HFV/@@download/ENCFF727HFV.bed.gz
https://www.encodeproject.org/files/ENCFF610JVS/@@download/ENCFF610JVS.bed.gz
https://www.encodeproject.org/files/ENCFF270TFI/@@download/ENCFF270TFI.bed.gz
https://www.encodeproject.org/files/ENCFF733UOT/@@download/ENCFF733UOT.bed.gz
https://www.encodeproject.org/files/ENCFF480AIG/@@download/ENCFF480AIG.bed.gz
https://www.encodeproject.org/files/ENCFF964MKU/@@download/ENCFF964MKU.bed.gz
https://www.encodeproject.org/files/ENCFF001WCS/@@download/ENCFF001WCS.bed.gz
https://www.encodeproject.org/files/ENCFF001WTI/@@download/ENCFF001WTI.bed.gz
https://www.encodeproject.org/files/ENCFF215XIO/@@download/ENCFF215XIO.bed.gz
https://www.encodeproject.org/files/ENCFF195KNE/@@download/ENCFF195KNE.bed.gz
https://www.encodeproject.org/files/ENCFF608NJK/@@download/ENCFF608NJK.bed.gz
https://www.encodeproject.org/files/ENCFF987AZZ/@@download/ENCFF987AZZ.bed.gz
https://www.encodeproject.org/files/ENCFF866OCE/@@download/ENCFF866OCE.bed.gz
https://www.encodeproject.org/files/ENCFF051VXY/@@download/ENCFF051VXY.bed.gz
https://www.encodeproject.org/files/ENCFF557HFW/@@download/ENCFF557HFW.bed.gz
https://www.encodeproject.org/files/ENCFF181GIN/@@download/ENCFF181GIN.bed.gz

Supplementary Table 1

https://www.encodeproject.org/files/ENCFF140XEZ/@@download/ENCFF140XEZ.bed.gz
https://www.encodeproject.org/files/ENCFF326MMH/@@download/ENCFF326MMH.bed.gz
https://www.encodeproject.org/files/ENCFF667BSO/@@download/ENCFF667BSO.bed.gz
https://www.encodeproject.org/files/ENCFF389QWJ/@@download/ENCFF389QWJ.bed.gz
https://www.encodeproject.org/files/ENCFF752JDS/@@download/ENCFF752JDS.bed.gz
https://www.encodeproject.org/files/ENCFF765ADK/@@download/ENCFF765ADK.bed.gz
https://www.encodeproject.org/files/ENCFF765SZB/@@download/ENCFF765SZB.bed.gz
https://www.encodeproject.org/files/ENCFF431WVQ/@@download/ENCFF431WVQ.bed.gz
https://www.encodeproject.org/files/ENCFF793VFP/@@download/ENCFF793VFP.bed.gz
https://www.encodeproject.org/files/ENCFF662RWK/@@download/ENCFF662RWK.bed.gz
https://www.encodeproject.org/files/ENCFF363HZW/@@download/ENCFF363HZW.bed.gz
https://www.encodeproject.org/files/ENCFF302YSW/@@download/ENCFF302YSW.bed.gz
https://www.encodeproject.org/files/ENCFF459ENG/@@download/ENCFF459ENG.bed.gz
https://www.encodeproject.org/files/ENCFF741SPF/@@download/ENCFF741SPF.bed.gz
https://www.encodeproject.org/files/ENCFF860TLU/@@download/ENCFF860TLU.bed.gz
https://www.encodeproject.org/files/ENCFF587SIS/@@download/ENCFF587SIS.bed.gz
https://www.encodeproject.org/files/ENCFF453JYL/@@download/ENCFF453JYL.bed.gz
https://www.encodeproject.org/files/ENCFF951BED/@@download/ENCFF951BED.bed.gz
https://www.encodeproject.org/files/ENCFF692EWL/@@download/ENCFF692EWL.bed.gz
https://www.encodeproject.org/files/ENCFF397GLL/@@download/ENCFF397GLL.bed.gz
https://www.encodeproject.org/files/ENCFF455NZK/@@download/ENCFF455NZK.bed.gz
https://www.encodeproject.org/files/ENCFF461EHY/@@download/ENCFF461EHY.bed.gz
https://www.encodeproject.org/files/ENCFF892CBZ/@@download/ENCFF892CBZ.bed.gz
https://www.encodeproject.org/files/ENCFF382TQZ/@@download/ENCFF382TQZ.bed.gz
https://www.encodeproject.org/files/ENCFF902VCD/@@download/ENCFF902VCD.bed.gz
https://www.encodeproject.org/files/ENCFF701IZA/@@download/ENCFF701IZA.bed.gz
https://www.encodeproject.org/files/ENCFF775ZBH/@@download/ENCFF775ZBH.bed.gz
https://www.encodeproject.org/files/ENCFF001SOV/@@download/ENCFF001SOV.bed.gz
https://www.encodeproject.org/files/ENCFF001UVT/@@download/ENCFF001UVT.bed.gz
https://www.encodeproject.org/files/ENCFF296IJA/@@download/ENCFF296IJA.bed.gz
https://www.encodeproject.org/files/ENCFF813GDL/@@download/ENCFF813GDL.bed.gz
https://www.encodeproject.org/files/ENCFF953XAB/@@download/ENCFF953XAB.bed.gz
https://www.encodeproject.org/files/ENCFF295BEY/@@download/ENCFF295BEY.bed.gz
https://www.encodeproject.org/files/ENCFF001SQW/@@download/ENCFF001SQW.bed.gz
https://www.encodeproject.org/files/ENCFF001VZY/@@download/ENCFF001VZY.bed.gz
https://www.encodeproject.org/files/ENCFF001WJA/@@download/ENCFF001WJA.bed.gz
https://www.encodeproject.org/files/ENCFF001WIZ/@@download/ENCFF001WIZ.bed.gz
https://www.encodeproject.org/files/ENCFF629TYN/@@download/ENCFF629TYN.bed.gz
https://www.encodeproject.org/files/ENCFF128YPL/@@download/ENCFF128YPL.bed.gz
https://www.encodeproject.org/files/ENCFF090PQC/@@download/ENCFF090PQC.bed.gz
https://www.encodeproject.org/files/ENCFF032LRL/@@download/ENCFF032LRL.bed.gz
https://www.encodeproject.org/files/ENCFF879ZZA/@@download/ENCFF879ZZA.bed.gz
https://www.encodeproject.org/files/ENCFF337NAS/@@download/ENCFF337NAS.bed.gz
https://www.encodeproject.org/files/ENCFF053XFC/@@download/ENCFF053XFC.bed.gz
https://www.encodeproject.org/files/ENCFF482VEA/@@download/ENCFF482VEA.bed.gz
https://www.encodeproject.org/files/ENCFF083YCN/@@download/ENCFF083YCN.bed.gz
https://www.encodeproject.org/files/ENCFF226AOZ/@@download/ENCFF226AOZ.bed.gz
https://www.encodeproject.org/files/ENCFF655ZJF/@@download/ENCFF655ZJF.bed.gz
https://www.encodeproject.org/files/ENCFF548SVP/@@download/ENCFF548SVP.bed.gz
https://www.encodeproject.org/files/ENCFF677BGN/@@download/ENCFF677BGN.bed.gz
https://www.encodeproject.org/files/ENCFF685YFE/@@download/ENCFF685YFE.bed.gz
https://www.encodeproject.org/files/ENCFF475UDQ/@@download/ENCFF475UDQ.bed.gz
https://www.encodeproject.org/files/ENCFF283LTB/@@download/ENCFF283LTB.bed.gz

https://www.encodeproject.org/files/ENCFF857MCB/@@download/ENCFF857MCB.bed.gz
https://www.encodeproject.org/files/ENCFF364XUA/@@download/ENCFF364XUA.bed.gz
https://www.encodeproject.org/files/ENCFF954RRL/@@download/ENCFF954RRL.bed.gz
https://www.encodeproject.org/files/ENCFF812LBJ/@@download/ENCFF812LBJ.bed.gz
https://www.encodeproject.org/files/ENCFF123DFX/@@download/ENCFF123DFX.bed.gz
https://www.encodeproject.org/files/ENCFF709LRW/@@download/ENCFF709LRW.bed.gz
https://www.encodeproject.org/files/ENCFF153TXZ/@@download/ENCFF153TXZ.bed.gz
https://www.encodeproject.org/files/ENCFF402ADZ/@@download/ENCFF402ADZ.bed.gz
https://www.encodeproject.org/files/ENCFF828NES/@@download/ENCFF828NES.bed.gz
https://www.encodeproject.org/files/ENCFF413SWP/@@download/ENCFF413SWP.bed.gz
https://www.encodeproject.org/files/ENCFF659ZTB/@@download/ENCFF659ZTB.bed.gz
https://www.encodeproject.org/files/ENCFF436MWY/@@download/ENCFF436MWY.bed.gz
https://www.encodeproject.org/files/ENCFF844BRK/@@download/ENCFF844BRK.bed.gz
https://www.encodeproject.org/files/ENCFF300MKL/@@download/ENCFF300MKL.bed.gz
https://www.encodeproject.org/files/ENCFF358HYU/@@download/ENCFF358HYU.bed.gz
https://www.encodeproject.org/files/ENCFF800XKN/@@download/ENCFF800XKN.bed.gz
https://www.encodeproject.org/files/ENCFF397VXC/@@download/ENCFF397VXC.bed.gz
https://www.encodeproject.org/files/ENCFF442KIW/@@download/ENCFF442KIW.bed.gz
https://www.encodeproject.org/files/ENCFF061IDT/@@download/ENCFF061IDT.bed.gz
https://www.encodeproject.org/files/ENCFF553OXP/@@download/ENCFF553OXP.bed.gz
https://www.encodeproject.org/files/ENCFF896VGY/@@download/ENCFF896VGY.bed.gz
https://www.encodeproject.org/files/ENCFF440OAH/@@download/ENCFF440OAH.bed.gz
https://www.encodeproject.org/files/ENCFF666SXY/@@download/ENCFF666SXY.bed.gz
https://www.encodeproject.org/files/ENCFF958UFJ/@@download/ENCFF958UFJ.bed.gz
https://www.encodeproject.org/files/ENCFF835QIK/@@download/ENCFF835QIK.bed.gz
https://www.encodeproject.org/files/ENCFF937DVO/@@download/ENCFF937DVO.bed.gz
https://www.encodeproject.org/files/ENCFF549DIW/@@download/ENCFF549DIW.bed.gz
https://www.encodeproject.org/files/ENCFF615GGO/@@download/ENCFF615GGO.bed.gz
https://www.encodeproject.org/files/ENCFF802QQT/@@download/ENCFF802QQT.bed.gz
https://www.encodeproject.org/files/ENCFF471TLK/@@download/ENCFF471TLK.bed.gz
https://www.encodeproject.org/files/ENCFF702TTL/@@download/ENCFF702TTL.bed.gz
https://www.encodeproject.org/files/ENCFF329BDX/@@download/ENCFF329BDX.bed.gz
https://www.encodeproject.org/files/ENCFF391VLP/@@download/ENCFF391VLP.bed.gz
https://www.encodeproject.org/files/ENCFF230RHI/@@download/ENCFF230RHI.bed.gz
https://www.encodeproject.org/files/ENCFF122IES/@@download/ENCFF122IES.bed.gz
https://www.encodeproject.org/files/ENCFF652NCR/@@download/ENCFF652NCR.bed.gz
https://www.encodeproject.org/files/ENCFF878LQP/@@download/ENCFF878LQP.bed.gz
https://www.encodeproject.org/files/ENCFF860VMW/@@download/ENCFF860VMW.bed.gz
https://www.encodeproject.org/files/ENCFF369GLM/@@download/ENCFF369GLM.bed.gz
https://www.encodeproject.org/files/ENCFF730GIJ/@@download/ENCFF730GIJ.bed.gz
https://www.encodeproject.org/files/ENCFF270OTN/@@download/ENCFF270OTN.bed.gz
https://www.encodeproject.org/files/ENCFF097UOL/@@download/ENCFF097UOL.bed.gz
https://www.encodeproject.org/files/ENCFF463KFI/@@download/ENCFF463KFI.bed.gz
https://www.encodeproject.org/files/ENCFF011XSC/@@download/ENCFF011XSC.bed.gz
https://www.encodeproject.org/files/ENCFF141TWX/@@download/ENCFF141TWX.bed.gz
https://www.encodeproject.org/files/ENCFF001SRM/@@download/ENCFF001SRM.bed.gz
https://www.encodeproject.org/files/ENCFF001WAQ/@@download/ENCFF001WAQ.bed.gz
https://www.encodeproject.org/files/ENCFF001WLO/@@download/ENCFF001WLO.bed.gz
https://www.encodeproject.org/files/ENCFF001WLN/@@download/ENCFF001WLN.bed.gz
https://www.encodeproject.org/files/ENCFF781MSJ/@@download/ENCFF781MSJ.bed.gz
https://www.encodeproject.org/files/ENCFF336ZKT/@@download/ENCFF336ZKT.bed.gz
https://www.encodeproject.org/files/ENCFF385ZNB/@@download/ENCFF385ZNB.bed.gz
https://www.encodeproject.org/files/ENCFF417VBX/@@download/ENCFF417VBX.bed.gz

https://www.encodeproject.org/files/ENCFF597SVJ/@@download/ENCFF597SVJ.bed.gz
https://www.encodeproject.org/files/ENCFF478KIZ/@@download/ENCFF478KIZ.bed.gz
https://www.encodeproject.org/files/ENCFF713NNM/@@download/ENCFF713NNM.bed.gz
https://www.encodeproject.org/files/ENCFF051JWD/@@download/ENCFF051JWD.bed.gz
https://www.encodeproject.org/files/ENCFF027MCW/@@download/ENCFF027MCW.bed.gz
https://www.encodeproject.org/files/ENCFF905MWC/@@download/ENCFF905MWC.bed.gz
https://www.encodeproject.org/files/ENCFF912FJN/@@download/ENCFF912FJN.bed.gz
https://www.encodeproject.org/files/ENCFF504DCZ/@@download/ENCFF504DCZ.bed.gz
https://www.encodeproject.org/files/ENCFF737WUQ/@@download/ENCFF737WUQ.bed.gz
https://www.encodeproject.org/files/ENCFF018ATG/@@download/ENCFF018ATG.bed.gz
https://www.encodeproject.org/files/ENCFF936VAD/@@download/ENCFF936VAD.bed.gz
https://www.encodeproject.org/files/ENCFF303THQ/@@download/ENCFF303THQ.bed.gz
https://www.encodeproject.org/files/ENCFF331KXA/@@download/ENCFF331KXA.bed.gz
https://www.encodeproject.org/files/ENCFF542IRR/@@download/ENCFF542IRR.bed.gz
https://www.encodeproject.org/files/ENCFF698UAJ/@@download/ENCFF698UAJ.bed.gz
https://www.encodeproject.org/files/ENCFF170QIG/@@download/ENCFF170QIG.bed.gz
https://www.encodeproject.org/files/ENCFF612OQV/@@download/ENCFF612OQV.bed.gz
https://www.encodeproject.org/files/ENCFF925ION/@@download/ENCFF925ION.bed.gz
https://www.encodeproject.org/files/ENCFF266BUC/@@download/ENCFF266BUC.bed.gz
https://www.encodeproject.org/files/ENCFF255MJU/@@download/ENCFF255MJU.bed.gz
https://www.encodeproject.org/files/ENCFF443QHY/@@download/ENCFF443QHY.bed.gz
https://www.encodeproject.org/files/ENCFF966LOF/@@download/ENCFF966LOF.bed.gz
https://www.encodeproject.org/files/ENCFF885VHR/@@download/ENCFF885VHR.bed.gz
https://www.encodeproject.org/files/ENCFF241TQC/@@download/ENCFF241TQC.bed.gz
https://www.encodeproject.org/files/ENCFF583GGY/@@download/ENCFF583GGY.bed.gz
https://www.encodeproject.org/files/ENCFF512ZNR/@@download/ENCFF512ZNR.bed.gz
https://www.encodeproject.org/files/ENCFF233RAN/@@download/ENCFF233RAN.bed.gz
https://www.encodeproject.org/files/ENCFF362VKQ/@@download/ENCFF362VKQ.bed.gz
https://www.encodeproject.org/files/ENCFF081JVT/@@download/ENCFF081JVT.bed.gz
https://www.encodeproject.org/files/ENCFF173YTQ/@@download/ENCFF173YTQ.bed.gz
https://www.encodeproject.org/files/ENCFF633NUP/@@download/ENCFF633NUP.bed.gz
https://www.encodeproject.org/files/ENCFF286LYP/@@download/ENCFF286LYP.bed.gz
https://www.encodeproject.org/files/ENCFF039XCN/@@download/ENCFF039XCN.bed.gz
https://www.encodeproject.org/files/ENCFF589IQE/@@download/ENCFF589IQE.bed.gz
https://www.encodeproject.org/files/ENCFF445OCG/@@download/ENCFF445OCG.bed.gz
https://www.encodeproject.org/files/ENCFF504YNM/@@download/ENCFF504YNM.bed.gz
https://www.encodeproject.org/files/ENCFF336YFC/@@download/ENCFF336YFC.bed.gz
https://www.encodeproject.org/files/ENCFF029JFN/@@download/ENCFF029JFN.bed.gz
https://www.encodeproject.org/files/ENCFF612BRI/@@download/ENCFF612BRI.bed.gz
https://www.encodeproject.org/files/ENCFF956PBI/@@download/ENCFF956PBI.bed.gz
https://www.encodeproject.org/files/ENCFF001SSG/@@download/ENCFF001SSG.bed.gz
https://www.encodeproject.org/files/ENCFF001WCG/@@download/ENCFF001WCG.bed.gz
https://www.encodeproject.org/files/ENCFF001WSO/@@download/ENCFF001WSO.bed.gz
https://www.encodeproject.org/files/ENCFF001WSN/@@download/ENCFF001WSN.bed.gz
https://www.encodeproject.org/files/ENCFF394WVR/@@download/ENCFF394WVR.bed.gz
https://www.encodeproject.org/files/ENCFF502RQJ/@@download/ENCFF502RQJ.bed.gz
https://www.encodeproject.org/files/ENCFF567KMJ/@@download/ENCFF567KMJ.bed.gz
https://www.encodeproject.org/files/ENCFF825ONF/@@download/ENCFF825ONF.bed.gz
https://www.encodeproject.org/files/ENCFF091FNC/@@download/ENCFF091FNC.bed.gz
https://www.encodeproject.org/files/ENCFF203SSC/@@download/ENCFF203SSC.bed.gz
https://www.encodeproject.org/files/ENCFF365EXK/@@download/ENCFF365EXK.bed.gz
https://www.encodeproject.org/files/ENCFF699CDH/@@download/ENCFF699CDH.bed.gz
https://www.encodeproject.org/files/ENCFF720OXL/@@download/ENCFF720OXL.bed.gz

Supplementary Table 1

https://www.encodeproject.org/files/ENCFF066MBH/@@download/ENCFF066MBH.bed.gz
https://www.encodeproject.org/files/ENCFF273TNO/@@download/ENCFF273TNO.bed.gz
https://www.encodeproject.org/files/ENCFF336OGZ/@@download/ENCFF336OGZ.bed.gz
https://www.encodeproject.org/files/ENCFF158JPT/@@download/ENCFF158JPT.bed.gz
https://www.encodeproject.org/files/ENCFF590BSA/@@download/ENCFF590BSA.bed.gz
https://www.encodeproject.org/files/ENCFF632RKP/@@download/ENCFF632RKP.bed.gz
https://www.encodeproject.org/files/ENCFF391OGO/@@download/ENCFF391OGO.bed.gz
https://www.encodeproject.org/files/ENCFF004LTR/@@download/ENCFF004LTR.bed.gz
https://www.encodeproject.org/files/ENCFF439GRJ/@@download/ENCFF439GRJ.bed.gz
https://www.encodeproject.org/files/ENCFF791OUO/@@download/ENCFF791OUO.bed.gz
https://www.encodeproject.org/files/ENCFF271JAF/@@download/ENCFF271JAF.bed.gz
https://www.encodeproject.org/files/ENCFF157JLM/@@download/ENCFF157JLM.bed.gz
https://www.encodeproject.org/files/ENCFF263ZFE/@@download/ENCFF263ZFE.bed.gz
https://www.encodeproject.org/files/ENCFF289IMF/@@download/ENCFF289IMF.bed.gz
https://www.encodeproject.org/files/ENCFF207NXB/@@download/ENCFF207NXB.bed.gz
https://www.encodeproject.org/files/ENCFF687FIQ/@@download/ENCFF687FIQ.bed.gz
https://www.encodeproject.org/files/ENCFF250FRA/@@download/ENCFF250FRA.bed.gz
https://www.encodeproject.org/files/ENCFF893JHA/@@download/ENCFF893JHA.bed.gz
https://www.encodeproject.org/files/ENCFF104UPL/@@download/ENCFF104UPL.bed.gz
https://www.encodeproject.org/files/ENCFF274OCE/@@download/ENCFF274OCE.bed.gz
https://www.encodeproject.org/files/ENCFF227SBF/@@download/ENCFF227SBF.bed.gz
https://www.encodeproject.org/files/ENCFF796YWP/@@download/ENCFF796YWP.bed.gz
https://www.encodeproject.org/files/ENCFF605MPK/@@download/ENCFF605MPK.bed.gz
https://www.encodeproject.org/files/ENCFF811RTH/@@download/ENCFF811RTH.bed.gz
https://www.encodeproject.org/files/ENCFF395KUT/@@download/ENCFF395KUT.bed.gz
https://www.encodeproject.org/files/ENCFF169QJZ/@@download/ENCFF169QJZ.bed.gz
https://www.encodeproject.org/files/ENCFF547ZRE/@@download/ENCFF547ZRE.bed.gz
https://www.encodeproject.org/files/ENCFF064EYI/@@download/ENCFF064EYI.bed.gz
https://www.encodeproject.org/files/ENCFF577HIH/@@download/ENCFF577HIH.bed.gz
https://www.encodeproject.org/files/ENCFF998CMA/@@download/ENCFF998CMA.bed.gz
https://www.encodeproject.org/files/ENCFF787FBC/@@download/ENCFF787FBC.bed.gz
https://www.encodeproject.org/files/ENCFF018UIV/@@download/ENCFF018UIV.bed.gz
https://www.encodeproject.org/files/ENCFF693XMO/@@download/ENCFF693XMO.bed.gz
https://www.encodeproject.org/files/ENCFF010YFK/@@download/ENCFF010YFK.bed.gz
https://www.encodeproject.org/files/ENCFF688ETO/@@download/ENCFF688ETO.bed.gz
https://www.encodeproject.org/files/ENCFF393CNI/@@download/ENCFF393CNI.bed.gz
https://www.encodeproject.org/files/ENCFF005KDI/@@download/ENCFF005KDI.bed.gz
https://www.encodeproject.org/files/ENCFF764COR/@@download/ENCFF764COR.bed.gz
https://www.encodeproject.org/files/ENCFF422EWO/@@download/ENCFF422EWO.bed.gz
https://www.encodeproject.org/files/ENCFF669AZU/@@download/ENCFF669AZU.bed.gz
https://www.encodeproject.org/files/ENCFF728TGG/@@download/ENCFF728TGG.bed.gz
https://www.encodeproject.org/files/ENCFF173CEG/@@download/ENCFF173CEG.bed.gz
https://www.encodeproject.org/files/ENCFF831SBJ/@@download/ENCFF831SBJ.bed.gz
https://www.encodeproject.org/files/ENCFF695MWM/@@download/ENCFF695MWM.bed.gz
https://www.encodeproject.org/files/ENCFF364YXZ/@@download/ENCFF364YXZ.bed.gz
https://www.encodeproject.org/files/ENCFF952XRR/@@download/ENCFF952XRR.bed.gz
https://www.encodeproject.org/files/ENCFF001WCY/@@download/ENCFF001WCY.bed.gz
https://www.encodeproject.org/files/ENCFF001WTW/@@download/ENCFF001WTW.bed.gz
https://www.encodeproject.org/files/ENCFF474XPL/@@download/ENCFF474XPL.bed.gz
https://www.encodeproject.org/files/ENCFF261BEF/@@download/ENCFF261BEF.bed.gz
https://www.encodeproject.org/files/ENCFF001SRT/@@download/ENCFF001SRT.bed.gz
https://www.encodeproject.org/files/ENCFF001WBC/@@download/ENCFF001WBC.bed.gz
https://www.encodeproject.org/files/ENCFF001WNG/@@download/ENCFF001WNG.bed.gz

Supplementary Table 1

https://www.encodeproject.org/files/ENCFF001WNF/@@download/ENCFF001WNF.bed.gz
https://www.encodeproject.org/files/ENCFF055IFJ/@@download/ENCFF055IFJ.bed.gz
https://www.encodeproject.org/files/ENCFF236XVM/@@download/ENCFF236XVM.bed.gz
https://www.encodeproject.org/files/ENCFF311UAO/@@download/ENCFF311UAO.bed.gz
https://www.encodeproject.org/files/ENCFF221FPW/@@download/ENCFF221FPW.bed.gz
https://www.encodeproject.org/files/ENCFF015MZL/@@download/ENCFF015MZL.bed.gz
https://www.encodeproject.org/files/ENCFF153XYG/@@download/ENCFF153XYG.bed.gz
https://www.encodeproject.org/files/ENCFF765AZQ/@@download/ENCFF765AZQ.bed.gz
https://www.encodeproject.org/files/ENCFF187BOF/@@download/ENCFF187BOF.bed.gz
https://www.encodeproject.org/files/ENCFF104PLB/@@download/ENCFF104PLB.bed.gz
https://www.encodeproject.org/files/ENCFF543NNF/@@download/ENCFF543NNF.bed.gz
https://www.encodeproject.org/files/ENCFF760JNT/@@download/ENCFF760JNT.bed.gz
https://www.encodeproject.org/files/ENCFF388MIC/@@download/ENCFF388MIC.bed.gz
https://www.encodeproject.org/files/ENCFF568UQT/@@download/ENCFF568UQT.bed.gz
https://www.encodeproject.org/files/ENCFF774SBQ/@@download/ENCFF774SBQ.bed.gz
https://www.encodeproject.org/files/ENCFF621IAQ/@@download/ENCFF621IAQ.bed.gz
https://www.encodeproject.org/files/ENCFF688IFG/@@download/ENCFF688IFG.bed.gz
https://www.encodeproject.org/files/ENCFF145VMR/@@download/ENCFF145VMR.bed.gz
https://www.encodeproject.org/files/ENCFF330QXG/@@download/ENCFF330QXG.bed.gz
https://www.encodeproject.org/files/ENCFF643WVI/@@download/ENCFF643WVI.bed.gz
https://www.encodeproject.org/files/ENCFF424BYY/@@download/ENCFF424BYY.bed.gz
https://www.encodeproject.org/files/ENCFF643MAK/@@download/ENCFF643MAK.bed.gz
https://www.encodeproject.org/files/ENCFF938NQJ/@@download/ENCFF938NQJ.bed.gz
https://www.encodeproject.org/files/ENCFF001SQT/@@download/ENCFF001SQT.bed.gz
https://www.encodeproject.org/files/ENCFF001VZS/@@download/ENCFF001VZS.bed.gz
https://www.encodeproject.org/files/ENCFF001WIG/@@download/ENCFF001WIG.bed.gz
https://www.encodeproject.org/files/ENCFF001WIF/@@download/ENCFF001WIF.bed.gz
https://www.encodeproject.org/files/ENCFF876SGV/@@download/ENCFF876SGV.bed.gz
https://www.encodeproject.org/files/ENCFF897RZM/@@download/ENCFF897RZM.bed.gz
https://www.encodeproject.org/files/ENCFF719FXT/@@download/ENCFF719FXT.bed.gz
https://www.encodeproject.org/files/ENCFF512IPA/@@download/ENCFF512IPA.bed.gz
https://www.encodeproject.org/files/ENCFF523YCQ/@@download/ENCFF523YCQ.bed.gz
https://www.encodeproject.org/files/ENCFF471LRB/@@download/ENCFF471LRB.bed.gz
https://www.encodeproject.org/files/ENCFF455ZFS/@@download/ENCFF455ZFS.bed.gz
https://www.encodeproject.org/files/ENCFF175GCC/@@download/ENCFF175GCC.bed.gz
https://www.encodeproject.org/files/ENCFF520YBR/@@download/ENCFF520YBR.bed.gz
https://www.encodeproject.org/files/ENCFF762WJJ/@@download/ENCFF762WJJ.bed.gz
https://www.encodeproject.org/files/ENCFF593YSG/@@download/ENCFF593YSG.bed.gz
https://www.encodeproject.org/files/ENCFF178CKS/@@download/ENCFF178CKS.bed.gz
https://www.encodeproject.org/files/ENCFF985KHI/@@download/ENCFF985KHI.bed.gz
https://www.encodeproject.org/files/ENCFF530EKX/@@download/ENCFF530EKX.bed.gz
https://www.encodeproject.org/files/ENCFF611KEX/@@download/ENCFF611KEX.bed.gz
https://www.encodeproject.org/files/ENCFF999XYV/@@download/ENCFF999XYV.bed.gz
https://www.encodeproject.org/files/ENCFF218ZFS/@@download/ENCFF218ZFS.bed.gz
https://www.encodeproject.org/files/ENCFF904HGV/@@download/ENCFF904HGV.bed.gz
https://www.encodeproject.org/files/ENCFF904WAI/@@download/ENCFF904WAI.bed.gz
https://www.encodeproject.org/files/ENCFF659LPO/@@download/ENCFF659LPO.bed.gz
https://www.encodeproject.org/files/ENCFF451HYJ/@@download/ENCFF451HYJ.bed.gz
https://www.encodeproject.org/files/ENCFF836JKO/@@download/ENCFF836JKO.bed.gz
https://www.encodeproject.org/files/ENCFF122TWP/@@download/ENCFF122TWP.bed.gz
https://www.encodeproject.org/files/ENCFF911LYH/@@download/ENCFF911LYH.bed.gz
https://www.encodeproject.org/files/ENCFF120FFI/@@download/ENCFF120FFI.bed.gz
https://www.encodeproject.org/files/ENCFF404LWH/@@download/ENCFF404LWH.bed.gz

Supplementary Table 1

https://www.encodeproject.org/files/ENCFF514GYQ/@@download/ENCFF514GYQ.bed.gz
https://www.encodeproject.org/files/ENCFF438AUW/@@download/ENCFF438AUW.bed.gz
https://www.encodeproject.org/files/ENCFF604WBU/@@download/ENCFF604WBU.bed.gz
https://www.encodeproject.org/files/ENCFF992EPV/@@download/ENCFF992EPV.bed.gz
https://www.encodeproject.org/files/ENCFF183AEI/@@download/ENCFF183AEI.bed.gz
https://www.encodeproject.org/files/ENCFF153WQN/@@download/ENCFF153WQN.bed.gz
https://www.encodeproject.org/files/ENCFF215RET/@@download/ENCFF215RET.bed.gz
https://www.encodeproject.org/files/ENCFF050XEY/@@download/ENCFF050XEY.bed.gz
https://www.encodeproject.org/files/ENCFF338EDR/@@download/ENCFF338EDR.bed.gz
https://www.encodeproject.org/files/ENCFF133DIO/@@download/ENCFF133DIO.bed.gz
https://www.encodeproject.org/files/ENCFF593BZP/@@download/ENCFF593BZP.bed.gz
https://www.encodeproject.org/files/ENCFF954SSK/@@download/ENCFF954SSK.bed.gz
https://www.encodeproject.org/files/ENCFF146TSF/@@download/ENCFF146TSF.bed.gz
https://www.encodeproject.org/files/ENCFF256RVA/@@download/ENCFF256RVA.bed.gz
https://www.encodeproject.org/files/ENCFF600YFF/@@download/ENCFF600YFF.bed.gz
https://www.encodeproject.org/files/ENCFF217BIX/@@download/ENCFF217BIX.bed.gz
https://www.encodeproject.org/files/ENCFF858IEB/@@download/ENCFF858IEB.bed.gz
https://www.encodeproject.org/files/ENCFF240MGQ/@@download/ENCFF240MGQ.bed.gz
https://www.encodeproject.org/files/ENCFF704BPH/@@download/ENCFF704BPH.bed.gz
https://www.encodeproject.org/files/ENCFF001UYP/@@download/ENCFF001UYP.bed.gz
https://www.encodeproject.org/files/ENCFF001SRG/@@download/ENCFF001SRG.bed.gz
https://www.encodeproject.org/files/ENCFF001WAM/@@download/ENCFF001WAM.bed.gz
https://www.encodeproject.org/files/ENCFF001WKS/@@download/ENCFF001WKS.bed.gz
https://www.encodeproject.org/files/ENCFF001WKR/@@download/ENCFF001WKR.bed.gz
https://www.encodeproject.org/files/ENCFF414XSB/@@download/ENCFF414XSB.bed.gz
https://www.encodeproject.org/files/ENCFF128JEJ/@@download/ENCFF128JEJ.bed.gz
https://www.encodeproject.org/files/ENCFF606XQS/@@download/ENCFF606XQS.bed.gz
https://www.encodeproject.org/files/ENCFF366UHV/@@download/ENCFF366UHV.bed.gz
https://www.encodeproject.org/files/ENCFF420FYX/@@download/ENCFF420FYX.bed.gz
https://www.encodeproject.org/files/ENCFF409PTH/@@download/ENCFF409PTH.bed.gz
https://www.encodeproject.org/files/ENCFF001UXE/@@download/ENCFF001UXE.bed.gz
https://www.encodeproject.org/files/ENCFF159CSX/@@download/ENCFF159CSX.bed.gz
https://www.encodeproject.org/files/ENCFF203EKC/@@download/ENCFF203EKC.bed.gz
https://www.encodeproject.org/files/ENCFF363XGK/@@download/ENCFF363XGK.bed.gz
https://www.encodeproject.org/files/ENCFF376RLJ/@@download/ENCFF376RLJ.bed.gz
https://www.encodeproject.org/files/ENCFF039YGO/@@download/ENCFF039YGO.bed.gz
https://www.encodeproject.org/files/ENCFF173ZQS/@@download/ENCFF173ZQS.bed.gz
https://www.encodeproject.org/files/ENCFF794ZPU/@@download/ENCFF794ZPU.bed.gz
https://www.encodeproject.org/files/ENCFF507VGO/@@download/ENCFF507VGO.bed.gz
https://www.encodeproject.org/files/ENCFF001SQO/@@download/ENCFF001SQO.bed.gz
https://www.encodeproject.org/files/ENCFF001WHF/@@download/ENCFF001WHF.bed.gz
https://www.encodeproject.org/files/ENCFF001WHG/@@download/ENCFF001WHG.bed.gz
https://www.encodeproject.org/files/ENCFF210JHJ/@@download/ENCFF210JHJ.bed.gz
https://www.encodeproject.org/files/ENCFF194IKX/@@download/ENCFF194IKX.bed.gz
https://www.encodeproject.org/files/ENCFF833HUK/@@download/ENCFF833HUK.bed.gz
https://www.encodeproject.org/files/ENCFF224NMM/@@download/ENCFF224NMM.bed.gz
https://www.encodeproject.org/files/ENCFF613TQY/@@download/ENCFF613TQY.bed.gz
https://www.encodeproject.org/files/ENCFF283RDM/@@download/ENCFF283RDM.bed.gz
https://www.encodeproject.org/files/ENCFF384MYP/@@download/ENCFF384MYP.bed.gz
https://www.encodeproject.org/files/ENCFF744YCN/@@download/ENCFF744YCN.bed.gz
https://www.encodeproject.org/files/ENCFF171CTL/@@download/ENCFF171CTL.bed.gz
https://www.encodeproject.org/files/ENCFF951DOZ/@@download/ENCFF951DOZ.bed.gz
https://www.encodeproject.org/files/ENCFF704JIN/@@download/ENCFF704JIN.bed.gz

Supplementary Table 1

https://www.encodeproject.org/files/ENCFF737DPY/@@download/ENCFF737DPY.bed.gz
https://www.encodeproject.org/files/ENCFF001WHL/@@download/ENCFF001WHL.bed.gz
https://www.encodeproject.org/files/ENCFF001WHM/@@download/ENCFF001WHM.bed.gz
https://www.encodeproject.org/files/ENCFF291PDD/@@download/ENCFF291PDD.bed.gz
https://www.encodeproject.org/files/ENCFF590DPJ/@@download/ENCFF590DPJ.bed.gz
https://www.encodeproject.org/files/ENCFF788MXD/@@download/ENCFF788MXD.bed.gz
https://www.encodeproject.org/files/ENCFF268UJI/@@download/ENCFF268UJI.bed.gz
https://www.encodeproject.org/files/ENCFF578YUB/@@download/ENCFF578YUB.bed.gz
https://www.encodeproject.org/files/ENCFF402ILF/@@download/ENCFF402ILF.bed.gz
https://www.encodeproject.org/files/ENCFF376KPO/@@download/ENCFF376KPO.bed.gz
https://www.encodeproject.org/files/ENCFF379CCP/@@download/ENCFF379CCP.bed.gz
https://www.encodeproject.org/files/ENCFF996VFH/@@download/ENCFF996VFH.bed.gz
https://www.encodeproject.org/files/ENCFF776GEG/@@download/ENCFF776GEG.bed.gz
https://www.encodeproject.org/files/ENCFF966MDV/@@download/ENCFF966MDV.bed.gz
https://www.encodeproject.org/files/ENCFF565HPT/@@download/ENCFF565HPT.bed.gz
https://www.encodeproject.org/files/ENCFF885UDD/@@download/ENCFF885UDD.bed.gz
https://www.encodeproject.org/files/ENCFF286WRO/@@download/ENCFF286WRO.bed.gz
https://www.encodeproject.org/files/ENCFF226NAB/@@download/ENCFF226NAB.bed.gz
https://www.encodeproject.org/files/ENCFF546ZMB/@@download/ENCFF546ZMB.bed.gz
https://www.encodeproject.org/files/ENCFF984RUT/@@download/ENCFF984RUT.bed.gz
https://www.encodeproject.org/files/ENCFF720MLF/@@download/ENCFF720MLF.bed.gz
https://www.encodeproject.org/files/ENCFF504ZVA/@@download/ENCFF504ZVA.bed.gz
https://www.encodeproject.org/files/ENCFF636PSO/@@download/ENCFF636PSO.bed.gz
https://www.encodeproject.org/files/ENCFF287SHD/@@download/ENCFF287SHD.bed.gz
https://www.encodeproject.org/files/ENCFF314YMQ/@@download/ENCFF314YMQ.bed.gz
https://www.encodeproject.org/files/ENCFF654GWE/@@download/ENCFF654GWE.bed.gz
https://www.encodeproject.org/files/ENCFF184GKA/@@download/ENCFF184GKA.bed.gz
https://www.encodeproject.org/files/ENCFF446SWE/@@download/ENCFF446SWE.bed.gz
https://www.encodeproject.org/files/ENCFF830VEQ/@@download/ENCFF830VEQ.bed.gz
https://www.encodeproject.org/files/ENCFF627LLR/@@download/ENCFF627LLR.bed.gz
https://www.encodeproject.org/files/ENCFF942ULR/@@download/ENCFF942ULR.bed.gz
https://www.encodeproject.org/files/ENCFF422YFH/@@download/ENCFF422YFH.bed.gz
https://www.encodeproject.org/files/ENCFF794QIS/@@download/ENCFF794QIS.bed.gz
https://www.encodeproject.org/files/ENCFF084GLJ/@@download/ENCFF084GLJ.bed.gz
https://www.encodeproject.org/files/ENCFF101FOH/@@download/ENCFF101FOH.bed.gz
https://www.encodeproject.org/files/ENCFF791HOY/@@download/ENCFF791HOY.bed.gz
https://www.encodeproject.org/files/ENCFF484TLE/@@download/ENCFF484TLE.bed.gz
https://www.encodeproject.org/files/ENCFF273EGX/@@download/ENCFF273EGX.bed.gz
https://www.encodeproject.org/files/ENCFF461SHV/@@download/ENCFF461SHV.bed.gz
https://www.encodeproject.org/files/ENCFF402THI/@@download/ENCFF402THI.bed.gz
https://www.encodeproject.org/files/ENCFF305WVB/@@download/ENCFF305WVB.bed.gz
https://www.encodeproject.org/files/ENCFF403LQM/@@download/ENCFF403LQM.bed.gz
https://www.encodeproject.org/files/ENCFF288VDM/@@download/ENCFF288VDM.bed.gz
https://www.encodeproject.org/files/ENCFF971IBL/@@download/ENCFF971IBL.bed.gz
https://www.encodeproject.org/files/ENCFF109TAE/@@download/ENCFF109TAE.bed.gz
https://www.encodeproject.org/files/ENCFF908XTT/@@download/ENCFF908XTT.bed.gz
https://www.encodeproject.org/files/ENCFF156QXF/@@download/ENCFF156QXF.bed.gz
https://www.encodeproject.org/files/ENCFF001SRO/@@download/ENCFF001SRO.bed.gz
https://www.encodeproject.org/files/ENCFF001WAU/@@download/ENCFF001WAU.bed.gz
https://www.encodeproject.org/files/ENCFF001WLW/@@download/ENCFF001WLW.bed.gz
https://www.encodeproject.org/files/ENCFF001WLV/@@download/ENCFF001WLV.bed.gz
https://www.encodeproject.org/files/ENCFF016RJA/@@download/ENCFF016RJA.bed.gz
https://www.encodeproject.org/files/ENCFF237KZE/@@download/ENCFF237KZE.bed.gz

https://www.encodeproject.org/files/ENCFF644XOI/@@download/ENCFF644XOI.bed.gz
https://www.encodeproject.org/files/ENCFF334RSR/@@download/ENCFF334RSR.bed.gz
https://www.encodeproject.org/files/ENCFF632IDM/@@download/ENCFF632IDM.bed.gz
https://www.encodeproject.org/files/ENCFF269BLL/@@download/ENCFF269BLL.bed.gz
https://www.encodeproject.org/files/ENCFF329KIL/@@download/ENCFF329KIL.bed.gz
https://www.encodeproject.org/files/ENCFF882RAE/@@download/ENCFF882RAE.bed.gz
https://www.encodeproject.org/files/ENCFF064YMI/@@download/ENCFF064YMI.bed.gz
https://www.encodeproject.org/files/ENCFF388VOD/@@download/ENCFF388VOD.bed.gz
https://www.encodeproject.org/files/ENCFF066WNI/@@download/ENCFF066WNI.bed.gz
https://www.encodeproject.org/files/ENCFF487VSR/@@download/ENCFF487VSR.bed.gz
https://www.encodeproject.org/files/ENCFF001SRZ/@@download/ENCFF001SRZ.bed.gz
https://www.encodeproject.org/files/ENCFF001WBY/@@download/ENCFF001WBY.bed.gz
https://www.encodeproject.org/files/ENCFF001WRE/@@download/ENCFF001WRE.bed.gz
https://www.encodeproject.org/files/ENCFF001WRD/@@download/ENCFF001WRD.bed.gz
https://www.encodeproject.org/files/ENCFF674URJ/@@download/ENCFF674URJ.bed.gz
https://www.encodeproject.org/files/ENCFF902XQY/@@download/ENCFF902XQY.bed.gz
https://www.encodeproject.org/files/ENCFF479KXR/@@download/ENCFF479KXR.bed.gz
https://www.encodeproject.org/files/ENCFF329APP/@@download/ENCFF329APP.bed.gz
https://www.encodeproject.org/files/ENCFF988ZPF/@@download/ENCFF988ZPF.bed.gz
https://www.encodeproject.org/files/ENCFF229RBJ/@@download/ENCFF229RBJ.bed.gz
https://www.encodeproject.org/files/ENCFF621XOE/@@download/ENCFF621XOE.bed.gz
https://www.encodeproject.org/files/ENCFF637BPM/@@download/ENCFF637BPM.bed.gz
https://www.encodeproject.org/files/ENCFF779JBV/@@download/ENCFF779JBV.bed.gz
https://www.encodeproject.org/files/ENCFF077XGP/@@download/ENCFF077XGP.bed.gz
https://www.encodeproject.org/files/ENCFF001SRF/@@download/ENCFF001SRF.bed.gz
https://www.encodeproject.org/files/ENCFF001WKN/@@download/ENCFF001WKN.bed.gz
https://www.encodeproject.org/files/ENCFF001WKO/@@download/ENCFF001WKO.bed.gz
https://www.encodeproject.org/files/ENCFF082KVB/@@download/ENCFF082KVB.bed.gz
https://www.encodeproject.org/files/ENCFF179XSD/@@download/ENCFF179XSD.bed.gz
https://www.encodeproject.org/files/ENCFF734QSB/@@download/ENCFF734QSB.bed.gz
https://www.encodeproject.org/files/ENCFF878LVP/@@download/ENCFF878LVP.bed.gz
https://www.encodeproject.org/files/ENCFF245TVT/@@download/ENCFF245TVT.bed.gz
https://www.encodeproject.org/files/ENCFF889ZCA/@@download/ENCFF889ZCA.bed.gz
https://www.encodeproject.org/files/ENCFF867OXG/@@download/ENCFF867OXG.bed.gz
https://www.encodeproject.org/files/ENCFF085LQJ/@@download/ENCFF085LQJ.bed.gz
https://www.encodeproject.org/files/ENCFF097WPC/@@download/ENCFF097WPC.bed.gz
https://www.encodeproject.org/files/ENCFF771PFT/@@download/ENCFF771PFT.bed.gz
https://www.encodeproject.org/files/ENCFF397IHK/@@download/ENCFF397IHK.bed.gz
https://www.encodeproject.org/files/ENCFF164HLK/@@download/ENCFF164HLK.bed.gz
https://www.encodeproject.org/files/ENCFF652DKF/@@download/ENCFF652DKF.bed.gz
https://www.encodeproject.org/files/ENCFF852FLF/@@download/ENCFF852FLF.bed.gz
https://www.encodeproject.org/files/ENCFF138MZJ/@@download/ENCFF138MZJ.bed.gz
https://www.encodeproject.org/files/ENCFF934DSX/@@download/ENCFF934DSX.bed.gz
https://www.encodeproject.org/files/ENCFF954PTR/@@download/ENCFF954PTR.bed.gz
https://www.encodeproject.org/files/ENCFF914GVC/@@download/ENCFF914GVC.bed.gz
https://www.encodeproject.org/files/ENCFF404USC/@@download/ENCFF404USC.bed.gz
https://www.encodeproject.org/files/ENCFF855AKX/@@download/ENCFF855AKX.bed.gz
https://www.encodeproject.org/files/ENCFF968IAI/@@download/ENCFF968IAI.bed.gz
https://www.encodeproject.org/files/ENCFF603RPR/@@download/ENCFF603RPR.bed.gz
https://www.encodeproject.org/files/ENCFF214TMC/@@download/ENCFF214TMC.bed.gz
https://www.encodeproject.org/files/ENCFF377OSW/@@download/ENCFF377OSW.bed.gz
https://www.encodeproject.org/files/ENCFF856OWH/@@download/ENCFF856OWH.bed.gz
https://www.encodeproject.org/files/ENCFF665WNI/@@download/ENCFF665WNI.bed.gz

Supplementary Table 1

https://www.encodeproject.org/files/ENCFF514PVZ/@@download/ENCFF514PVZ.bed.gz

https://www.encodeproject.org/files/ENCFF530SVX/@@download/ENCFF530SVX.bed.gz

https://www.encodeproject.org/files/ENCFF384WXP/@@download/ENCFF384WXP.bed.gz

https://www.encodeproject.org/files/ENCFF562ESR/@@download/ENCFF562ESR.bed.gz

https://www.encodeproject.org/files/ENCFF731IXN/@@download/ENCFF731IXN.bed.gz

https://www.encodeproject.org/files/ENCFF884CGH/@@download/ENCFF884CGH.bed.gz

https://www.encodeproject.org/files/ENCFF460WME/@@download/ENCFF460WME.bed.gz

https://www.encodeproject.org/files/ENCFF116OFN/@@download/ENCFF116OFN.bed.gz

https://www.encodeproject.org/files/ENCFF374WHQ/@@download/ENCFF374WHQ.bed.gz

https://www.encodeproject.org/files/ENCFF815BRT/@@download/ENCFF815BRT.bed.gz

https://www.encodeproject.org/files/ENCFF534XLO/@@download/ENCFF534XLO.bed.gz

https://www.encodeproject.org/files/ENCFF609AWS/@@download/ENCFF609AWS.bed.gz

https://www.encodeproject.org/files/ENCFF192ORU/@@download/ENCFF192ORU.bed.gz

https://www.encodeproject.org/files/ENCFF021ICC/@@download/ENCFF021ICC.bed.gz

https://www.encodeproject.org/files/ENCFF159SOA/@@download/ENCFF159SOA.bed.gz

https://www.encodeproject.org/files/ENCFF142WAA/@@download/ENCFF142WAA.bed.gz

https://www.encodeproject.org/files/ENCFF628DXI/@@download/ENCFF628DXI.bed.gz

https://www.encodeproject.org/files/ENCFF021XXM/@@download/ENCFF021XXM.bed.gz

https://www.encodeproject.org/files/ENCFF407LKC/@@download/ENCFF407LKC.bed.gz

https://www.encodeproject.org/files/ENCFF758SYE/@@download/ENCFF758SYE.bed.gz

https://www.encodeproject.org/files/ENCFF400WHP/@@download/ENCFF400WHP.bed.gz

https://www.encodeproject.org/files/ENCFF174RKM/@@download/ENCFF174RKM.bed.gz

https://www.encodeproject.org/files/ENCFF134KRY/@@download/ENCFF134KRY.bed.gz

https://www.encodeproject.org/files/ENCFF161NFM/@@download/ENCFF161NFM.bed.gz

https://www.encodeproject.org/files/ENCFF402AOJ/@@download/ENCFF402AOJ.bed.gz

https://www.encodeproject.org/files/ENCFF922SBV/@@download/ENCFF922SBV.bed.gz

Supplementary Table 1

ENCODE TFBS

https://www.encodeproject.org/metadata/type%3DExperiment%26status%3Dreleased%26assay_slims%3DDNA%2Bbinding%26assay_title%3DTF%2BChIP-seq%26replicates.library.biosample.donor.orga [illegible]
%3DHomo%2Bsapiens%26biosample_ontology.classification%3Dprimary%2Bcell%26biosample_ontology.classification%3Dtissue%26files.file_type%3Dbed%2BnarrowPeak%26audit.ERROR.category%2 [illegible]
%2Bcontrol%2Balignments%26audit.ERROR.category%2521%3Dextremely%2Blow%2Bread%2Bdepth%26audit.ERROR.category%2521%3Dcontrol%2Bextremely%2Blow%2Bread%2Bdepth/metadata. [illegible]

https://www.encodeproject.org/files/ENCFF002CEH/@@download/ENCFF002CEH.bed.gz

https://www.encodeproject.org/files/ENCFF084VMV/@@download/ENCFF084VMV.bed.gz

https://www.encodeproject.org/files/ENCFF655XWN/@@download/ENCFF655XWN.bed.gz

https://www.encodeproject.org/files/ENCFF522JCV/@@download/ENCFF522JCV.bed.gz

https://www.encodeproject.org/files/ENCFF536OTV/@@download/ENCFF536OTV.bed.gz

https://www.encodeproject.org/files/ENCFF733VAF/@@download/ENCFF733VAF.bed.gz

https://www.encodeproject.org/files/ENCFF873BHU/@@download/ENCFF873BHU.bed.gz

https://www.encodeproject.org/files/ENCFF843BCC/@@download/ENCFF843BCC.bed.gz

https://www.encodeproject.org/files/ENCFF846VQK/@@download/ENCFF846VQK.bed.gz

https://www.encodeproject.org/files/ENCFF249NQH/@@download/ENCFF249NQH.bed.gz

https://www.encodeproject.org/files/ENCFF637HAC/@@download/ENCFF637HAC.bed.gz

https://www.encodeproject.org/files/ENCFF173WQR/@@download/ENCFF173WQR.bed.gz

https://www.encodeproject.org/files/ENCFF668SIT/@@download/ENCFF668SIT.bed.gz

https://www.encodeproject.org/files/ENCFF385FXV/@@download/ENCFF385FXV.bed.gz

https://www.encodeproject.org/files/ENCFF409FXR/@@download/ENCFF409FXR.bed.gz

https://www.encodeproject.org/files/ENCFF179QLF/@@download/ENCFF179QLF.bed.gz

https://www.encodeproject.org/files/ENCFF873NGI/@@download/ENCFF873NGI.bed.gz

https://www.encodeproject.org/files/ENCFF300XXC/@@download/ENCFF300XXC.bed.gz

https://www.encodeproject.org/files/ENCFF446PEN/@@download/ENCFF446PEN.bed.gz

https://www.encodeproject.org/files/ENCFF097DFJ/@@download/ENCFF097DFJ.bed.gz

https://www.encodeproject.org/files/ENCFF920STO/@@download/ENCFF920STO.bed.gz

https://www.encodeproject.org/files/ENCFF920JJP/@@download/ENCFF920JJP.bed.gz

https://www.encodeproject.org/files/ENCFF437LHG/@@download/ENCFF437LHG.bed.gz

https://www.encodeproject.org/files/ENCFF571KJQ/@@download/ENCFF571KJQ.bed.gz

https://www.encodeproject.org/files/ENCFF628TDS/@@download/ENCFF628TDS.bed.gz

https://www.encodeproject.org/files/ENCFF875ZVM/@@download/ENCFF875ZVM.bed.gz

https://www.encodeproject.org/files/ENCFF872XQU/@@download/ENCFF872XQU.bed.gz

https://www.encodeproject.org/files/ENCFF308HRH/@@download/ENCFF308HRH.bed.gz

https://www.encodeproject.org/files/ENCFF141XWJ/@@download/ENCFF141XWJ.bed.gz

https://www.encodeproject.org/files/ENCFF913TNP/@@download/ENCFF913TNP.bed.gz

https://www.encodeproject.org/files/ENCFF591QYQ/@@download/ENCFF591QYQ.bed.gz

https://www.encodeproject.org/files/ENCFF236RJT/@@download/ENCFF236RJT.bed.gz

https://www.encodeproject.org/files/ENCFF552TWC/@@download/ENCFF552TWC.bed.gz

https://www.encodeproject.org/files/ENCFF673WMU/@@download/ENCFF673WMU.bed.gz

https://www.encodeproject.org/files/ENCFF459YEI/@@download/ENCFF459YEI.bed.gz

https://www.encodeproject.org/files/ENCFF379YTS/@@download/ENCFF379YTS.bed.gz

https://www.encodeproject.org/files/ENCFF774PLL/@@download/ENCFF774PLL.bed.gz

https://www.encodeproject.org/files/ENCFF396ZQL/@@download/ENCFF396ZQL.bed.gz

https://www.encodeproject.org/files/ENCFF996AFD/@@download/ENCFF996AFD.bed.gz

https://www.encodeproject.org/files/ENCFF035VNM/@@download/ENCFF035VNM.bed.gz

https://www.encodeproject.org/files/ENCFF672HZG/@@download/ENCFF672HZG.bed.gz

https://www.encodeproject.org/files/ENCFF566UPM/@@download/ENCFF566UPM.bed.gz

https://www.encodeproject.org/files/ENCFF156VNT/@@download/ENCFF156VNT.bed.gz

https://www.encodeproject.org/files/ENCFF900GKE/@@download/ENCFF900GKE.bed.gz

https://www.encodeproject.org/files/ENCFF401WKA/@@download/ENCFF401WKA.bed.gz

https://www.encodeproject.org/files/ENCFF733AVG/@@download/ENCFF733AVG.bed.gz

https://www.encodeproject.org/files/ENCFF325VGL/@@download/ENCFF325VGL.bed.gz

https://www.encodeproject.org/files/ENCFF137VWI/@@download/ENCFF137VWI.bed.gz

Supplementary Table 1

https://www.encodeproject.org/files/ENCFF777RPJ/@@download/ENCFF777RPJ.bed.gz
https://www.encodeproject.org/files/ENCFF637FOR/@@download/ENCFF637FOR.bed.gz
https://www.encodeproject.org/files/ENCFF173TPC/@@download/ENCFF173TPC.bed.gz
https://www.encodeproject.org/files/ENCFF436ENK/@@download/ENCFF436ENK.bed.gz
https://www.encodeproject.org/files/ENCFF916FGF/@@download/ENCFF916FGF.bed.gz
https://www.encodeproject.org/files/ENCFF245JXB/@@download/ENCFF245JXB.bed.gz
https://www.encodeproject.org/files/ENCFF215GGE/@@download/ENCFF215GGE.bed.gz
https://www.encodeproject.org/files/ENCFF411KGD/@@download/ENCFF411KGD.bed.gz
https://www.encodeproject.org/files/ENCFF574FIL/@@download/ENCFF574FIL.bed.gz
https://www.encodeproject.org/files/ENCFF001XQD/@@download/ENCFF001XQD.bed.gz
https://www.encodeproject.org/files/ENCFF001XQE/@@download/ENCFF001XQE.bed.gz
https://www.encodeproject.org/files/ENCFF002DCR/@@download/ENCFF002DCR.bed.gz
https://www.encodeproject.org/files/ENCFF356GCX/@@download/ENCFF356GCX.bed.gz
https://www.encodeproject.org/files/ENCFF530JSJ/@@download/ENCFF530JSJ.bed.gz
https://www.encodeproject.org/files/ENCFF306OJZ/@@download/ENCFF306OJZ.bed.gz
https://www.encodeproject.org/files/ENCFF700UZN/@@download/ENCFF700UZN.bed.gz
https://www.encodeproject.org/files/ENCFF301YXM/@@download/ENCFF301YXM.bed.gz
https://www.encodeproject.org/files/ENCFF574BPV/@@download/ENCFF574BPV.bed.gz
https://www.encodeproject.org/files/ENCFF504WUQ/@@download/ENCFF504WUQ.bed.gz
https://www.encodeproject.org/files/ENCFF386NQE/@@download/ENCFF386NQE.bed.gz
https://www.encodeproject.org/files/ENCFF266GGD/@@download/ENCFF266GGD.bed.gz
https://www.encodeproject.org/files/ENCFF883SBZ/@@download/ENCFF883SBZ.bed.gz
https://www.encodeproject.org/files/ENCFF313WYX/@@download/ENCFF313WYX.bed.gz
https://www.encodeproject.org/files/ENCFF296AFJ/@@download/ENCFF296AFJ.bed.gz
https://www.encodeproject.org/files/ENCFF628UBC/@@download/ENCFF628UBC.bed.gz
https://www.encodeproject.org/files/ENCFF446QIR/@@download/ENCFF446QIR.bed.gz
https://www.encodeproject.org/files/ENCFF101PDP/@@download/ENCFF101PDP.bed.gz
https://www.encodeproject.org/files/ENCFF693ETV/@@download/ENCFF693ETV.bed.gz
https://www.encodeproject.org/files/ENCFF281XHU/@@download/ENCFF281XHU.bed.gz
https://www.encodeproject.org/files/ENCFF587UBP/@@download/ENCFF587UBP.bed.gz
https://www.encodeproject.org/files/ENCFF001XRR/@@download/ENCFF001XRR.bed.gz
https://www.encodeproject.org/files/ENCFF001XRS/@@download/ENCFF001XRS.bed.gz
https://www.encodeproject.org/files/ENCFF002DDC/@@download/ENCFF002DDC.bed.gz
https://www.encodeproject.org/files/ENCFF836OOE/@@download/ENCFF836OOE.bed.gz
https://www.encodeproject.org/files/ENCFF196CRQ/@@download/ENCFF196CRQ.bed.gz
https://www.encodeproject.org/files/ENCFF257JPC/@@download/ENCFF257JPC.bed.gz
https://www.encodeproject.org/files/ENCFF152YHL/@@download/ENCFF152YHL.bed.gz
https://www.encodeproject.org/files/ENCFF090XCI/@@download/ENCFF090XCI.bed.gz
https://www.encodeproject.org/files/ENCFF681HOI/@@download/ENCFF681HOI.bed.gz
https://www.encodeproject.org/files/ENCFF438XHB/@@download/ENCFF438XHB.bed.gz
https://www.encodeproject.org/files/ENCFF831MNI/@@download/ENCFF831MNI.bed.gz
https://www.encodeproject.org/files/ENCFF383NCL/@@download/ENCFF383NCL.bed.gz
https://www.encodeproject.org/files/ENCFF054PUP/@@download/ENCFF054PUP.bed.gz
https://www.encodeproject.org/files/ENCFF031IUJ/@@download/ENCFF031IUJ.bed.gz
https://www.encodeproject.org/files/ENCFF992DNN/@@download/ENCFF992DNN.bed.gz
https://www.encodeproject.org/files/ENCFF002CLL/@@download/ENCFF002CLL.bed.gz
https://www.encodeproject.org/files/ENCFF003WNJ/@@download/ENCFF003WNJ.bed.gz
https://www.encodeproject.org/files/ENCFF423WPT/@@download/ENCFF423WPT.bed.gz
https://www.encodeproject.org/files/ENCFF129JER/@@download/ENCFF129JER.bed.gz
https://www.encodeproject.org/files/ENCFF821DBG/@@download/ENCFF821DBG.bed.gz
https://www.encodeproject.org/files/ENCFF347JZD/@@download/ENCFF347JZD.bed.gz
https://www.encodeproject.org/files/ENCFF351KKK/@@download/ENCFF351KKK.bed.gz
https://www.encodeproject.org/files/ENCFF509LBE/@@download/ENCFF509LBE.bed.gz

https://www.encodeproject.org/files/ENCFF560WFS/@@download/ENCFF560WFS.bed.gz
https://www.encodeproject.org/files/ENCFF777TLS/@@download/ENCFF777TLS.bed.gz
https://www.encodeproject.org/files/ENCFF766ZRX/@@download/ENCFF766ZRX.bed.gz
https://www.encodeproject.org/files/ENCFF749ROX/@@download/ENCFF749ROX.bed.gz
https://www.encodeproject.org/files/ENCFF985OZU/@@download/ENCFF985OZU.bed.gz
https://www.encodeproject.org/files/ENCFF100SKI/@@download/ENCFF100SKI.bed.gz
https://www.encodeproject.org/files/ENCFF106ONT/@@download/ENCFF106ONT.bed.gz
https://www.encodeproject.org/files/ENCFF001VQT/@@download/ENCFF001VQT.bed.gz
https://www.encodeproject.org/files/ENCFF002CZX/@@download/ENCFF002CZX.bed.gz
https://www.encodeproject.org/files/ENCFF871KMV/@@download/ENCFF871KMV.bed.gz
https://www.encodeproject.org/files/ENCFF468KWV/@@download/ENCFF468KWV.bed.gz
https://www.encodeproject.org/files/ENCFF450JIP/@@download/ENCFF450JIP.bed.gz
https://www.encodeproject.org/files/ENCFF789ZAT/@@download/ENCFF789ZAT.bed.gz
https://www.encodeproject.org/files/ENCFF176LMS/@@download/ENCFF176LMS.bed.gz
https://www.encodeproject.org/files/ENCFF039AWP/@@download/ENCFF039AWP.bed.gz
https://www.encodeproject.org/files/ENCFF218LZW/@@download/ENCFF218LZW.bed.gz
https://www.encodeproject.org/files/ENCFF535FRY/@@download/ENCFF535FRY.bed.gz
https://www.encodeproject.org/files/ENCFF455BVU/@@download/ENCFF455BVU.bed.gz
https://www.encodeproject.org/files/ENCFF762DRA/@@download/ENCFF762DRA.bed.gz
https://www.encodeproject.org/files/ENCFF002CLM/@@download/ENCFF002CLM.bed.gz
https://www.encodeproject.org/files/ENCFF704JVW/@@download/ENCFF704JVW.bed.gz
https://www.encodeproject.org/files/ENCFF082NLH/@@download/ENCFF082NLH.bed.gz
https://www.encodeproject.org/files/ENCFF297ZIE/@@download/ENCFF297ZIE.bed.gz
https://www.encodeproject.org/files/ENCFF510UHF/@@download/ENCFF510UHF.bed.gz
https://www.encodeproject.org/files/ENCFF876VZQ/@@download/ENCFF876VZQ.bed.gz
https://www.encodeproject.org/files/ENCFF791OTS/@@download/ENCFF791OTS.bed.gz
https://www.encodeproject.org/files/ENCFF387VGY/@@download/ENCFF387VGY.bed.gz
https://www.encodeproject.org/files/ENCFF024IBB/@@download/ENCFF024IBB.bed.gz
https://www.encodeproject.org/files/ENCFF482VYS/@@download/ENCFF482VYS.bed.gz
https://www.encodeproject.org/files/ENCFF138NNW/@@download/ENCFF138NNW.bed.gz
https://www.encodeproject.org/files/ENCFF800RTH/@@download/ENCFF800RTH.bed.gz
https://www.encodeproject.org/files/ENCFF501YCY/@@download/ENCFF501YCY.bed.gz
https://www.encodeproject.org/files/ENCFF654DFC/@@download/ENCFF654DFC.bed.gz
https://www.encodeproject.org/files/ENCFF857VSJ/@@download/ENCFF857VSJ.bed.gz
https://www.encodeproject.org/files/ENCFF233DOW/@@download/ENCFF233DOW.bed.gz
https://www.encodeproject.org/files/ENCFF134AVY/@@download/ENCFF134AVY.bed.gz
https://www.encodeproject.org/files/ENCFF315BSV/@@download/ENCFF315BSV.bed.gz
https://www.encodeproject.org/files/ENCFF318YTP/@@download/ENCFF318YTP.bed.gz
https://www.encodeproject.org/files/ENCFF429VMY/@@download/ENCFF429VMY.bed.gz
https://www.encodeproject.org/files/ENCFF500XIG/@@download/ENCFF500XIG.bed.gz
https://www.encodeproject.org/files/ENCFF885SEW/@@download/ENCFF885SEW.bed.gz
https://www.encodeproject.org/files/ENCFF392GXE/@@download/ENCFF392GXE.bed.gz
https://www.encodeproject.org/files/ENCFF743OES/@@download/ENCFF743OES.bed.gz
https://www.encodeproject.org/files/ENCFF605HWY/@@download/ENCFF605HWY.bed.gz
https://www.encodeproject.org/files/ENCFF237NQY/@@download/ENCFF237NQY.bed.gz
https://www.encodeproject.org/files/ENCFF261BWI/@@download/ENCFF261BWI.bed.gz
https://www.encodeproject.org/files/ENCFF886WWT/@@download/ENCFF886WWT.bed.gz
https://www.encodeproject.org/files/ENCFF786QZK/@@download/ENCFF786QZK.bed.gz
https://www.encodeproject.org/files/ENCFF956IFC/@@download/ENCFF956IFC.bed.gz
https://www.encodeproject.org/files/ENCFF128OKK/@@download/ENCFF128OKK.bed.gz
https://www.encodeproject.org/files/ENCFF562OUS/@@download/ENCFF562OUS.bed.gz
https://www.encodeproject.org/files/ENCFF023IZV/@@download/ENCFF023IZV.bed.gz
https://www.encodeproject.org/files/ENCFF250ZAH/@@download/ENCFF250ZAH.bed.gz

https://www.encodeproject.org/files/ENCFF822XRK/@@download/ENCFF822XRK.bed.gz
https://www.encodeproject.org/files/ENCFF941YYK/@@download/ENCFF941YYK.bed.gz
https://www.encodeproject.org/files/ENCFF482LGP/@@download/ENCFF482LGP.bed.gz
https://www.encodeproject.org/files/ENCFF954XNA/@@download/ENCFF954XNA.bed.gz
https://www.encodeproject.org/files/ENCFF349RNE/@@download/ENCFF349RNE.bed.gz
https://www.encodeproject.org/files/ENCFF291RDN/@@download/ENCFF291RDN.bed.gz
https://www.encodeproject.org/files/ENCFF052MLC/@@download/ENCFF052MLC.bed.gz
https://www.encodeproject.org/files/ENCFF597JRR/@@download/ENCFF597JRR.bed.gz
https://www.encodeproject.org/files/ENCFF149EWP/@@download/ENCFF149EWP.bed.gz
https://www.encodeproject.org/files/ENCFF002CEX/@@download/ENCFF002CEX.bed.gz
https://www.encodeproject.org/files/ENCFF951JAH/@@download/ENCFF951JAH.bed.gz
https://www.encodeproject.org/files/ENCFF034NUV/@@download/ENCFF034NUV.bed.gz
https://www.encodeproject.org/files/ENCFF072MPX/@@download/ENCFF072MPX.bed.gz
https://www.encodeproject.org/files/ENCFF672PKP/@@download/ENCFF672PKP.bed.gz
https://www.encodeproject.org/files/ENCFF473KUA/@@download/ENCFF473KUA.bed.gz
https://www.encodeproject.org/files/ENCFF399OOG/@@download/ENCFF399OOG.bed.gz
https://www.encodeproject.org/files/ENCFF285ODQ/@@download/ENCFF285ODQ.bed.gz
https://www.encodeproject.org/files/ENCFF050TAS/@@download/ENCFF050TAS.bed.gz
https://www.encodeproject.org/files/ENCFF001XRE/@@download/ENCFF001XRE.bed.gz
https://www.encodeproject.org/files/ENCFF002DCY/@@download/ENCFF002DCY.bed.gz
https://www.encodeproject.org/files/ENCFF017PXX/@@download/ENCFF017PXX.bed.gz
https://www.encodeproject.org/files/ENCFF652MLT/@@download/ENCFF652MLT.bed.gz
https://www.encodeproject.org/files/ENCFF412LSU/@@download/ENCFF412LSU.bed.gz
https://www.encodeproject.org/files/ENCFF273NIW/@@download/ENCFF273NIW.bed.gz
https://www.encodeproject.org/files/ENCFF619FOL/@@download/ENCFF619FOL.bed.gz
https://www.encodeproject.org/files/ENCFF306CZZ/@@download/ENCFF306CZZ.bed.gz
https://www.encodeproject.org/files/ENCFF994CVU/@@download/ENCFF994CVU.bed.gz
https://www.encodeproject.org/files/ENCFF760CPU/@@download/ENCFF760CPU.bed.gz
https://www.encodeproject.org/files/ENCFF002CEE/@@download/ENCFF002CEE.bed.gz
https://www.encodeproject.org/files/ENCFF901AJE/@@download/ENCFF901AJE.bed.gz
https://www.encodeproject.org/files/ENCFF629BKP/@@download/ENCFF629BKP.bed.gz
https://www.encodeproject.org/files/ENCFF610BYU/@@download/ENCFF610BYU.bed.gz
https://www.encodeproject.org/files/ENCFF120NIX/@@download/ENCFF120NIX.bed.gz
https://www.encodeproject.org/files/ENCFF128OWK/@@download/ENCFF128OWK.bed.gz
https://www.encodeproject.org/files/ENCFF965BIL/@@download/ENCFF965BIL.bed.gz
https://www.encodeproject.org/files/ENCFF442AZU/@@download/ENCFF442AZU.bed.gz
https://www.encodeproject.org/files/ENCFF465WVW/@@download/ENCFF465WVW.bed.gz
https://www.encodeproject.org/files/ENCFF380TJK/@@download/ENCFF380TJK.bed.gz
https://www.encodeproject.org/files/ENCFF801WBW/@@download/ENCFF801WBW.bed.gz
https://www.encodeproject.org/files/ENCFF972ODR/@@download/ENCFF972ODR.bed.gz
https://www.encodeproject.org/files/ENCFF091KSY/@@download/ENCFF091KSY.bed.gz
https://www.encodeproject.org/files/ENCFF885NNA/@@download/ENCFF885NNA.bed.gz
https://www.encodeproject.org/files/ENCFF038BWR/@@download/ENCFF038BWR.bed.gz
https://www.encodeproject.org/files/ENCFF846YGT/@@download/ENCFF846YGT.bed.gz
https://www.encodeproject.org/files/ENCFF044MZF/@@download/ENCFF044MZF.bed.gz
https://www.encodeproject.org/files/ENCFF727LYG/@@download/ENCFF727LYG.bed.gz
https://www.encodeproject.org/files/ENCFF013PVV/@@download/ENCFF013PVV.bed.gz
https://www.encodeproject.org/files/ENCFF184KZB/@@download/ENCFF184KZB.bed.gz
https://www.encodeproject.org/files/ENCFF628MXS/@@download/ENCFF628MXS.bed.gz
https://www.encodeproject.org/files/ENCFF208GHS/@@download/ENCFF208GHS.bed.gz
https://www.encodeproject.org/files/ENCFF229WFR/@@download/ENCFF229WFR.bed.gz
https://www.encodeproject.org/files/ENCFF271YUO/@@download/ENCFF271YUO.bed.gz
https://www.encodeproject.org/files/ENCFF108ZTV/@@download/ENCFF108ZTV.bed.gz

https://www.encodeproject.org/files/ENCFF149VRW/@@download/ENCFF149VRW.bed.gz
https://www.encodeproject.org/files/ENCFF704KAE/@@download/ENCFF704KAE.bed.gz
https://www.encodeproject.org/files/ENCFF208SZB/@@download/ENCFF208SZB.bed.gz
https://www.encodeproject.org/files/ENCFF266KJH/@@download/ENCFF266KJH.bed.gz
https://www.encodeproject.org/files/ENCFF617HUR/@@download/ENCFF617HUR.bed.gz
https://www.encodeproject.org/files/ENCFF525BTB/@@download/ENCFF525BTB.bed.gz
https://www.encodeproject.org/files/ENCFF348QEP/@@download/ENCFF348QEP.bed.gz
https://www.encodeproject.org/files/ENCFF266KLW/@@download/ENCFF266KLW.bed.gz
https://www.encodeproject.org/files/ENCFF192NOU/@@download/ENCFF192NOU.bed.gz
https://www.encodeproject.org/files/ENCFF495GMC/@@download/ENCFF495GMC.bed.gz
https://www.encodeproject.org/files/ENCFF150BYS/@@download/ENCFF150BYS.bed.gz
https://www.encodeproject.org/files/ENCFF775AMH/@@download/ENCFF775AMH.bed.gz
https://www.encodeproject.org/files/ENCFF200LDF/@@download/ENCFF200LDF.bed.gz
https://www.encodeproject.org/files/ENCFF243CQL/@@download/ENCFF243CQL.bed.gz
https://www.encodeproject.org/files/ENCFF467WAZ/@@download/ENCFF467WAZ.bed.gz
https://www.encodeproject.org/files/ENCFF606DWB/@@download/ENCFF606DWB.bed.gz
https://www.encodeproject.org/files/ENCFF379TVQ/@@download/ENCFF379TVQ.bed.gz
https://www.encodeproject.org/files/ENCFF014JMW/@@download/ENCFF014JMW.bed.gz
https://www.encodeproject.org/files/ENCFF133YMX/@@download/ENCFF133YMX.bed.gz
https://www.encodeproject.org/files/ENCFF426TDF/@@download/ENCFF426TDF.bed.gz
https://www.encodeproject.org/files/ENCFF719LYG/@@download/ENCFF719LYG.bed.gz
https://www.encodeproject.org/files/ENCFF873YWK/@@download/ENCFF873YWK.bed.gz
https://www.encodeproject.org/files/ENCFF749NSI/@@download/ENCFF749NSI.bed.gz
https://www.encodeproject.org/files/ENCFF189TVG/@@download/ENCFF189TVG.bed.gz
https://www.encodeproject.org/files/ENCFF774TWW/@@download/ENCFF774TWW.bed.gz
https://www.encodeproject.org/files/ENCFF862YTD/@@download/ENCFF862YTD.bed.gz
https://www.encodeproject.org/files/ENCFF868FGE/@@download/ENCFF868FGE.bed.gz
https://www.encodeproject.org/files/ENCFF565RDY/@@download/ENCFF565RDY.bed.gz
https://www.encodeproject.org/files/ENCFF113UWU/@@download/ENCFF113UWU.bed.gz
https://www.encodeproject.org/files/ENCFF759EYE/@@download/ENCFF759EYE.bed.gz
https://www.encodeproject.org/files/ENCFF882UHR/@@download/ENCFF882UHR.bed.gz
https://www.encodeproject.org/files/ENCFF520SXF/@@download/ENCFF520SXF.bed.gz
https://www.encodeproject.org/files/ENCFF376RVN/@@download/ENCFF376RVN.bed.gz
https://www.encodeproject.org/files/ENCFF756LUP/@@download/ENCFF756LUP.bed.gz
https://www.encodeproject.org/files/ENCFF086YFF/@@download/ENCFF086YFF.bed.gz
https://www.encodeproject.org/files/ENCFF079CJA/@@download/ENCFF079CJA.bed.gz
https://www.encodeproject.org/files/ENCFF884TKH/@@download/ENCFF884TKH.bed.gz
https://www.encodeproject.org/files/ENCFF313YXM/@@download/ENCFF313YXM.bed.gz
https://www.encodeproject.org/files/ENCFF566WSG/@@download/ENCFF566WSG.bed.gz
https://www.encodeproject.org/files/ENCFF061SPB/@@download/ENCFF061SPB.bed.gz
https://www.encodeproject.org/files/ENCFF335ZZX/@@download/ENCFF335ZZX.bed.gz
https://www.encodeproject.org/files/ENCFF800NJQ/@@download/ENCFF800NJQ.bed.gz
https://www.encodeproject.org/files/ENCFF150WJN/@@download/ENCFF150WJN.bed.gz
https://www.encodeproject.org/files/ENCFF927HII/@@download/ENCFF927HII.bed.gz
https://www.encodeproject.org/files/ENCFF505BIK/@@download/ENCFF505BIK.bed.gz
https://www.encodeproject.org/files/ENCFF631ZVW/@@download/ENCFF631ZVW.bed.gz
https://www.encodeproject.org/files/ENCFF459TWF/@@download/ENCFF459TWF.bed.gz
https://www.encodeproject.org/files/ENCFF603OCV/@@download/ENCFF603OCV.bed.gz
https://www.encodeproject.org/files/ENCFF026EYC/@@download/ENCFF026EYC.bed.gz
https://www.encodeproject.org/files/ENCFF341DIQ/@@download/ENCFF341DIQ.bed.gz
https://www.encodeproject.org/files/ENCFF039JXQ/@@download/ENCFF039JXQ.bed.gz
https://www.encodeproject.org/files/ENCFF433LTF/@@download/ENCFF433LTF.bed.gz
https://www.encodeproject.org/files/ENCFF669ZUC/@@download/ENCFF669ZUC.bed.gz

https://www.encodeproject.org/files/ENCFF570HDD/@@download/ENCFF570HDD.bed.gz
https://www.encodeproject.org/files/ENCFF912XIE/@@download/ENCFF912XIE.bed.gz
https://www.encodeproject.org/files/ENCFF286IKG/@@download/ENCFF286IKG.bed.gz
https://www.encodeproject.org/files/ENCFF751UAF/@@download/ENCFF751UAF.bed.gz
https://www.encodeproject.org/files/ENCFF001XMK/@@download/ENCFF001XMK.bed.gz
https://www.encodeproject.org/files/ENCFF001XMM/@@download/ENCFF001XMM.bed.gz
https://www.encodeproject.org/files/ENCFF002DCA/@@download/ENCFF002DCA.bed.gz
https://www.encodeproject.org/files/ENCFF387TDH/@@download/ENCFF387TDH.bed.gz
https://www.encodeproject.org/files/ENCFF348AGS/@@download/ENCFF348AGS.bed.gz
https://www.encodeproject.org/files/ENCFF107LKZ/@@download/ENCFF107LKZ.bed.gz
https://www.encodeproject.org/files/ENCFF322FBH/@@download/ENCFF322FBH.bed.gz
https://www.encodeproject.org/files/ENCFF715GFM/@@download/ENCFF715GFM.bed.gz
https://www.encodeproject.org/files/ENCFF241LIY/@@download/ENCFF241LIY.bed.gz
https://www.encodeproject.org/files/ENCFF005RNW/@@download/ENCFF005RNW.bed.gz
https://www.encodeproject.org/files/ENCFF567GAL/@@download/ENCFF567GAL.bed.gz
https://www.encodeproject.org/files/ENCFF337GZT/@@download/ENCFF337GZT.bed.gz
https://www.encodeproject.org/files/ENCFF859PRV/@@download/ENCFF859PRV.bed.gz
https://www.encodeproject.org/files/ENCFF001XPT/@@download/ENCFF001XPT.bed.gz
https://www.encodeproject.org/files/ENCFF001XPU/@@download/ENCFF001XPU.bed.gz
https://www.encodeproject.org/files/ENCFF002DCO/@@download/ENCFF002DCO.bed.gz
https://www.encodeproject.org/files/ENCFF600CYD/@@download/ENCFF600CYD.bed.gz
https://www.encodeproject.org/files/ENCFF777LEG/@@download/ENCFF777LEG.bed.gz
https://www.encodeproject.org/files/ENCFF934HQC/@@download/ENCFF934HQC.bed.gz
https://www.encodeproject.org/files/ENCFF684QJY/@@download/ENCFF684QJY.bed.gz
https://www.encodeproject.org/files/ENCFF183YLB/@@download/ENCFF183YLB.bed.gz
https://www.encodeproject.org/files/ENCFF312HCK/@@download/ENCFF312HCK.bed.gz
https://www.encodeproject.org/files/ENCFF729AWY/@@download/ENCFF729AWY.bed.gz
https://www.encodeproject.org/files/ENCFF994JFX/@@download/ENCFF994JFX.bed.gz
https://www.encodeproject.org/files/ENCFF892VYV/@@download/ENCFF892VYV.bed.gz
https://www.encodeproject.org/files/ENCFF787GLH/@@download/ENCFF787GLH.bed.gz
https://www.encodeproject.org/files/ENCFF779QTR/@@download/ENCFF779QTR.bed.gz
https://www.encodeproject.org/files/ENCFF622LJD/@@download/ENCFF622LJD.bed.gz
https://www.encodeproject.org/files/ENCFF412TMX/@@download/ENCFF412TMX.bed.gz
https://www.encodeproject.org/files/ENCFF636RLY/@@download/ENCFF636RLY.bed.gz
https://www.encodeproject.org/files/ENCFF001VQR/@@download/ENCFF001VQR.bed.gz
https://www.encodeproject.org/files/ENCFF002CZV/@@download/ENCFF002CZV.bed.gz
https://www.encodeproject.org/files/ENCFF904HSP/@@download/ENCFF904HSP.bed.gz
https://www.encodeproject.org/files/ENCFF289ZYB/@@download/ENCFF289ZYB.bed.gz
https://www.encodeproject.org/files/ENCFF799NBQ/@@download/ENCFF799NBQ.bed.gz
https://www.encodeproject.org/files/ENCFF115XBG/@@download/ENCFF115XBG.bed.gz
https://www.encodeproject.org/files/ENCFF180BYN/@@download/ENCFF180BYN.bed.gz
https://www.encodeproject.org/files/ENCFF091ANC/@@download/ENCFF091ANC.bed.gz
https://www.encodeproject.org/files/ENCFF957CWW/@@download/ENCFF957CWW.bed.gz
https://www.encodeproject.org/files/ENCFF372YKO/@@download/ENCFF372YKO.bed.gz
https://www.encodeproject.org/files/ENCFF330USX/@@download/ENCFF330USX.bed.gz
https://www.encodeproject.org/files/ENCFF787KOF/@@download/ENCFF787KOF.bed.gz
https://www.encodeproject.org/files/ENCFF001XSH/@@download/ENCFF001XSH.bed.gz
https://www.encodeproject.org/files/ENCFF001XSI/@@download/ENCFF001XSI.bed.gz
https://www.encodeproject.org/files/ENCFF002DDG/@@download/ENCFF002DDG.bed.gz
https://www.encodeproject.org/files/ENCFF184NDC/@@download/ENCFF184NDC.bed.gz
https://www.encodeproject.org/files/ENCFF975BQZ/@@download/ENCFF975BQZ.bed.gz
https://www.encodeproject.org/files/ENCFF409DTL/@@download/ENCFF409DTL.bed.gz
https://www.encodeproject.org/files/ENCFF237DEX/@@download/ENCFF237DEX.bed.gz

https://www.encodeproject.org/files/ENCFF647XYZ/@@download/ENCFF647XYZ.bed.gz
https://www.encodeproject.org/files/ENCFF139DOR/@@download/ENCFF139DOR.bed.gz
https://www.encodeproject.org/files/ENCFF412BGE/@@download/ENCFF412BGE.bed.gz
https://www.encodeproject.org/files/ENCFF695UND/@@download/ENCFF695UND.bed.gz
https://www.encodeproject.org/files/ENCFF230HWI/@@download/ENCFF230HWI.bed.gz
https://www.encodeproject.org/files/ENCFF246HSO/@@download/ENCFF246HSO.bed.gz
https://www.encodeproject.org/files/ENCFF637OOY/@@download/ENCFF637OOY.bed.gz
https://www.encodeproject.org/files/ENCFF615AFS/@@download/ENCFF615AFS.bed.gz
https://www.encodeproject.org/files/ENCFF333FVM/@@download/ENCFF333FVM.bed.gz
https://www.encodeproject.org/files/ENCFF522DLJ/@@download/ENCFF522DLJ.bed.gz
https://www.encodeproject.org/files/ENCFF858QSY/@@download/ENCFF858QSY.bed.gz
https://www.encodeproject.org/files/ENCFF006YGI/@@download/ENCFF006YGI.bed.gz
https://www.encodeproject.org/files/ENCFF181YOI/@@download/ENCFF181YOI.bed.gz
https://www.encodeproject.org/files/ENCFF715OSJ/@@download/ENCFF715OSJ.bed.gz
https://www.encodeproject.org/files/ENCFF287QRZ/@@download/ENCFF287QRZ.bed.gz
https://www.encodeproject.org/files/ENCFF261IHN/@@download/ENCFF261IHN.bed.gz
https://www.encodeproject.org/files/ENCFF777TZZ/@@download/ENCFF777TZZ.bed.gz
https://www.encodeproject.org/files/ENCFF821KSJ/@@download/ENCFF821KSJ.bed.gz
https://www.encodeproject.org/files/ENCFF987VYY/@@download/ENCFF987VYY.bed.gz
https://www.encodeproject.org/files/ENCFF805FIF/@@download/ENCFF805FIF.bed.gz
https://www.encodeproject.org/files/ENCFF401PJP/@@download/ENCFF401PJP.bed.gz
https://www.encodeproject.org/files/ENCFF207KQM/@@download/ENCFF207KQM.bed.gz
https://www.encodeproject.org/files/ENCFF173JJJ/@@download/ENCFF173JJJ.bed.gz
https://www.encodeproject.org/files/ENCFF182ETN/@@download/ENCFF182ETN.bed.gz
https://www.encodeproject.org/files/ENCFF001XSP/@@download/ENCFF001XSP.bed.gz
https://www.encodeproject.org/files/ENCFF001XSQ/@@download/ENCFF001XSQ.bed.gz
https://www.encodeproject.org/files/ENCFF002DDI/@@download/ENCFF002DDI.bed.gz
https://www.encodeproject.org/files/ENCFF439OCI/@@download/ENCFF439OCI.bed.gz
https://www.encodeproject.org/files/ENCFF645SOL/@@download/ENCFF645SOL.bed.gz
https://www.encodeproject.org/files/ENCFF005IIT/@@download/ENCFF005IIT.bed.gz
https://www.encodeproject.org/files/ENCFF823OGN/@@download/ENCFF823OGN.bed.gz
https://www.encodeproject.org/files/ENCFF032BJW/@@download/ENCFF032BJW.bed.gz
https://www.encodeproject.org/files/ENCFF883QKB/@@download/ENCFF883QKB.bed.gz
https://www.encodeproject.org/files/ENCFF199ZDU/@@download/ENCFF199ZDU.bed.gz
https://www.encodeproject.org/files/ENCFF232MQQ/@@download/ENCFF232MQQ.bed.gz
https://www.encodeproject.org/files/ENCFF569PZS/@@download/ENCFF569PZS.bed.gz
https://www.encodeproject.org/files/ENCFF738CXX/@@download/ENCFF738CXX.bed.gz
https://www.encodeproject.org/files/ENCFF142FYE/@@download/ENCFF142FYE.bed.gz
https://www.encodeproject.org/files/ENCFF665OBP/@@download/ENCFF665OBP.bed.gz
https://www.encodeproject.org/files/ENCFF749PCR/@@download/ENCFF749PCR.bed.gz
https://www.encodeproject.org/files/ENCFF136LAP/@@download/ENCFF136LAP.bed.gz
https://www.encodeproject.org/files/ENCFF267OZD/@@download/ENCFF267OZD.bed.gz
https://www.encodeproject.org/files/ENCFF371GSC/@@download/ENCFF371GSC.bed.gz
https://www.encodeproject.org/files/ENCFF386FNQ/@@download/ENCFF386FNQ.bed.gz
https://www.encodeproject.org/files/ENCFF422YRY/@@download/ENCFF422YRY.bed.gz
https://www.encodeproject.org/files/ENCFF953NJX/@@download/ENCFF953NJX.bed.gz
https://www.encodeproject.org/files/ENCFF570SMG/@@download/ENCFF570SMG.bed.gz
https://www.encodeproject.org/files/ENCFF835RDW/@@download/ENCFF835RDW.bed.gz
https://www.encodeproject.org/files/ENCFF016APK/@@download/ENCFF016APK.bed.gz
https://www.encodeproject.org/files/ENCFF001XTJ/@@download/ENCFF001XTJ.bed.gz
https://www.encodeproject.org/files/ENCFF002DDO/@@download/ENCFF002DDO.bed.gz
https://www.encodeproject.org/files/ENCFF181KEB/@@download/ENCFF181KEB.bed.gz
https://www.encodeproject.org/files/ENCFF148VQH/@@download/ENCFF148VQH.bed.gz

https://www.encodeproject.org/files/ENCFF510CUI/@@download/ENCFF510CUI.bed.gz
https://www.encodeproject.org/files/ENCFF218LOB/@@download/ENCFF218LOB.bed.gz
https://www.encodeproject.org/files/ENCFF306XCP/@@download/ENCFF306XCP.bed.gz
https://www.encodeproject.org/files/ENCFF181QAF/@@download/ENCFF181QAF.bed.gz
https://www.encodeproject.org/files/ENCFF690BYG/@@download/ENCFF690BYG.bed.gz
https://www.encodeproject.org/files/ENCFF234OFQ/@@download/ENCFF234OFQ.bed.gz
https://www.encodeproject.org/files/ENCFF143HEE/@@download/ENCFF143HEE.bed.gz
https://www.encodeproject.org/files/ENCFF322UFN/@@download/ENCFF322UFN.bed.gz
https://www.encodeproject.org/files/ENCFF543EHV/@@download/ENCFF543EHV.bed.gz
https://www.encodeproject.org/files/ENCFF511PPM/@@download/ENCFF511PPM.bed.gz
https://www.encodeproject.org/files/ENCFF403GVA/@@download/ENCFF403GVA.bed.gz
https://www.encodeproject.org/files/ENCFF536RRU/@@download/ENCFF536RRU.bed.gz
https://www.encodeproject.org/files/ENCFF363MKS/@@download/ENCFF363MKS.bed.gz
https://www.encodeproject.org/files/ENCFF709PEA/@@download/ENCFF709PEA.bed.gz
https://www.encodeproject.org/files/ENCFF199GNW/@@download/ENCFF199GNW.bed.gz
https://www.encodeproject.org/files/ENCFF399NTP/@@download/ENCFF399NTP.bed.gz
https://www.encodeproject.org/files/ENCFF088SYH/@@download/ENCFF088SYH.bed.gz
https://www.encodeproject.org/files/ENCFF544XZU/@@download/ENCFF544XZU.bed.gz
https://www.encodeproject.org/files/ENCFF817YDQ/@@download/ENCFF817YDQ.bed.gz
https://www.encodeproject.org/files/ENCFF749CMN/@@download/ENCFF749CMN.bed.gz
https://www.encodeproject.org/files/ENCFF046OHJ/@@download/ENCFF046OHJ.bed.gz
https://www.encodeproject.org/files/ENCFF967EOL/@@download/ENCFF967EOL.bed.gz
https://www.encodeproject.org/files/ENCFF223LYF/@@download/ENCFF223LYF.bed.gz
https://www.encodeproject.org/files/ENCFF262QIB/@@download/ENCFF262QIB.bed.gz
https://www.encodeproject.org/files/ENCFF591HNR/@@download/ENCFF591HNR.bed.gz
https://www.encodeproject.org/files/ENCFF846FIG/@@download/ENCFF846FIG.bed.gz
https://www.encodeproject.org/files/ENCFF634IMR/@@download/ENCFF634IMR.bed.gz
https://www.encodeproject.org/files/ENCFF142JXX/@@download/ENCFF142JXX.bed.gz
https://www.encodeproject.org/files/ENCFF300UJN/@@download/ENCFF300UJN.bed.gz
https://www.encodeproject.org/files/ENCFF112ACP/@@download/ENCFF112ACP.bed.gz
https://www.encodeproject.org/files/ENCFF975SBX/@@download/ENCFF975SBX.bed.gz
https://www.encodeproject.org/files/ENCFF486UBE/@@download/ENCFF486UBE.bed.gz
https://www.encodeproject.org/files/ENCFF755ATB/@@download/ENCFF755ATB.bed.gz
https://www.encodeproject.org/files/ENCFF391YXI/@@download/ENCFF391YXI.bed.gz
https://www.encodeproject.org/files/ENCFF983AVN/@@download/ENCFF983AVN.bed.gz
https://www.encodeproject.org/files/ENCFF092KCB/@@download/ENCFF092KCB.bed.gz
https://www.encodeproject.org/files/ENCFF002CFB/@@download/ENCFF002CFB.bed.gz
https://www.encodeproject.org/files/ENCFF300KXC/@@download/ENCFF300KXC.bed.gz
https://www.encodeproject.org/files/ENCFF547RTC/@@download/ENCFF547RTC.bed.gz
https://www.encodeproject.org/files/ENCFF394RPT/@@download/ENCFF394RPT.bed.gz
https://www.encodeproject.org/files/ENCFF957HGU/@@download/ENCFF957HGU.bed.gz
https://www.encodeproject.org/files/ENCFF841AAT/@@download/ENCFF841AAT.bed.gz
https://www.encodeproject.org/files/ENCFF610UCL/@@download/ENCFF610UCL.bed.gz
https://www.encodeproject.org/files/ENCFF622QGA/@@download/ENCFF622QGA.bed.gz
https://www.encodeproject.org/files/ENCFF263MVV/@@download/ENCFF263MVV.bed.gz
https://www.encodeproject.org/files/ENCFF052ZQB/@@download/ENCFF052ZQB.bed.gz
https://www.encodeproject.org/files/ENCFF770WCW/@@download/ENCFF770WCW.bed.gz
https://www.encodeproject.org/files/ENCFF592GSR/@@download/ENCFF592GSR.bed.gz
https://www.encodeproject.org/files/ENCFF457LLF/@@download/ENCFF457LLF.bed.gz
https://www.encodeproject.org/files/ENCFF808WST/@@download/ENCFF808WST.bed.gz
https://www.encodeproject.org/files/ENCFF963HHX/@@download/ENCFF963HHX.bed.gz
https://www.encodeproject.org/files/ENCFF831MEQ/@@download/ENCFF831MEQ.bed.gz
https://www.encodeproject.org/files/ENCFF145YNF/@@download/ENCFF145YNF.bed.gz

https://www.encodeproject.org/files/ENCFF618LWB/@@download/ENCFF618LWB.bed.gz
https://www.encodeproject.org/files/ENCFF737IIW/@@download/ENCFF737IIW.bed.gz
https://www.encodeproject.org/files/ENCFF305OHM/@@download/ENCFF305OHM.bed.gz
https://www.encodeproject.org/files/ENCFF138OAF/@@download/ENCFF138OAF.bed.gz
https://www.encodeproject.org/files/ENCFF493ZMX/@@download/ENCFF493ZMX.bed.gz
https://www.encodeproject.org/files/ENCFF392UBG/@@download/ENCFF392UBG.bed.gz
https://www.encodeproject.org/files/ENCFF327VVW/@@download/ENCFF327VVW.bed.gz
https://www.encodeproject.org/files/ENCFF151FBW/@@download/ENCFF151FBW.bed.gz
https://www.encodeproject.org/files/ENCFF810FLM/@@download/ENCFF810FLM.bed.gz
https://www.encodeproject.org/files/ENCFF981QWW/@@download/ENCFF981QWW.bed.gz
https://www.encodeproject.org/files/ENCFF399DQN/@@download/ENCFF399DQN.bed.gz
https://www.encodeproject.org/files/ENCFF840NOO/@@download/ENCFF840NOO.bed.gz
https://www.encodeproject.org/files/ENCFF082LUY/@@download/ENCFF082LUY.bed.gz
https://www.encodeproject.org/files/ENCFF079TMN/@@download/ENCFF079TMN.bed.gz
https://www.encodeproject.org/files/ENCFF478WOV/@@download/ENCFF478WOV.bed.gz
https://www.encodeproject.org/files/ENCFF451GFT/@@download/ENCFF451GFT.bed.gz
https://www.encodeproject.org/files/ENCFF438LQP/@@download/ENCFF438LQP.bed.gz
https://www.encodeproject.org/files/ENCFF003CMQ/@@download/ENCFF003CMQ.bed.gz
https://www.encodeproject.org/files/ENCFF308TCJ/@@download/ENCFF308TCJ.bed.gz
https://www.encodeproject.org/files/ENCFF717PMK/@@download/ENCFF717PMK.bed.gz
https://www.encodeproject.org/files/ENCFF001VLL/@@download/ENCFF001VLL.bed.gz
https://www.encodeproject.org/files/ENCFF002CVF/@@download/ENCFF002CVF.bed.gz
https://www.encodeproject.org/files/ENCFF005FQK/@@download/ENCFF005FQK.bed.gz
https://www.encodeproject.org/files/ENCFF407FUL/@@download/ENCFF407FUL.bed.gz
https://www.encodeproject.org/files/ENCFF071RNS/@@download/ENCFF071RNS.bed.gz
https://www.encodeproject.org/files/ENCFF237VLQ/@@download/ENCFF237VLQ.bed.gz
https://www.encodeproject.org/files/ENCFF867EUD/@@download/ENCFF867EUD.bed.gz
https://www.encodeproject.org/files/ENCFF437YNA/@@download/ENCFF437YNA.bed.gz
https://www.encodeproject.org/files/ENCFF741PQE/@@download/ENCFF741PQE.bed.gz
https://www.encodeproject.org/files/ENCFF459FMQ/@@download/ENCFF459FMQ.bed.gz
https://www.encodeproject.org/files/ENCFF324QGE/@@download/ENCFF324QGE.bed.gz
https://www.encodeproject.org/files/ENCFF841RCY/@@download/ENCFF841RCY.bed.gz
https://www.encodeproject.org/files/ENCFF207YNL/@@download/ENCFF207YNL.bed.gz
https://www.encodeproject.org/files/ENCFF712EUK/@@download/ENCFF712EUK.bed.gz
https://www.encodeproject.org/files/ENCFF924RTU/@@download/ENCFF924RTU.bed.gz
https://www.encodeproject.org/files/ENCFF832YJX/@@download/ENCFF832YJX.bed.gz
https://www.encodeproject.org/files/ENCFF629UPV/@@download/ENCFF629UPV.bed.gz
https://www.encodeproject.org/files/ENCFF809RZH/@@download/ENCFF809RZH.bed.gz
https://www.encodeproject.org/files/ENCFF868DFG/@@download/ENCFF868DFG.bed.gz
https://www.encodeproject.org/files/ENCFF560XSC/@@download/ENCFF560XSC.bed.gz
https://www.encodeproject.org/files/ENCFF369LXG/@@download/ENCFF369LXG.bed.gz
https://www.encodeproject.org/files/ENCFF578ZTX/@@download/ENCFF578ZTX.bed.gz
https://www.encodeproject.org/files/ENCFF663YRF/@@download/ENCFF663YRF.bed.gz
https://www.encodeproject.org/files/ENCFF898JJD/@@download/ENCFF898JJD.bed.gz
https://www.encodeproject.org/files/ENCFF001USZ/@@download/ENCFF001USZ.bed.gz
https://www.encodeproject.org/files/ENCFF002DAZ/@@download/ENCFF002DAZ.bed.gz
https://www.encodeproject.org/files/ENCFF353AUC/@@download/ENCFF353AUC.bed.gz
https://www.encodeproject.org/files/ENCFF541ERF/@@download/ENCFF541ERF.bed.gz
https://www.encodeproject.org/files/ENCFF199TXU/@@download/ENCFF199TXU.bed.gz
https://www.encodeproject.org/files/ENCFF457PBA/@@download/ENCFF457PBA.bed.gz
https://www.encodeproject.org/files/ENCFF422OZA/@@download/ENCFF422OZA.bed.gz
https://www.encodeproject.org/files/ENCFF638MFT/@@download/ENCFF638MFT.bed.gz
https://www.encodeproject.org/files/ENCFF626AFW/@@download/ENCFF626AFW.bed.gz

https://www.encodeproject.org/files/ENCFF579MSA/@@download/ENCFF579MSA.bed.gz
https://www.encodeproject.org/files/ENCFF033XFJ/@@download/ENCFF033XFJ.bed.gz
https://www.encodeproject.org/files/ENCFF334LTK/@@download/ENCFF334LTK.bed.gz
https://www.encodeproject.org/files/ENCFF363GNR/@@download/ENCFF363GNR.bed.gz
https://www.encodeproject.org/files/ENCFF089HBQ/@@download/ENCFF089HBQ.bed.gz
https://www.encodeproject.org/files/ENCFF735EHK/@@download/ENCFF735EHK.bed.gz
https://www.encodeproject.org/files/ENCFF586WMF/@@download/ENCFF586WMF.bed.gz
https://www.encodeproject.org/files/ENCFF505WZO/@@download/ENCFF505WZO.bed.gz
https://www.encodeproject.org/files/ENCFF725FJK/@@download/ENCFF725FJK.bed.gz
https://www.encodeproject.org/files/ENCFF240CUH/@@download/ENCFF240CUH.bed.gz
https://www.encodeproject.org/files/ENCFF761JIU/@@download/ENCFF761JIU.bed.gz
https://www.encodeproject.org/files/ENCFF946ALH/@@download/ENCFF946ALH.bed.gz
https://www.encodeproject.org/files/ENCFF661IIS/@@download/ENCFF661IIS.bed.gz
https://www.encodeproject.org/files/ENCFF344AQO/@@download/ENCFF344AQO.bed.gz
https://www.encodeproject.org/files/ENCFF887AQJ/@@download/ENCFF887AQJ.bed.gz
https://www.encodeproject.org/files/ENCFF276RMO/@@download/ENCFF276RMO.bed.gz
https://www.encodeproject.org/files/ENCFF698WAX/@@download/ENCFF698WAX.bed.gz
https://www.encodeproject.org/files/ENCFF011NRT/@@download/ENCFF011NRT.bed.gz
https://www.encodeproject.org/files/ENCFF759LYG/@@download/ENCFF759LYG.bed.gz
https://www.encodeproject.org/files/ENCFF234SFH/@@download/ENCFF234SFH.bed.gz
https://www.encodeproject.org/files/ENCFF305CKS/@@download/ENCFF305CKS.bed.gz
https://www.encodeproject.org/files/ENCFF734LKN/@@download/ENCFF734LKN.bed.gz
https://www.encodeproject.org/files/ENCFF712LFQ/@@download/ENCFF712LFQ.bed.gz
https://www.encodeproject.org/files/ENCFF232YWT/@@download/ENCFF232YWT.bed.gz
https://www.encodeproject.org/files/ENCFF509HVO/@@download/ENCFF509HVO.bed.gz
https://www.encodeproject.org/files/ENCFF691KZX/@@download/ENCFF691KZX.bed.gz
https://www.encodeproject.org/files/ENCFF933GGM/@@download/ENCFF933GGM.bed.gz
https://www.encodeproject.org/files/ENCFF060WTK/@@download/ENCFF060WTK.bed.gz
https://www.encodeproject.org/files/ENCFF007ACV/@@download/ENCFF007ACV.bed.gz
https://www.encodeproject.org/files/ENCFF633IHZ/@@download/ENCFF633IHZ.bed.gz
https://www.encodeproject.org/files/ENCFF244MXM/@@download/ENCFF244MXM.bed.gz
https://www.encodeproject.org/files/ENCFF239HMM/@@download/ENCFF239HMM.bed.gz
https://www.encodeproject.org/files/ENCFF908BOL/@@download/ENCFF908BOL.bed.gz
https://www.encodeproject.org/files/ENCFF450XID/@@download/ENCFF450XID.bed.gz
https://www.encodeproject.org/files/ENCFF176MPT/@@download/ENCFF176MPT.bed.gz
https://www.encodeproject.org/files/ENCFF099LBC/@@download/ENCFF099LBC.bed.gz
https://www.encodeproject.org/files/ENCFF508LRF/@@download/ENCFF508LRF.bed.gz
https://www.encodeproject.org/files/ENCFF001XRN/@@download/ENCFF001XRN.bed.gz
https://www.encodeproject.org/files/ENCFF001XRO/@@download/ENCFF001XRO.bed.gz
https://www.encodeproject.org/files/ENCFF002DDB/@@download/ENCFF002DDB.bed.gz
https://www.encodeproject.org/files/ENCFF493HJH/@@download/ENCFF493HJH.bed.gz
https://www.encodeproject.org/files/ENCFF826PZY/@@download/ENCFF826PZY.bed.gz
https://www.encodeproject.org/files/ENCFF214JJE/@@download/ENCFF214JJE.bed.gz
https://www.encodeproject.org/files/ENCFF839YLN/@@download/ENCFF839YLN.bed.gz
https://www.encodeproject.org/files/ENCFF288RFS/@@download/ENCFF288RFS.bed.gz
https://www.encodeproject.org/files/ENCFF924YJI/@@download/ENCFF924YJI.bed.gz
https://www.encodeproject.org/files/ENCFF929WKT/@@download/ENCFF929WKT.bed.gz
https://www.encodeproject.org/files/ENCFF938HQH/@@download/ENCFF938HQH.bed.gz
https://www.encodeproject.org/files/ENCFF430AWA/@@download/ENCFF430AWA.bed.gz
https://www.encodeproject.org/files/ENCFF818LBB/@@download/ENCFF818LBB.bed.gz
https://www.encodeproject.org/files/ENCFF954SMI/@@download/ENCFF954SMI.bed.gz
https://www.encodeproject.org/files/ENCFF818GNJ/@@download/ENCFF818GNJ.bed.gz
https://www.encodeproject.org/files/ENCFF379TEL/@@download/ENCFF379TEL.bed.gz

https://www.encodeproject.org/files/ENCFF279FQM/@@download/ENCFF279FQM.bed.gz
https://www.encodeproject.org/files/ENCFF035MJS/@@download/ENCFF035MJS.bed.gz
https://www.encodeproject.org/files/ENCFF113NNM/@@download/ENCFF113NNM.bed.gz
https://www.encodeproject.org/files/ENCFF371RAL/@@download/ENCFF371RAL.bed.gz
https://www.encodeproject.org/files/ENCFF815CRR/@@download/ENCFF815CRR.bed.gz
https://www.encodeproject.org/files/ENCFF575LSG/@@download/ENCFF575LSG.bed.gz
https://www.encodeproject.org/files/ENCFF420GLO/@@download/ENCFF420GLO.bed.gz
https://www.encodeproject.org/files/ENCFF572MCI/@@download/ENCFF572MCI.bed.gz
https://www.encodeproject.org/files/ENCFF020HWP/@@download/ENCFF020HWP.bed.gz
https://www.encodeproject.org/files/ENCFF328XQD/@@download/ENCFF328XQD.bed.gz
https://www.encodeproject.org/files/ENCFF002OGO/@@download/ENCFF002OGO.bed.gz
https://www.encodeproject.org/files/ENCFF458DFL/@@download/ENCFF458DFL.bed.gz
https://www.encodeproject.org/files/ENCFF651LIG/@@download/ENCFF651LIG.bed.gz
https://www.encodeproject.org/files/ENCFF737IOU/@@download/ENCFF737IOU.bed.gz
https://www.encodeproject.org/files/ENCFF161OVA/@@download/ENCFF161OVA.bed.gz
https://www.encodeproject.org/files/ENCFF906CSG/@@download/ENCFF906CSG.bed.gz
https://www.encodeproject.org/files/ENCFF894RYL/@@download/ENCFF894RYL.bed.gz
https://www.encodeproject.org/files/ENCFF973ISL/@@download/ENCFF973ISL.bed.gz
https://www.encodeproject.org/files/ENCFF927QZZ/@@download/ENCFF927QZZ.bed.gz
https://www.encodeproject.org/files/ENCFF208JPL/@@download/ENCFF208JPL.bed.gz
https://www.encodeproject.org/files/ENCFF688KFE/@@download/ENCFF688KFE.bed.gz
https://www.encodeproject.org/files/ENCFF624XKD/@@download/ENCFF624XKD.bed.gz
https://www.encodeproject.org/files/ENCFF950LOX/@@download/ENCFF950LOX.bed.gz
https://www.encodeproject.org/files/ENCFF974XKH/@@download/ENCFF974XKH.bed.gz
https://www.encodeproject.org/files/ENCFF443IDR/@@download/ENCFF443IDR.bed.gz
https://www.encodeproject.org/files/ENCFF645KBN/@@download/ENCFF645KBN.bed.gz
https://www.encodeproject.org/files/ENCFF063UKS/@@download/ENCFF063UKS.bed.gz
https://www.encodeproject.org/files/ENCFF556AEV/@@download/ENCFF556AEV.bed.gz
https://www.encodeproject.org/files/ENCFF295GOD/@@download/ENCFF295GOD.bed.gz
https://www.encodeproject.org/files/ENCFF427XJF/@@download/ENCFF427XJF.bed.gz
https://www.encodeproject.org/files/ENCFF708GMO/@@download/ENCFF708GMO.bed.gz
https://www.encodeproject.org/files/ENCFF002CEI/@@download/ENCFF002CEI.bed.gz
https://www.encodeproject.org/files/ENCFF671LMY/@@download/ENCFF671LMY.bed.gz
https://www.encodeproject.org/files/ENCFF411FEE/@@download/ENCFF411FEE.bed.gz
https://www.encodeproject.org/files/ENCFF491JFL/@@download/ENCFF491JFL.bed.gz
https://www.encodeproject.org/files/ENCFF615HHV/@@download/ENCFF615HHV.bed.gz
https://www.encodeproject.org/files/ENCFF168INE/@@download/ENCFF168INE.bed.gz
https://www.encodeproject.org/files/ENCFF703VGN/@@download/ENCFF703VGN.bed.gz
https://www.encodeproject.org/files/ENCFF414TMK/@@download/ENCFF414TMK.bed.gz
https://www.encodeproject.org/files/ENCFF517GNM/@@download/ENCFF517GNM.bed.gz
https://www.encodeproject.org/files/ENCFF154XEH/@@download/ENCFF154XEH.bed.gz
https://www.encodeproject.org/files/ENCFF172QSW/@@download/ENCFF172QSW.bed.gz
https://www.encodeproject.org/files/ENCFF461OKL/@@download/ENCFF461OKL.bed.gz
https://www.encodeproject.org/files/ENCFF819WNB/@@download/ENCFF819WNB.bed.gz
https://www.encodeproject.org/files/ENCFF643EJZ/@@download/ENCFF643EJZ.bed.gz
https://www.encodeproject.org/files/ENCFF043HEN/@@download/ENCFF043HEN.bed.gz
https://www.encodeproject.org/files/ENCFF486IPS/@@download/ENCFF486IPS.bed.gz
https://www.encodeproject.org/files/ENCFF958ZSA/@@download/ENCFF958ZSA.bed.gz
https://www.encodeproject.org/files/ENCFF503UCK/@@download/ENCFF503UCK.bed.gz
https://www.encodeproject.org/files/ENCFF654MRF/@@download/ENCFF654MRF.bed.gz
https://www.encodeproject.org/files/ENCFF844KZH/@@download/ENCFF844KZH.bed.gz
https://www.encodeproject.org/files/ENCFF746ZUT/@@download/ENCFF746ZUT.bed.gz
https://www.encodeproject.org/files/ENCFF240UFV/@@download/ENCFF240UFV.bed.gz

https://www.encodeproject.org/files/ENCFF852FMV/@@download/ENCFF852FMV.bed.gz
https://www.encodeproject.org/files/ENCFF552XDP/@@download/ENCFF552XDP.bed.gz
https://www.encodeproject.org/files/ENCFF001VLI/@@download/ENCFF001VLI.bed.gz
https://www.encodeproject.org/files/ENCFF002CVC/@@download/ENCFF002CVC.bed.gz
https://www.encodeproject.org/files/ENCFF675VWT/@@download/ENCFF675VWT.bed.gz
https://www.encodeproject.org/files/ENCFF635MUK/@@download/ENCFF635MUK.bed.gz
https://www.encodeproject.org/files/ENCFF621LUI/@@download/ENCFF621LUI.bed.gz
https://www.encodeproject.org/files/ENCFF608UAY/@@download/ENCFF608UAY.bed.gz
https://www.encodeproject.org/files/ENCFF695DXW/@@download/ENCFF695DXW.bed.gz
https://www.encodeproject.org/files/ENCFF574WKH/@@download/ENCFF574WKH.bed.gz
https://www.encodeproject.org/files/ENCFF698JRH/@@download/ENCFF698JRH.bed.gz
https://www.encodeproject.org/files/ENCFF218PDI/@@download/ENCFF218PDI.bed.gz
https://www.encodeproject.org/files/ENCFF845XIS/@@download/ENCFF845XIS.bed.gz
https://www.encodeproject.org/files/ENCFF820IFR/@@download/ENCFF820IFR.bed.gz
https://www.encodeproject.org/files/ENCFF344XWK/@@download/ENCFF344XWK.bed.gz
https://www.encodeproject.org/files/ENCFF661JYP/@@download/ENCFF661JYP.bed.gz
https://www.encodeproject.org/files/ENCFF030PHB/@@download/ENCFF030PHB.bed.gz
https://www.encodeproject.org/files/ENCFF314VOK/@@download/ENCFF314VOK.bed.gz
https://www.encodeproject.org/files/ENCFF236XBR/@@download/ENCFF236XBR.bed.gz
https://www.encodeproject.org/files/ENCFF808RKL/@@download/ENCFF808RKL.bed.gz
https://www.encodeproject.org/files/ENCFF698DYH/@@download/ENCFF698DYH.bed.gz
https://www.encodeproject.org/files/ENCFF985ZJL/@@download/ENCFF985ZJL.bed.gz
https://www.encodeproject.org/files/ENCFF965ZSY/@@download/ENCFF965ZSY.bed.gz
https://www.encodeproject.org/files/ENCFF174CEI/@@download/ENCFF174CEI.bed.gz
https://www.encodeproject.org/files/ENCFF001XQH/@@download/ENCFF001XQH.bed.gz
https://www.encodeproject.org/files/ENCFF001XQI/@@download/ENCFF001XQI.bed.gz
https://www.encodeproject.org/files/ENCFF002DCS/@@download/ENCFF002DCS.bed.gz
https://www.encodeproject.org/files/ENCFF727MVC/@@download/ENCFF727MVC.bed.gz
https://www.encodeproject.org/files/ENCFF993NKI/@@download/ENCFF993NKI.bed.gz
https://www.encodeproject.org/files/ENCFF730JDO/@@download/ENCFF730JDO.bed.gz
https://www.encodeproject.org/files/ENCFF136ZAK/@@download/ENCFF136ZAK.bed.gz
https://www.encodeproject.org/files/ENCFF009MRG/@@download/ENCFF009MRG.bed.gz
https://www.encodeproject.org/files/ENCFF680YPK/@@download/ENCFF680YPK.bed.gz
https://www.encodeproject.org/files/ENCFF507VHP/@@download/ENCFF507VHP.bed.gz
https://www.encodeproject.org/files/ENCFF700ILD/@@download/ENCFF700ILD.bed.gz
https://www.encodeproject.org/files/ENCFF927GGF/@@download/ENCFF927GGF.bed.gz
https://www.encodeproject.org/files/ENCFF587LBC/@@download/ENCFF587LBC.bed.gz
https://www.encodeproject.org/files/ENCFF135EPC/@@download/ENCFF135EPC.bed.gz
https://www.encodeproject.org/files/ENCFF505VMB/@@download/ENCFF505VMB.bed.gz
https://www.encodeproject.org/files/ENCFF856FKS/@@download/ENCFF856FKS.bed.gz
https://www.encodeproject.org/files/ENCFF148LAV/@@download/ENCFF148LAV.bed.gz
https://www.encodeproject.org/files/ENCFF102XCU/@@download/ENCFF102XCU.bed.gz
https://www.encodeproject.org/files/ENCFF854YVQ/@@download/ENCFF854YVQ.bed.gz
https://www.encodeproject.org/files/ENCFF010BAD/@@download/ENCFF010BAD.bed.gz
https://www.encodeproject.org/files/ENCFF971ILA/@@download/ENCFF971ILA.bed.gz
https://www.encodeproject.org/files/ENCFF204QPK/@@download/ENCFF204QPK.bed.gz
https://www.encodeproject.org/files/ENCFF687WWO/@@download/ENCFF687WWO.bed.gz
https://www.encodeproject.org/files/ENCFF488NTO/@@download/ENCFF488NTO.bed.gz
https://www.encodeproject.org/files/ENCFF591BNO/@@download/ENCFF591BNO.bed.gz
https://www.encodeproject.org/files/ENCFF054GYB/@@download/ENCFF054GYB.bed.gz
https://www.encodeproject.org/files/ENCFF172IAS/@@download/ENCFF172IAS.bed.gz
https://www.encodeproject.org/files/ENCFF229COM/@@download/ENCFF229COM.bed.gz
https://www.encodeproject.org/files/ENCFF373EUK/@@download/ENCFF373EUK.bed.gz

Supplementary Table 1

https://www.encodeproject.org/files/ENCFF436DJC/@@download/ENCFF436DJC.bed.gz
https://www.encodeproject.org/files/ENCFF301CFJ/@@download/ENCFF301CFJ.bed.gz
https://www.encodeproject.org/files/ENCFF133FXT/@@download/ENCFF133FXT.bed.gz
https://www.encodeproject.org/files/ENCFF804UDG/@@download/ENCFF804UDG.bed.gz
https://www.encodeproject.org/files/ENCFF495YBQ/@@download/ENCFF495YBQ.bed.gz
https://www.encodeproject.org/files/ENCFF981JOH/@@download/ENCFF981JOH.bed.gz
https://www.encodeproject.org/files/ENCFF049JNK/@@download/ENCFF049JNK.bed.gz
https://www.encodeproject.org/files/ENCFF835VBA/@@download/ENCFF835VBA.bed.gz
https://www.encodeproject.org/files/ENCFF001UTB/@@download/ENCFF001UTB.bed.gz
https://www.encodeproject.org/files/ENCFF002DBA/@@download/ENCFF002DBA.bed.gz
https://www.encodeproject.org/files/ENCFF458RGL/@@download/ENCFF458RGL.bed.gz
https://www.encodeproject.org/files/ENCFF887ONX/@@download/ENCFF887ONX.bed.gz
https://www.encodeproject.org/files/ENCFF977AFL/@@download/ENCFF977AFL.bed.gz
https://www.encodeproject.org/files/ENCFF691ARB/@@download/ENCFF691ARB.bed.gz
https://www.encodeproject.org/files/ENCFF728JUW/@@download/ENCFF728JUW.bed.gz
https://www.encodeproject.org/files/ENCFF517HGL/@@download/ENCFF517HGL.bed.gz
https://www.encodeproject.org/files/ENCFF217FNA/@@download/ENCFF217FNA.bed.gz
https://www.encodeproject.org/files/ENCFF668UDC/@@download/ENCFF668UDC.bed.gz
https://www.encodeproject.org/files/ENCFF673VPE/@@download/ENCFF673VPE.bed.gz
https://www.encodeproject.org/files/ENCFF477KMO/@@download/ENCFF477KMO.bed.gz
https://www.encodeproject.org/files/ENCFF957IBE/@@download/ENCFF957IBE.bed.gz
https://www.encodeproject.org/files/ENCFF639EJK/@@download/ENCFF639EJK.bed.gz
https://www.encodeproject.org/files/ENCFF837QHJ/@@download/ENCFF837QHJ.bed.gz
https://www.encodeproject.org/files/ENCFF618OVE/@@download/ENCFF618OVE.bed.gz
https://www.encodeproject.org/files/ENCFF901DDA/@@download/ENCFF901DDA.bed.gz
https://www.encodeproject.org/files/ENCFF088ETE/@@download/ENCFF088ETE.bed.gz
https://www.encodeproject.org/files/ENCFF201MOM/@@download/ENCFF201MOM.bed.gz
https://www.encodeproject.org/files/ENCFF562IOY/@@download/ENCFF562IOY.bed.gz
https://www.encodeproject.org/files/ENCFF844HAN/@@download/ENCFF844HAN.bed.gz
https://www.encodeproject.org/files/ENCFF864HFH/@@download/ENCFF864HFH.bed.gz
https://www.encodeproject.org/files/ENCFF928ZSB/@@download/ENCFF928ZSB.bed.gz
https://www.encodeproject.org/files/ENCFF954XKP/@@download/ENCFF954XKP.bed.gz
https://www.encodeproject.org/files/ENCFF155UYD/@@download/ENCFF155UYD.bed.gz
https://www.encodeproject.org/files/ENCFF191HFX/@@download/ENCFF191HFX.bed.gz
https://www.encodeproject.org/files/ENCFF297BUU/@@download/ENCFF297BUU.bed.gz
https://www.encodeproject.org/files/ENCFF433EFF/@@download/ENCFF433EFF.bed.gz
https://www.encodeproject.org/files/ENCFF842FFW/@@download/ENCFF842FFW.bed.gz
https://www.encodeproject.org/files/ENCFF571PKW/@@download/ENCFF571PKW.bed.gz
https://www.encodeproject.org/files/ENCFF139YFA/@@download/ENCFF139YFA.bed.gz
https://www.encodeproject.org/files/ENCFF084INO/@@download/ENCFF084INO.bed.gz
https://www.encodeproject.org/files/ENCFF017YUI/@@download/ENCFF017YUI.bed.gz
https://www.encodeproject.org/files/ENCFF027RDL/@@download/ENCFF027RDL.bed.gz
https://www.encodeproject.org/files/ENCFF039SAY/@@download/ENCFF039SAY.bed.gz
https://www.encodeproject.org/files/ENCFF468WHP/@@download/ENCFF468WHP.bed.gz
https://www.encodeproject.org/files/ENCFF933XOI/@@download/ENCFF933XOI.bed.gz
https://www.encodeproject.org/files/ENCFF825XAC/@@download/ENCFF825XAC.bed.gz
https://www.encodeproject.org/files/ENCFF122UUO/@@download/ENCFF122UUO.bed.gz
https://www.encodeproject.org/files/ENCFF073AVA/@@download/ENCFF073AVA.bed.gz
https://www.encodeproject.org/files/ENCFF794MZC/@@download/ENCFF794MZC.bed.gz
https://www.encodeproject.org/files/ENCFF912ZJS/@@download/ENCFF912ZJS.bed.gz
https://www.encodeproject.org/files/ENCFF411YXA/@@download/ENCFF411YXA.bed.gz
https://www.encodeproject.org/files/ENCFF276XLL/@@download/ENCFF276XLL.bed.gz
https://www.encodeproject.org/files/ENCFF223DJX/@@download/ENCFF223DJX.bed.gz

Supplementary Table 1

https://www.encodeproject.org/files/ENCFF229NAV/@@download/ENCFF229NAV.bed.gz
https://www.encodeproject.org/files/ENCFF607YLT/@@download/ENCFF607YLT.bed.gz
https://www.encodeproject.org/files/ENCFF872PLP/@@download/ENCFF872PLP.bed.gz
https://www.encodeproject.org/files/ENCFF614UCN/@@download/ENCFF614UCN.bed.gz
https://www.encodeproject.org/files/ENCFF433ZKP/@@download/ENCFF433ZKP.bed.gz
https://www.encodeproject.org/files/ENCFF362XGP/@@download/ENCFF362XGP.bed.gz
https://www.encodeproject.org/files/ENCFF415VAZ/@@download/ENCFF415VAZ.bed.gz
https://www.encodeproject.org/files/ENCFF614GUV/@@download/ENCFF614GUV.bed.gz
https://www.encodeproject.org/files/ENCFF823LVL/@@download/ENCFF823LVL.bed.gz
https://www.encodeproject.org/files/ENCFF951VPZ/@@download/ENCFF951VPZ.bed.gz
https://www.encodeproject.org/files/ENCFF972ILN/@@download/ENCFF972ILN.bed.gz
https://www.encodeproject.org/files/ENCFF584JHM/@@download/ENCFF584JHM.bed.gz
https://www.encodeproject.org/files/ENCFF126PNX/@@download/ENCFF126PNX.bed.gz
https://www.encodeproject.org/files/ENCFF971SJB/@@download/ENCFF971SJB.bed.gz
https://www.encodeproject.org/files/ENCFF363RKC/@@download/ENCFF363RKC.bed.gz
https://www.encodeproject.org/files/ENCFF651MBI/@@download/ENCFF651MBI.bed.gz
https://www.encodeproject.org/files/ENCFF003GWJ/@@download/ENCFF003GWJ.bed.gz
https://www.encodeproject.org/files/ENCFF129GGF/@@download/ENCFF129GGF.bed.gz
https://www.encodeproject.org/files/ENCFF189LVJ/@@download/ENCFF189LVJ.bed.gz
https://www.encodeproject.org/files/ENCFF971QHN/@@download/ENCFF971QHN.bed.gz
https://www.encodeproject.org/files/ENCFF955CAT/@@download/ENCFF955CAT.bed.gz
https://www.encodeproject.org/files/ENCFF741KXN/@@download/ENCFF741KXN.bed.gz
https://www.encodeproject.org/files/ENCFF765FDF/@@download/ENCFF765FDF.bed.gz
https://www.encodeproject.org/files/ENCFF512ZCE/@@download/ENCFF512ZCE.bed.gz
https://www.encodeproject.org/files/ENCFF593OTD/@@download/ENCFF593OTD.bed.gz
https://www.encodeproject.org/files/ENCFF173XPM/@@download/ENCFF173XPM.bed.gz
https://www.encodeproject.org/files/ENCFF497MUF/@@download/ENCFF497MUF.bed.gz
https://www.encodeproject.org/files/ENCFF185IMR/@@download/ENCFF185IMR.bed.gz
https://www.encodeproject.org/files/ENCFF862NGG/@@download/ENCFF862NGG.bed.gz
https://www.encodeproject.org/files/ENCFF029BXB/@@download/ENCFF029BXB.bed.gz
https://www.encodeproject.org/files/ENCFF696JKX/@@download/ENCFF696JKX.bed.gz
https://www.encodeproject.org/files/ENCFF979TJN/@@download/ENCFF979TJN.bed.gz
https://www.encodeproject.org/files/ENCFF414ZTT/@@download/ENCFF414ZTT.bed.gz
https://www.encodeproject.org/files/ENCFF579XTC/@@download/ENCFF579XTC.bed.gz
https://www.encodeproject.org/files/ENCFF515NYK/@@download/ENCFF515NYK.bed.gz
https://www.encodeproject.org/files/ENCFF811WQQ/@@download/ENCFF811WQQ.bed.gz
https://www.encodeproject.org/files/ENCFF025EPQ/@@download/ENCFF025EPQ.bed.gz
https://www.encodeproject.org/files/ENCFF967QUY/@@download/ENCFF967QUY.bed.gz
https://www.encodeproject.org/files/ENCFF566FVB/@@download/ENCFF566FVB.bed.gz
https://www.encodeproject.org/files/ENCFF128ZQH/@@download/ENCFF128ZQH.bed.gz
https://www.encodeproject.org/files/ENCFF853ZNE/@@download/ENCFF853ZNE.bed.gz
https://www.encodeproject.org/files/ENCFF315FCJ/@@download/ENCFF315FCJ.bed.gz
https://www.encodeproject.org/files/ENCFF146NMG/@@download/ENCFF146NMG.bed.gz
https://www.encodeproject.org/files/ENCFF782SGI/@@download/ENCFF782SGI.bed.gz
https://www.encodeproject.org/files/ENCFF614BYF/@@download/ENCFF614BYF.bed.gz
https://www.encodeproject.org/files/ENCFF655AOY/@@download/ENCFF655AOY.bed.gz
https://www.encodeproject.org/files/ENCFF996MTB/@@download/ENCFF996MTB.bed.gz
https://www.encodeproject.org/files/ENCFF966CBP/@@download/ENCFF966CBP.bed.gz
https://www.encodeproject.org/files/ENCFF963GDB/@@download/ENCFF963GDB.bed.gz
https://www.encodeproject.org/files/ENCFF637JPV/@@download/ENCFF637JPV.bed.gz
https://www.encodeproject.org/files/ENCFF653ILB/@@download/ENCFF653ILB.bed.gz
https://www.encodeproject.org/files/ENCFF401DJJ/@@download/ENCFF401DJJ.bed.gz
https://www.encodeproject.org/files/ENCFF788JPL/@@download/ENCFF788JPL.bed.gz

https://www.encodeproject.org/files/ENCFF645DIC/@@download/ENCFF645DIC.bed.gz
https://www.encodeproject.org/files/ENCFF538YID/@@download/ENCFF538YID.bed.gz
https://www.encodeproject.org/files/ENCFF326UJU/@@download/ENCFF326UJU.bed.gz
https://www.encodeproject.org/files/ENCFF157FXA/@@download/ENCFF157FXA.bed.gz
https://www.encodeproject.org/files/ENCFF849CHG/@@download/ENCFF849CHG.bed.gz
https://www.encodeproject.org/files/ENCFF739MCU/@@download/ENCFF739MCU.bed.gz
https://www.encodeproject.org/files/ENCFF603GGT/@@download/ENCFF603GGT.bed.gz
https://www.encodeproject.org/files/ENCFF905CUU/@@download/ENCFF905CUU.bed.gz
https://www.encodeproject.org/files/ENCFF627DHJ/@@download/ENCFF627DHJ.bed.gz
https://www.encodeproject.org/files/ENCFF872LIM/@@download/ENCFF872LIM.bed.gz
https://www.encodeproject.org/files/ENCFF114FNT/@@download/ENCFF114FNT.bed.gz
https://www.encodeproject.org/files/ENCFF814SXM/@@download/ENCFF814SXM.bed.gz
https://www.encodeproject.org/files/ENCFF721JKB/@@download/ENCFF721JKB.bed.gz
https://www.encodeproject.org/files/ENCFF033XQB/@@download/ENCFF033XQB.bed.gz
https://www.encodeproject.org/files/ENCFF572ESJ/@@download/ENCFF572ESJ.bed.gz
https://www.encodeproject.org/files/ENCFF797OLU/@@download/ENCFF797OLU.bed.gz
https://www.encodeproject.org/files/ENCFF657HTU/@@download/ENCFF657HTU.bed.gz
https://www.encodeproject.org/files/ENCFF051USR/@@download/ENCFF051USR.bed.gz
https://www.encodeproject.org/files/ENCFF043PIW/@@download/ENCFF043PIW.bed.gz
https://www.encodeproject.org/files/ENCFF719VDM/@@download/ENCFF719VDM.bed.gz
https://www.encodeproject.org/files/ENCFF960NYN/@@download/ENCFF960NYN.bed.gz
https://www.encodeproject.org/files/ENCFF968MST/@@download/ENCFF968MST.bed.gz
https://www.encodeproject.org/files/ENCFF086GJP/@@download/ENCFF086GJP.bed.gz
https://www.encodeproject.org/files/ENCFF668CLC/@@download/ENCFF668CLC.bed.gz
https://www.encodeproject.org/files/ENCFF617JQS/@@download/ENCFF617JQS.bed.gz
https://www.encodeproject.org/files/ENCFF603GMZ/@@download/ENCFF603GMZ.bed.gz
https://www.encodeproject.org/files/ENCFF876JTC/@@download/ENCFF876JTC.bed.gz
https://www.encodeproject.org/files/ENCFF484TJY/@@download/ENCFF484TJY.bed.gz
https://www.encodeproject.org/files/ENCFF560WHY/@@download/ENCFF560WHY.bed.gz
https://www.encodeproject.org/files/ENCFF862SRI/@@download/ENCFF862SRI.bed.gz
https://www.encodeproject.org/files/ENCFF653SEN/@@download/ENCFF653SEN.bed.gz
https://www.encodeproject.org/files/ENCFF638SAX/@@download/ENCFF638SAX.bed.gz
https://www.encodeproject.org/files/ENCFF621NXV/@@download/ENCFF621NXV.bed.gz
https://www.encodeproject.org/files/ENCFF769PHB/@@download/ENCFF769PHB.bed.gz
https://www.encodeproject.org/files/ENCFF287ZYU/@@download/ENCFF287ZYU.bed.gz
https://www.encodeproject.org/files/ENCFF711DHZ/@@download/ENCFF711DHZ.bed.gz
https://www.encodeproject.org/files/ENCFF851EKF/@@download/ENCFF851EKF.bed.gz
https://www.encodeproject.org/files/ENCFF032ACV/@@download/ENCFF032ACV.bed.gz
https://www.encodeproject.org/files/ENCFF420PED/@@download/ENCFF420PED.bed.gz
https://www.encodeproject.org/files/ENCFF755MSZ/@@download/ENCFF755MSZ.bed.gz
https://www.encodeproject.org/files/ENCFF063GQS/@@download/ENCFF063GQS.bed.gz
https://www.encodeproject.org/files/ENCFF235XPK/@@download/ENCFF235XPK.bed.gz
https://www.encodeproject.org/files/ENCFF567PTY/@@download/ENCFF567PTY.bed.gz
https://www.encodeproject.org/files/ENCFF693FRV/@@download/ENCFF693FRV.bed.gz
https://www.encodeproject.org/files/ENCFF915RRJ/@@download/ENCFF915RRJ.bed.gz
https://www.encodeproject.org/files/ENCFF841CBQ/@@download/ENCFF841CBQ.bed.gz
https://www.encodeproject.org/files/ENCFF028HOI/@@download/ENCFF028HOI.bed.gz
https://www.encodeproject.org/files/ENCFF940TMU/@@download/ENCFF940TMU.bed.gz
https://www.encodeproject.org/files/ENCFF293LRQ/@@download/ENCFF293LRQ.bed.gz
https://www.encodeproject.org/files/ENCFF216JDJ/@@download/ENCFF216JDJ.bed.gz
https://www.encodeproject.org/files/ENCFF539QIT/@@download/ENCFF539QIT.bed.gz
https://www.encodeproject.org/files/ENCFF344EDE/@@download/ENCFF344EDE.bed.gz
https://www.encodeproject.org/files/ENCFF295GBJ/@@download/ENCFF295GBJ.bed.gz

Supplementary Table 1

https://www.encodeproject.org/files/ENCFF555YAB/@@download/ENCFF555YAB.bed.gz

https://www.encodeproject.org/files/ENCFF001XTH/@@download/ENCFF001XTH.bed.gz

https://www.encodeproject.org/files/ENCFF001XTI/@@download/ENCFF001XTI.bed.gz

https://www.encodeproject.org/files/ENCFF002DDN/@@download/ENCFF002DDN.bed.gz

https://www.encodeproject.org/files/ENCFF722THT/@@download/ENCFF722THT.bed.gz

https://www.encodeproject.org/files/ENCFF041YKU/@@download/ENCFF041YKU.bed.gz

https://www.encodeproject.org/files/ENCFF739DVS/@@download/ENCFF739DVS.bed.gz

https://www.encodeproject.org/files/ENCFF113XGW/@@download/ENCFF113XGW.bed.gz

https://www.encodeproject.org/files/ENCFF189KUR/@@download/ENCFF189KUR.bed.gz

https://www.encodeproject.org/files/ENCFF770XDD/@@download/ENCFF770XDD.bed.gz

https://www.encodeproject.org/files/ENCFF788BDL/@@download/ENCFF788BDL.bed.gz

https://www.encodeproject.org/files/ENCFF249KQN/@@download/ENCFF249KQN.bed.gz

https://www.encodeproject.org/files/ENCFF455JVE/@@download/ENCFF455JVE.bed.gz

https://www.encodeproject.org/files/ENCFF334TIY/@@download/ENCFF334TIY.bed.gz

https://www.encodeproject.org/files/ENCFF919URJ/@@download/ENCFF919URJ.bed.gz

https://www.encodeproject.org/files/ENCFF146URA/@@download/ENCFF146URA.bed.gz

https://www.encodeproject.org/files/ENCFF337QJW/@@download/ENCFF337QJW.bed.gz

https://www.encodeproject.org/files/ENCFF975PTM/@@download/ENCFF975PTM.bed.gz

https://www.encodeproject.org/files/ENCFF797UDY/@@download/ENCFF797UDY.bed.gz

https://www.encodeproject.org/files/ENCFF041MRZ/@@download/ENCFF041MRZ.bed.gz

https://www.encodeproject.org/files/ENCFF895LVK/@@download/ENCFF895LVK.bed.gz

https://www.encodeproject.org/files/ENCFF480OTT/@@download/ENCFF480OTT.bed.gz

https://www.encodeproject.org/files/ENCFF833NHM/@@download/ENCFF833NHM.bed.gz

https://www.encodeproject.org/files/ENCFF322MFZ/@@download/ENCFF322MFZ.bed.gz

https://www.encodeproject.org/files/ENCFF145NMU/@@download/ENCFF145NMU.bed.gz

https://www.encodeproject.org/files/ENCFF839MBM/@@download/ENCFF839MBM.bed.gz

https://www.encodeproject.org/files/ENCFF435XEZ/@@download/ENCFF435XEZ.bed.gz

https://www.encodeproject.org/files/ENCFF683XUE/@@download/ENCFF683XUE.bed.gz

https://www.encodeproject.org/files/ENCFF917NOB/@@download/ENCFF917NOB.bed.gz

https://www.encodeproject.org/files/ENCFF211FVC/@@download/ENCFF211FVC.bed.gz

https://www.encodeproject.org/files/ENCFF851HUJ/@@download/ENCFF851HUJ.bed.gz

https://www.encodeproject.org/files/ENCFF396QYK/@@download/ENCFF396QYK.bed.gz

https://www.encodeproject.org/files/ENCFF579AYO/@@download/ENCFF579AYO.bed.gz

https://www.encodeproject.org/files/ENCFF642PNJ/@@download/ENCFF642PNJ.bed.gz

https://www.encodeproject.org/files/ENCFF152LUE/@@download/ENCFF152LUE.bed.gz

https://www.encodeproject.org/files/ENCFF478PUG/@@download/ENCFF478PUG.bed.gz

https://www.encodeproject.org/files/ENCFF720EBM/@@download/ENCFF720EBM.bed.gz

https://www.encodeproject.org/files/ENCFF838VFX/@@download/ENCFF838VFX.bed.gz

https://www.encodeproject.org/files/ENCFF805MUO/@@download/ENCFF805MUO.bed.gz

https://www.encodeproject.org/files/ENCFF214QUX/@@download/ENCFF214QUX.bed.gz

https://www.encodeproject.org/files/ENCFF089DNN/@@download/ENCFF089DNN.bed.gz

https://www.encodeproject.org/files/ENCFF278BOV/@@download/ENCFF278BOV.bed.gz

https://www.encodeproject.org/files/ENCFF165GTT/@@download/ENCFF165GTT.bed.gz

https://www.encodeproject.org/files/ENCFF669BQN/@@download/ENCFF669BQN.bed.gz

https://www.encodeproject.org/files/ENCFF421FKH/@@download/ENCFF421FKH.bed.gz

https://www.encodeproject.org/files/ENCFF457PHY/@@download/ENCFF457PHY.bed.gz

https://www.encodeproject.org/files/ENCFF409TGM/@@download/ENCFF409TGM.bed.gz

https://www.encodeproject.org/files/ENCFF371DUN/@@download/ENCFF371DUN.bed.gz

https://www.encodeproject.org/files/ENCFF612TRG/@@download/ENCFF612TRG.bed.gz

https://www.encodeproject.org/files/ENCFF137BQM/@@download/ENCFF137BQM.bed.gz

https://www.encodeproject.org/files/ENCFF046CGT/@@download/ENCFF046CGT.bed.gz

https://www.encodeproject.org/files/ENCFF353CLB/@@download/ENCFF353CLB.bed.gz

https://www.encodeproject.org/files/ENCFF565FJL/@@download/ENCFF565FJL.bed.gz

Supplementary Table 1

https://www.encodeproject.org/files/ENCFF761ORT/@@download/ENCFF761ORT.bed.gz
https://www.encodeproject.org/files/ENCFF934XEQ/@@download/ENCFF934XEQ.bed.gz
https://www.encodeproject.org/files/ENCFF797KQK/@@download/ENCFF797KQK.bed.gz
https://www.encodeproject.org/files/ENCFF169FFA/@@download/ENCFF169FFA.bed.gz
https://www.encodeproject.org/files/ENCFF817OVB/@@download/ENCFF817OVB.bed.gz
https://www.encodeproject.org/files/ENCFF531NFE/@@download/ENCFF531NFE.bed.gz
https://www.encodeproject.org/files/ENCFF771DOD/@@download/ENCFF771DOD.bed.gz
https://www.encodeproject.org/files/ENCFF930MUR/@@download/ENCFF930MUR.bed.gz
https://www.encodeproject.org/files/ENCFF052LSZ/@@download/ENCFF052LSZ.bed.gz
https://www.encodeproject.org/files/ENCFF865ACL/@@download/ENCFF865ACL.bed.gz
https://www.encodeproject.org/files/ENCFF532PJX/@@download/ENCFF532PJX.bed.gz
https://www.encodeproject.org/files/ENCFF977ZBK/@@download/ENCFF977ZBK.bed.gz
https://www.encodeproject.org/files/ENCFF015AQY/@@download/ENCFF015AQY.bed.gz
https://www.encodeproject.org/files/ENCFF835VAP/@@download/ENCFF835VAP.bed.gz
https://www.encodeproject.org/files/ENCFF913NGH/@@download/ENCFF913NGH.bed.gz
https://www.encodeproject.org/files/ENCFF287SLI/@@download/ENCFF287SLI.bed.gz
https://www.encodeproject.org/files/ENCFF614IMK/@@download/ENCFF614IMK.bed.gz
https://www.encodeproject.org/files/ENCFF739FWZ/@@download/ENCFF739FWZ.bed.gz
https://www.encodeproject.org/files/ENCFF903KIH/@@download/ENCFF903KIH.bed.gz
https://www.encodeproject.org/files/ENCFF225XDH/@@download/ENCFF225XDH.bed.gz
https://www.encodeproject.org/files/ENCFF407JBN/@@download/ENCFF407JBN.bed.gz
https://www.encodeproject.org/files/ENCFF994NGF/@@download/ENCFF994NGF.bed.gz
https://www.encodeproject.org/files/ENCFF977MOV/@@download/ENCFF977MOV.bed.gz
https://www.encodeproject.org/files/ENCFF777TAV/@@download/ENCFF777TAV.bed.gz
https://www.encodeproject.org/files/ENCFF782XGQ/@@download/ENCFF782XGQ.bed.gz
https://www.encodeproject.org/files/ENCFF214OJW/@@download/ENCFF214OJW.bed.gz
https://www.encodeproject.org/files/ENCFF151OYY/@@download/ENCFF151OYY.bed.gz
https://www.encodeproject.org/files/ENCFF739PUT/@@download/ENCFF739PUT.bed.gz
https://www.encodeproject.org/files/ENCFF004BWM/@@download/ENCFF004BWM.bed.gz
https://www.encodeproject.org/files/ENCFF071EJZ/@@download/ENCFF071EJZ.bed.gz
https://www.encodeproject.org/files/ENCFF251FHE/@@download/ENCFF251FHE.bed.gz
https://www.encodeproject.org/files/ENCFF978TMH/@@download/ENCFF978TMH.bed.gz
https://www.encodeproject.org/files/ENCFF881EJW/@@download/ENCFF881EJW.bed.gz
https://www.encodeproject.org/files/ENCFF502LWJ/@@download/ENCFF502LWJ.bed.gz
https://www.encodeproject.org/files/ENCFF512BFW/@@download/ENCFF512BFW.bed.gz
https://www.encodeproject.org/files/ENCFF204PED/@@download/ENCFF204PED.bed.gz
https://www.encodeproject.org/files/ENCFF001VQS/@@download/ENCFF001VQS.bed.gz
https://www.encodeproject.org/files/ENCFF002CZW/@@download/ENCFF002CZW.bed.gz
https://www.encodeproject.org/files/ENCFF582WLM/@@download/ENCFF582WLM.bed.gz
https://www.encodeproject.org/files/ENCFF373SRH/@@download/ENCFF373SRH.bed.gz
https://www.encodeproject.org/files/ENCFF080EAQ/@@download/ENCFF080EAQ.bed.gz
https://www.encodeproject.org/files/ENCFF430QRQ/@@download/ENCFF430QRQ.bed.gz
https://www.encodeproject.org/files/ENCFF326ZRY/@@download/ENCFF326ZRY.bed.gz
https://www.encodeproject.org/files/ENCFF915VTY/@@download/ENCFF915VTY.bed.gz
https://www.encodeproject.org/files/ENCFF517PUF/@@download/ENCFF517PUF.bed.gz
https://www.encodeproject.org/files/ENCFF727ZIT/@@download/ENCFF727ZIT.bed.gz
https://www.encodeproject.org/files/ENCFF896PDY/@@download/ENCFF896PDY.bed.gz
https://www.encodeproject.org/files/ENCFF688LUP/@@download/ENCFF688LUP.bed.gz
https://www.encodeproject.org/files/ENCFF001XQA/@@download/ENCFF001XQA.bed.gz
https://www.encodeproject.org/files/ENCFF002DCQ/@@download/ENCFF002DCQ.bed.gz
https://www.encodeproject.org/files/ENCFF344UBE/@@download/ENCFF344UBE.bed.gz
https://www.encodeproject.org/files/ENCFF079UMY/@@download/ENCFF079UMY.bed.gz
https://www.encodeproject.org/files/ENCFF243AGG/@@download/ENCFF243AGG.bed.gz

https://www.encodeproject.org/files/ENCFF861NLK/@@download/ENCFF861NLK.bed.gz
https://www.encodeproject.org/files/ENCFF783YGC/@@download/ENCFF783YGC.bed.gz
https://www.encodeproject.org/files/ENCFF462XAK/@@download/ENCFF462XAK.bed.gz
https://www.encodeproject.org/files/ENCFF285JQX/@@download/ENCFF285JQX.bed.gz
https://www.encodeproject.org/files/ENCFF997IFW/@@download/ENCFF997IFW.bed.gz
https://www.encodeproject.org/files/ENCFF280YAF/@@download/ENCFF280YAF.bed.gz
https://www.encodeproject.org/files/ENCFF045GWC/@@download/ENCFF045GWC.bed.gz
https://www.encodeproject.org/files/ENCFF634EKL/@@download/ENCFF634EKL.bed.gz
https://www.encodeproject.org/files/ENCFF957WCX/@@download/ENCFF957WCX.bed.gz
https://www.encodeproject.org/files/ENCFF556UZM/@@download/ENCFF556UZM.bed.gz
https://www.encodeproject.org/files/ENCFF258PIW/@@download/ENCFF258PIW.bed.gz
https://www.encodeproject.org/files/ENCFF698APF/@@download/ENCFF698APF.bed.gz
https://www.encodeproject.org/files/ENCFF594PYS/@@download/ENCFF594PYS.bed.gz
https://www.encodeproject.org/files/ENCFF748WSU/@@download/ENCFF748WSU.bed.gz
https://www.encodeproject.org/files/ENCFF247UJX/@@download/ENCFF247UJX.bed.gz
https://www.encodeproject.org/files/ENCFF178WRO/@@download/ENCFF178WRO.bed.gz
https://www.encodeproject.org/files/ENCFF783JKF/@@download/ENCFF783JKF.bed.gz
https://www.encodeproject.org/files/ENCFF034ALK/@@download/ENCFF034ALK.bed.gz
https://www.encodeproject.org/files/ENCFF085VWO/@@download/ENCFF085VWO.bed.gz
https://www.encodeproject.org/files/ENCFF510UBQ/@@download/ENCFF510UBQ.bed.gz
https://www.encodeproject.org/files/ENCFF501HAG/@@download/ENCFF501HAG.bed.gz
https://www.encodeproject.org/files/ENCFF330BPK/@@download/ENCFF330BPK.bed.gz
https://www.encodeproject.org/files/ENCFF159MIH/@@download/ENCFF159MIH.bed.gz
https://www.encodeproject.org/files/ENCFF285PQA/@@download/ENCFF285PQA.bed.gz
https://www.encodeproject.org/files/ENCFF538LRG/@@download/ENCFF538LRG.bed.gz
https://www.encodeproject.org/files/ENCFF545BKB/@@download/ENCFF545BKB.bed.gz
https://www.encodeproject.org/files/ENCFF168JLI/@@download/ENCFF168JLI.bed.gz
https://www.encodeproject.org/files/ENCFF168JTA/@@download/ENCFF168JTA.bed.gz
https://www.encodeproject.org/files/ENCFF332CDD/@@download/ENCFF332CDD.bed.gz
https://www.encodeproject.org/files/ENCFF282KOR/@@download/ENCFF282KOR.bed.gz
https://www.encodeproject.org/files/ENCFF474ZPF/@@download/ENCFF474ZPF.bed.gz
https://www.encodeproject.org/files/ENCFF320OWB/@@download/ENCFF320OWB.bed.gz
https://www.encodeproject.org/files/ENCFF125PLO/@@download/ENCFF125PLO.bed.gz
https://www.encodeproject.org/files/ENCFF047WBC/@@download/ENCFF047WBC.bed.gz
https://www.encodeproject.org/files/ENCFF786DKS/@@download/ENCFF786DKS.bed.gz
https://www.encodeproject.org/files/ENCFF027LSP/@@download/ENCFF027LSP.bed.gz
https://www.encodeproject.org/files/ENCFF892FWF/@@download/ENCFF892FWF.bed.gz
https://www.encodeproject.org/files/ENCFF307CSN/@@download/ENCFF307CSN.bed.gz
https://www.encodeproject.org/files/ENCFF905JAC/@@download/ENCFF905JAC.bed.gz
https://www.encodeproject.org/files/ENCFF022XIX/@@download/ENCFF022XIX.bed.gz
https://www.encodeproject.org/files/ENCFF957ICB/@@download/ENCFF957ICB.bed.gz
https://www.encodeproject.org/files/ENCFF713MCF/@@download/ENCFF713MCF.bed.gz
https://www.encodeproject.org/files/ENCFF240XXW/@@download/ENCFF240XXW.bed.gz
https://www.encodeproject.org/files/ENCFF839MCU/@@download/ENCFF839MCU.bed.gz
https://www.encodeproject.org/files/ENCFF001VLK/@@download/ENCFF001VLK.bed.gz
https://www.encodeproject.org/files/ENCFF002CVE/@@download/ENCFF002CVE.bed.gz
https://www.encodeproject.org/files/ENCFF072JVL/@@download/ENCFF072JVL.bed.gz
https://www.encodeproject.org/files/ENCFF250QNK/@@download/ENCFF250QNK.bed.gz
https://www.encodeproject.org/files/ENCFF335UAO/@@download/ENCFF335UAO.bed.gz
https://www.encodeproject.org/files/ENCFF498MXX/@@download/ENCFF498MXX.bed.gz
https://www.encodeproject.org/files/ENCFF704TBE/@@download/ENCFF704TBE.bed.gz
https://www.encodeproject.org/files/ENCFF782GCQ/@@download/ENCFF782GCQ.bed.gz
https://www.encodeproject.org/files/ENCFF338TGS/@@download/ENCFF338TGS.bed.gz

https://www.encodeproject.org/files/ENCFF804QQN/@@download/ENCFF804QQN.bed.gz
https://www.encodeproject.org/files/ENCFF174FQT/@@download/ENCFF174FQT.bed.gz
https://www.encodeproject.org/files/ENCFF236CLN/@@download/ENCFF236CLN.bed.gz
https://www.encodeproject.org/files/ENCFF349WRG/@@download/ENCFF349WRG.bed.gz
https://www.encodeproject.org/files/ENCFF128UUT/@@download/ENCFF128UUT.bed.gz
https://www.encodeproject.org/files/ENCFF001UTX/@@download/ENCFF001UTX.bed.gz
https://www.encodeproject.org/files/ENCFF002DBR/@@download/ENCFF002DBR.bed.gz
https://www.encodeproject.org/files/ENCFF226MQR/@@download/ENCFF226MQR.bed.gz
https://www.encodeproject.org/files/ENCFF705JDO/@@download/ENCFF705JDO.bed.gz
https://www.encodeproject.org/files/ENCFF680MAX/@@download/ENCFF680MAX.bed.gz
https://www.encodeproject.org/files/ENCFF974JVU/@@download/ENCFF974JVU.bed.gz
https://www.encodeproject.org/files/ENCFF623KYU/@@download/ENCFF623KYU.bed.gz
https://www.encodeproject.org/files/ENCFF465MVV/@@download/ENCFF465MVV.bed.gz
https://www.encodeproject.org/files/ENCFF028IIR/@@download/ENCFF028IIR.bed.gz
https://www.encodeproject.org/files/ENCFF301UPP/@@download/ENCFF301UPP.bed.gz
https://www.encodeproject.org/files/ENCFF246QUK/@@download/ENCFF246QUK.bed.gz
https://www.encodeproject.org/files/ENCFF258KBF/@@download/ENCFF258KBF.bed.gz
https://www.encodeproject.org/files/ENCFF591PIT/@@download/ENCFF591PIT.bed.gz
https://www.encodeproject.org/files/ENCFF960TOX/@@download/ENCFF960TOX.bed.gz
https://www.encodeproject.org/files/ENCFF001XSD/@@download/ENCFF001XSD.bed.gz
https://www.encodeproject.org/files/ENCFF001XSE/@@download/ENCFF001XSE.bed.gz
https://www.encodeproject.org/files/ENCFF002DDF/@@download/ENCFF002DDF.bed.gz
https://www.encodeproject.org/files/ENCFF423CGM/@@download/ENCFF423CGM.bed.gz
https://www.encodeproject.org/files/ENCFF516DWM/@@download/ENCFF516DWM.bed.gz
https://www.encodeproject.org/files/ENCFF832CJL/@@download/ENCFF832CJL.bed.gz
https://www.encodeproject.org/files/ENCFF010WHH/@@download/ENCFF010WHH.bed.gz
https://www.encodeproject.org/files/ENCFF674KUN/@@download/ENCFF674KUN.bed.gz
https://www.encodeproject.org/files/ENCFF583GIR/@@download/ENCFF583GIR.bed.gz
https://www.encodeproject.org/files/ENCFF536XBD/@@download/ENCFF536XBD.bed.gz
https://www.encodeproject.org/files/ENCFF168VBK/@@download/ENCFF168VBK.bed.gz
https://www.encodeproject.org/files/ENCFF444ZJW/@@download/ENCFF444ZJW.bed.gz
https://www.encodeproject.org/files/ENCFF727BRP/@@download/ENCFF727BRP.bed.gz
https://www.encodeproject.org/files/ENCFF856ZCX/@@download/ENCFF856ZCX.bed.gz
https://www.encodeproject.org/files/ENCFF045AYR/@@download/ENCFF045AYR.bed.gz
https://www.encodeproject.org/files/ENCFF943XYS/@@download/ENCFF943XYS.bed.gz
https://www.encodeproject.org/files/ENCFF973KKY/@@download/ENCFF973KKY.bed.gz
https://www.encodeproject.org/files/ENCFF001XRZ/@@download/ENCFF001XRZ.bed.gz
https://www.encodeproject.org/files/ENCFF001XSA/@@download/ENCFF001XSA.bed.gz
https://www.encodeproject.org/files/ENCFF002DDE/@@download/ENCFF002DDE.bed.gz
https://www.encodeproject.org/files/ENCFF484FSJ/@@download/ENCFF484FSJ.bed.gz
https://www.encodeproject.org/files/ENCFF523ROB/@@download/ENCFF523ROB.bed.gz
https://www.encodeproject.org/files/ENCFF909NRN/@@download/ENCFF909NRN.bed.gz
https://www.encodeproject.org/files/ENCFF777ODE/@@download/ENCFF777ODE.bed.gz
https://www.encodeproject.org/files/ENCFF213JJM/@@download/ENCFF213JJM.bed.gz
https://www.encodeproject.org/files/ENCFF129FBO/@@download/ENCFF129FBO.bed.gz
https://www.encodeproject.org/files/ENCFF519REF/@@download/ENCFF519REF.bed.gz
https://www.encodeproject.org/files/ENCFF570FLB/@@download/ENCFF570FLB.bed.gz
https://www.encodeproject.org/files/ENCFF001XTN/@@download/ENCFF001XTN.bed.gz
https://www.encodeproject.org/files/ENCFF001XTO/@@download/ENCFF001XTO.bed.gz
https://www.encodeproject.org/files/ENCFF002DDP/@@download/ENCFF002DDP.bed.gz
https://www.encodeproject.org/files/ENCFF297PJW/@@download/ENCFF297PJW.bed.gz
https://www.encodeproject.org/files/ENCFF932EHP/@@download/ENCFF932EHP.bed.gz
https://www.encodeproject.org/files/ENCFF390JNR/@@download/ENCFF390JNR.bed.gz

https://www.encodeproject.org/files/ENCFF192UQS/@@download/ENCFF192UQS.bed.gz
https://www.encodeproject.org/files/ENCFF447AUB/@@download/ENCFF447AUB.bed.gz
https://www.encodeproject.org/files/ENCFF077RDH/@@download/ENCFF077RDH.bed.gz
https://www.encodeproject.org/files/ENCFF773EJN/@@download/ENCFF773EJN.bed.gz
https://www.encodeproject.org/files/ENCFF470PNM/@@download/ENCFF470PNM.bed.gz
https://www.encodeproject.org/files/ENCFF943ZQC/@@download/ENCFF943ZQC.bed.gz
https://www.encodeproject.org/files/ENCFF245IPV/@@download/ENCFF245IPV.bed.gz
https://www.encodeproject.org/files/ENCFF603KUP/@@download/ENCFF603KUP.bed.gz
https://www.encodeproject.org/files/ENCFF490PIH/@@download/ENCFF490PIH.bed.gz
https://www.encodeproject.org/files/ENCFF951SRP/@@download/ENCFF951SRP.bed.gz
https://www.encodeproject.org/files/ENCFF483EHQ/@@download/ENCFF483EHQ.bed.gz
https://www.encodeproject.org/files/ENCFF454PSG/@@download/ENCFF454PSG.bed.gz
https://www.encodeproject.org/files/ENCFF737SPJ/@@download/ENCFF737SPJ.bed.gz
https://www.encodeproject.org/files/ENCFF157OEN/@@download/ENCFF157OEN.bed.gz
https://www.encodeproject.org/files/ENCFF002CED/@@download/ENCFF002CED.bed.gz
https://www.encodeproject.org/files/ENCFF001VLJ/@@download/ENCFF001VLJ.bed.gz
https://www.encodeproject.org/files/ENCFF002CVD/@@download/ENCFF002CVD.bed.gz
https://www.encodeproject.org/files/ENCFF822SAU/@@download/ENCFF822SAU.bed.gz
https://www.encodeproject.org/files/ENCFF991EAY/@@download/ENCFF991EAY.bed.gz
https://www.encodeproject.org/files/ENCFF311XFB/@@download/ENCFF311XFB.bed.gz
https://www.encodeproject.org/files/ENCFF107EHZ/@@download/ENCFF107EHZ.bed.gz
https://www.encodeproject.org/files/ENCFF222CSK/@@download/ENCFF222CSK.bed.gz
https://www.encodeproject.org/files/ENCFF813LKS/@@download/ENCFF813LKS.bed.gz
https://www.encodeproject.org/files/ENCFF756UDU/@@download/ENCFF756UDU.bed.gz
https://www.encodeproject.org/files/ENCFF987YIJ/@@download/ENCFF987YIJ.bed.gz
https://www.encodeproject.org/files/ENCFF010FVB/@@download/ENCFF010FVB.bed.gz
https://www.encodeproject.org/files/ENCFF694HYO/@@download/ENCFF694HYO.bed.gz
https://www.encodeproject.org/files/ENCFF957WEQ/@@download/ENCFF957WEQ.bed.gz
https://www.encodeproject.org/files/ENCFF367GGX/@@download/ENCFF367GGX.bed.gz
https://www.encodeproject.org/files/ENCFF827SHP/@@download/ENCFF827SHP.bed.gz
https://www.encodeproject.org/files/ENCFF265BAG/@@download/ENCFF265BAG.bed.gz
https://www.encodeproject.org/files/ENCFF358WEO/@@download/ENCFF358WEO.bed.gz
https://www.encodeproject.org/files/ENCFF459AHK/@@download/ENCFF459AHK.bed.gz
https://www.encodeproject.org/files/ENCFF340BQM/@@download/ENCFF340BQM.bed.gz
https://www.encodeproject.org/files/ENCFF596RDA/@@download/ENCFF596RDA.bed.gz
https://www.encodeproject.org/files/ENCFF002CEW/@@download/ENCFF002CEW.bed.gz
https://www.encodeproject.org/files/ENCFF113DAW/@@download/ENCFF113DAW.bed.gz
https://www.encodeproject.org/files/ENCFF527FXX/@@download/ENCFF527FXX.bed.gz
https://www.encodeproject.org/files/ENCFF857XBX/@@download/ENCFF857XBX.bed.gz
https://www.encodeproject.org/files/ENCFF415WKV/@@download/ENCFF415WKV.bed.gz
https://www.encodeproject.org/files/ENCFF678OVT/@@download/ENCFF678OVT.bed.gz
https://www.encodeproject.org/files/ENCFF567MRO/@@download/ENCFF567MRO.bed.gz
https://www.encodeproject.org/files/ENCFF660ZJK/@@download/ENCFF660ZJK.bed.gz
https://www.encodeproject.org/files/ENCFF046QCH/@@download/ENCFF046QCH.bed.gz
https://www.encodeproject.org/files/ENCFF148BSH/@@download/ENCFF148BSH.bed.gz
https://www.encodeproject.org/files/ENCFF287UAV/@@download/ENCFF287UAV.bed.gz
https://www.encodeproject.org/files/ENCFF601XVS/@@download/ENCFF601XVS.bed.gz
https://www.encodeproject.org/files/ENCFF893NMN/@@download/ENCFF893NMN.bed.gz
https://www.encodeproject.org/files/ENCFF081PSJ/@@download/ENCFF081PSJ.bed.gz
https://www.encodeproject.org/files/ENCFF711TIK/@@download/ENCFF711TIK.bed.gz
https://www.encodeproject.org/files/ENCFF450VON/@@download/ENCFF450VON.bed.gz
https://www.encodeproject.org/files/ENCFF979XUR/@@download/ENCFF979XUR.bed.gz
https://www.encodeproject.org/files/ENCFF148UOD/@@download/ENCFF148UOD.bed.gz

https://www.encodeproject.org/files/ENCFF002CEC/@@download/ENCFF002CEC.bed.gz
https://www.encodeproject.org/files/ENCFF357WXQ/@@download/ENCFF357WXQ.bed.gz
https://www.encodeproject.org/files/ENCFF177LBB/@@download/ENCFF177LBB.bed.gz
https://www.encodeproject.org/files/ENCFF015WVN/@@download/ENCFF015WVN.bed.gz
https://www.encodeproject.org/files/ENCFF743FCW/@@download/ENCFF743FCW.bed.gz
https://www.encodeproject.org/files/ENCFF385LJM/@@download/ENCFF385LJM.bed.gz
https://www.encodeproject.org/files/ENCFF324RIT/@@download/ENCFF324RIT.bed.gz
https://www.encodeproject.org/files/ENCFF633TFF/@@download/ENCFF633TFF.bed.gz
https://www.encodeproject.org/files/ENCFF710QDO/@@download/ENCFF710QDO.bed.gz
https://www.encodeproject.org/files/ENCFF856VSR/@@download/ENCFF856VSR.bed.gz
https://www.encodeproject.org/files/ENCFF420KMT/@@download/ENCFF420KMT.bed.gz
https://www.encodeproject.org/files/ENCFF658WYM/@@download/ENCFF658WYM.bed.gz
https://www.encodeproject.org/files/ENCFF355LBT/@@download/ENCFF355LBT.bed.gz
https://www.encodeproject.org/files/ENCFF177UJN/@@download/ENCFF177UJN.bed.gz
https://www.encodeproject.org/files/ENCFF980GQH/@@download/ENCFF980GQH.bed.gz
https://www.encodeproject.org/files/ENCFF001VLH/@@download/ENCFF001VLH.bed.gz
https://www.encodeproject.org/files/ENCFF002CVB/@@download/ENCFF002CVB.bed.gz
https://www.encodeproject.org/files/ENCFF390MXI/@@download/ENCFF390MXI.bed.gz
https://www.encodeproject.org/files/ENCFF221BNG/@@download/ENCFF221BNG.bed.gz
https://www.encodeproject.org/files/ENCFF587UQZ/@@download/ENCFF587UQZ.bed.gz
https://www.encodeproject.org/files/ENCFF387HZM/@@download/ENCFF387HZM.bed.gz
https://www.encodeproject.org/files/ENCFF971LQR/@@download/ENCFF971LQR.bed.gz
https://www.encodeproject.org/files/ENCFF854VZK/@@download/ENCFF854VZK.bed.gz
https://www.encodeproject.org/files/ENCFF913JJY/@@download/ENCFF913JJY.bed.gz
https://www.encodeproject.org/files/ENCFF722HUF/@@download/ENCFF722HUF.bed.gz
https://www.encodeproject.org/files/ENCFF679RTG/@@download/ENCFF679RTG.bed.gz
https://www.encodeproject.org/files/ENCFF327GZX/@@download/ENCFF327GZX.bed.gz
https://www.encodeproject.org/files/ENCFF002CEZ/@@download/ENCFF002CEZ.bed.gz
https://www.encodeproject.org/files/ENCFF585OAO/@@download/ENCFF585OAO.bed.gz
https://www.encodeproject.org/files/ENCFF551TJN/@@download/ENCFF551TJN.bed.gz
https://www.encodeproject.org/files/ENCFF226DWG/@@download/ENCFF226DWG.bed.gz
https://www.encodeproject.org/files/ENCFF639HLV/@@download/ENCFF639HLV.bed.gz
https://www.encodeproject.org/files/ENCFF156CFD/@@download/ENCFF156CFD.bed.gz
https://www.encodeproject.org/files/ENCFF124DUJ/@@download/ENCFF124DUJ.bed.gz
https://www.encodeproject.org/files/ENCFF797UMO/@@download/ENCFF797UMO.bed.gz
https://www.encodeproject.org/files/ENCFF119YFP/@@download/ENCFF119YFP.bed.gz
https://www.encodeproject.org/files/ENCFF618OSB/@@download/ENCFF618OSB.bed.gz
https://www.encodeproject.org/files/ENCFF340LPI/@@download/ENCFF340LPI.bed.gz
https://www.encodeproject.org/files/ENCFF265XHP/@@download/ENCFF265XHP.bed.gz
https://www.encodeproject.org/files/ENCFF537XJK/@@download/ENCFF537XJK.bed.gz
https://www.encodeproject.org/files/ENCFF444PFG/@@download/ENCFF444PFG.bed.gz
https://www.encodeproject.org/files/ENCFF639IZT/@@download/ENCFF639IZT.bed.gz
https://www.encodeproject.org/files/ENCFF298LBC/@@download/ENCFF298LBC.bed.gz
https://www.encodeproject.org/files/ENCFF158RRC/@@download/ENCFF158RRC.bed.gz
https://www.encodeproject.org/files/ENCFF639MNI/@@download/ENCFF639MNI.bed.gz
https://www.encodeproject.org/files/ENCFF625CSU/@@download/ENCFF625CSU.bed.gz
https://www.encodeproject.org/files/ENCFF848EEE/@@download/ENCFF848EEE.bed.gz
https://www.encodeproject.org/files/ENCFF565EAB/@@download/ENCFF565EAB.bed.gz
https://www.encodeproject.org/files/ENCFF167UCC/@@download/ENCFF167UCC.bed.gz
https://www.encodeproject.org/files/ENCFF086ABE/@@download/ENCFF086ABE.bed.gz
https://www.encodeproject.org/files/ENCFF444TJW/@@download/ENCFF444TJW.bed.gz
https://www.encodeproject.org/files/ENCFF609RFQ/@@download/ENCFF609RFQ.bed.gz
https://www.encodeproject.org/files/ENCFF913EHI/@@download/ENCFF913EHI.bed.gz

https://www.encodeproject.org/files/ENCFF968WAS/@@download/ENCFF968WAS.bed.gz
https://www.encodeproject.org/files/ENCFF201KGJ/@@download/ENCFF201KGJ.bed.gz
https://www.encodeproject.org/files/ENCFF255SKA/@@download/ENCFF255SKA.bed.gz
https://www.encodeproject.org/files/ENCFF515RCQ/@@download/ENCFF515RCQ.bed.gz
https://www.encodeproject.org/files/ENCFF094URN/@@download/ENCFF094URN.bed.gz
https://www.encodeproject.org/files/ENCFF183ZZZ/@@download/ENCFF183ZZZ.bed.gz
https://www.encodeproject.org/files/ENCFF189HPG/@@download/ENCFF189HPG.bed.gz
https://www.encodeproject.org/files/ENCFF541GTF/@@download/ENCFF541GTF.bed.gz
https://www.encodeproject.org/files/ENCFF646ODW/@@download/ENCFF646ODW.bed.gz
https://www.encodeproject.org/files/ENCFF383YWV/@@download/ENCFF383YWV.bed.gz
https://www.encodeproject.org/files/ENCFF611MLV/@@download/ENCFF611MLV.bed.gz
https://www.encodeproject.org/files/ENCFF398VGB/@@download/ENCFF398VGB.bed.gz
https://www.encodeproject.org/files/ENCFF401DFB/@@download/ENCFF401DFB.bed.gz
https://www.encodeproject.org/files/ENCFF555BRJ/@@download/ENCFF555BRJ.bed.gz
https://www.encodeproject.org/files/ENCFF026ZWL/@@download/ENCFF026ZWL.bed.gz
https://www.encodeproject.org/files/ENCFF124PUR/@@download/ENCFF124PUR.bed.gz
https://www.encodeproject.org/files/ENCFF168HOV/@@download/ENCFF168HOV.bed.gz
https://www.encodeproject.org/files/ENCFF195WDA/@@download/ENCFF195WDA.bed.gz
https://www.encodeproject.org/files/ENCFF280GHS/@@download/ENCFF280GHS.bed.gz
https://www.encodeproject.org/files/ENCFF406GXU/@@download/ENCFF406GXU.bed.gz
https://www.encodeproject.org/files/ENCFF940TNN/@@download/ENCFF940TNN.bed.gz
https://www.encodeproject.org/files/ENCFF651APG/@@download/ENCFF651APG.bed.gz
https://www.encodeproject.org/files/ENCFF535DHF/@@download/ENCFF535DHF.bed.gz
https://www.encodeproject.org/files/ENCFF167CVF/@@download/ENCFF167CVF.bed.gz
https://www.encodeproject.org/files/ENCFF822OTY/@@download/ENCFF822OTY.bed.gz
https://www.encodeproject.org/files/ENCFF394ZUH/@@download/ENCFF394ZUH.bed.gz
https://www.encodeproject.org/files/ENCFF198EUQ/@@download/ENCFF198EUQ.bed.gz
https://www.encodeproject.org/files/ENCFF828UBR/@@download/ENCFF828UBR.bed.gz
https://www.encodeproject.org/files/ENCFF142PDO/@@download/ENCFF142PDO.bed.gz
https://www.encodeproject.org/files/ENCFF896ZBU/@@download/ENCFF896ZBU.bed.gz
https://www.encodeproject.org/files/ENCFF831BFL/@@download/ENCFF831BFL.bed.gz
https://www.encodeproject.org/files/ENCFF737WAY/@@download/ENCFF737WAY.bed.gz
https://www.encodeproject.org/files/ENCFF793QRF/@@download/ENCFF793QRF.bed.gz
https://www.encodeproject.org/files/ENCFF288XHG/@@download/ENCFF288XHG.bed.gz
https://www.encodeproject.org/files/ENCFF382IQD/@@download/ENCFF382IQD.bed.gz
https://www.encodeproject.org/files/ENCFF373LJO/@@download/ENCFF373LJO.bed.gz
https://www.encodeproject.org/files/ENCFF748SCA/@@download/ENCFF748SCA.bed.gz
https://www.encodeproject.org/files/ENCFF516VNN/@@download/ENCFF516VNN.bed.gz
https://www.encodeproject.org/files/ENCFF506HHV/@@download/ENCFF506HHV.bed.gz
https://www.encodeproject.org/files/ENCFF592OPP/@@download/ENCFF592OPP.bed.gz
https://www.encodeproject.org/files/ENCFF334FKO/@@download/ENCFF334FKO.bed.gz
https://www.encodeproject.org/files/ENCFF363JNV/@@download/ENCFF363JNV.bed.gz
https://www.encodeproject.org/files/ENCFF580YDN/@@download/ENCFF580YDN.bed.gz
https://www.encodeproject.org/files/ENCFF562MDO/@@download/ENCFF562MDO.bed.gz
https://www.encodeproject.org/files/ENCFF767YMW/@@download/ENCFF767YMW.bed.gz
https://www.encodeproject.org/files/ENCFF289FHU/@@download/ENCFF289FHU.bed.gz
https://www.encodeproject.org/files/ENCFF579GUD/@@download/ENCFF579GUD.bed.gz
https://www.encodeproject.org/files/ENCFF738RTR/@@download/ENCFF738RTR.bed.gz
https://www.encodeproject.org/files/ENCFF900JGZ/@@download/ENCFF900JGZ.bed.gz
https://www.encodeproject.org/files/ENCFF875HGQ/@@download/ENCFF875HGQ.bed.gz
https://www.encodeproject.org/files/ENCFF594WWB/@@download/ENCFF594WWB.bed.gz
https://www.encodeproject.org/files/ENCFF280LUO/@@download/ENCFF280LUO.bed.gz
https://www.encodeproject.org/files/ENCFF755JEJ/@@download/ENCFF755JEJ.bed.gz

Supplementary Table 1

https://www.encodeproject.org/files/ENCFF276DMI/@@download/ENCFF276DMI.bed.gz
https://www.encodeproject.org/files/ENCFF724RDJ/@@download/ENCFF724RDJ.bed.gz
https://www.encodeproject.org/files/ENCFF238FUX/@@download/ENCFF238FUX.bed.gz
https://www.encodeproject.org/files/ENCFF164YIY/@@download/ENCFF164YIY.bed.gz
https://www.encodeproject.org/files/ENCFF236XBY/@@download/ENCFF236XBY.bed.gz
https://www.encodeproject.org/files/ENCFF629KNX/@@download/ENCFF629KNX.bed.gz
https://www.encodeproject.org/files/ENCFF988VKY/@@download/ENCFF988VKY.bed.gz
https://www.encodeproject.org/files/ENCFF289MUX/@@download/ENCFF289MUX.bed.gz
https://www.encodeproject.org/files/ENCFF928JLW/@@download/ENCFF928JLW.bed.gz
https://www.encodeproject.org/files/ENCFF865QLX/@@download/ENCFF865QLX.bed.gz
https://www.encodeproject.org/files/ENCFF690LWY/@@download/ENCFF690LWY.bed.gz
https://www.encodeproject.org/files/ENCFF765IPM/@@download/ENCFF765IPM.bed.gz
https://www.encodeproject.org/files/ENCFF911WIL/@@download/ENCFF911WIL.bed.gz
https://www.encodeproject.org/files/ENCFF665TLS/@@download/ENCFF665TLS.bed.gz
https://www.encodeproject.org/files/ENCFF567XKZ/@@download/ENCFF567XKZ.bed.gz
https://www.encodeproject.org/files/ENCFF759YZA/@@download/ENCFF759YZA.bed.gz
https://www.encodeproject.org/files/ENCFF433YXI/@@download/ENCFF433YXI.bed.gz
https://www.encodeproject.org/files/ENCFF423CKN/@@download/ENCFF423CKN.bed.gz
https://www.encodeproject.org/files/ENCFF372LOW/@@download/ENCFF372LOW.bed.gz
https://www.encodeproject.org/files/ENCFF678WCZ/@@download/ENCFF678WCZ.bed.gz
https://www.encodeproject.org/files/ENCFF288EXD/@@download/ENCFF288EXD.bed.gz
https://www.encodeproject.org/files/ENCFF674MDG/@@download/ENCFF674MDG.bed.gz
https://www.encodeproject.org/files/ENCFF230IDW/@@download/ENCFF230IDW.bed.gz
https://www.encodeproject.org/files/ENCFF974SIH/@@download/ENCFF974SIH.bed.gz
https://www.encodeproject.org/files/ENCFF167SCX/@@download/ENCFF167SCX.bed.gz
https://www.encodeproject.org/files/ENCFF930EVC/@@download/ENCFF930EVC.bed.gz
https://www.encodeproject.org/files/ENCFF146REQ/@@download/ENCFF146REQ.bed.gz
https://www.encodeproject.org/files/ENCFF432XLE/@@download/ENCFF432XLE.bed.gz
https://www.encodeproject.org/files/ENCFF300WML/@@download/ENCFF300WML.bed.gz
https://www.encodeproject.org/files/ENCFF788RFY/@@download/ENCFF788RFY.bed.gz
https://www.encodeproject.org/files/ENCFF978GEZ/@@download/ENCFF978GEZ.bed.gz
https://www.encodeproject.org/files/ENCFF294TAI/@@download/ENCFF294TAI.bed.gz
https://www.encodeproject.org/files/ENCFF575RKM/@@download/ENCFF575RKM.bed.gz
https://www.encodeproject.org/files/ENCFF013FRY/@@download/ENCFF013FRY.bed.gz
https://www.encodeproject.org/files/ENCFF333BFY/@@download/ENCFF333BFY.bed.gz
https://www.encodeproject.org/files/ENCFF999DBK/@@download/ENCFF999DBK.bed.gz
https://www.encodeproject.org/files/ENCFF751MNI/@@download/ENCFF751MNI.bed.gz
https://www.encodeproject.org/files/ENCFF792QGT/@@download/ENCFF792QGT.bed.gz
https://www.encodeproject.org/files/ENCFF251GJD/@@download/ENCFF251GJD.bed.gz
https://www.encodeproject.org/files/ENCFF109VPF/@@download/ENCFF109VPF.bed.gz
https://www.encodeproject.org/files/ENCFF852XOL/@@download/ENCFF852XOL.bed.gz
https://www.encodeproject.org/files/ENCFF515ANF/@@download/ENCFF515ANF.bed.gz
https://www.encodeproject.org/files/ENCFF215JJY/@@download/ENCFF215JJY.bed.gz
https://www.encodeproject.org/files/ENCFF823BWT/@@download/ENCFF823BWT.bed.gz
https://www.encodeproject.org/files/ENCFF854WGL/@@download/ENCFF854WGL.bed.gz
https://www.encodeproject.org/files/ENCFF355SDI/@@download/ENCFF355SDI.bed.gz
https://www.encodeproject.org/files/ENCFF559EHQ/@@download/ENCFF559EHQ.bed.gz
https://www.encodeproject.org/files/ENCFF310RRY/@@download/ENCFF310RRY.bed.gz
https://www.encodeproject.org/files/ENCFF709SVG/@@download/ENCFF709SVG.bed.gz
https://www.encodeproject.org/files/ENCFF024YSG/@@download/ENCFF024YSG.bed.gz
https://www.encodeproject.org/files/ENCFF813MTO/@@download/ENCFF813MTO.bed.gz
https://www.encodeproject.org/files/ENCFF461EJN/@@download/ENCFF461EJN.bed.gz
https://www.encodeproject.org/files/ENCFF445NVA/@@download/ENCFF445NVA.bed.gz

https://www.encodeproject.org/files/ENCFF156SPI/@@download/ENCFF156SPI.bed.gz
https://www.encodeproject.org/files/ENCFF267ZRT/@@download/ENCFF267ZRT.bed.gz
https://www.encodeproject.org/files/ENCFF957ZLA/@@download/ENCFF957ZLA.bed.gz
https://www.encodeproject.org/files/ENCFF684EDS/@@download/ENCFF684EDS.bed.gz
https://www.encodeproject.org/files/ENCFF710ZQC/@@download/ENCFF710ZQC.bed.gz
https://www.encodeproject.org/files/ENCFF039PUW/@@download/ENCFF039PUW.bed.gz
https://www.encodeproject.org/files/ENCFF984EZB/@@download/ENCFF984EZB.bed.gz
https://www.encodeproject.org/files/ENCFF932CWN/@@download/ENCFF932CWN.bed.gz
https://www.encodeproject.org/files/ENCFF716XFO/@@download/ENCFF716XFO.bed.gz
https://www.encodeproject.org/files/ENCFF560VSK/@@download/ENCFF560VSK.bed.gz
https://www.encodeproject.org/files/ENCFF108QPP/@@download/ENCFF108QPP.bed.gz
https://www.encodeproject.org/files/ENCFF220VAH/@@download/ENCFF220VAH.bed.gz
https://www.encodeproject.org/files/ENCFF828ASK/@@download/ENCFF828ASK.bed.gz
https://www.encodeproject.org/files/ENCFF762LFD/@@download/ENCFF762LFD.bed.gz
https://www.encodeproject.org/files/ENCFF776AUH/@@download/ENCFF776AUH.bed.gz
https://www.encodeproject.org/files/ENCFF946CXU/@@download/ENCFF946CXU.bed.gz
https://www.encodeproject.org/files/ENCFF427HSH/@@download/ENCFF427HSH.bed.gz
https://www.encodeproject.org/files/ENCFF620DNX/@@download/ENCFF620DNX.bed.gz
https://www.encodeproject.org/files/ENCFF547PLX/@@download/ENCFF547PLX.bed.gz
https://www.encodeproject.org/files/ENCFF693TBO/@@download/ENCFF693TBO.bed.gz
https://www.encodeproject.org/files/ENCFF554DUQ/@@download/ENCFF554DUQ.bed.gz
https://www.encodeproject.org/files/ENCFF483ENA/@@download/ENCFF483ENA.bed.gz
https://www.encodeproject.org/files/ENCFF899MQP/@@download/ENCFF899MQP.bed.gz
https://www.encodeproject.org/files/ENCFF341UHT/@@download/ENCFF341UHT.bed.gz
https://www.encodeproject.org/files/ENCFF072CBX/@@download/ENCFF072CBX.bed.gz
https://www.encodeproject.org/files/ENCFF088TUO/@@download/ENCFF088TUO.bed.gz
https://www.encodeproject.org/files/ENCFF369UWV/@@download/ENCFF369UWV.bed.gz
https://www.encodeproject.org/files/ENCFF538QPY/@@download/ENCFF538QPY.bed.gz
https://www.encodeproject.org/files/ENCFF919KHH/@@download/ENCFF919KHH.bed.gz
https://www.encodeproject.org/files/ENCFF321VDV/@@download/ENCFF321VDV.bed.gz
https://www.encodeproject.org/files/ENCFF637KHE/@@download/ENCFF637KHE.bed.gz
https://www.encodeproject.org/files/ENCFF031PBW/@@download/ENCFF031PBW.bed.gz
https://www.encodeproject.org/files/ENCFF290BOD/@@download/ENCFF290BOD.bed.gz
https://www.encodeproject.org/files/ENCFF316LSD/@@download/ENCFF316LSD.bed.gz
https://www.encodeproject.org/files/ENCFF999RRH/@@download/ENCFF999RRH.bed.gz
https://www.encodeproject.org/files/ENCFF306LCT/@@download/ENCFF306LCT.bed.gz
https://www.encodeproject.org/files/ENCFF907KEJ/@@download/ENCFF907KEJ.bed.gz
https://www.encodeproject.org/files/ENCFF665VIR/@@download/ENCFF665VIR.bed.gz
https://www.encodeproject.org/files/ENCFF956QVQ/@@download/ENCFF956QVQ.bed.gz
https://www.encodeproject.org/files/ENCFF530FGP/@@download/ENCFF530FGP.bed.gz
https://www.encodeproject.org/files/ENCFF135CBQ/@@download/ENCFF135CBQ.bed.gz
https://www.encodeproject.org/files/ENCFF005DKU/@@download/ENCFF005DKU.bed.gz
https://www.encodeproject.org/files/ENCFF169NVW/@@download/ENCFF169NVW.bed.gz
https://www.encodeproject.org/files/ENCFF736LBL/@@download/ENCFF736LBL.bed.gz
https://www.encodeproject.org/files/ENCFF374BNP/@@download/ENCFF374BNP.bed.gz
https://www.encodeproject.org/files/ENCFF536TDH/@@download/ENCFF536TDH.bed.gz
https://www.encodeproject.org/files/ENCFF423JIG/@@download/ENCFF423JIG.bed.gz
https://www.encodeproject.org/files/ENCFF122IMV/@@download/ENCFF122IMV.bed.gz
https://www.encodeproject.org/files/ENCFF070WIW/@@download/ENCFF070WIW.bed.gz
https://www.encodeproject.org/files/ENCFF514ZSQ/@@download/ENCFF514ZSQ.bed.gz
https://www.encodeproject.org/files/ENCFF983ZND/@@download/ENCFF983ZND.bed.gz
https://www.encodeproject.org/files/ENCFF379CDW/@@download/ENCFF379CDW.bed.gz
https://www.encodeproject.org/files/ENCFF122PDP/@@download/ENCFF122PDP.bed.gz

Supplementary Table 1

https://www.encodeproject.org/files/ENCFF049IPS/@@download/ENCFF049IPS.bed.gz
https://www.encodeproject.org/files/ENCFF577WJR/@@download/ENCFF577WJR.bed.gz
https://www.encodeproject.org/files/ENCFF941WRM/@@download/ENCFF941WRM.bed.gz
https://www.encodeproject.org/files/ENCFF718QZR/@@download/ENCFF718QZR.bed.gz
https://www.encodeproject.org/files/ENCFF365NAB/@@download/ENCFF365NAB.bed.gz
https://www.encodeproject.org/files/ENCFF606JRJ/@@download/ENCFF606JRJ.bed.gz
https://www.encodeproject.org/files/ENCFF868CZR/@@download/ENCFF868CZR.bed.gz
https://www.encodeproject.org/files/ENCFF178SIY/@@download/ENCFF178SIY.bed.gz
https://www.encodeproject.org/files/ENCFF224JGZ/@@download/ENCFF224JGZ.bed.gz
https://www.encodeproject.org/files/ENCFF732VLO/@@download/ENCFF732VLO.bed.gz
https://www.encodeproject.org/files/ENCFF557QPA/@@download/ENCFF557QPA.bed.gz
https://www.encodeproject.org/files/ENCFF124DEE/@@download/ENCFF124DEE.bed.gz
https://www.encodeproject.org/files/ENCFF250KJL/@@download/ENCFF250KJL.bed.gz
https://www.encodeproject.org/files/ENCFF945RSD/@@download/ENCFF945RSD.bed.gz
https://www.encodeproject.org/files/ENCFF961BPY/@@download/ENCFF961BPY.bed.gz
https://www.encodeproject.org/files/ENCFF406OWW/@@download/ENCFF406OWW.bed.gz
https://www.encodeproject.org/files/ENCFF555PVZ/@@download/ENCFF555PVZ.bed.gz
https://www.encodeproject.org/files/ENCFF444VGY/@@download/ENCFF444VGY.bed.gz
https://www.encodeproject.org/files/ENCFF134BTP/@@download/ENCFF134BTP.bed.gz
https://www.encodeproject.org/files/ENCFF870LOR/@@download/ENCFF870LOR.bed.gz
https://www.encodeproject.org/files/ENCFF977BTX/@@download/ENCFF977BTX.bed.gz
https://www.encodeproject.org/files/ENCFF382QJG/@@download/ENCFF382QJG.bed.gz
https://www.encodeproject.org/files/ENCFF162PRX/@@download/ENCFF162PRX.bed.gz
https://www.encodeproject.org/files/ENCFF211VGU/@@download/ENCFF211VGU.bed.gz
https://www.encodeproject.org/files/ENCFF104VHX/@@download/ENCFF104VHX.bed.gz
https://www.encodeproject.org/files/ENCFF781EZZ/@@download/ENCFF781EZZ.bed.gz
https://www.encodeproject.org/files/ENCFF343RNG/@@download/ENCFF343RNG.bed.gz
https://www.encodeproject.org/files/ENCFF254NYT/@@download/ENCFF254NYT.bed.gz
https://www.encodeproject.org/files/ENCFF460IIT/@@download/ENCFF460IIT.bed.gz
https://www.encodeproject.org/files/ENCFF998QVK/@@download/ENCFF998QVK.bed.gz
https://www.encodeproject.org/files/ENCFF275SGU/@@download/ENCFF275SGU.bed.gz
https://www.encodeproject.org/files/ENCFF840MMD/@@download/ENCFF840MMD.bed.gz
https://www.encodeproject.org/files/ENCFF017JVH/@@download/ENCFF017JVH.bed.gz
https://www.encodeproject.org/files/ENCFF036CYJ/@@download/ENCFF036CYJ.bed.gz
https://www.encodeproject.org/files/ENCFF327KXY/@@download/ENCFF327KXY.bed.gz
https://www.encodeproject.org/files/ENCFF860CBO/@@download/ENCFF860CBO.bed.gz
https://www.encodeproject.org/files/ENCFF999KNQ/@@download/ENCFF999KNQ.bed.gz
https://www.encodeproject.org/files/ENCFF851DEQ/@@download/ENCFF851DEQ.bed.gz
https://www.encodeproject.org/files/ENCFF606FEE/@@download/ENCFF606FEE.bed.gz
https://www.encodeproject.org/files/ENCFF070PSR/@@download/ENCFF070PSR.bed.gz
https://www.encodeproject.org/files/ENCFF036SCR/@@download/ENCFF036SCR.bed.gz
https://www.encodeproject.org/files/ENCFF600CFA/@@download/ENCFF600CFA.bed.gz
https://www.encodeproject.org/files/ENCFF571PCW/@@download/ENCFF571PCW.bed.gz
https://www.encodeproject.org/files/ENCFF363MAG/@@download/ENCFF363MAG.bed.gz
https://www.encodeproject.org/files/ENCFF558ONH/@@download/ENCFF558ONH.bed.gz
https://www.encodeproject.org/files/ENCFF382FXA/@@download/ENCFF382FXA.bed.gz
https://www.encodeproject.org/files/ENCFF833TSX/@@download/ENCFF833TSX.bed.gz
https://www.encodeproject.org/files/ENCFF384ALJ/@@download/ENCFF384ALJ.bed.gz
https://www.encodeproject.org/files/ENCFF509RCR/@@download/ENCFF509RCR.bed.gz
https://www.encodeproject.org/files/ENCFF989JUA/@@download/ENCFF989JUA.bed.gz
https://www.encodeproject.org/files/ENCFF435JAM/@@download/ENCFF435JAM.bed.gz
https://www.encodeproject.org/files/ENCFF239EJM/@@download/ENCFF239EJM.bed.gz
https://www.encodeproject.org/files/ENCFF691IPU/@@download/ENCFF691IPU.bed.gz

https://www.encodeproject.org/files/ENCFF712PGZ/@@download/ENCFF712PGZ.bed.gz
https://www.encodeproject.org/files/ENCFF351LRU/@@download/ENCFF351LRU.bed.gz
https://www.encodeproject.org/files/ENCFF228GCQ/@@download/ENCFF228GCQ.bed.gz
https://www.encodeproject.org/files/ENCFF962OEY/@@download/ENCFF962OEY.bed.gz
https://www.encodeproject.org/files/ENCFF292TAR/@@download/ENCFF292TAR.bed.gz
https://www.encodeproject.org/files/ENCFF694ZGV/@@download/ENCFF694ZGV.bed.gz
https://www.encodeproject.org/files/ENCFF407HQG/@@download/ENCFF407HQG.bed.gz
https://www.encodeproject.org/files/ENCFF257QZF/@@download/ENCFF257QZF.bed.gz
https://www.encodeproject.org/files/ENCFF135FPB/@@download/ENCFF135FPB.bed.gz
https://www.encodeproject.org/files/ENCFF792VDM/@@download/ENCFF792VDM.bed.gz
https://www.encodeproject.org/files/ENCFF025MZL/@@download/ENCFF025MZL.bed.gz
https://www.encodeproject.org/files/ENCFF627LTJ/@@download/ENCFF627LTJ.bed.gz
https://www.encodeproject.org/files/ENCFF693MYU/@@download/ENCFF693MYU.bed.gz
https://www.encodeproject.org/files/ENCFF418FRJ/@@download/ENCFF418FRJ.bed.gz
https://www.encodeproject.org/files/ENCFF226RSS/@@download/ENCFF226RSS.bed.gz
https://www.encodeproject.org/files/ENCFF052CLZ/@@download/ENCFF052CLZ.bed.gz
https://www.encodeproject.org/files/ENCFF129YTH/@@download/ENCFF129YTH.bed.gz
https://www.encodeproject.org/files/ENCFF469IKB/@@download/ENCFF469IKB.bed.gz
https://www.encodeproject.org/files/ENCFF864AXI/@@download/ENCFF864AXI.bed.gz
https://www.encodeproject.org/files/ENCFF423TZA/@@download/ENCFF423TZA.bed.gz
https://www.encodeproject.org/files/ENCFF868HMT/@@download/ENCFF868HMT.bed.gz
https://www.encodeproject.org/files/ENCFF403LBP/@@download/ENCFF403LBP.bed.gz
https://www.encodeproject.org/files/ENCFF541DBX/@@download/ENCFF541DBX.bed.gz
https://www.encodeproject.org/files/ENCFF485VLI/@@download/ENCFF485VLI.bed.gz
https://www.encodeproject.org/files/ENCFF762LPY/@@download/ENCFF762LPY.bed.gz
https://www.encodeproject.org/files/ENCFF335DOL/@@download/ENCFF335DOL.bed.gz
https://www.encodeproject.org/files/ENCFF756YOL/@@download/ENCFF756YOL.bed.gz
https://www.encodeproject.org/files/ENCFF628ZQL/@@download/ENCFF628ZQL.bed.gz
https://www.encodeproject.org/files/ENCFF435QJB/@@download/ENCFF435QJB.bed.gz
https://www.encodeproject.org/files/ENCFF232DOG/@@download/ENCFF232DOG.bed.gz
https://www.encodeproject.org/files/ENCFF144ZWB/@@download/ENCFF144ZWB.bed.gz
https://www.encodeproject.org/files/ENCFF411LPV/@@download/ENCFF411LPV.bed.gz
https://www.encodeproject.org/files/ENCFF321XTU/@@download/ENCFF321XTU.bed.gz
https://www.encodeproject.org/files/ENCFF605OIT/@@download/ENCFF605OIT.bed.gz
https://www.encodeproject.org/files/ENCFF714IQU/@@download/ENCFF714IQU.bed.gz
https://www.encodeproject.org/files/ENCFF672MXN/@@download/ENCFF672MXN.bed.gz
https://www.encodeproject.org/files/ENCFF372DVA/@@download/ENCFF372DVA.bed.gz
https://www.encodeproject.org/files/ENCFF279POO/@@download/ENCFF279POO.bed.gz
https://www.encodeproject.org/files/ENCFF689ZNH/@@download/ENCFF689ZNH.bed.gz
https://www.encodeproject.org/files/ENCFF066NBQ/@@download/ENCFF066NBQ.bed.gz
https://www.encodeproject.org/files/ENCFF314SQB/@@download/ENCFF314SQB.bed.gz
https://www.encodeproject.org/files/ENCFF437LBW/@@download/ENCFF437LBW.bed.gz
https://www.encodeproject.org/files/ENCFF325IOO/@@download/ENCFF325IOO.bed.gz
https://www.encodeproject.org/files/ENCFF994QQT/@@download/ENCFF994QQT.bed.gz
https://www.encodeproject.org/files/ENCFF153EBU/@@download/ENCFF153EBU.bed.gz
https://www.encodeproject.org/files/ENCFF348MWL/@@download/ENCFF348MWL.bed.gz
https://www.encodeproject.org/files/ENCFF524EST/@@download/ENCFF524EST.bed.gz
https://www.encodeproject.org/files/ENCFF860SHD/@@download/ENCFF860SHD.bed.gz
https://www.encodeproject.org/files/ENCFF700HZX/@@download/ENCFF700HZX.bed.gz
https://www.encodeproject.org/files/ENCFF498QYW/@@download/ENCFF498QYW.bed.gz
https://www.encodeproject.org/files/ENCFF405VWN/@@download/ENCFF405VWN.bed.gz
https://www.encodeproject.org/files/ENCFF754JIP/@@download/ENCFF754JIP.bed.gz
https://www.encodeproject.org/files/ENCFF507DHS/@@download/ENCFF507DHS.bed.gz

Supplementary Table 1

https://www.encodeproject.org/files/ENCFF557UIB/@@download/ENCFF557UIB.bed.gz
https://www.encodeproject.org/files/ENCFF564SPI/@@download/ENCFF564SPI.bed.gz
https://www.encodeproject.org/files/ENCFF028GMS/@@download/ENCFF028GMS.bed.gz
https://www.encodeproject.org/files/ENCFF771XOY/@@download/ENCFF771XOY.bed.gz
https://www.encodeproject.org/files/ENCFF975UMB/@@download/ENCFF975UMB.bed.gz
https://www.encodeproject.org/files/ENCFF788ZYA/@@download/ENCFF788ZYA.bed.gz
https://www.encodeproject.org/files/ENCFF025OLL/@@download/ENCFF025OLL.bed.gz
https://www.encodeproject.org/files/ENCFF396UUU/@@download/ENCFF396UUU.bed.gz
https://www.encodeproject.org/files/ENCFF464EQV/@@download/ENCFF464EQV.bed.gz
https://www.encodeproject.org/files/ENCFF362VON/@@download/ENCFF362VON.bed.gz
https://www.encodeproject.org/files/ENCFF548AAT/@@download/ENCFF548AAT.bed.gz
https://www.encodeproject.org/files/ENCFF676WYA/@@download/ENCFF676WYA.bed.gz
https://www.encodeproject.org/files/ENCFF200AXJ/@@download/ENCFF200AXJ.bed.gz
https://www.encodeproject.org/files/ENCFF294RNI/@@download/ENCFF294RNI.bed.gz
https://www.encodeproject.org/files/ENCFF608WWL/@@download/ENCFF608WWL.bed.gz
https://www.encodeproject.org/files/ENCFF534MPD/@@download/ENCFF534MPD.bed.gz
https://www.encodeproject.org/files/ENCFF132VFC/@@download/ENCFF132VFC.bed.gz
https://www.encodeproject.org/files/ENCFF328BTO/@@download/ENCFF328BTO.bed.gz
https://www.encodeproject.org/files/ENCFF651APA/@@download/ENCFF651APA.bed.gz
https://www.encodeproject.org/files/ENCFF733JNB/@@download/ENCFF733JNB.bed.gz
https://www.encodeproject.org/files/ENCFF581DTH/@@download/ENCFF581DTH.bed.gz
https://www.encodeproject.org/files/ENCFF231LOU/@@download/ENCFF231LOU.bed.gz
https://www.encodeproject.org/files/ENCFF109JXK/@@download/ENCFF109JXK.bed.gz
https://www.encodeproject.org/files/ENCFF202BAI/@@download/ENCFF202BAI.bed.gz
https://www.encodeproject.org/files/ENCFF489VAN/@@download/ENCFF489VAN.bed.gz
https://www.encodeproject.org/files/ENCFF373DVN/@@download/ENCFF373DVN.bed.gz
https://www.encodeproject.org/files/ENCFF645DZX/@@download/ENCFF645DZX.bed.gz
https://www.encodeproject.org/files/ENCFF718QZC/@@download/ENCFF718QZC.bed.gz
https://www.encodeproject.org/files/ENCFF808HMO/@@download/ENCFF808HMO.bed.gz
https://www.encodeproject.org/files/ENCFF080KJG/@@download/ENCFF080KJG.bed.gz
https://www.encodeproject.org/files/ENCFF270LLX/@@download/ENCFF270LLX.bed.gz
https://www.encodeproject.org/files/ENCFF925OBU/@@download/ENCFF925OBU.bed.gz
https://www.encodeproject.org/files/ENCFF874RLV/@@download/ENCFF874RLV.bed.gz
https://www.encodeproject.org/files/ENCFF538TCF/@@download/ENCFF538TCF.bed.gz
https://www.encodeproject.org/files/ENCFF449AAE/@@download/ENCFF449AAE.bed.gz
https://www.encodeproject.org/files/ENCFF323RCO/@@download/ENCFF323RCO.bed.gz
https://www.encodeproject.org/files/ENCFF179EGK/@@download/ENCFF179EGK.bed.gz
https://www.encodeproject.org/files/ENCFF358ZLQ/@@download/ENCFF358ZLQ.bed.gz
https://www.encodeproject.org/files/ENCFF161FDV/@@download/ENCFF161FDV.bed.gz
https://www.encodeproject.org/files/ENCFF227YCI/@@download/ENCFF227YCI.bed.gz
https://www.encodeproject.org/files/ENCFF307VSP/@@download/ENCFF307VSP.bed.gz
https://www.encodeproject.org/files/ENCFF334VVP/@@download/ENCFF334VVP.bed.gz
https://www.encodeproject.org/files/ENCFF891SUX/@@download/ENCFF891SUX.bed.gz
https://www.encodeproject.org/files/ENCFF680JEG/@@download/ENCFF680JEG.bed.gz
https://www.encodeproject.org/files/ENCFF917PXT/@@download/ENCFF917PXT.bed.gz
https://www.encodeproject.org/files/ENCFF495TAN/@@download/ENCFF495TAN.bed.gz
https://www.encodeproject.org/files/ENCFF698UIT/@@download/ENCFF698UIT.bed.gz
https://www.encodeproject.org/files/ENCFF616YFR/@@download/ENCFF616YFR.bed.gz
https://www.encodeproject.org/files/ENCFF743WCA/@@download/ENCFF743WCA.bed.gz
https://www.encodeproject.org/files/ENCFF368ROI/@@download/ENCFF368ROI.bed.gz
https://www.encodeproject.org/files/ENCFF834DID/@@download/ENCFF834DID.bed.gz
https://www.encodeproject.org/files/ENCFF846ZOS/@@download/ENCFF846ZOS.bed.gz
https://www.encodeproject.org/files/ENCFF233DXO/@@download/ENCFF233DXO.bed.gz

https://www.encodeproject.org/files/ENCFF797VLQ/@@download/ENCFF797VLQ.bed.gz
https://www.encodeproject.org/files/ENCFF342CPM/@@download/ENCFF342CPM.bed.gz
https://www.encodeproject.org/files/ENCFF433MPJ/@@download/ENCFF433MPJ.bed.gz
https://www.encodeproject.org/files/ENCFF272QJD/@@download/ENCFF272QJD.bed.gz
https://www.encodeproject.org/files/ENCFF733OFW/@@download/ENCFF733OFW.bed.gz
https://www.encodeproject.org/files/ENCFF196MBO/@@download/ENCFF196MBO.bed.gz
https://www.encodeproject.org/files/ENCFF833YST/@@download/ENCFF833YST.bed.gz
https://www.encodeproject.org/files/ENCFF867LEE/@@download/ENCFF867LEE.bed.gz
https://www.encodeproject.org/files/ENCFF066JWI/@@download/ENCFF066JWI.bed.gz
https://www.encodeproject.org/files/ENCFF741INS/@@download/ENCFF741INS.bed.gz
https://www.encodeproject.org/files/ENCFF517CGI/@@download/ENCFF517CGI.bed.gz
https://www.encodeproject.org/files/ENCFF649WCV/@@download/ENCFF649WCV.bed.gz
https://www.encodeproject.org/files/ENCFF885KKQ/@@download/ENCFF885KKQ.bed.gz
https://www.encodeproject.org/files/ENCFF559LDF/@@download/ENCFF559LDF.bed.gz
https://www.encodeproject.org/files/ENCFF644JKD/@@download/ENCFF644JKD.bed.gz
https://www.encodeproject.org/files/ENCFF282BOE/@@download/ENCFF282BOE.bed.gz
https://www.encodeproject.org/files/ENCFF513GHF/@@download/ENCFF513GHF.bed.gz
https://www.encodeproject.org/files/ENCFF815HSK/@@download/ENCFF815HSK.bed.gz
https://www.encodeproject.org/files/ENCFF179YWB/@@download/ENCFF179YWB.bed.gz
https://www.encodeproject.org/files/ENCFF190TRG/@@download/ENCFF190TRG.bed.gz
https://www.encodeproject.org/files/ENCFF769VAM/@@download/ENCFF769VAM.bed.gz
https://www.encodeproject.org/files/ENCFF586DZY/@@download/ENCFF586DZY.bed.gz
https://www.encodeproject.org/files/ENCFF620KKE/@@download/ENCFF620KKE.bed.gz
https://www.encodeproject.org/files/ENCFF389ULP/@@download/ENCFF389ULP.bed.gz
https://www.encodeproject.org/files/ENCFF689PNU/@@download/ENCFF689PNU.bed.gz
https://www.encodeproject.org/files/ENCFF098ZOI/@@download/ENCFF098ZOI.bed.gz
https://www.encodeproject.org/files/ENCFF665RHY/@@download/ENCFF665RHY.bed.gz
https://www.encodeproject.org/files/ENCFF354MGW/@@download/ENCFF354MGW.bed.gz
https://www.encodeproject.org/files/ENCFF634DDY/@@download/ENCFF634DDY.bed.gz
https://www.encodeproject.org/files/ENCFF175QRT/@@download/ENCFF175QRT.bed.gz
https://www.encodeproject.org/files/ENCFF866EIC/@@download/ENCFF866EIC.bed.gz
https://www.encodeproject.org/files/ENCFF402UYK/@@download/ENCFF402UYK.bed.gz
https://www.encodeproject.org/files/ENCFF226MKP/@@download/ENCFF226MKP.bed.gz
https://www.encodeproject.org/files/ENCFF163OSZ/@@download/ENCFF163OSZ.bed.gz
https://www.encodeproject.org/files/ENCFF114ZFU/@@download/ENCFF114ZFU.bed.gz
https://www.encodeproject.org/files/ENCFF703CWY/@@download/ENCFF703CWY.bed.gz
https://www.encodeproject.org/files/ENCFF092GNG/@@download/ENCFF092GNG.bed.gz
https://www.encodeproject.org/files/ENCFF882LTN/@@download/ENCFF882LTN.bed.gz
https://www.encodeproject.org/files/ENCFF481CNC/@@download/ENCFF481CNC.bed.gz
https://www.encodeproject.org/files/ENCFF767XFY/@@download/ENCFF767XFY.bed.gz
https://www.encodeproject.org/files/ENCFF736DQP/@@download/ENCFF736DQP.bed.gz
https://www.encodeproject.org/files/ENCFF079QGD/@@download/ENCFF079QGD.bed.gz
https://www.encodeproject.org/files/ENCFF445NPR/@@download/ENCFF445NPR.bed.gz
https://www.encodeproject.org/files/ENCFF938NIP/@@download/ENCFF938NIP.bed.gz
https://www.encodeproject.org/files/ENCFF745NPN/@@download/ENCFF745NPN.bed.gz
https://www.encodeproject.org/files/ENCFF846IMW/@@download/ENCFF846IMW.bed.gz
https://www.encodeproject.org/files/ENCFF869YGK/@@download/ENCFF869YGK.bed.gz
https://www.encodeproject.org/files/ENCFF302MVG/@@download/ENCFF302MVG.bed.gz
https://www.encodeproject.org/files/ENCFF007ITX/@@download/ENCFF007ITX.bed.gz
https://www.encodeproject.org/files/ENCFF542QZT/@@download/ENCFF542QZT.bed.gz
https://www.encodeproject.org/files/ENCFF588XCW/@@download/ENCFF588XCW.bed.gz
https://www.encodeproject.org/files/ENCFF299CAD/@@download/ENCFF299CAD.bed.gz
https://www.encodeproject.org/files/ENCFF023MUY/@@download/ENCFF023MUY.bed.gz

https://www.encodeproject.org/files/ENCFF270VBT/@@download/ENCFF270VBT.bed.gz

https://www.encodeproject.org/files/ENCFF468AEV/@@download/ENCFF468AEV.bed.gz

https://www.encodeproject.org/files/ENCFF935FCJ/@@download/ENCFF935FCJ.bed.gz

https://www.encodeproject.org/files/ENCFF990AVH/@@download/ENCFF990AVH.bed.gz

https://www.encodeproject.org/files/ENCFF646XTO/@@download/ENCFF646XTO.bed.gz

https://www.encodeproject.org/files/ENCFF443CQH/@@download/ENCFF443CQH.bed.gz

https://www.encodeproject.org/files/ENCFF256VHD/@@download/ENCFF256VHD.bed.gz

https://www.encodeproject.org/files/ENCFF147RBN/@@download/ENCFF147RBN.bed.gz

https://www.encodeproject.org/files/ENCFF135IYN/@@download/ENCFF135IYN.bed.gz

https://www.encodeproject.org/files/ENCFF374MIO/@@download/ENCFF374MIO.bed.gz

https://www.encodeproject.org/files/ENCFF476MXK/@@download/ENCFF476MXK.bed.gz

https://www.encodeproject.org/files/ENCFF958QPG/@@download/ENCFF958QPG.bed.gz

https://www.encodeproject.org/files/ENCFF728SVI/@@download/ENCFF728SVI.bed.gz

https://www.encodeproject.org/files/ENCFF542QLV/@@download/ENCFF542QLV.bed.gz

https://www.encodeproject.org/files/ENCFF425DIK/@@download/ENCFF425DIK.bed.gz

https://www.encodeproject.org/files/ENCFF396NYG/@@download/ENCFF396NYG.bed.gz

https://www.encodeproject.org/files/ENCFF932QYK/@@download/ENCFF932QYK.bed.gz

https://www.encodeproject.org/files/ENCFF016OGE/@@download/ENCFF016OGE.bed.gz

https://www.encodeproject.org/files/ENCFF806NLH/@@download/ENCFF806NLH.bed.gz

https://www.encodeproject.org/files/ENCFF874HRJ/@@download/ENCFF874HRJ.bed.gz

https://www.encodeproject.org/files/ENCFF880FUR/@@download/ENCFF880FUR.bed.gz

https://www.encodeproject.org/files/ENCFF075FKU/@@download/ENCFF075FKU.bed.gz

https://www.encodeproject.org/files/ENCFF251IXD/@@download/ENCFF251IXD.bed.gz

https://www.encodeproject.org/files/ENCFF618NMD/@@download/ENCFF618NMD.bed.gz

https://www.encodeproject.org/files/ENCFF616QVQ/@@download/ENCFF616QVQ.bed.gz

https://www.encodeproject.org/files/ENCFF607VAP/@@download/ENCFF607VAP.bed.gz

https://www.encodeproject.org/files/ENCFF466FUW/@@download/ENCFF466FUW.bed.gz

https://www.encodeproject.org/files/ENCFF839UGI/@@download/ENCFF839UGI.bed.gz

https://www.encodeproject.org/files/ENCFF129ZVT/@@download/ENCFF129ZVT.bed.gz

https://www.encodeproject.org/files/ENCFF500PJR/@@download/ENCFF500PJR.bed.gz

https://www.encodeproject.org/files/ENCFF174XDP/@@download/ENCFF174XDP.bed.gz

https://www.encodeproject.org/files/ENCFF252FBJ/@@download/ENCFF252FBJ.bed.gz

https://www.encodeproject.org/files/ENCFF176ZCA/@@download/ENCFF176ZCA.bed.gz

https://www.encodeproject.org/files/ENCFF089XKW/@@download/ENCFF089XKW.bed.gz

https://www.encodeproject.org/files/ENCFF022MCD/@@download/ENCFF022MCD.bed.gz

https://www.encodeproject.org/files/ENCFF348YEE/@@download/ENCFF348YEE.bed.gz

https://www.encodeproject.org/files/ENCFF185LTG/@@download/ENCFF185LTG.bed.gz

https://www.encodeproject.org/files/ENCFF004RBP/@@download/ENCFF004RBP.bed.gz

https://www.encodeproject.org/files/ENCFF972NPO/@@download/ENCFF972NPO.bed.gz

https://www.encodeproject.org/files/ENCFF968UJE/@@download/ENCFF968UJE.bed.gz

https://www.encodeproject.org/files/ENCFF667IJC/@@download/ENCFF667IJC.bed.gz

https://www.encodeproject.org/files/ENCFF768LTV/@@download/ENCFF768LTV.bed.gz

https://www.encodeproject.org/files/ENCFF624CUY/@@download/ENCFF624CUY.bed.gz

https://www.encodeproject.org/files/ENCFF855EVV/@@download/ENCFF855EVV.bed.gz

https://www.encodeproject.org/files/ENCFF834PHF/@@download/ENCFF834PHF.bed.gz

https://www.encodeproject.org/files/ENCFF767LMP/@@download/ENCFF767LMP.bed.gz

https://www.encodeproject.org/files/ENCFF759UNB/@@download/ENCFF759UNB.bed.gz

https://www.encodeproject.org/files/ENCFF986TTQ/@@download/ENCFF986TTQ.bed.gz

https://www.encodeproject.org/files/ENCFF543CLZ/@@download/ENCFF543CLZ.bed.gz

https://www.encodeproject.org/files/ENCFF028RZP/@@download/ENCFF028RZP.bed.gz

https://www.encodeproject.org/files/ENCFF067BYK/@@download/ENCFF067BYK.bed.gz

https://www.encodeproject.org/files/ENCFF228NVN/@@download/ENCFF228NVN.bed.gz

https://www.encodeproject.org/files/ENCFF911TTD/@@download/ENCFF911TTD.bed.gz

Supplementary Table 1

https://www.encodeproject.org/files/ENCFF717YOR/@@download/ENCFF717YOR.bed.gz

https://www.encodeproject.org/files/ENCFF937JXF/@@download/ENCFF937JXF.bed.gz

https://www.encodeproject.org/files/ENCFF185NAU/@@download/ENCFF185NAU.bed.gz

https://www.encodeproject.org/files/ENCFF076OEB/@@download/ENCFF076OEB.bed.gz

https://www.encodeproject.org/files/ENCFF845DVI/@@download/ENCFF845DVI.bed.gz

https://www.encodeproject.org/files/ENCFF987VRF/@@download/ENCFF987VRF.bed.gz

https://www.encodeproject.org/files/ENCFF470NNW/@@download/ENCFF470NNW.bed.gz

https://www.encodeproject.org/files/ENCFF934LQZ/@@download/ENCFF934LQZ.bed.gz

https://www.encodeproject.org/files/ENCFF350AMQ/@@download/ENCFF350AMQ.bed.gz

https://www.encodeproject.org/files/ENCFF596FFJ/@@download/ENCFF596FFJ.bed.gz

https://www.encodeproject.org/files/ENCFF622MMP/@@download/ENCFF622MMP.bed.gz

https://www.encodeproject.org/files/ENCFF704PUS/@@download/ENCFF704PUS.bed.gz

https://www.encodeproject.org/files/ENCFF226GKH/@@download/ENCFF226GKH.bed.gz

https://www.encodeproject.org/files/ENCFF422ROX/@@download/ENCFF422ROX.bed.gz

https://www.encodeproject.org/files/ENCFF673GBN/@@download/ENCFF673GBN.bed.gz

https://www.encodeproject.org/files/ENCFF801TOC/@@download/ENCFF801TOC.bed.gz

https://www.encodeproject.org/files/ENCFF480EJK/@@download/ENCFF480EJK.bed.gz

https://www.encodeproject.org/files/ENCFF877QMQ/@@download/ENCFF877QMQ.bed.gz

https://www.encodeproject.org/files/ENCFF003YLJ/@@download/ENCFF003YLJ.bed.gz

https://www.encodeproject.org/files/ENCFF248QUD/@@download/ENCFF248QUD.bed.gz

https://www.encodeproject.org/files/ENCFF882NVP/@@download/ENCFF882NVP.bed.gz

https://www.encodeproject.org/files/ENCFF484KKN/@@download/ENCFF484KKN.bed.gz

https://www.encodeproject.org/files/ENCFF509NRY/@@download/ENCFF509NRY.bed.gz

https://www.encodeproject.org/files/ENCFF223CEU/@@download/ENCFF223CEU.bed.gz

https://www.encodeproject.org/files/ENCFF289MOQ/@@download/ENCFF289MOQ.bed.gz

https://www.encodeproject.org/files/ENCFF541FYT/@@download/ENCFF541FYT.bed.gz

https://www.encodeproject.org/files/ENCFF768SLC/@@download/ENCFF768SLC.bed.gz

https://www.encodeproject.org/files/ENCFF942EQC/@@download/ENCFF942EQC.bed.gz

https://www.encodeproject.org/files/ENCFF745FBX/@@download/ENCFF745FBX.bed.gz

https://www.encodeproject.org/files/ENCFF445TVF/@@download/ENCFF445TVF.bed.gz

https://www.encodeproject.org/files/ENCFF921JEC/@@download/ENCFF921JEC.bed.gz

https://www.encodeproject.org/files/ENCFF663DIG/@@download/ENCFF663DIG.bed.gz

https://www.encodeproject.org/files/ENCFF087XEZ/@@download/ENCFF087XEZ.bed.gz

https://www.encodeproject.org/files/ENCFF423BKD/@@download/ENCFF423BKD.bed.gz

https://www.encodeproject.org/files/ENCFF108GJC/@@download/ENCFF108GJC.bed.gz

https://www.encodeproject.org/files/ENCFF128AIK/@@download/ENCFF128AIK.bed.gz

https://www.encodeproject.org/files/ENCFF622HHY/@@download/ENCFF622HHY.bed.gz

https://www.encodeproject.org/files/ENCFF853WPY/@@download/ENCFF853WPY.bed.gz

https://www.encodeproject.org/files/ENCFF491LOE/@@download/ENCFF491LOE.bed.gz

https://www.encodeproject.org/files/ENCFF823LGP/@@download/ENCFF823LGP.bed.gz

https://www.encodeproject.org/files/ENCFF223ZXD/@@download/ENCFF223ZXD.bed.gz

https://www.encodeproject.org/files/ENCFF267BAU/@@download/ENCFF267BAU.bed.gz

https://www.encodeproject.org/files/ENCFF170QKI/@@download/ENCFF170QKI.bed.gz

https://www.encodeproject.org/files/ENCFF239WMO/@@download/ENCFF239WMO.bed.gz

https://www.encodeproject.org/files/ENCFF322GBN/@@download/ENCFF322GBN.bed.gz

https://www.encodeproject.org/files/ENCFF633CLW/@@download/ENCFF633CLW.bed.gz

https://www.encodeproject.org/files/ENCFF189WHF/@@download/ENCFF189WHF.bed.gz

https://www.encodeproject.org/files/ENCFF393VDG/@@download/ENCFF393VDG.bed.gz

https://www.encodeproject.org/files/ENCFF857XNT/@@download/ENCFF857XNT.bed.gz

https://www.encodeproject.org/files/ENCFF271TFD/@@download/ENCFF271TFD.bed.gz

https://www.encodeproject.org/files/ENCFF992XPI/@@download/ENCFF992XPI.bed.gz

https://www.encodeproject.org/files/ENCFF854RKR/@@download/ENCFF854RKR.bed.gz

https://www.encodeproject.org/files/ENCFF150ABP/@@download/ENCFF150ABP.bed.gz

https://www.encodeproject.org/files/ENCFF376DXN/@@download/ENCFF376DXN.bed.gz
https://www.encodeproject.org/files/ENCFF747FMA/@@download/ENCFF747FMA.bed.gz
https://www.encodeproject.org/files/ENCFF171RBT/@@download/ENCFF171RBT.bed.gz
https://www.encodeproject.org/files/ENCFF729QNR/@@download/ENCFF729QNR.bed.gz
https://www.encodeproject.org/files/ENCFF503NAW/@@download/ENCFF503NAW.bed.gz
https://www.encodeproject.org/files/ENCFF469SGL/@@download/ENCFF469SGL.bed.gz
https://www.encodeproject.org/files/ENCFF506XWQ/@@download/ENCFF506XWQ.bed.gz
https://www.encodeproject.org/files/ENCFF887XGB/@@download/ENCFF887XGB.bed.gz
https://www.encodeproject.org/files/ENCFF252YMA/@@download/ENCFF252YMA.bed.gz
https://www.encodeproject.org/files/ENCFF991SQZ/@@download/ENCFF991SQZ.bed.gz
https://www.encodeproject.org/files/ENCFF923GAD/@@download/ENCFF923GAD.bed.gz
https://www.encodeproject.org/files/ENCFF379SGB/@@download/ENCFF379SGB.bed.gz
https://www.encodeproject.org/files/ENCFF606GLW/@@download/ENCFF606GLW.bed.gz
https://www.encodeproject.org/files/ENCFF265CIX/@@download/ENCFF265CIX.bed.gz
https://www.encodeproject.org/files/ENCFF546CLW/@@download/ENCFF546CLW.bed.gz
https://www.encodeproject.org/files/ENCFF923UEP/@@download/ENCFF923UEP.bed.gz
https://www.encodeproject.org/files/ENCFF507DLC/@@download/ENCFF507DLC.bed.gz
https://www.encodeproject.org/files/ENCFF044VMQ/@@download/ENCFF044VMQ.bed.gz
https://www.encodeproject.org/files/ENCFF006OUJ/@@download/ENCFF006OUJ.bed.gz
https://www.encodeproject.org/files/ENCFF076CAA/@@download/ENCFF076CAA.bed.gz
https://www.encodeproject.org/files/ENCFF733WIP/@@download/ENCFF733WIP.bed.gz
https://www.encodeproject.org/files/ENCFF920IEV/@@download/ENCFF920IEV.bed.gz
https://www.encodeproject.org/files/ENCFF379HKV/@@download/ENCFF379HKV.bed.gz
https://www.encodeproject.org/files/ENCFF110CNT/@@download/ENCFF110CNT.bed.gz
https://www.encodeproject.org/files/ENCFF945IDU/@@download/ENCFF945IDU.bed.gz
https://www.encodeproject.org/files/ENCFF611IFP/@@download/ENCFF611IFP.bed.gz
https://www.encodeproject.org/files/ENCFF350ZNU/@@download/ENCFF350ZNU.bed.gz
https://www.encodeproject.org/files/ENCFF781YVT/@@download/ENCFF781YVT.bed.gz
https://www.encodeproject.org/files/ENCFF421ENV/@@download/ENCFF421ENV.bed.gz
https://www.encodeproject.org/files/ENCFF864IMZ/@@download/ENCFF864IMZ.bed.gz
https://www.encodeproject.org/files/ENCFF434HDF/@@download/ENCFF434HDF.bed.gz
https://www.encodeproject.org/files/ENCFF066PWZ/@@download/ENCFF066PWZ.bed.gz
https://www.encodeproject.org/files/ENCFF656OCH/@@download/ENCFF656OCH.bed.gz
https://www.encodeproject.org/files/ENCFF981HPG/@@download/ENCFF981HPG.bed.gz
https://www.encodeproject.org/files/ENCFF175ZHF/@@download/ENCFF175ZHF.bed.gz
https://www.encodeproject.org/files/ENCFF050WTT/@@download/ENCFF050WTT.bed.gz
https://www.encodeproject.org/files/ENCFF455DJE/@@download/ENCFF455DJE.bed.gz
https://www.encodeproject.org/files/ENCFF727MDC/@@download/ENCFF727MDC.bed.gz
https://www.encodeproject.org/files/ENCFF128PQG/@@download/ENCFF128PQG.bed.gz
https://www.encodeproject.org/files/ENCFF112GTB/@@download/ENCFF112GTB.bed.gz
https://www.encodeproject.org/files/ENCFF979BDX/@@download/ENCFF979BDX.bed.gz
https://www.encodeproject.org/files/ENCFF065MTT/@@download/ENCFF065MTT.bed.gz
https://www.encodeproject.org/files/ENCFF161GSW/@@download/ENCFF161GSW.bed.gz
https://www.encodeproject.org/files/ENCFF799TEQ/@@download/ENCFF799TEQ.bed.gz
https://www.encodeproject.org/files/ENCFF049TMA/@@download/ENCFF049TMA.bed.gz
https://www.encodeproject.org/files/ENCFF323FPP/@@download/ENCFF323FPP.bed.gz
https://www.encodeproject.org/files/ENCFF435EDS/@@download/ENCFF435EDS.bed.gz
https://www.encodeproject.org/files/ENCFF294TXO/@@download/ENCFF294TXO.bed.gz
https://www.encodeproject.org/files/ENCFF577LSV/@@download/ENCFF577LSV.bed.gz
https://www.encodeproject.org/files/ENCFF897UFD/@@download/ENCFF897UFD.bed.gz
https://www.encodeproject.org/files/ENCFF466HVV/@@download/ENCFF466HVV.bed.gz
https://www.encodeproject.org/files/ENCFF704OEE/@@download/ENCFF704OEE.bed.gz
https://www.encodeproject.org/files/ENCFF563GSK/@@download/ENCFF563GSK.bed.gz

https://www.encodeproject.org/files/ENCFF485EPD/@@download/ENCFF485EPD.bed.gz
https://www.encodeproject.org/files/ENCFF642IPW/@@download/ENCFF642IPW.bed.gz
https://www.encodeproject.org/files/ENCFF591ARF/@@download/ENCFF591ARF.bed.gz
https://www.encodeproject.org/files/ENCFF773XKG/@@download/ENCFF773XKG.bed.gz
https://www.encodeproject.org/files/ENCFF797JDM/@@download/ENCFF797JDM.bed.gz
https://www.encodeproject.org/files/ENCFF181YMK/@@download/ENCFF181YMK.bed.gz
https://www.encodeproject.org/files/ENCFF840QPF/@@download/ENCFF840QPF.bed.gz
https://www.encodeproject.org/files/ENCFF808WUX/@@download/ENCFF808WUX.bed.gz
https://www.encodeproject.org/files/ENCFF343CNN/@@download/ENCFF343CNN.bed.gz
https://www.encodeproject.org/files/ENCFF547BEE/@@download/ENCFF547BEE.bed.gz
https://www.encodeproject.org/files/ENCFF261ULN/@@download/ENCFF261ULN.bed.gz
https://www.encodeproject.org/files/ENCFF774FYA/@@download/ENCFF774FYA.bed.gz
https://www.encodeproject.org/files/ENCFF336ZND/@@download/ENCFF336ZND.bed.gz
https://www.encodeproject.org/files/ENCFF389DLX/@@download/ENCFF389DLX.bed.gz
https://www.encodeproject.org/files/ENCFF547NJW/@@download/ENCFF547NJW.bed.gz
https://www.encodeproject.org/files/ENCFF536MOF/@@download/ENCFF536MOF.bed.gz
https://www.encodeproject.org/files/ENCFF160SYU/@@download/ENCFF160SYU.bed.gz
https://www.encodeproject.org/files/ENCFF023GHQ/@@download/ENCFF023GHQ.bed.gz
https://www.encodeproject.org/files/ENCFF026WPP/@@download/ENCFF026WPP.bed.gz
https://www.encodeproject.org/files/ENCFF107OQO/@@download/ENCFF107OQO.bed.gz
https://www.encodeproject.org/files/ENCFF318FXB/@@download/ENCFF318FXB.bed.gz
https://www.encodeproject.org/files/ENCFF715AKU/@@download/ENCFF715AKU.bed.gz
https://www.encodeproject.org/files/ENCFF229EII/@@download/ENCFF229EII.bed.gz
https://www.encodeproject.org/files/ENCFF930NQQ/@@download/ENCFF930NQQ.bed.gz
https://www.encodeproject.org/files/ENCFF214IHP/@@download/ENCFF214IHP.bed.gz
https://www.encodeproject.org/files/ENCFF039AUA/@@download/ENCFF039AUA.bed.gz
https://www.encodeproject.org/files/ENCFF072UWP/@@download/ENCFF072UWP.bed.gz
https://www.encodeproject.org/files/ENCFF320GJE/@@download/ENCFF320GJE.bed.gz
https://www.encodeproject.org/files/ENCFF393OKG/@@download/ENCFF393OKG.bed.gz
https://www.encodeproject.org/files/ENCFF495JYR/@@download/ENCFF495JYR.bed.gz
https://www.encodeproject.org/files/ENCFF244FQD/@@download/ENCFF244FQD.bed.gz
https://www.encodeproject.org/files/ENCFF035UIJ/@@download/ENCFF035UIJ.bed.gz
https://www.encodeproject.org/files/ENCFF913GNW/@@download/ENCFF913GNW.bed.gz
https://www.encodeproject.org/files/ENCFF987IKB/@@download/ENCFF987IKB.bed.gz
https://www.encodeproject.org/files/ENCFF549FZH/@@download/ENCFF549FZH.bed.gz
https://www.encodeproject.org/files/ENCFF233SVA/@@download/ENCFF233SVA.bed.gz
https://www.encodeproject.org/files/ENCFF007BBM/@@download/ENCFF007BBM.bed.gz
https://www.encodeproject.org/files/ENCFF156HCI/@@download/ENCFF156HCI.bed.gz
https://www.encodeproject.org/files/ENCFF053BWK/@@download/ENCFF053BWK.bed.gz
https://www.encodeproject.org/files/ENCFF623DIA/@@download/ENCFF623DIA.bed.gz
https://www.encodeproject.org/files/ENCFF402FNX/@@download/ENCFF402FNX.bed.gz
https://www.encodeproject.org/files/ENCFF741BED/@@download/ENCFF741BED.bed.gz
https://www.encodeproject.org/files/ENCFF399TIE/@@download/ENCFF399TIE.bed.gz
https://www.encodeproject.org/files/ENCFF234VTM/@@download/ENCFF234VTM.bed.gz
https://www.encodeproject.org/files/ENCFF881FZQ/@@download/ENCFF881FZQ.bed.gz
https://www.encodeproject.org/files/ENCFF095IET/@@download/ENCFF095IET.bed.gz
https://www.encodeproject.org/files/ENCFF746HAO/@@download/ENCFF746HAO.bed.gz
https://www.encodeproject.org/files/ENCFF074LXY/@@download/ENCFF074LXY.bed.gz
https://www.encodeproject.org/files/ENCFF914HYV/@@download/ENCFF914HYV.bed.gz
https://www.encodeproject.org/files/ENCFF520JZM/@@download/ENCFF520JZM.bed.gz
https://www.encodeproject.org/files/ENCFF747PUI/@@download/ENCFF747PUI.bed.gz
https://www.encodeproject.org/files/ENCFF866OIP/@@download/ENCFF866OIP.bed.gz
https://www.encodeproject.org/files/ENCFF728IYI/@@download/ENCFF728IYI.bed.gz

https://www.encodeproject.org/files/ENCFF185QCG/@@download/ENCFF185QCG.bed.gz
https://www.encodeproject.org/files/ENCFF255JJG/@@download/ENCFF255JJG.bed.gz
https://www.encodeproject.org/files/ENCFF668KJN/@@download/ENCFF668KJN.bed.gz
https://www.encodeproject.org/files/ENCFF070OJM/@@download/ENCFF070OJM.bed.gz
https://www.encodeproject.org/files/ENCFF674VEK/@@download/ENCFF674VEK.bed.gz
https://www.encodeproject.org/files/ENCFF513FOP/@@download/ENCFF513FOP.bed.gz
https://www.encodeproject.org/files/ENCFF571XUW/@@download/ENCFF571XUW.bed.gz
https://www.encodeproject.org/files/ENCFF235TGO/@@download/ENCFF235TGO.bed.gz
https://www.encodeproject.org/files/ENCFF767AVJ/@@download/ENCFF767AVJ.bed.gz
https://www.encodeproject.org/files/ENCFF163ACG/@@download/ENCFF163ACG.bed.gz
https://www.encodeproject.org/files/ENCFF719EWH/@@download/ENCFF719EWH.bed.gz
https://www.encodeproject.org/files/ENCFF302QQX/@@download/ENCFF302QQX.bed.gz
https://www.encodeproject.org/files/ENCFF559LUB/@@download/ENCFF559LUB.bed.gz
https://www.encodeproject.org/files/ENCFF654IUM/@@download/ENCFF654IUM.bed.gz
https://www.encodeproject.org/files/ENCFF396TUD/@@download/ENCFF396TUD.bed.gz
https://www.encodeproject.org/files/ENCFF518NZX/@@download/ENCFF518NZX.bed.gz
https://www.encodeproject.org/files/ENCFF540DVR/@@download/ENCFF540DVR.bed.gz
https://www.encodeproject.org/files/ENCFF250RMB/@@download/ENCFF250RMB.bed.gz
https://www.encodeproject.org/files/ENCFF899FTS/@@download/ENCFF899FTS.bed.gz
https://www.encodeproject.org/files/ENCFF579GMB/@@download/ENCFF579GMB.bed.gz
https://www.encodeproject.org/files/ENCFF875DLM/@@download/ENCFF875DLM.bed.gz
https://www.encodeproject.org/files/ENCFF807YPG/@@download/ENCFF807YPG.bed.gz
https://www.encodeproject.org/files/ENCFF248DZQ/@@download/ENCFF248DZQ.bed.gz
https://www.encodeproject.org/files/ENCFF679KFK/@@download/ENCFF679KFK.bed.gz
https://www.encodeproject.org/files/ENCFF111FTU/@@download/ENCFF111FTU.bed.gz
https://www.encodeproject.org/files/ENCFF760XJE/@@download/ENCFF760XJE.bed.gz

Supplementary Table 1

SCREEN cREs

https://www.encodeproject.org/report/?type=Annotation&encyclopedia_version=4&annotation_type=candidate+Cis-
Regulatory+Elements&organism.scientific_name=Homo+sapiens&biosample_ontology.classification=tissue&biosample_ontology.classification=primary+cell&field=
%40id&field=accession&field=targets.label&field=biosample_ontology.term_name&field=description&field=lab.title&field=award.project&field=status&field=organism.scientific_name&field=relevant_life_stag[illegible]
d=relevant_timepoint&field=relevant_timepoint_units&field=software_used.software.name&field=schema_version&field=related_files&field=alternate_accessions&field=date_released&field=dbxrefs&field=i[illegible]
_tags&field=biosample_ontology&field=documents&field=notes&field=date_created&field=submitted_by&field=references&field=award&field=aliases&field=annotation_type&field=encyclopedia_version&fie[illegible]
persedes&field=assembly&field=contributing_files&field=original_files&field=revoked_files&field=month_released&field=files&field=superseded_by&field=hub&files.file_type=bed+bed3%2B&sort=biosampl[illegible]
ogy&limit=all

https://www.encodeproject.org/files/ENCFF697GSZ/@@download/ENCFF697GSZ.bed.gz

https://www.encodeproject.org/files/ENCFF052IVI/@@download/ENCFF052IVI.bed.gz

https://www.encodeproject.org/files/ENCFF625FCU/@@download/ENCFF625FCU.bed.gz

https://www.encodeproject.org/files/ENCFF529UWB/@@download/ENCFF529UWB.bed.gz

https://www.encodeproject.org/files/ENCFF158KVT/@@download/ENCFF158KVT.bed.gz

https://www.encodeproject.org/files/ENCFF097FJF/@@download/ENCFF097FJF.bed.gz

https://www.encodeproject.org/files/ENCFF216KWB/@@download/ENCFF216KWB.bed.gz

https://www.encodeproject.org/files/ENCFF846WZC/@@download/ENCFF846WZC.bed.gz

https://www.encodeproject.org/files/ENCFF098NHL/@@download/ENCFF098NHL.bed.gz

https://www.encodeproject.org/files/ENCFF518HVH/@@download/ENCFF518HVH.bed.gz

https://www.encodeproject.org/files/ENCFF728VRE/@@download/ENCFF728VRE.bed.gz

https://www.encodeproject.org/files/ENCFF471MFR/@@download/ENCFF471MFR.bed.gz

https://www.encodeproject.org/files/ENCFF505JOA/@@download/ENCFF505JOA.bed.gz

https://www.encodeproject.org/files/ENCFF760KSK/@@download/ENCFF760KSK.bed.gz

https://www.encodeproject.org/files/ENCFF503REI/@@download/ENCFF503REI.bed.gz

https://www.encodeproject.org/files/ENCFF995XTT/@@download/ENCFF995XTT.bed.gz

https://www.encodeproject.org/files/ENCFF227XAZ/@@download/ENCFF227XAZ.bed.gz

https://www.encodeproject.org/files/ENCFF866KUO/@@download/ENCFF866KUO.bed.gz

https://www.encodeproject.org/files/ENCFF375ODC/@@download/ENCFF375ODC.bed.gz

https://www.encodeproject.org/files/ENCFF382JSL/@@download/ENCFF382JSL.bed.gz

https://www.encodeproject.org/files/ENCFF971VSU/@@download/ENCFF971VSU.bed.gz

https://www.encodeproject.org/files/ENCFF810LHY/@@download/ENCFF810LHY.bed.gz

https://www.encodeproject.org/files/ENCFF765KVD/@@download/ENCFF765KVD.bed.gz

https://www.encodeproject.org/files/ENCFF623QNC/@@download/ENCFF623QNC.bed.gz

https://www.encodeproject.org/files/ENCFF942EEI/@@download/ENCFF942EEI.bed.gz

https://www.encodeproject.org/files/ENCFF023ZTQ/@@download/ENCFF023ZTQ.bed.gz

https://www.encodeproject.org/files/ENCFF806ALM/@@download/ENCFF806ALM.bed.gz

https://www.encodeproject.org/files/ENCFF451MZN/@@download/ENCFF451MZN.bed.gz

https://www.encodeproject.org/files/ENCFF516VPL/@@download/ENCFF516VPL.bed.gz

https://www.encodeproject.org/files/ENCFF024PHK/@@download/ENCFF024PHK.bed.gz

https://www.encodeproject.org/files/ENCFF622ZWB/@@download/ENCFF622ZWB.bed.gz

https://www.encodeproject.org/files/ENCFF499JAR/@@download/ENCFF499JAR.bed.gz

https://www.encodeproject.org/files/ENCFF493ZPV/@@download/ENCFF493ZPV.bed.gz

https://www.encodeproject.org/files/ENCFF725QLM/@@download/ENCFF725QLM.bed.gz

https://www.encodeproject.org/files/ENCFF133YDZ/@@download/ENCFF133YDZ.bed.gz

https://www.encodeproject.org/files/ENCFF740KNG/@@download/ENCFF740KNG.bed.gz

https://www.encodeproject.org/files/ENCFF868YIZ/@@download/ENCFF868YIZ.bed.gz

https://www.encodeproject.org/files/ENCFF348AEV/@@download/ENCFF348AEV.bed.gz

https://www.encodeproject.org/files/ENCFF633QNH/@@download/ENCFF633QNH.bed.gz

https://www.encodeproject.org/files/ENCFF750YRT/@@download/ENCFF750YRT.bed.gz

https://www.encodeproject.org/files/ENCFF651BDQ/@@download/ENCFF651BDQ.bed.gz

https://www.encodeproject.org/files/ENCFF080ILL/@@download/ENCFF080ILL.bed.gz

https://www.encodeproject.org/files/ENCFF953RKH/@@download/ENCFF953RKH.bed.gz

https://www.encodeproject.org/files/ENCFF288QES/@@download/ENCFF288QES.bed.gz

https://www.encodeproject.org/files/ENCFF427LEK/@@download/ENCFF427LEK.bed.gz

https://www.encodeproject.org/files/ENCFF530GZB/@@download/ENCFF530GZB.bed.gz

https://www.encodeproject.org/files/ENCFF663AMF/@@download/ENCFF663AMF.bed.gz

https://www.encodeproject.org/files/ENCFF236CAP/@@download/ENCFF236CAP.bed.gz

https://www.encodeproject.org/files/ENCFF137ROK/@@download/ENCFF137ROK.bed.gz
https://www.encodeproject.org/files/ENCFF055CJM/@@download/ENCFF055CJM.bed.gz
https://www.encodeproject.org/files/ENCFF519CJL/@@download/ENCFF519CJL.bed.gz
https://www.encodeproject.org/files/ENCFF770LRR/@@download/ENCFF770LRR.bed.gz
https://www.encodeproject.org/files/ENCFF301XZH/@@download/ENCFF301XZH.bed.gz
https://www.encodeproject.org/files/ENCFF672SSU/@@download/ENCFF672SSU.bed.gz
https://www.encodeproject.org/files/ENCFF005GNA/@@download/ENCFF005GNA.bed.gz
https://www.encodeproject.org/files/ENCFF043JQE/@@download/ENCFF043JQE.bed.gz
https://www.encodeproject.org/files/ENCFF695XPO/@@download/ENCFF695XPO.bed.gz
https://www.encodeproject.org/files/ENCFF707SIJ/@@download/ENCFF707SIJ.bed.gz
https://www.encodeproject.org/files/ENCFF849FSP/@@download/ENCFF849FSP.bed.gz
https://www.encodeproject.org/files/ENCFF905VEJ/@@download/ENCFF905VEJ.bed.gz
https://www.encodeproject.org/files/ENCFF066XTG/@@download/ENCFF066XTG.bed.gz
https://www.encodeproject.org/files/ENCFF402HRM/@@download/ENCFF402HRM.bed.gz
https://www.encodeproject.org/files/ENCFF704DIE/@@download/ENCFF704DIE.bed.gz
https://www.encodeproject.org/files/ENCFF459JSC/@@download/ENCFF459JSC.bed.gz
https://www.encodeproject.org/files/ENCFF986FAC/@@download/ENCFF986FAC.bed.gz
https://www.encodeproject.org/files/ENCFF729WZG/@@download/ENCFF729WZG.bed.gz
https://www.encodeproject.org/files/ENCFF838FXE/@@download/ENCFF838FXE.bed.gz
https://www.encodeproject.org/files/ENCFF992WUW/@@download/ENCFF992WUW.bed.gz
https://www.encodeproject.org/files/ENCFF733OTT/@@download/ENCFF733OTT.bed.gz
https://www.encodeproject.org/files/ENCFF561CDY/@@download/ENCFF561CDY.bed.gz
https://www.encodeproject.org/files/ENCFF069ZQD/@@download/ENCFF069ZQD.bed.gz
https://www.encodeproject.org/files/ENCFF072UFW/@@download/ENCFF072UFW.bed.gz
https://www.encodeproject.org/files/ENCFF431IJT/@@download/ENCFF431IJT.bed.gz
https://www.encodeproject.org/files/ENCFF456ONA/@@download/ENCFF456ONA.bed.gz
https://www.encodeproject.org/files/ENCFF707RIP/@@download/ENCFF707RIP.bed.gz
https://www.encodeproject.org/files/ENCFF083APU/@@download/ENCFF083APU.bed.gz
https://www.encodeproject.org/files/ENCFF778WQF/@@download/ENCFF778WQF.bed.gz
https://www.encodeproject.org/files/ENCFF132NTX/@@download/ENCFF132NTX.bed.gz
https://www.encodeproject.org/files/ENCFF100QNG/@@download/ENCFF100QNG.bed.gz
https://www.encodeproject.org/files/ENCFF998BPH/@@download/ENCFF998BPH.bed.gz
https://www.encodeproject.org/files/ENCFF982VCJ/@@download/ENCFF982VCJ.bed.gz
https://www.encodeproject.org/files/ENCFF132WVZ/@@download/ENCFF132WVZ.bed.gz
https://www.encodeproject.org/files/ENCFF144PXA/@@download/ENCFF144PXA.bed.gz
https://www.encodeproject.org/files/ENCFF397AOA/@@download/ENCFF397AOA.bed.gz
https://www.encodeproject.org/files/ENCFF707JUZ/@@download/ENCFF707JUZ.bed.gz
https://www.encodeproject.org/files/ENCFF648UWH/@@download/ENCFF648UWH.bed.gz
https://www.encodeproject.org/files/ENCFF513NBU/@@download/ENCFF513NBU.bed.gz
https://www.encodeproject.org/files/ENCFF635YIX/@@download/ENCFF635YIX.bed.gz
https://www.encodeproject.org/files/ENCFF648LUN/@@download/ENCFF648LUN.bed.gz
https://www.encodeproject.org/files/ENCFF594PVG/@@download/ENCFF594PVG.bed.gz
https://www.encodeproject.org/files/ENCFF794UOT/@@download/ENCFF794UOT.bed.gz
https://www.encodeproject.org/files/ENCFF037UZZ/@@download/ENCFF037UZZ.bed.gz
https://www.encodeproject.org/files/ENCFF901IGB/@@download/ENCFF901IGB.bed.gz
https://www.encodeproject.org/files/ENCFF733RMG/@@download/ENCFF733RMG.bed.gz
https://www.encodeproject.org/files/ENCFF713HEC/@@download/ENCFF713HEC.bed.gz
https://www.encodeproject.org/files/ENCFF479ZBF/@@download/ENCFF479ZBF.bed.gz
https://www.encodeproject.org/files/ENCFF431HOQ/@@download/ENCFF431HOQ.bed.gz
https://www.encodeproject.org/files/ENCFF890YOJ/@@download/ENCFF890YOJ.bed.gz
https://www.encodeproject.org/files/ENCFF186EHN/@@download/ENCFF186EHN.bed.gz
https://www.encodeproject.org/files/ENCFF880IXV/@@download/ENCFF880IXV.bed.gz
https://www.encodeproject.org/files/ENCFF487SHX/@@download/ENCFF487SHX.bed.gz

Supplementary Table 1

https://www.encodeproject.org/files/ENCFF398URY/@@download/ENCFF398URY.bed.gz
https://www.encodeproject.org/files/ENCFF578TJB/@@download/ENCFF578TJB.bed.gz
https://www.encodeproject.org/files/ENCFF736LZJ/@@download/ENCFF736LZJ.bed.gz
https://www.encodeproject.org/files/ENCFF414ZOK/@@download/ENCFF414ZOK.bed.gz
https://www.encodeproject.org/files/ENCFF522ISW/@@download/ENCFF522ISW.bed.gz
https://www.encodeproject.org/files/ENCFF542WDK/@@download/ENCFF542WDK.bed.gz
https://www.encodeproject.org/files/ENCFF849TGP/@@download/ENCFF849TGP.bed.gz
https://www.encodeproject.org/files/ENCFF388TYY/@@download/ENCFF388TYY.bed.gz
https://www.encodeproject.org/files/ENCFF823FZA/@@download/ENCFF823FZA.bed.gz
https://www.encodeproject.org/files/ENCFF303JFX/@@download/ENCFF303JFX.bed.gz
https://www.encodeproject.org/files/ENCFF038IGH/@@download/ENCFF038IGH.bed.gz
https://www.encodeproject.org/files/ENCFF227EPV/@@download/ENCFF227EPV.bed.gz
https://www.encodeproject.org/files/ENCFF225INN/@@download/ENCFF225INN.bed.gz
https://www.encodeproject.org/files/ENCFF051QTK/@@download/ENCFF051QTK.bed.gz
https://www.encodeproject.org/files/ENCFF424TSP/@@download/ENCFF424TSP.bed.gz
https://www.encodeproject.org/files/ENCFF401ESC/@@download/ENCFF401ESC.bed.gz
https://www.encodeproject.org/files/ENCFF937HTU/@@download/ENCFF937HTU.bed.gz
https://www.encodeproject.org/files/ENCFF261XTE/@@download/ENCFF261XTE.bed.gz
https://www.encodeproject.org/files/ENCFF405CIV/@@download/ENCFF405CIV.bed.gz
https://www.encodeproject.org/files/ENCFF783VLL/@@download/ENCFF783VLL.bed.gz
https://www.encodeproject.org/files/ENCFF808ZRZ/@@download/ENCFF808ZRZ.bed.gz
https://www.encodeproject.org/files/ENCFF963AYK/@@download/ENCFF963AYK.bed.gz
https://www.encodeproject.org/files/ENCFF673YUR/@@download/ENCFF673YUR.bed.gz
https://www.encodeproject.org/files/ENCFF039TKU/@@download/ENCFF039TKU.bed.gz
https://www.encodeproject.org/files/ENCFF050RDD/@@download/ENCFF050RDD.bed.gz
https://www.encodeproject.org/files/ENCFF005MFL/@@download/ENCFF005MFL.bed.gz
https://www.encodeproject.org/files/ENCFF961FXT/@@download/ENCFF961FXT.bed.gz
https://www.encodeproject.org/files/ENCFF783AJR/@@download/ENCFF783AJR.bed.gz
https://www.encodeproject.org/files/ENCFF816HRJ/@@download/ENCFF816HRJ.bed.gz
https://www.encodeproject.org/files/ENCFF222SVT/@@download/ENCFF222SVT.bed.gz
https://www.encodeproject.org/files/ENCFF710UQM/@@download/ENCFF710UQM.bed.gz
https://www.encodeproject.org/files/ENCFF289FOL/@@download/ENCFF289FOL.bed.gz
https://www.encodeproject.org/files/ENCFF572TLW/@@download/ENCFF572TLW.bed.gz
https://www.encodeproject.org/files/ENCFF995WEQ/@@download/ENCFF995WEQ.bed.gz
https://www.encodeproject.org/files/ENCFF336GKS/@@download/ENCFF336GKS.bed.gz
https://www.encodeproject.org/files/ENCFF469ITP/@@download/ENCFF469ITP.bed.gz
https://www.encodeproject.org/files/ENCFF330VQI/@@download/ENCFF330VQI.bed.gz
https://www.encodeproject.org/files/ENCFF653BYH/@@download/ENCFF653BYH.bed.gz
https://www.encodeproject.org/files/ENCFF295EZT/@@download/ENCFF295EZT.bed.gz
https://www.encodeproject.org/files/ENCFF058ESK/@@download/ENCFF058ESK.bed.gz
https://www.encodeproject.org/files/ENCFF493QCA/@@download/ENCFF493QCA.bed.gz
https://www.encodeproject.org/files/ENCFF394FMA/@@download/ENCFF394FMA.bed.gz
https://www.encodeproject.org/files/ENCFF082FIQ/@@download/ENCFF082FIQ.bed.gz
https://www.encodeproject.org/files/ENCFF217LSR/@@download/ENCFF217LSR.bed.gz
https://www.encodeproject.org/files/ENCFF810UIY/@@download/ENCFF810UIY.bed.gz
https://www.encodeproject.org/files/ENCFF399UCT/@@download/ENCFF399UCT.bed.gz
https://www.encodeproject.org/files/ENCFF068YWM/@@download/ENCFF068YWM.bed.gz
https://www.encodeproject.org/files/ENCFF345DAD/@@download/ENCFF345DAD.bed.gz
https://www.encodeproject.org/files/ENCFF780JVV/@@download/ENCFF780JVV.bed.gz
https://www.encodeproject.org/files/ENCFF552RIH/@@download/ENCFF552RIH.bed.gz
https://www.encodeproject.org/files/ENCFF643CTL/@@download/ENCFF643CTL.bed.gz
https://www.encodeproject.org/files/ENCFF932HSR/@@download/ENCFF932HSR.bed.gz
https://www.encodeproject.org/files/ENCFF085HAY/@@download/ENCFF085HAY.bed.gz

Supplementary Table 1

https://www.encodeproject.org/files/ENCFF393QYW/@@download/ENCFF393QYW.bed.gz
https://www.encodeproject.org/files/ENCFF754FTY/@@download/ENCFF754FTY.bed.gz
https://www.encodeproject.org/files/ENCFF416RWE/@@download/ENCFF416RWE.bed.gz
https://www.encodeproject.org/files/ENCFF442GZN/@@download/ENCFF442GZN.bed.gz
https://www.encodeproject.org/files/ENCFF183FCX/@@download/ENCFF183FCX.bed.gz
https://www.encodeproject.org/files/ENCFF726JTT/@@download/ENCFF726JTT.bed.gz
https://www.encodeproject.org/files/ENCFF113RCV/@@download/ENCFF113RCV.bed.gz
https://www.encodeproject.org/files/ENCFF591DAC/@@download/ENCFF591DAC.bed.gz
https://www.encodeproject.org/files/ENCFF128WWL/@@download/ENCFF128WWL.bed.gz
https://www.encodeproject.org/files/ENCFF253AZO/@@download/ENCFF253AZO.bed.gz
https://www.encodeproject.org/files/ENCFF187SKV/@@download/ENCFF187SKV.bed.gz
https://www.encodeproject.org/files/ENCFF449UUG/@@download/ENCFF449UUG.bed.gz
https://www.encodeproject.org/files/ENCFF746OZP/@@download/ENCFF746OZP.bed.gz
https://www.encodeproject.org/files/ENCFF858FZP/@@download/ENCFF858FZP.bed.gz
https://www.encodeproject.org/files/ENCFF338PVZ/@@download/ENCFF338PVZ.bed.gz
https://www.encodeproject.org/files/ENCFF823NGF/@@download/ENCFF823NGF.bed.gz
https://www.encodeproject.org/files/ENCFF234XKL/@@download/ENCFF234XKL.bed.gz
https://www.encodeproject.org/files/ENCFF024GOE/@@download/ENCFF024GOE.bed.gz
https://www.encodeproject.org/files/ENCFF116QKX/@@download/ENCFF116QKX.bed.gz
https://www.encodeproject.org/files/ENCFF284NEK/@@download/ENCFF284NEK.bed.gz
https://www.encodeproject.org/files/ENCFF498LCJ/@@download/ENCFF498LCJ.bed.gz
https://www.encodeproject.org/files/ENCFF682VTN/@@download/ENCFF682VTN.bed.gz
https://www.encodeproject.org/files/ENCFF627TRY/@@download/ENCFF627TRY.bed.gz
https://www.encodeproject.org/files/ENCFF093MDL/@@download/ENCFF093MDL.bed.gz
https://www.encodeproject.org/files/ENCFF557BKY/@@download/ENCFF557BKY.bed.gz
https://www.encodeproject.org/files/ENCFF278RUJ/@@download/ENCFF278RUJ.bed.gz
https://www.encodeproject.org/files/ENCFF653SQA/@@download/ENCFF653SQA.bed.gz
https://www.encodeproject.org/files/ENCFF843YJW/@@download/ENCFF843YJW.bed.gz
https://www.encodeproject.org/files/ENCFF359RWI/@@download/ENCFF359RWI.bed.gz
https://www.encodeproject.org/files/ENCFF605FCG/@@download/ENCFF605FCG.bed.gz
https://www.encodeproject.org/files/ENCFF912KIC/@@download/ENCFF912KIC.bed.gz
https://www.encodeproject.org/files/ENCFF529PMI/@@download/ENCFF529PMI.bed.gz
https://www.encodeproject.org/files/ENCFF967VCS/@@download/ENCFF967VCS.bed.gz
https://www.encodeproject.org/files/ENCFF935PCF/@@download/ENCFF935PCF.bed.gz
https://www.encodeproject.org/files/ENCFF953RWV/@@download/ENCFF953RWV.bed.gz
https://www.encodeproject.org/files/ENCFF806JNQ/@@download/ENCFF806JNQ.bed.gz
https://www.encodeproject.org/files/ENCFF392EIR/@@download/ENCFF392EIR.bed.gz
https://www.encodeproject.org/files/ENCFF681EOT/@@download/ENCFF681EOT.bed.gz
https://www.encodeproject.org/files/ENCFF220UFR/@@download/ENCFF220UFR.bed.gz
https://www.encodeproject.org/files/ENCFF354RTY/@@download/ENCFF354RTY.bed.gz
https://www.encodeproject.org/files/ENCFF508CFM/@@download/ENCFF508CFM.bed.gz
https://www.encodeproject.org/files/ENCFF779DXI/@@download/ENCFF779DXI.bed.gz
https://www.encodeproject.org/files/ENCFF322EIN/@@download/ENCFF322EIN.bed.gz
https://www.encodeproject.org/files/ENCFF603RBB/@@download/ENCFF603RBB.bed.gz
https://www.encodeproject.org/files/ENCFF339EKY/@@download/ENCFF339EKY.bed.gz
https://www.encodeproject.org/files/ENCFF364HYH/@@download/ENCFF364HYH.bed.gz
https://www.encodeproject.org/files/ENCFF372ZIN/@@download/ENCFF372ZIN.bed.gz
https://www.encodeproject.org/files/ENCFF370GXF/@@download/ENCFF370GXF.bed.gz
https://www.encodeproject.org/files/ENCFF348OSO/@@download/ENCFF348OSO.bed.gz
https://www.encodeproject.org/files/ENCFF765GEH/@@download/ENCFF765GEH.bed.gz
https://www.encodeproject.org/files/ENCFF456CLI/@@download/ENCFF456CLI.bed.gz
https://www.encodeproject.org/files/ENCFF231XXA/@@download/ENCFF231XXA.bed.gz
https://www.encodeproject.org/files/ENCFF867TJN/@@download/ENCFF867TJN.bed.gz

https://www.encodeproject.org/files/ENCFF492HOC/@@download/ENCFF492HOC.bed.gz
https://www.encodeproject.org/files/ENCFF985TZN/@@download/ENCFF985TZN.bed.gz
https://www.encodeproject.org/files/ENCFF656WAW/@@download/ENCFF656WAW.bed.gz
https://www.encodeproject.org/files/ENCFF536UAC/@@download/ENCFF536UAC.bed.gz
https://www.encodeproject.org/files/ENCFF984PMK/@@download/ENCFF984PMK.bed.gz
https://www.encodeproject.org/files/ENCFF134BRW/@@download/ENCFF134BRW.bed.gz
https://www.encodeproject.org/files/ENCFF692UHV/@@download/ENCFF692UHV.bed.gz
https://www.encodeproject.org/files/ENCFF429KYQ/@@download/ENCFF429KYQ.bed.gz
https://www.encodeproject.org/files/ENCFF568YOQ/@@download/ENCFF568YOQ.bed.gz
https://www.encodeproject.org/files/ENCFF342YLS/@@download/ENCFF342YLS.bed.gz
https://www.encodeproject.org/files/ENCFF937EED/@@download/ENCFF937EED.bed.gz
https://www.encodeproject.org/files/ENCFF720RSL/@@download/ENCFF720RSL.bed.gz
https://www.encodeproject.org/files/ENCFF073EXW/@@download/ENCFF073EXW.bed.gz
https://www.encodeproject.org/files/ENCFF840ANN/@@download/ENCFF840ANN.bed.gz
https://www.encodeproject.org/files/ENCFF957OYM/@@download/ENCFF957OYM.bed.gz
https://www.encodeproject.org/files/ENCFF985HCI/@@download/ENCFF985HCI.bed.gz
https://www.encodeproject.org/files/ENCFF831CMX/@@download/ENCFF831CMX.bed.gz
https://www.encodeproject.org/files/ENCFF601IEG/@@download/ENCFF601IEG.bed.gz
https://www.encodeproject.org/files/ENCFF662KVX/@@download/ENCFF662KVX.bed.gz
https://www.encodeproject.org/files/ENCFF721KCY/@@download/ENCFF721KCY.bed.gz
https://www.encodeproject.org/files/ENCFF095CMH/@@download/ENCFF095CMH.bed.gz
https://www.encodeproject.org/files/ENCFF289JWO/@@download/ENCFF289JWO.bed.gz
https://www.encodeproject.org/files/ENCFF593BVU/@@download/ENCFF593BVU.bed.gz
https://www.encodeproject.org/files/ENCFF355YJW/@@download/ENCFF355YJW.bed.gz
https://www.encodeproject.org/files/ENCFF829ZRY/@@download/ENCFF829ZRY.bed.gz
https://www.encodeproject.org/files/ENCFF183SSO/@@download/ENCFF183SSO.bed.gz
https://www.encodeproject.org/files/ENCFF059PHA/@@download/ENCFF059PHA.bed.gz
https://www.encodeproject.org/files/ENCFF967MJU/@@download/ENCFF967MJU.bed.gz
https://www.encodeproject.org/files/ENCFF348RJV/@@download/ENCFF348RJV.bed.gz
https://www.encodeproject.org/files/ENCFF151JRK/@@download/ENCFF151JRK.bed.gz
https://www.encodeproject.org/files/ENCFF205EDL/@@download/ENCFF205EDL.bed.gz
https://www.encodeproject.org/files/ENCFF476MEG/@@download/ENCFF476MEG.bed.gz
https://www.encodeproject.org/files/ENCFF161TKO/@@download/ENCFF161TKO.bed.gz
https://www.encodeproject.org/files/ENCFF082SRN/@@download/ENCFF082SRN.bed.gz
https://www.encodeproject.org/files/ENCFF554BDD/@@download/ENCFF554BDD.bed.gz
https://www.encodeproject.org/files/ENCFF961YHZ/@@download/ENCFF961YHZ.bed.gz
https://www.encodeproject.org/files/ENCFF178GDW/@@download/ENCFF178GDW.bed.gz
https://www.encodeproject.org/files/ENCFF175XWM/@@download/ENCFF175XWM.bed.gz
https://www.encodeproject.org/files/ENCFF303XLT/@@download/ENCFF303XLT.bed.gz
https://www.encodeproject.org/files/ENCFF404JZG/@@download/ENCFF404JZG.bed.gz
https://www.encodeproject.org/files/ENCFF025UNX/@@download/ENCFF025UNX.bed.gz
https://www.encodeproject.org/files/ENCFF115DVR/@@download/ENCFF115DVR.bed.gz
https://www.encodeproject.org/files/ENCFF069NQY/@@download/ENCFF069NQY.bed.gz
https://www.encodeproject.org/files/ENCFF206DFM/@@download/ENCFF206DFM.bed.gz
https://www.encodeproject.org/files/ENCFF740ZVL/@@download/ENCFF740ZVL.bed.gz
https://www.encodeproject.org/files/ENCFF740AOK/@@download/ENCFF740AOK.bed.gz
https://www.encodeproject.org/files/ENCFF551KMS/@@download/ENCFF551KMS.bed.gz
https://www.encodeproject.org/files/ENCFF222HPL/@@download/ENCFF222HPL.bed.gz
https://www.encodeproject.org/files/ENCFF719ETK/@@download/ENCFF719ETK.bed.gz
https://www.encodeproject.org/files/ENCFF264OYS/@@download/ENCFF264OYS.bed.gz
https://www.encodeproject.org/files/ENCFF035PTC/@@download/ENCFF035PTC.bed.gz
https://www.encodeproject.org/files/ENCFF941BGV/@@download/ENCFF941BGV.bed.gz
https://www.encodeproject.org/files/ENCFF613ORM/@@download/ENCFF613ORM.bed.gz

Supplementary Table 1

https://www.encodeproject.org/files/ENCFF365ETC/@@download/ENCFF365ETC.bed.gz
https://www.encodeproject.org/files/ENCFF782IWF/@@download/ENCFF782IWF.bed.gz
https://www.encodeproject.org/files/ENCFF825TRE/@@download/ENCFF825TRE.bed.gz
https://www.encodeproject.org/files/ENCFF286UVK/@@download/ENCFF286UVK.bed.gz
https://www.encodeproject.org/files/ENCFF043RVP/@@download/ENCFF043RVP.bed.gz
https://www.encodeproject.org/files/ENCFF786SJZ/@@download/ENCFF786SJZ.bed.gz
https://www.encodeproject.org/files/ENCFF337QMO/@@download/ENCFF337QMO.bed.gz
https://www.encodeproject.org/files/ENCFF179OZM/@@download/ENCFF179OZM.bed.gz
https://www.encodeproject.org/files/ENCFF480EJF/@@download/ENCFF480EJF.bed.gz
https://www.encodeproject.org/files/ENCFF487YOF/@@download/ENCFF487YOF.bed.gz
https://www.encodeproject.org/files/ENCFF953TJU/@@download/ENCFF953TJU.bed.gz
https://www.encodeproject.org/files/ENCFF218UBN/@@download/ENCFF218UBN.bed.gz
https://www.encodeproject.org/files/ENCFF084EUA/@@download/ENCFF084EUA.bed.gz
https://www.encodeproject.org/files/ENCFF684YBF/@@download/ENCFF684YBF.bed.gz
https://www.encodeproject.org/files/ENCFF862BGI/@@download/ENCFF862BGI.bed.gz
https://www.encodeproject.org/files/ENCFF081XNY/@@download/ENCFF081XNY.bed.gz
https://www.encodeproject.org/files/ENCFF080AUX/@@download/ENCFF080AUX.bed.gz
https://www.encodeproject.org/files/ENCFF831PAM/@@download/ENCFF831PAM.bed.gz
https://www.encodeproject.org/files/ENCFF341ZWC/@@download/ENCFF341ZWC.bed.gz
https://www.encodeproject.org/files/ENCFF271MYX/@@download/ENCFF271MYX.bed.gz
https://www.encodeproject.org/files/ENCFF544QKF/@@download/ENCFF544QKF.bed.gz
https://www.encodeproject.org/files/ENCFF598KKD/@@download/ENCFF598KKD.bed.gz
https://www.encodeproject.org/files/ENCFF996DHI/@@download/ENCFF996DHI.bed.gz
https://www.encodeproject.org/files/ENCFF673XBT/@@download/ENCFF673XBT.bed.gz
https://www.encodeproject.org/files/ENCFF098BRI/@@download/ENCFF098BRI.bed.gz
https://www.encodeproject.org/files/ENCFF977TUJ/@@download/ENCFF977TUJ.bed.gz
https://www.encodeproject.org/files/ENCFF509DLH/@@download/ENCFF509DLH.bed.gz
https://www.encodeproject.org/files/ENCFF577PNX/@@download/ENCFF577PNX.bed.gz
https://www.encodeproject.org/files/ENCFF760CCU/@@download/ENCFF760CCU.bed.gz
https://www.encodeproject.org/files/ENCFF788TFS/@@download/ENCFF788TFS.bed.gz
https://www.encodeproject.org/files/ENCFF579ESV/@@download/ENCFF579ESV.bed.gz
https://www.encodeproject.org/files/ENCFF490LZW/@@download/ENCFF490LZW.bed.gz
https://www.encodeproject.org/files/ENCFF265IGY/@@download/ENCFF265IGY.bed.gz
https://www.encodeproject.org/files/ENCFF758HQC/@@download/ENCFF758HQC.bed.gz
https://www.encodeproject.org/files/ENCFF380FUY/@@download/ENCFF380FUY.bed.gz
https://www.encodeproject.org/files/ENCFF725EPR/@@download/ENCFF725EPR.bed.gz
https://www.encodeproject.org/files/ENCFF751WVS/@@download/ENCFF751WVS.bed.gz
https://www.encodeproject.org/files/ENCFF082FRD/@@download/ENCFF082FRD.bed.gz
https://www.encodeproject.org/files/ENCFF693UIM/@@download/ENCFF693UIM.bed.gz
https://www.encodeproject.org/files/ENCFF254CZA/@@download/ENCFF254CZA.bed.gz
https://www.encodeproject.org/files/ENCFF226MVC/@@download/ENCFF226MVC.bed.gz
https://www.encodeproject.org/files/ENCFF674DSB/@@download/ENCFF674DSB.bed.gz
https://www.encodeproject.org/files/ENCFF776RKQ/@@download/ENCFF776RKQ.bed.gz
https://www.encodeproject.org/files/ENCFF341JOR/@@download/ENCFF341JOR.bed.gz
https://www.encodeproject.org/files/ENCFF330BNE/@@download/ENCFF330BNE.bed.gz
https://www.encodeproject.org/files/ENCFF543DVJ/@@download/ENCFF543DVJ.bed.gz
https://www.encodeproject.org/files/ENCFF577MSU/@@download/ENCFF577MSU.bed.gz
https://www.encodeproject.org/files/ENCFF608CZO/@@download/ENCFF608CZO.bed.gz
https://www.encodeproject.org/files/ENCFF965UGZ/@@download/ENCFF965UGZ.bed.gz
https://www.encodeproject.org/files/ENCFF133ZQJ/@@download/ENCFF133ZQJ.bed.gz
https://www.encodeproject.org/files/ENCFF876FFK/@@download/ENCFF876FFK.bed.gz
https://www.encodeproject.org/files/ENCFF024NHU/@@download/ENCFF024NHU.bed.gz
https://www.encodeproject.org/files/ENCFF051FGI/@@download/ENCFF051FGI.bed.gz

https://www.encodeproject.org/files/ENCFF055IFC/@@download/ENCFF055IFC.bed.gz
https://www.encodeproject.org/files/ENCFF627GSG/@@download/ENCFF627GSG.bed.gz
https://www.encodeproject.org/files/ENCFF096KIK/@@download/ENCFF096KIK.bed.gz
https://www.encodeproject.org/files/ENCFF283KZZ/@@download/ENCFF283KZZ.bed.gz
https://www.encodeproject.org/files/ENCFF356UHI/@@download/ENCFF356UHI.bed.gz
https://www.encodeproject.org/files/ENCFF357QNI/@@download/ENCFF357QNI.bed.gz
https://www.encodeproject.org/files/ENCFF683YMU/@@download/ENCFF683YMU.bed.gz
https://www.encodeproject.org/files/ENCFF739NCT/@@download/ENCFF739NCT.bed.gz
https://www.encodeproject.org/files/ENCFF178GTK/@@download/ENCFF178GTK.bed.gz
https://www.encodeproject.org/files/ENCFF098RKQ/@@download/ENCFF098RKQ.bed.gz
https://www.encodeproject.org/files/ENCFF967ZKN/@@download/ENCFF967ZKN.bed.gz
https://www.encodeproject.org/files/ENCFF946XSM/@@download/ENCFF946XSM.bed.gz
https://www.encodeproject.org/files/ENCFF283XEW/@@download/ENCFF283XEW.bed.gz
https://www.encodeproject.org/files/ENCFF144JLM/@@download/ENCFF144JLM.bed.gz
https://www.encodeproject.org/files/ENCFF697CXP/@@download/ENCFF697CXP.bed.gz
https://www.encodeproject.org/files/ENCFF363RDZ/@@download/ENCFF363RDZ.bed.gz
https://www.encodeproject.org/files/ENCFF484OVD/@@download/ENCFF484OVD.bed.gz
https://www.encodeproject.org/files/ENCFF047IQQ/@@download/ENCFF047IQQ.bed.gz
https://www.encodeproject.org/files/ENCFF641HDY/@@download/ENCFF641HDY.bed.gz
https://www.encodeproject.org/files/ENCFF455OCN/@@download/ENCFF455OCN.bed.gz
https://www.encodeproject.org/files/ENCFF784QUR/@@download/ENCFF784QUR.bed.gz
https://www.encodeproject.org/files/ENCFF179UGG/@@download/ENCFF179UGG.bed.gz
https://www.encodeproject.org/files/ENCFF203XLN/@@download/ENCFF203XLN.bed.gz
https://www.encodeproject.org/files/ENCFF323DPV/@@download/ENCFF323DPV.bed.gz
https://www.encodeproject.org/files/ENCFF669LFS/@@download/ENCFF669LFS.bed.gz
https://www.encodeproject.org/files/ENCFF306FGX/@@download/ENCFF306FGX.bed.gz
https://www.encodeproject.org/files/ENCFF253AWN/@@download/ENCFF253AWN.bed.gz
https://www.encodeproject.org/files/ENCFF217MLR/@@download/ENCFF217MLR.bed.gz
https://www.encodeproject.org/files/ENCFF810IQU/@@download/ENCFF810IQU.bed.gz
https://www.encodeproject.org/files/ENCFF756CUZ/@@download/ENCFF756CUZ.bed.gz
https://www.encodeproject.org/files/ENCFF941JIE/@@download/ENCFF941JIE.bed.gz
https://www.encodeproject.org/files/ENCFF323QHP/@@download/ENCFF323QHP.bed.gz
https://www.encodeproject.org/files/ENCFF356XKJ/@@download/ENCFF356XKJ.bed.gz
https://www.encodeproject.org/files/ENCFF802YXQ/@@download/ENCFF802YXQ.bed.gz
https://www.encodeproject.org/files/ENCFF839YJV/@@download/ENCFF839YJV.bed.gz
https://www.encodeproject.org/files/ENCFF381YPQ/@@download/ENCFF381YPQ.bed.gz
https://www.encodeproject.org/files/ENCFF718WMQ/@@download/ENCFF718WMQ.bed.gz
https://www.encodeproject.org/files/ENCFF368NIK/@@download/ENCFF368NIK.bed.gz
https://www.encodeproject.org/files/ENCFF241SXE/@@download/ENCFF241SXE.bed.gz
https://www.encodeproject.org/files/ENCFF751CCZ/@@download/ENCFF751CCZ.bed.gz
https://www.encodeproject.org/files/ENCFF611IMR/@@download/ENCFF611IMR.bed.gz
https://www.encodeproject.org/files/ENCFF038LAU/@@download/ENCFF038LAU.bed.gz
https://www.encodeproject.org/files/ENCFF889FYQ/@@download/ENCFF889FYQ.bed.gz
https://www.encodeproject.org/files/ENCFF687KJI/@@download/ENCFF687KJI.bed.gz
https://www.encodeproject.org/files/ENCFF586NXH/@@download/ENCFF586NXH.bed.gz
https://www.encodeproject.org/files/ENCFF126PHE/@@download/ENCFF126PHE.bed.gz
https://www.encodeproject.org/files/ENCFF771UBH/@@download/ENCFF771UBH.bed.gz
https://www.encodeproject.org/files/ENCFF116NKB/@@download/ENCFF116NKB.bed.gz
https://www.encodeproject.org/files/ENCFF023XVP/@@download/ENCFF023XVP.bed.gz
https://www.encodeproject.org/files/ENCFF167YAT/@@download/ENCFF167YAT.bed.gz
https://www.encodeproject.org/files/ENCFF155CXG/@@download/ENCFF155CXG.bed.gz
https://www.encodeproject.org/files/ENCFF614SGM/@@download/ENCFF614SGM.bed.gz
https://www.encodeproject.org/files/ENCFF059QBC/@@download/ENCFF059QBC.bed.gz

https://www.encodeproject.org/files/ENCFF878VCR/@@download/ENCFF878VCR.bed.gz
https://www.encodeproject.org/files/ENCFF379TAE/@@download/ENCFF379TAE.bed.gz
https://www.encodeproject.org/files/ENCFF578DNQ/@@download/ENCFF578DNQ.bed.gz
https://www.encodeproject.org/files/ENCFF179NIU/@@download/ENCFF179NIU.bed.gz
https://www.encodeproject.org/files/ENCFF512FJV/@@download/ENCFF512FJV.bed.gz
https://www.encodeproject.org/files/ENCFF494WCN/@@download/ENCFF494WCN.bed.gz
https://www.encodeproject.org/files/ENCFF207BUC/@@download/ENCFF207BUC.bed.gz
https://www.encodeproject.org/files/ENCFF532KSQ/@@download/ENCFF532KSQ.bed.gz
https://www.encodeproject.org/files/ENCFF881NYW/@@download/ENCFF881NYW.bed.gz
https://www.encodeproject.org/files/ENCFF123OBY/@@download/ENCFF123OBY.bed.gz
https://www.encodeproject.org/files/ENCFF483JUF/@@download/ENCFF483JUF.bed.gz
https://www.encodeproject.org/files/ENCFF464SXS/@@download/ENCFF464SXS.bed.gz
https://www.encodeproject.org/files/ENCFF080EPK/@@download/ENCFF080EPK.bed.gz
https://www.encodeproject.org/files/ENCFF932RJS/@@download/ENCFF932RJS.bed.gz
https://www.encodeproject.org/files/ENCFF237HLI/@@download/ENCFF237HLI.bed.gz
https://www.encodeproject.org/files/ENCFF603MCZ/@@download/ENCFF603MCZ.bed.gz
https://www.encodeproject.org/files/ENCFF564RCE/@@download/ENCFF564RCE.bed.gz
https://www.encodeproject.org/files/ENCFF510ERX/@@download/ENCFF510ERX.bed.gz
https://www.encodeproject.org/files/ENCFF831RDE/@@download/ENCFF831RDE.bed.gz
https://www.encodeproject.org/files/ENCFF344FQS/@@download/ENCFF344FQS.bed.gz
https://www.encodeproject.org/files/ENCFF669GMM/@@download/ENCFF669GMM.bed.gz
https://www.encodeproject.org/files/ENCFF252IDZ/@@download/ENCFF252IDZ.bed.gz
https://www.encodeproject.org/files/ENCFF027OZY/@@download/ENCFF027OZY.bed.gz
https://www.encodeproject.org/files/ENCFF547YDB/@@download/ENCFF547YDB.bed.gz
https://www.encodeproject.org/files/ENCFF942KAC/@@download/ENCFF942KAC.bed.gz
https://www.encodeproject.org/files/ENCFF251FTP/@@download/ENCFF251FTP.bed.gz
https://www.encodeproject.org/files/ENCFF455SVK/@@download/ENCFF455SVK.bed.gz
https://www.encodeproject.org/files/ENCFF821QIK/@@download/ENCFF821QIK.bed.gz
https://www.encodeproject.org/files/ENCFF797FDV/@@download/ENCFF797FDV.bed.gz
https://www.encodeproject.org/files/ENCFF437RMZ/@@download/ENCFF437RMZ.bed.gz
https://www.encodeproject.org/files/ENCFF888GJS/@@download/ENCFF888GJS.bed.gz
https://www.encodeproject.org/files/ENCFF328PGB/@@download/ENCFF328PGB.bed.gz
https://www.encodeproject.org/files/ENCFF898NSK/@@download/ENCFF898NSK.bed.gz
https://www.encodeproject.org/files/ENCFF837BHT/@@download/ENCFF837BHT.bed.gz
https://www.encodeproject.org/files/ENCFF305RMG/@@download/ENCFF305RMG.bed.gz
https://www.encodeproject.org/files/ENCFF509DPX/@@download/ENCFF509DPX.bed.gz
https://www.encodeproject.org/files/ENCFF330YIP/@@download/ENCFF330YIP.bed.gz
https://www.encodeproject.org/files/ENCFF267QJU/@@download/ENCFF267QJU.bed.gz
https://www.encodeproject.org/files/ENCFF929PUX/@@download/ENCFF929PUX.bed.gz
https://www.encodeproject.org/files/ENCFF854RND/@@download/ENCFF854RND.bed.gz
https://www.encodeproject.org/files/ENCFF282SRX/@@download/ENCFF282SRX.bed.gz
https://www.encodeproject.org/files/ENCFF543PJT/@@download/ENCFF543PJT.bed.gz
https://www.encodeproject.org/files/ENCFF977VPY/@@download/ENCFF977VPY.bed.gz
https://www.encodeproject.org/files/ENCFF930TFE/@@download/ENCFF930TFE.bed.gz
https://www.encodeproject.org/files/ENCFF981TYC/@@download/ENCFF981TYC.bed.gz
https://www.encodeproject.org/files/ENCFF699UMY/@@download/ENCFF699UMY.bed.gz
https://www.encodeproject.org/files/ENCFF833CQA/@@download/ENCFF833CQA.bed.gz
https://www.encodeproject.org/files/ENCFF678HQI/@@download/ENCFF678HQI.bed.gz
https://www.encodeproject.org/files/ENCFF792AMA/@@download/ENCFF792AMA.bed.gz
https://www.encodeproject.org/files/ENCFF490SDP/@@download/ENCFF490SDP.bed.gz
https://www.encodeproject.org/files/ENCFF287ZXQ/@@download/ENCFF287ZXQ.bed.gz
https://www.encodeproject.org/files/ENCFF883ZAL/@@download/ENCFF883ZAL.bed.gz
https://www.encodeproject.org/files/ENCFF248HRV/@@download/ENCFF248HRV.bed.gz

https://www.encodeproject.org/files/ENCFF413VPA/@@download/ENCFF413VPA.bed.gz
https://www.encodeproject.org/files/ENCFF727WYP/@@download/ENCFF727WYP.bed.gz
https://www.encodeproject.org/files/ENCFF283UUB/@@download/ENCFF283UUB.bed.gz
https://www.encodeproject.org/files/ENCFF088NJH/@@download/ENCFF088NJH.bed.gz
https://www.encodeproject.org/files/ENCFF658TQG/@@download/ENCFF658TQG.bed.gz
https://www.encodeproject.org/files/ENCFF632VQY/@@download/ENCFF632VQY.bed.gz
https://www.encodeproject.org/files/ENCFF095DLT/@@download/ENCFF095DLT.bed.gz
https://www.encodeproject.org/files/ENCFF067TCB/@@download/ENCFF067TCB.bed.gz
https://www.encodeproject.org/files/ENCFF102UTF/@@download/ENCFF102UTF.bed.gz
https://www.encodeproject.org/files/ENCFF438LUB/@@download/ENCFF438LUB.bed.gz
https://www.encodeproject.org/files/ENCFF995IOW/@@download/ENCFF995IOW.bed.gz
https://www.encodeproject.org/files/ENCFF132SVE/@@download/ENCFF132SVE.bed.gz
https://www.encodeproject.org/files/ENCFF354HMB/@@download/ENCFF354HMB.bed.gz
https://www.encodeproject.org/files/ENCFF744JZU/@@download/ENCFF744JZU.bed.gz
https://www.encodeproject.org/files/ENCFF988FNC/@@download/ENCFF988FNC.bed.gz
https://www.encodeproject.org/files/ENCFF561NGZ/@@download/ENCFF561NGZ.bed.gz
https://www.encodeproject.org/files/ENCFF811HUR/@@download/ENCFF811HUR.bed.gz
https://www.encodeproject.org/files/ENCFF634MEO/@@download/ENCFF634MEO.bed.gz
https://www.encodeproject.org/files/ENCFF449LXB/@@download/ENCFF449LXB.bed.gz
https://www.encodeproject.org/files/ENCFF314QVZ/@@download/ENCFF314QVZ.bed.gz
https://www.encodeproject.org/files/ENCFF645PQQ/@@download/ENCFF645PQQ.bed.gz
https://www.encodeproject.org/files/ENCFF370FFT/@@download/ENCFF370FFT.bed.gz
https://www.encodeproject.org/files/ENCFF115AGU/@@download/ENCFF115AGU.bed.gz
https://www.encodeproject.org/files/ENCFF794RIO/@@download/ENCFF794RIO.bed.gz
https://www.encodeproject.org/files/ENCFF900BGT/@@download/ENCFF900BGT.bed.gz
https://www.encodeproject.org/files/ENCFF127GRP/@@download/ENCFF127GRP.bed.gz
https://www.encodeproject.org/files/ENCFF738LRU/@@download/ENCFF738LRU.bed.gz
https://www.encodeproject.org/files/ENCFF014AQR/@@download/ENCFF014AQR.bed.gz
https://www.encodeproject.org/files/ENCFF835FYW/@@download/ENCFF835FYW.bed.gz
https://www.encodeproject.org/files/ENCFF614GJT/@@download/ENCFF614GJT.bed.gz
https://www.encodeproject.org/files/ENCFF877UTR/@@download/ENCFF877UTR.bed.gz
https://www.encodeproject.org/files/ENCFF948JBG/@@download/ENCFF948JBG.bed.gz
https://www.encodeproject.org/files/ENCFF002RLZ/@@download/ENCFF002RLZ.bed.gz
https://www.encodeproject.org/files/ENCFF144EQZ/@@download/ENCFF144EQZ.bed.gz
https://www.encodeproject.org/files/ENCFF058DMA/@@download/ENCFF058DMA.bed.gz
https://www.encodeproject.org/files/ENCFF506GEP/@@download/ENCFF506GEP.bed.gz
https://www.encodeproject.org/files/ENCFF918ZWH/@@download/ENCFF918ZWH.bed.gz
https://www.encodeproject.org/files/ENCFF249IGM/@@download/ENCFF249IGM.bed.gz
https://www.encodeproject.org/files/ENCFF377DLY/@@download/ENCFF377DLY.bed.gz
https://www.encodeproject.org/files/ENCFF474MUM/@@download/ENCFF474MUM.bed.gz
https://www.encodeproject.org/files/ENCFF786QSA/@@download/ENCFF786QSA.bed.gz
https://www.encodeproject.org/files/ENCFF479LKG/@@download/ENCFF479LKG.bed.gz
https://www.encodeproject.org/files/ENCFF245AMN/@@download/ENCFF245AMN.bed.gz
https://www.encodeproject.org/files/ENCFF848HTT/@@download/ENCFF848HTT.bed.gz
https://www.encodeproject.org/files/ENCFF627IDZ/@@download/ENCFF627IDZ.bed.gz
https://www.encodeproject.org/files/ENCFF402NNV/@@download/ENCFF402NNV.bed.gz
https://www.encodeproject.org/files/ENCFF225VYC/@@download/ENCFF225VYC.bed.gz
https://www.encodeproject.org/files/ENCFF835GGZ/@@download/ENCFF835GGZ.bed.gz
https://www.encodeproject.org/files/ENCFF159NZA/@@download/ENCFF159NZA.bed.gz
https://www.encodeproject.org/files/ENCFF949VFY/@@download/ENCFF949VFY.bed.gz
https://www.encodeproject.org/files/ENCFF410ASB/@@download/ENCFF410ASB.bed.gz
https://www.encodeproject.org/files/ENCFF508GKP/@@download/ENCFF508GKP.bed.gz
https://www.encodeproject.org/files/ENCFF108HDV/@@download/ENCFF108HDV.bed.gz

https://www.encodeproject.org/files/ENCFF491HXG/@@download/ENCFF491HXG.bed.gz
https://www.encodeproject.org/files/ENCFF791VTB/@@download/ENCFF791VTB.bed.gz
https://www.encodeproject.org/files/ENCFF721QQT/@@download/ENCFF721QQT.bed.gz
https://www.encodeproject.org/files/ENCFF806YXI/@@download/ENCFF806YXI.bed.gz
https://www.encodeproject.org/files/ENCFF448VYX/@@download/ENCFF448VYX.bed.gz
https://www.encodeproject.org/files/ENCFF274KRI/@@download/ENCFF274KRI.bed.gz
https://www.encodeproject.org/files/ENCFF082XYS/@@download/ENCFF082XYS.bed.gz
https://www.encodeproject.org/files/ENCFF298UHT/@@download/ENCFF298UHT.bed.gz
https://www.encodeproject.org/files/ENCFF111PGT/@@download/ENCFF111PGT.bed.gz
https://www.encodeproject.org/files/ENCFF775BVI/@@download/ENCFF775BVI.bed.gz
https://www.encodeproject.org/files/ENCFF067YHO/@@download/ENCFF067YHO.bed.gz
https://www.encodeproject.org/files/ENCFF726IPM/@@download/ENCFF726IPM.bed.gz
https://www.encodeproject.org/files/ENCFF117MCB/@@download/ENCFF117MCB.bed.gz
https://www.encodeproject.org/files/ENCFF810GHH/@@download/ENCFF810GHH.bed.gz
https://www.encodeproject.org/files/ENCFF070EXF/@@download/ENCFF070EXF.bed.gz
https://www.encodeproject.org/files/ENCFF835IAW/@@download/ENCFF835IAW.bed.gz
https://www.encodeproject.org/files/ENCFF113VYB/@@download/ENCFF113VYB.bed.gz
https://www.encodeproject.org/files/ENCFF369CBR/@@download/ENCFF369CBR.bed.gz
https://www.encodeproject.org/files/ENCFF074VON/@@download/ENCFF074VON.bed.gz
https://www.encodeproject.org/files/ENCFF244MXA/@@download/ENCFF244MXA.bed.gz
https://www.encodeproject.org/files/ENCFF970WQF/@@download/ENCFF970WQF.bed.gz
https://www.encodeproject.org/files/ENCFF233VRB/@@download/ENCFF233VRB.bed.gz
https://www.encodeproject.org/files/ENCFF351RSH/@@download/ENCFF351RSH.bed.gz
https://www.encodeproject.org/files/ENCFF516XXS/@@download/ENCFF516XXS.bed.gz
https://www.encodeproject.org/files/ENCFF602OWO/@@download/ENCFF602OWO.bed.gz
https://www.encodeproject.org/files/ENCFF554HJE/@@download/ENCFF554HJE.bed.gz
https://www.encodeproject.org/files/ENCFF122ORV/@@download/ENCFF122ORV.bed.gz
https://www.encodeproject.org/files/ENCFF952ABT/@@download/ENCFF952ABT.bed.gz
https://www.encodeproject.org/files/ENCFF315WDO/@@download/ENCFF315WDO.bed.gz
https://www.encodeproject.org/files/ENCFF717ILJ/@@download/ENCFF717ILJ.bed.gz
https://www.encodeproject.org/files/ENCFF621BDL/@@download/ENCFF621BDL.bed.gz
https://www.encodeproject.org/files/ENCFF673ETL/@@download/ENCFF673ETL.bed.gz
https://www.encodeproject.org/files/ENCFF047DFA/@@download/ENCFF047DFA.bed.gz
https://www.encodeproject.org/files/ENCFF937ZBL/@@download/ENCFF937ZBL.bed.gz
https://www.encodeproject.org/files/ENCFF773YHG/@@download/ENCFF773YHG.bed.gz
https://www.encodeproject.org/files/ENCFF723DYF/@@download/ENCFF723DYF.bed.gz
https://www.encodeproject.org/files/ENCFF392UNO/@@download/ENCFF392UNO.bed.gz
https://www.encodeproject.org/files/ENCFF899CCD/@@download/ENCFF899CCD.bed.gz
https://www.encodeproject.org/files/ENCFF247IBC/@@download/ENCFF247IBC.bed.gz
https://www.encodeproject.org/files/ENCFF678TOB/@@download/ENCFF678TOB.bed.gz
https://www.encodeproject.org/files/ENCFF926GLH/@@download/ENCFF926GLH.bed.gz
https://www.encodeproject.org/files/ENCFF190HQP/@@download/ENCFF190HQP.bed.gz
https://www.encodeproject.org/files/ENCFF454EYZ/@@download/ENCFF454EYZ.bed.gz
https://www.encodeproject.org/files/ENCFF718AJN/@@download/ENCFF718AJN.bed.gz
https://www.encodeproject.org/files/ENCFF683KSB/@@download/ENCFF683KSB.bed.gz
https://www.encodeproject.org/files/ENCFF260IDN/@@download/ENCFF260IDN.bed.gz
https://www.encodeproject.org/files/ENCFF164XSB/@@download/ENCFF164XSB.bed.gz
https://www.encodeproject.org/files/ENCFF623KLE/@@download/ENCFF623KLE.bed.gz
https://www.encodeproject.org/files/ENCFF845ILV/@@download/ENCFF845ILV.bed.gz
https://www.encodeproject.org/files/ENCFF626ZLG/@@download/ENCFF626ZLG.bed.gz
https://www.encodeproject.org/files/ENCFF287KHD/@@download/ENCFF287KHD.bed.gz
https://www.encodeproject.org/files/ENCFF971GRY/@@download/ENCFF971GRY.bed.gz
https://www.encodeproject.org/files/ENCFF210YNK/@@download/ENCFF210YNK.bed.gz

https://www.encodeproject.org/files/ENCFF482IJM/@@download/ENCFF482IJM.bed.gz
https://www.encodeproject.org/files/ENCFF657CPG/@@download/ENCFF657CPG.bed.gz
https://www.encodeproject.org/files/ENCFF274OGC/@@download/ENCFF274OGC.bed.gz
https://www.encodeproject.org/files/ENCFF188UOD/@@download/ENCFF188UOD.bed.gz
https://www.encodeproject.org/files/ENCFF529BGM/@@download/ENCFF529BGM.bed.gz
https://www.encodeproject.org/files/ENCFF309OAJ/@@download/ENCFF309OAJ.bed.gz
https://www.encodeproject.org/files/ENCFF850TVH/@@download/ENCFF850TVH.bed.gz
https://www.encodeproject.org/files/ENCFF635ERG/@@download/ENCFF635ERG.bed.gz
https://www.encodeproject.org/files/ENCFF644BWH/@@download/ENCFF644BWH.bed.gz
https://www.encodeproject.org/files/ENCFF913CMT/@@download/ENCFF913CMT.bed.gz
https://www.encodeproject.org/files/ENCFF025HPW/@@download/ENCFF025HPW.bed.gz
https://www.encodeproject.org/files/ENCFF223XME/@@download/ENCFF223XME.bed.gz
https://www.encodeproject.org/files/ENCFF746EGY/@@download/ENCFF746EGY.bed.gz
https://www.encodeproject.org/files/ENCFF759LOA/@@download/ENCFF759LOA.bed.gz
https://www.encodeproject.org/files/ENCFF707IJH/@@download/ENCFF707IJH.bed.gz
https://www.encodeproject.org/files/ENCFF959NAB/@@download/ENCFF959NAB.bed.gz
https://www.encodeproject.org/files/ENCFF138NTW/@@download/ENCFF138NTW.bed.gz
https://www.encodeproject.org/files/ENCFF861NJF/@@download/ENCFF861NJF.bed.gz
https://www.encodeproject.org/files/ENCFF800YES/@@download/ENCFF800YES.bed.gz
https://www.encodeproject.org/files/ENCFF202HAL/@@download/ENCFF202HAL.bed.gz
https://www.encodeproject.org/files/ENCFF424YWG/@@download/ENCFF424YWG.bed.gz
https://www.encodeproject.org/files/ENCFF463WLS/@@download/ENCFF463WLS.bed.gz
https://www.encodeproject.org/files/ENCFF013GRB/@@download/ENCFF013GRB.bed.gz
https://www.encodeproject.org/files/ENCFF248OTB/@@download/ENCFF248OTB.bed.gz
https://www.encodeproject.org/files/ENCFF412NTU/@@download/ENCFF412NTU.bed.gz
https://www.encodeproject.org/files/ENCFF886RYV/@@download/ENCFF886RYV.bed.gz
https://www.encodeproject.org/files/ENCFF582GNL/@@download/ENCFF582GNL.bed.gz
https://www.encodeproject.org/files/ENCFF460TGC/@@download/ENCFF460TGC.bed.gz
https://www.encodeproject.org/files/ENCFF786UGS/@@download/ENCFF786UGS.bed.gz
https://www.encodeproject.org/files/ENCFF568SDS/@@download/ENCFF568SDS.bed.gz
https://www.encodeproject.org/files/ENCFF284IMI/@@download/ENCFF284IMI.bed.gz
https://www.encodeproject.org/files/ENCFF475MBO/@@download/ENCFF475MBO.bed.gz
https://www.encodeproject.org/files/ENCFF026NVH/@@download/ENCFF026NVH.bed.gz
https://www.encodeproject.org/files/ENCFF716XPO/@@download/ENCFF716XPO.bed.gz
https://www.encodeproject.org/files/ENCFF768HKV/@@download/ENCFF768HKV.bed.gz
https://www.encodeproject.org/files/ENCFF116UBZ/@@download/ENCFF116UBZ.bed.gz
https://www.encodeproject.org/files/ENCFF268IIL/@@download/ENCFF268IIL.bed.gz
https://www.encodeproject.org/files/ENCFF585MPQ/@@download/ENCFF585MPQ.bed.gz
https://www.encodeproject.org/files/ENCFF516ODL/@@download/ENCFF516ODL.bed.gz
https://www.encodeproject.org/files/ENCFF438JPV/@@download/ENCFF438JPV.bed.gz
https://www.encodeproject.org/files/ENCFF306KKQ/@@download/ENCFF306KKQ.bed.gz
https://www.encodeproject.org/files/ENCFF254NBN/@@download/ENCFF254NBN.bed.gz
https://www.encodeproject.org/files/ENCFF248MPL/@@download/ENCFF248MPL.bed.gz
https://www.encodeproject.org/files/ENCFF229KXO/@@download/ENCFF229KXO.bed.gz
https://www.encodeproject.org/files/ENCFF446WAB/@@download/ENCFF446WAB.bed.gz
https://www.encodeproject.org/files/ENCFF464COM/@@download/ENCFF464COM.bed.gz
https://www.encodeproject.org/files/ENCFF792JXG/@@download/ENCFF792JXG.bed.gz
https://www.encodeproject.org/files/ENCFF572HYL/@@download/ENCFF572HYL.bed.gz
https://www.encodeproject.org/files/ENCFF975XYF/@@download/ENCFF975XYF.bed.gz
https://www.encodeproject.org/files/ENCFF822CEI/@@download/ENCFF822CEI.bed.gz
https://www.encodeproject.org/files/ENCFF953YJN/@@download/ENCFF953YJN.bed.gz
https://www.encodeproject.org/files/ENCFF093SFQ/@@download/ENCFF093SFQ.bed.gz
https://www.encodeproject.org/files/ENCFF346GKF/@@download/ENCFF346GKF.bed.gz

Supplementary Table 1

https://www.encodeproject.org/files/ENCFF727BWZ/@@download/ENCFF727BWZ.bed.gz
https://www.encodeproject.org/files/ENCFF687VAE/@@download/ENCFF687VAE.bed.gz
https://www.encodeproject.org/files/ENCFF116NTS/@@download/ENCFF116NTS.bed.gz
https://www.encodeproject.org/files/ENCFF485ANA/@@download/ENCFF485ANA.bed.gz
https://www.encodeproject.org/files/ENCFF055JRL/@@download/ENCFF055JRL.bed.gz
https://www.encodeproject.org/files/ENCFF571UFN/@@download/ENCFF571UFN.bed.gz
https://www.encodeproject.org/files/ENCFF306NRN/@@download/ENCFF306NRN.bed.gz
https://www.encodeproject.org/files/ENCFF189SXL/@@download/ENCFF189SXL.bed.gz
https://www.encodeproject.org/files/ENCFF555TLF/@@download/ENCFF555TLF.bed.gz
https://www.encodeproject.org/files/ENCFF263KYC/@@download/ENCFF263KYC.bed.gz
https://www.encodeproject.org/files/ENCFF071UVR/@@download/ENCFF071UVR.bed.gz
https://www.encodeproject.org/files/ENCFF058LHW/@@download/ENCFF058LHW.bed.gz
https://www.encodeproject.org/files/ENCFF699DKM/@@download/ENCFF699DKM.bed.gz
https://www.encodeproject.org/files/ENCFF118ODG/@@download/ENCFF118ODG.bed.gz
https://www.encodeproject.org/files/ENCFF548MOQ/@@download/ENCFF548MOQ.bed.gz
https://www.encodeproject.org/files/ENCFF852GPA/@@download/ENCFF852GPA.bed.gz
https://www.encodeproject.org/files/ENCFF660JDD/@@download/ENCFF660JDD.bed.gz
https://www.encodeproject.org/files/ENCFF316YQP/@@download/ENCFF316YQP.bed.gz
https://www.encodeproject.org/files/ENCFF338YSD/@@download/ENCFF338YSD.bed.gz
https://www.encodeproject.org/files/ENCFF803VFI/@@download/ENCFF803VFI.bed.gz
https://www.encodeproject.org/files/ENCFF913EEV/@@download/ENCFF913EEV.bed.gz
https://www.encodeproject.org/files/ENCFF005GEE/@@download/ENCFF005GEE.bed.gz
https://www.encodeproject.org/files/ENCFF875YRD/@@download/ENCFF875YRD.bed.gz
https://www.encodeproject.org/files/ENCFF992HIZ/@@download/ENCFF992HIZ.bed.gz
https://www.encodeproject.org/files/ENCFF466HCI/@@download/ENCFF466HCI.bed.gz
https://www.encodeproject.org/files/ENCFF900NQT/@@download/ENCFF900NQT.bed.gz
https://www.encodeproject.org/files/ENCFF918IAJ/@@download/ENCFF918IAJ.bed.gz
https://www.encodeproject.org/files/ENCFF337SLL/@@download/ENCFF337SLL.bed.gz
https://www.encodeproject.org/files/ENCFF755HOD/@@download/ENCFF755HOD.bed.gz
https://www.encodeproject.org/files/ENCFF915IHU/@@download/ENCFF915IHU.bed.gz
https://www.encodeproject.org/files/ENCFF663ANR/@@download/ENCFF663ANR.bed.gz
https://www.encodeproject.org/files/ENCFF348RHC/@@download/ENCFF348RHC.bed.gz
https://www.encodeproject.org/files/ENCFF119ARG/@@download/ENCFF119ARG.bed.gz
https://www.encodeproject.org/files/ENCFF923DBX/@@download/ENCFF923DBX.bed.gz
https://www.encodeproject.org/files/ENCFF188USG/@@download/ENCFF188USG.bed.gz
https://www.encodeproject.org/files/ENCFF065JJR/@@download/ENCFF065JJR.bed.gz
https://www.encodeproject.org/files/ENCFF395DAZ/@@download/ENCFF395DAZ.bed.gz
https://www.encodeproject.org/files/ENCFF439BMK/@@download/ENCFF439BMK.bed.gz
https://www.encodeproject.org/files/ENCFF399YCU/@@download/ENCFF399YCU.bed.gz
https://www.encodeproject.org/files/ENCFF987VBG/@@download/ENCFF987VBG.bed.gz
https://www.encodeproject.org/files/ENCFF148SBK/@@download/ENCFF148SBK.bed.gz
https://www.encodeproject.org/files/ENCFF493BPV/@@download/ENCFF493BPV.bed.gz
https://www.encodeproject.org/files/ENCFF229LLS/@@download/ENCFF229LLS.bed.gz
https://www.encodeproject.org/files/ENCFF260YAY/@@download/ENCFF260YAY.bed.gz
https://www.encodeproject.org/files/ENCFF127BYS/@@download/ENCFF127BYS.bed.gz
https://www.encodeproject.org/files/ENCFF598QTT/@@download/ENCFF598QTT.bed.gz
https://www.encodeproject.org/files/ENCFF167FUX/@@download/ENCFF167FUX.bed.gz
https://www.encodeproject.org/files/ENCFF117CWJ/@@download/ENCFF117CWJ.bed.gz
https://www.encodeproject.org/files/ENCFF552MDX/@@download/ENCFF552MDX.bed.gz
https://www.encodeproject.org/files/ENCFF679ITN/@@download/ENCFF679ITN.bed.gz
https://www.encodeproject.org/files/ENCFF308AMU/@@download/ENCFF308AMU.bed.gz
https://www.encodeproject.org/files/ENCFF799FSB/@@download/ENCFF799FSB.bed.gz
https://www.encodeproject.org/files/ENCFF456UIA/@@download/ENCFF456UIA.bed.gz

Supplementary Table 1

https://www.encodeproject.org/files/ENCFF398HIB/@@download/ENCFF398HIB.bed.gz

https://www.encodeproject.org/files/ENCFF657QMD/@@download/ENCFF657QMD.bed.gz

https://www.encodeproject.org/files/ENCFF275UVL/@@download/ENCFF275UVL.bed.gz

https://www.encodeproject.org/files/ENCFF298SDR/@@download/ENCFF298SDR.bed.gz

https://www.encodeproject.org/files/ENCFF806NHO/@@download/ENCFF806NHO.bed.gz

https://www.encodeproject.org/files/ENCFF821XFX/@@download/ENCFF821XFX.bed.gz

https://www.encodeproject.org/files/ENCFF356HAP/@@download/ENCFF356HAP.bed.gz

https://www.encodeproject.org/files/ENCFF394BVY/@@download/ENCFF394BVY.bed.gz

https://www.encodeproject.org/files/ENCFF198ABE/@@download/ENCFF198ABE.bed.gz

https://www.encodeproject.org/files/ENCFF205YMJ/@@download/ENCFF205YMJ.bed.gz

https://www.encodeproject.org/files/ENCFF799WYZ/@@download/ENCFF799WYZ.bed.gz

https://www.encodeproject.org/files/ENCFF481NZV/@@download/ENCFF481NZV.bed.gz

https://www.encodeproject.org/files/ENCFF246EBI/@@download/ENCFF246EBI.bed.gz

https://www.encodeproject.org/files/ENCFF877UKM/@@download/ENCFF877UKM.bed.gz

https://www.encodeproject.org/files/ENCFF534GAZ/@@download/ENCFF534GAZ.bed.gz

https://www.encodeproject.org/files/ENCFF708IEW/@@download/ENCFF708IEW.bed.gz

https://www.encodeproject.org/files/ENCFF426OHP/@@download/ENCFF426OHP.bed.gz

https://www.encodeproject.org/files/ENCFF736GKJ/@@download/ENCFF736GKJ.bed.gz

https://www.encodeproject.org/files/ENCFF907UMA/@@download/ENCFF907UMA.bed.gz

https://www.encodeproject.org/files/ENCFF296SZK/@@download/ENCFF296SZK.bed.gz

https://www.encodeproject.org/files/ENCFF424EYY/@@download/ENCFF424EYY.bed.gz

https://www.encodeproject.org/files/ENCFF815UTJ/@@download/ENCFF815UTJ.bed.gz

https://www.encodeproject.org/files/ENCFF010SQF/@@download/ENCFF010SQF.bed.gz

https://www.encodeproject.org/files/ENCFF085LOV/@@download/ENCFF085LOV.bed.gz

https://www.encodeproject.org/files/ENCFF488YPI/@@download/ENCFF488YPI.bed.gz

https://www.encodeproject.org/files/ENCFF352IHV/@@download/ENCFF352IHV.bed.gz

https://www.encodeproject.org/files/ENCFF952ZNE/@@download/ENCFF952ZNE.bed.gz

https://www.encodeproject.org/files/ENCFF079VYY/@@download/ENCFF079VYY.bed.gz

https://www.encodeproject.org/files/ENCFF317LJX/@@download/ENCFF317LJX.bed.gz

https://www.encodeproject.org/files/ENCFF698VYI/@@download/ENCFF698VYI.bed.gz

https://www.encodeproject.org/files/ENCFF173JMR/@@download/ENCFF173JMR.bed.gz

https://www.encodeproject.org/files/ENCFF418THE/@@download/ENCFF418THE.bed.gz

https://www.encodeproject.org/files/ENCFF518QHP/@@download/ENCFF518QHP.bed.gz

https://www.encodeproject.org/files/ENCFF073SYL/@@download/ENCFF073SYL.bed.gz

https://www.encodeproject.org/files/ENCFF544ECF/@@download/ENCFF544ECF.bed.gz

https://www.encodeproject.org/files/ENCFF047DIR/@@download/ENCFF047DIR.bed.gz

https://www.encodeproject.org/files/ENCFF883LWA/@@download/ENCFF883LWA.bed.gz

https://www.encodeproject.org/files/ENCFF150STQ/@@download/ENCFF150STQ.bed.gz

https://www.encodeproject.org/files/ENCFF220SDJ/@@download/ENCFF220SDJ.bed.gz

https://www.encodeproject.org/files/ENCFF850HCP/@@download/ENCFF850HCP.bed.gz

https://www.encodeproject.org/files/ENCFF914EBJ/@@download/ENCFF914EBJ.bed.gz

https://www.encodeproject.org/files/ENCFF318RDL/@@download/ENCFF318RDL.bed.gz

https://www.encodeproject.org/files/ENCFF491JHY/@@download/ENCFF491JHY.bed.gz

https://www.encodeproject.org/files/ENCFF154PKN/@@download/ENCFF154PKN.bed.gz

https://www.encodeproject.org/files/ENCFF542LOT/@@download/ENCFF542LOT.bed.gz

https://www.encodeproject.org/files/ENCFF145GBT/@@download/ENCFF145GBT.bed.gz

https://www.encodeproject.org/files/ENCFF500DGK/@@download/ENCFF500DGK.bed.gz

https://www.encodeproject.org/files/ENCFF226UZW/@@download/ENCFF226UZW.bed.gz

https://www.encodeproject.org/files/ENCFF052KNT/@@download/ENCFF052KNT.bed.gz

https://www.encodeproject.org/files/ENCFF098GSW/@@download/ENCFF098GSW.bed.gz

https://www.encodeproject.org/files/ENCFF509JZU/@@download/ENCFF509JZU.bed.gz

https://www.encodeproject.org/files/ENCFF378MUZ/@@download/ENCFF378MUZ.bed.gz

https://www.encodeproject.org/files/ENCFF621SIB/@@download/ENCFF621SIB.bed.gz

https://www.encodeproject.org/files/ENCFF416SRE/@@download/ENCFF416SRE.bed.gz
https://www.encodeproject.org/files/ENCFF931CXU/@@download/ENCFF931CXU.bed.gz
https://www.encodeproject.org/files/ENCFF483WRG/@@download/ENCFF483WRG.bed.gz
https://www.encodeproject.org/files/ENCFF160YXN/@@download/ENCFF160YXN.bed.gz
https://www.encodeproject.org/files/ENCFF056KUA/@@download/ENCFF056KUA.bed.gz
https://www.encodeproject.org/files/ENCFF557ZKA/@@download/ENCFF557ZKA.bed.gz
https://www.encodeproject.org/files/ENCFF313VOM/@@download/ENCFF313VOM.bed.gz
https://www.encodeproject.org/files/ENCFF389FLT/@@download/ENCFF389FLT.bed.gz
https://www.encodeproject.org/files/ENCFF997BMC/@@download/ENCFF997BMC.bed.gz
https://www.encodeproject.org/files/ENCFF832DRI/@@download/ENCFF832DRI.bed.gz
https://www.encodeproject.org/files/ENCFF764KHJ/@@download/ENCFF764KHJ.bed.gz
https://www.encodeproject.org/files/ENCFF861SRE/@@download/ENCFF861SRE.bed.gz
https://www.encodeproject.org/files/ENCFF405NYU/@@download/ENCFF405NYU.bed.gz
https://www.encodeproject.org/files/ENCFF909EMZ/@@download/ENCFF909EMZ.bed.gz
https://www.encodeproject.org/files/ENCFF342MXT/@@download/ENCFF342MXT.bed.gz
https://www.encodeproject.org/files/ENCFF575XWL/@@download/ENCFF575XWL.bed.gz
https://www.encodeproject.org/files/ENCFF518TVR/@@download/ENCFF518TVR.bed.gz
https://www.encodeproject.org/files/ENCFF785MCM/@@download/ENCFF785MCM.bed.gz
https://www.encodeproject.org/files/ENCFF186CWH/@@download/ENCFF186CWH.bed.gz
https://www.encodeproject.org/files/ENCFF555BLZ/@@download/ENCFF555BLZ.bed.gz
https://www.encodeproject.org/files/ENCFF942YYG/@@download/ENCFF942YYG.bed.gz
https://www.encodeproject.org/files/ENCFF212NOS/@@download/ENCFF212NOS.bed.gz
https://www.encodeproject.org/files/ENCFF851PAH/@@download/ENCFF851PAH.bed.gz
https://www.encodeproject.org/files/ENCFF203BYO/@@download/ENCFF203BYO.bed.gz
https://www.encodeproject.org/files/ENCFF317DRV/@@download/ENCFF317DRV.bed.gz
https://www.encodeproject.org/files/ENCFF322RAX/@@download/ENCFF322RAX.bed.gz
https://www.encodeproject.org/files/ENCFF814HEC/@@download/ENCFF814HEC.bed.gz
https://www.encodeproject.org/files/ENCFF770QMJ/@@download/ENCFF770QMJ.bed.gz
https://www.encodeproject.org/files/ENCFF297WXV/@@download/ENCFF297WXV.bed.gz
https://www.encodeproject.org/files/ENCFF016IKD/@@download/ENCFF016IKD.bed.gz
https://www.encodeproject.org/files/ENCFF547PKP/@@download/ENCFF547PKP.bed.gz
https://www.encodeproject.org/files/ENCFF680CQF/@@download/ENCFF680CQF.bed.gz
https://www.encodeproject.org/files/ENCFF365KNL/@@download/ENCFF365KNL.bed.gz
https://www.encodeproject.org/files/ENCFF260LTM/@@download/ENCFF260LTM.bed.gz
https://www.encodeproject.org/files/ENCFF251BZF/@@download/ENCFF251BZF.bed.gz
https://www.encodeproject.org/files/ENCFF826UGF/@@download/ENCFF826UGF.bed.gz
https://www.encodeproject.org/files/ENCFF579BDZ/@@download/ENCFF579BDZ.bed.gz
https://www.encodeproject.org/files/ENCFF758XHR/@@download/ENCFF758XHR.bed.gz
https://www.encodeproject.org/files/ENCFF021LEU/@@download/ENCFF021LEU.bed.gz
https://www.encodeproject.org/files/ENCFF160QTS/@@download/ENCFF160QTS.bed.gz
https://www.encodeproject.org/files/ENCFF351FLS/@@download/ENCFF351FLS.bed.gz
https://www.encodeproject.org/files/ENCFF495QKC/@@download/ENCFF495QKC.bed.gz
https://www.encodeproject.org/files/ENCFF166QIT/@@download/ENCFF166QIT.bed.gz
https://www.encodeproject.org/files/ENCFF544RLB/@@download/ENCFF544RLB.bed.gz
https://www.encodeproject.org/files/ENCFF727TZS/@@download/ENCFF727TZS.bed.gz
https://www.encodeproject.org/files/ENCFF073JON/@@download/ENCFF073JON.bed.gz
https://www.encodeproject.org/files/ENCFF931KJY/@@download/ENCFF931KJY.bed.gz
https://www.encodeproject.org/files/ENCFF292NZP/@@download/ENCFF292NZP.bed.gz
https://www.encodeproject.org/files/ENCFF171HWH/@@download/ENCFF171HWH.bed.gz
https://www.encodeproject.org/files/ENCFF394LOL/@@download/ENCFF394LOL.bed.gz
https://www.encodeproject.org/files/ENCFF567QBH/@@download/ENCFF567QBH.bed.gz
https://www.encodeproject.org/files/ENCFF844TQP/@@download/ENCFF844TQP.bed.gz
https://www.encodeproject.org/files/ENCFF835CLD/@@download/ENCFF835CLD.bed.gz

https://www.encodeproject.org/files/ENCFF307FGI/@@download/ENCFF307FGI.bed.gz
https://www.encodeproject.org/files/ENCFF290KQV/@@download/ENCFF290KQV.bed.gz
https://www.encodeproject.org/files/ENCFF334MUD/@@download/ENCFF334MUD.bed.gz
https://www.encodeproject.org/files/ENCFF066NFS/@@download/ENCFF066NFS.bed.gz
https://www.encodeproject.org/files/ENCFF393UUZ/@@download/ENCFF393UUZ.bed.gz
https://www.encodeproject.org/files/ENCFF914CVQ/@@download/ENCFF914CVQ.bed.gz
https://www.encodeproject.org/files/ENCFF854NWJ/@@download/ENCFF854NWJ.bed.gz
https://www.encodeproject.org/files/ENCFF497NQR/@@download/ENCFF497NQR.bed.gz
https://www.encodeproject.org/files/ENCFF980AUB/@@download/ENCFF980AUB.bed.gz
https://www.encodeproject.org/files/ENCFF783VRQ/@@download/ENCFF783VRQ.bed.gz
https://www.encodeproject.org/files/ENCFF158FFO/@@download/ENCFF158FFO.bed.gz
https://www.encodeproject.org/files/ENCFF183YVN/@@download/ENCFF183YVN.bed.gz
https://www.encodeproject.org/files/ENCFF686TMW/@@download/ENCFF686TMW.bed.gz
https://www.encodeproject.org/files/ENCFF763ROU/@@download/ENCFF763ROU.bed.gz
https://www.encodeproject.org/files/ENCFF938YWJ/@@download/ENCFF938YWJ.bed.gz
https://www.encodeproject.org/files/ENCFF302UFM/@@download/ENCFF302UFM.bed.gz
https://www.encodeproject.org/files/ENCFF495RTY/@@download/ENCFF495RTY.bed.gz
https://www.encodeproject.org/files/ENCFF104EMQ/@@download/ENCFF104EMQ.bed.gz
https://www.encodeproject.org/files/ENCFF364TET/@@download/ENCFF364TET.bed.gz
https://www.encodeproject.org/files/ENCFF485CAM/@@download/ENCFF485CAM.bed.gz
https://www.encodeproject.org/files/ENCFF916BYN/@@download/ENCFF916BYN.bed.gz
https://www.encodeproject.org/files/ENCFF481ZAX/@@download/ENCFF481ZAX.bed.gz
https://www.encodeproject.org/files/ENCFF283JOC/@@download/ENCFF283JOC.bed.gz
https://www.encodeproject.org/files/ENCFF662HCT/@@download/ENCFF662HCT.bed.gz
https://www.encodeproject.org/files/ENCFF773MBD/@@download/ENCFF773MBD.bed.gz
https://www.encodeproject.org/files/ENCFF599PGC/@@download/ENCFF599PGC.bed.gz
https://www.encodeproject.org/files/ENCFF501VMI/@@download/ENCFF501VMI.bed.gz
https://www.encodeproject.org/files/ENCFF737CBH/@@download/ENCFF737CBH.bed.gz
https://www.encodeproject.org/files/ENCFF248HMV/@@download/ENCFF248HMV.bed.gz
https://www.encodeproject.org/files/ENCFF472MIL/@@download/ENCFF472MIL.bed.gz
https://www.encodeproject.org/files/ENCFF896SHZ/@@download/ENCFF896SHZ.bed.gz
https://www.encodeproject.org/files/ENCFF845AIH/@@download/ENCFF845AIH.bed.gz
https://www.encodeproject.org/files/ENCFF218DVF/@@download/ENCFF218DVF.bed.gz
https://www.encodeproject.org/files/ENCFF614HUN/@@download/ENCFF614HUN.bed.gz
https://www.encodeproject.org/files/ENCFF399QNN/@@download/ENCFF399QNN.bed.gz
https://www.encodeproject.org/files/ENCFF540PLW/@@download/ENCFF540PLW.bed.gz
https://www.encodeproject.org/files/ENCFF792NHV/@@download/ENCFF792NHV.bed.gz
https://www.encodeproject.org/files/ENCFF032ZKF/@@download/ENCFF032ZKF.bed.gz
https://www.encodeproject.org/files/ENCFF550DTC/@@download/ENCFF550DTC.bed.gz
https://www.encodeproject.org/files/ENCFF269VDL/@@download/ENCFF269VDL.bed.gz
https://www.encodeproject.org/files/ENCFF100XWO/@@download/ENCFF100XWO.bed.gz
https://www.encodeproject.org/files/ENCFF818WQY/@@download/ENCFF818WQY.bed.gz
https://www.encodeproject.org/files/ENCFF646OUE/@@download/ENCFF646OUE.bed.gz
https://www.encodeproject.org/files/ENCFF584YVL/@@download/ENCFF584YVL.bed.gz
https://www.encodeproject.org/files/ENCFF907GET/@@download/ENCFF907GET.bed.gz
https://www.encodeproject.org/files/ENCFF290OFM/@@download/ENCFF290OFM.bed.gz
https://www.encodeproject.org/files/ENCFF396FMM/@@download/ENCFF396FMM.bed.gz
https://www.encodeproject.org/files/ENCFF369PMK/@@download/ENCFF369PMK.bed.gz
https://www.encodeproject.org/files/ENCFF749IYG/@@download/ENCFF749IYG.bed.gz
https://www.encodeproject.org/files/ENCFF647RSA/@@download/ENCFF647RSA.bed.gz
https://www.encodeproject.org/files/ENCFF725NNB/@@download/ENCFF725NNB.bed.gz
https://www.encodeproject.org/files/ENCFF597VUG/@@download/ENCFF597VUG.bed.gz
https://www.encodeproject.org/files/ENCFF112VVJ/@@download/ENCFF112VVJ.bed.gz

https://www.encodeproject.org/files/ENCFF630FSN/@@download/ENCFF630FSN.bed.gz
https://www.encodeproject.org/files/ENCFF861OLF/@@download/ENCFF861OLF.bed.gz
https://www.encodeproject.org/files/ENCFF747OWN/@@download/ENCFF747OWN.bed.gz
https://www.encodeproject.org/files/ENCFF809OAP/@@download/ENCFF809OAP.bed.gz
https://www.encodeproject.org/files/ENCFF752HGZ/@@download/ENCFF752HGZ.bed.gz
https://www.encodeproject.org/files/ENCFF619EIT/@@download/ENCFF619EIT.bed.gz
https://www.encodeproject.org/files/ENCFF168HSH/@@download/ENCFF168HSH.bed.gz
https://www.encodeproject.org/files/ENCFF279XKB/@@download/ENCFF279XKB.bed.gz
https://www.encodeproject.org/files/ENCFF556WJF/@@download/ENCFF556WJF.bed.gz
https://www.encodeproject.org/files/ENCFF369AIC/@@download/ENCFF369AIC.bed.gz
https://www.encodeproject.org/files/ENCFF719QCH/@@download/ENCFF719QCH.bed.gz
https://www.encodeproject.org/files/ENCFF345CKO/@@download/ENCFF345CKO.bed.gz
https://www.encodeproject.org/files/ENCFF624PLW/@@download/ENCFF624PLW.bed.gz
https://www.encodeproject.org/files/ENCFF771HAS/@@download/ENCFF771HAS.bed.gz
https://www.encodeproject.org/files/ENCFF212TQN/@@download/ENCFF212TQN.bed.gz
https://www.encodeproject.org/files/ENCFF290VSA/@@download/ENCFF290VSA.bed.gz
https://www.encodeproject.org/files/ENCFF402OKG/@@download/ENCFF402OKG.bed.gz
https://www.encodeproject.org/files/ENCFF343SBA/@@download/ENCFF343SBA.bed.gz
https://www.encodeproject.org/files/ENCFF422MDT/@@download/ENCFF422MDT.bed.gz
https://www.encodeproject.org/files/ENCFF845BSG/@@download/ENCFF845BSG.bed.gz
https://www.encodeproject.org/files/ENCFF427JFX/@@download/ENCFF427JFX.bed.gz
https://www.encodeproject.org/files/ENCFF422GMV/@@download/ENCFF422GMV.bed.gz
https://www.encodeproject.org/files/ENCFF367RST/@@download/ENCFF367RST.bed.gz
https://www.encodeproject.org/files/ENCFF716CXV/@@download/ENCFF716CXV.bed.gz
https://www.encodeproject.org/files/ENCFF348MDM/@@download/ENCFF348MDM.bed.gz
https://www.encodeproject.org/files/ENCFF291ODP/@@download/ENCFF291ODP.bed.gz
https://www.encodeproject.org/files/ENCFF079KAZ/@@download/ENCFF079KAZ.bed.gz
https://www.encodeproject.org/files/ENCFF323ZQO/@@download/ENCFF323ZQO.bed.gz
https://www.encodeproject.org/files/ENCFF259NWL/@@download/ENCFF259NWL.bed.gz
https://www.encodeproject.org/files/ENCFF648XOK/@@download/ENCFF648XOK.bed.gz
https://www.encodeproject.org/files/ENCFF324EFY/@@download/ENCFF324EFY.bed.gz
https://www.encodeproject.org/files/ENCFF718FJN/@@download/ENCFF718FJN.bed.gz
https://www.encodeproject.org/files/ENCFF781BOK/@@download/ENCFF781BOK.bed.gz
https://www.encodeproject.org/files/ENCFF759YFL/@@download/ENCFF759YFL.bed.gz
https://www.encodeproject.org/files/ENCFF110LZX/@@download/ENCFF110LZX.bed.gz
https://www.encodeproject.org/files/ENCFF222HHP/@@download/ENCFF222HHP.bed.gz
https://www.encodeproject.org/files/ENCFF197MQD/@@download/ENCFF197MQD.bed.gz
https://www.encodeproject.org/files/ENCFF891FUQ/@@download/ENCFF891FUQ.bed.gz
https://www.encodeproject.org/files/ENCFF874PDN/@@download/ENCFF874PDN.bed.gz
https://www.encodeproject.org/files/ENCFF565OED/@@download/ENCFF565OED.bed.gz
https://www.encodeproject.org/files/ENCFF897CRR/@@download/ENCFF897CRR.bed.gz
https://www.encodeproject.org/files/ENCFF442FIH/@@download/ENCFF442FIH.bed.gz
https://www.encodeproject.org/files/ENCFF227AGE/@@download/ENCFF227AGE.bed.gz
https://www.encodeproject.org/files/ENCFF235YOZ/@@download/ENCFF235YOZ.bed.gz
https://www.encodeproject.org/files/ENCFF987CUM/@@download/ENCFF987CUM.bed.gz
https://www.encodeproject.org/files/ENCFF746EUO/@@download/ENCFF746EUO.bed.gz
https://www.encodeproject.org/files/ENCFF654SFN/@@download/ENCFF654SFN.bed.gz
https://www.encodeproject.org/files/ENCFF903RGX/@@download/ENCFF903RGX.bed.gz
https://www.encodeproject.org/files/ENCFF475OPC/@@download/ENCFF475OPC.bed.gz
https://www.encodeproject.org/files/ENCFF841KFM/@@download/ENCFF841KFM.bed.gz
https://www.encodeproject.org/files/ENCFF063XVV/@@download/ENCFF063XVV.bed.gz
https://www.encodeproject.org/files/ENCFF863OGG/@@download/ENCFF863OGG.bed.gz
https://www.encodeproject.org/files/ENCFF020SII/@@download/ENCFF020SII.bed.gz

https://www.encodeproject.org/files/ENCFF488CWN/@@download/ENCFF488CWN.bed.gz
https://www.encodeproject.org/files/ENCFF761FPO/@@download/ENCFF761FPO.bed.gz
https://www.encodeproject.org/files/ENCFF835IWS/@@download/ENCFF835IWS.bed.gz
https://www.encodeproject.org/files/ENCFF256NFT/@@download/ENCFF256NFT.bed.gz
https://www.encodeproject.org/files/ENCFF242OYW/@@download/ENCFF242OYW.bed.gz
https://www.encodeproject.org/files/ENCFF043VIM/@@download/ENCFF043VIM.bed.gz
https://www.encodeproject.org/files/ENCFF384EJB/@@download/ENCFF384EJB.bed.gz
https://www.encodeproject.org/files/ENCFF463HXS/@@download/ENCFF463HXS.bed.gz
https://www.encodeproject.org/files/ENCFF312EYQ/@@download/ENCFF312EYQ.bed.gz
https://www.encodeproject.org/files/ENCFF400CKV/@@download/ENCFF400CKV.bed.gz
https://www.encodeproject.org/files/ENCFF013DAM/@@download/ENCFF013DAM.bed.gz
https://www.encodeproject.org/files/ENCFF550MPR/@@download/ENCFF550MPR.bed.gz
https://www.encodeproject.org/files/ENCFF782RLY/@@download/ENCFF782RLY.bed.gz
https://www.encodeproject.org/files/ENCFF142UKF/@@download/ENCFF142UKF.bed.gz
https://www.encodeproject.org/files/ENCFF619VOG/@@download/ENCFF619VOG.bed.gz
https://www.encodeproject.org/files/ENCFF730HNQ/@@download/ENCFF730HNQ.bed.gz
https://www.encodeproject.org/files/ENCFF213HUR/@@download/ENCFF213HUR.bed.gz
https://www.encodeproject.org/files/ENCFF666ICF/@@download/ENCFF666ICF.bed.gz
https://www.encodeproject.org/files/ENCFF558NVB/@@download/ENCFF558NVB.bed.gz
https://www.encodeproject.org/files/ENCFF372WYY/@@download/ENCFF372WYY.bed.gz
https://www.encodeproject.org/files/ENCFF403IPC/@@download/ENCFF403IPC.bed.gz
https://www.encodeproject.org/files/ENCFF597EGN/@@download/ENCFF597EGN.bed.gz
https://www.encodeproject.org/files/ENCFF775TZL/@@download/ENCFF775TZL.bed.gz
https://www.encodeproject.org/files/ENCFF682BOJ/@@download/ENCFF682BOJ.bed.gz
https://www.encodeproject.org/files/ENCFF812GTX/@@download/ENCFF812GTX.bed.gz
https://www.encodeproject.org/files/ENCFF904XYE/@@download/ENCFF904XYE.bed.gz
https://www.encodeproject.org/files/ENCFF348NZA/@@download/ENCFF348NZA.bed.gz
https://www.encodeproject.org/files/ENCFF039SCX/@@download/ENCFF039SCX.bed.gz
https://www.encodeproject.org/files/ENCFF570IHV/@@download/ENCFF570IHV.bed.gz
https://www.encodeproject.org/files/ENCFF800XJG/@@download/ENCFF800XJG.bed.gz
https://www.encodeproject.org/files/ENCFF255IEF/@@download/ENCFF255IEF.bed.gz
https://www.encodeproject.org/files/ENCFF324DGQ/@@download/ENCFF324DGQ.bed.gz
https://www.encodeproject.org/files/ENCFF627HYQ/@@download/ENCFF627HYQ.bed.gz
https://www.encodeproject.org/files/ENCFF855RLW/@@download/ENCFF855RLW.bed.gz
https://www.encodeproject.org/files/ENCFF688RFE/@@download/ENCFF688RFE.bed.gz
https://www.encodeproject.org/files/ENCFF316MQA/@@download/ENCFF316MQA.bed.gz
https://www.encodeproject.org/files/ENCFF596DST/@@download/ENCFF596DST.bed.gz
https://www.encodeproject.org/files/ENCFF416FVT/@@download/ENCFF416FVT.bed.gz
https://www.encodeproject.org/files/ENCFF477HKC/@@download/ENCFF477HKC.bed.gz
https://www.encodeproject.org/files/ENCFF484XBS/@@download/ENCFF484XBS.bed.gz
https://www.encodeproject.org/files/ENCFF229AAU/@@download/ENCFF229AAU.bed.gz
https://www.encodeproject.org/files/ENCFF162ASN/@@download/ENCFF162ASN.bed.gz
https://www.encodeproject.org/files/ENCFF978IRB/@@download/ENCFF978IRB.bed.gz
https://www.encodeproject.org/files/ENCFF579BPA/@@download/ENCFF579BPA.bed.gz
https://www.encodeproject.org/files/ENCFF519OCH/@@download/ENCFF519OCH.bed.gz
https://www.encodeproject.org/files/ENCFF850IKH/@@download/ENCFF850IKH.bed.gz
https://www.encodeproject.org/files/ENCFF257XAQ/@@download/ENCFF257XAQ.bed.gz
https://www.encodeproject.org/files/ENCFF977VQE/@@download/ENCFF977VQE.bed.gz
https://www.encodeproject.org/files/ENCFF442HYL/@@download/ENCFF442HYL.bed.gz
https://www.encodeproject.org/files/ENCFF964BPN/@@download/ENCFF964BPN.bed.gz
https://www.encodeproject.org/files/ENCFF768JUC/@@download/ENCFF768JUC.bed.gz
https://www.encodeproject.org/files/ENCFF534TTO/@@download/ENCFF534TTO.bed.gz
https://www.encodeproject.org/files/ENCFF198ICP/@@download/ENCFF198ICP.bed.gz

Supplementary Table 1

https://www.encodeproject.org/files/ENCFF063POV/@@download/ENCFF063POV.bed.gz

https://www.encodeproject.org/files/ENCFF311MNY/@@download/ENCFF311MNY.bed.gz

https://www.encodeproject.org/files/ENCFF177APP/@@download/ENCFF177APP.bed.gz

https://www.encodeproject.org/files/ENCFF821ESA/@@download/ENCFF821ESA.bed.gz

https://www.encodeproject.org/files/ENCFF210QSG/@@download/ENCFF210QSG.bed.gz

https://www.encodeproject.org/files/ENCFF005TLD/@@download/ENCFF005TLD.bed.gz

https://www.encodeproject.org/files/ENCFF674CIO/@@download/ENCFF674CIO.bed.gz

https://www.encodeproject.org/files/ENCFF681HOL/@@download/ENCFF681HOL.bed.gz

https://www.encodeproject.org/files/ENCFF198WHL/@@download/ENCFF198WHL.bed.gz

https://www.encodeproject.org/files/ENCFF421LWX/@@download/ENCFF421LWX.bed.gz

Supplementary Table 1

FANTOM promoters and enhancers

http://fantom.gsc.riken.jp/5/datafiles/latest/extra/CAGE_peaks/hg19.cage_peak_phase1and2combined_tpm.osc.txt.gz

http://fantom.gsc.riken.jp/5/datafiles/latest/extra/Ontology/ff-phase2-170801.obo.txt

http://enhancer.binf.ku.dk/presets/facet_expressed_enhancers.tgz

Supplementary Table 1

miRWalk

http://mirwalk.umm.uni-heidelberg.de/download/hsa_miRWalk_3UTR.7z
