# Supplementary material for: Remus: A Web Application for Prioritization of Regulatory Regions and Variants in Monogenic Diseases
Source: Front Genet. 2021 Mar 5;12:638960. doi: 10.3389/fgene.2021.638960 (PMC7978111; doi:10.3389/fgene.2021.638960)
Supplement: Supplementary Figure 1 — Illustration of track collapsing and liftover. Original tracks for the same tissue (biological replicates) and in the same genome build were merged. Next, coordinates were lifted over to the other genome build (i.e., hg19 to hg38, and vice versa), and merging on the same tissues was performed again. [file Data_Sheet_1.zip › Supplementary Material/Data Sheet 5.pdf]

Supplementary Table 4

Repository	Regulator_type	Raw_tracks	Final_tracks	Tissues	Celltypes	NTR	Embryonic
fantom5	promoters	1829	145	53	92	0	0
fantom5	enhancers	112	112	41	71	0	0
encode	TFBS	608	55	32	23	0	0
encode	open chromatin	1072	147	73	63	11	0
screen	promoters	453	115	66	45	4	14
screen	enhancers		75	50	25	0	10
screen	open chromatin		142	98	42	2	44
