# Supplementary material for: Remus: A Web Application for Prioritization of Regulatory Regions and Variants in Monogenic Diseases
Source: Front Genet. 2021 Mar 5;12:638960. doi: 10.3389/fgene.2021.638960 (PMC7978111; doi:10.3389/fgene.2021.638960)
Supplement: Supplementary Figure 1 — Illustration of track collapsing and liftover. Original tracks for the same tissue (biological replicates) and in the same genome build were merged. Next, coordinates were lifted over to the other genome build (i.e., hg19 to hg38, and vice versa), and merging on the same tissues was performed again. [file Data_Sheet_1.zip › Supplementary Material/Table 1.pdf]

		Single tissue				Multiple tissues				Unselected		
		**Count**	**Size [kb]**	**Specificity [%]**	**Sensitivity**	**Count**	**Size [kb]**	**Specificity [%]**	**Identified**	**Count**	**Size [kb]**	**Identified**
Distal regulators	Min	1	0.1	63.4	18/26 (69.2%)	33	17.7	56.6	20/26 (76.9%)	645	370.7	23/26 (88.5%)
	Mean	135	48.2	91.6		334	105.6	82.1		1237	554.7	
	Median	97	42.7	93.3		272	93.1	80.9		1268	561.6	
	Max	909	175.7	100.0		1010	228.7	96.7		1676	801.8	
Intragenic regulators	Min	2	0.4	75.7	15/21 (71.4%)	5	3.2	56.9	19/21 (90.5%)	645	370.7	20/21 (95.2%)
	Mean	156	64.7	89.9		374	143.1	77.2		1237	554.7	
	Median	88	40.6	93.8		406	148.2	75.9		1268	561.6	
	Max	796	170.3	99.9		874	296.5	99.5		1676	801.8	
Promoters	Min	2	0.7	63.4	30/31 (96.8%)	26	17.4	56.6	31/31 (100%)	748	373.6	31/31 (100%)
	Mean	138	56.9	89.8		269	99.8	82.8		1267	554.6	
	Median	98	47.0	91.6		210	92.8	86.8		1268	534.7	
	Max	909	175.7	99.9		1010	240.9	97.9		1676	826.3	
**Overall**	Median	98	44.2	**92.73**	**63/78 (80.7%)**	254	98.4	**82.49**	**70/78 (89.7%)**	1265	561.6	**74/78 (94.9%)**
