# Supplementary figures and images for: Remus: A Web Application for Prioritization of Regulatory Regions and Variants in Monogenic Diseases
Source: Front Genet. 2021 Mar 5;12:638960. doi: 10.3389/fgene.2021.638960 (PMC7978111; doi:10.3389/fgene.2021.638960)

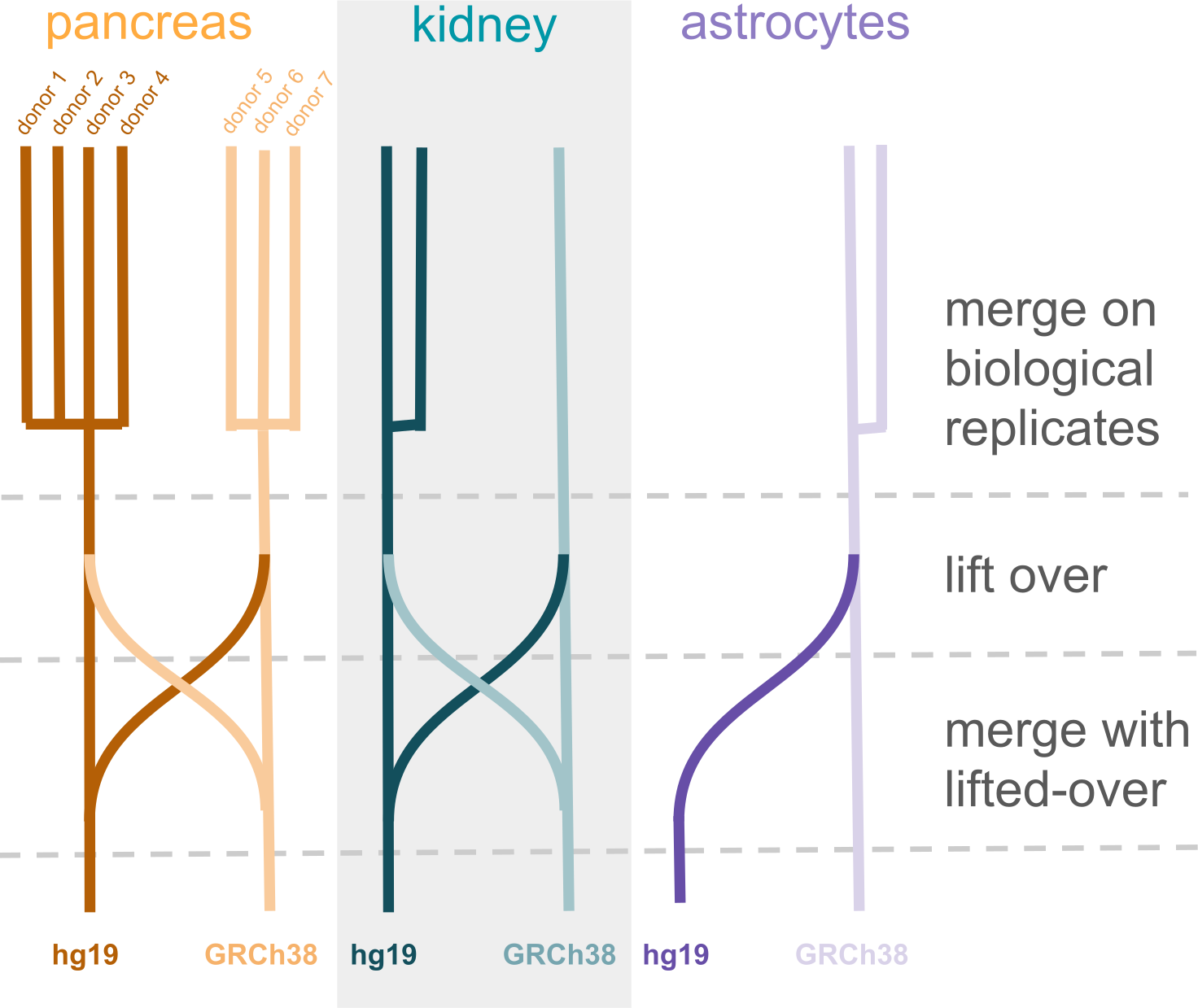

Supplement: Supplementary Figure 1 — Illustration of track collapsing and liftover. Original tracks for the same tissue (biological replicates) and in the same genome build were merged. Next, coordinates were lifted over to the other genome build (i.e., hg19 to hg38, and vice versa), and merging on the same tissues was performed again. [file Data_Sheet_1.zip › Supplementary Material/Image 1.tif]

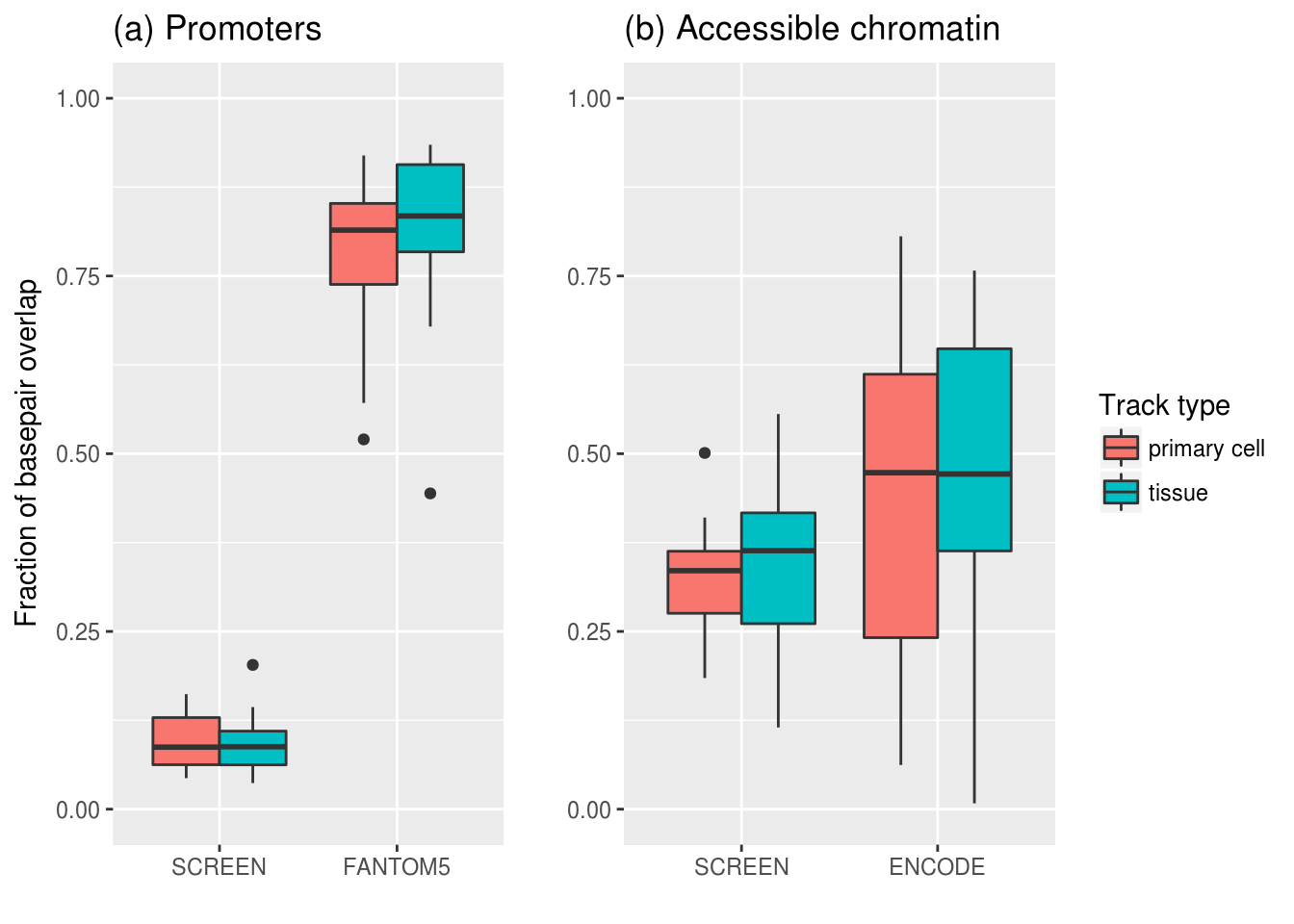

Supplement: Supplementary Figure 1 — Illustration of track collapsing and liftover. Original tracks for the same tissue (biological replicates) and in the same genome build were merged. Next, coordinates were lifted over to the other genome build (i.e., hg19 to hg38, and vice versa), and merging on the same tissues was performed again. [file Data_Sheet_1.zip › Supplementary Material/Image 2.tif]

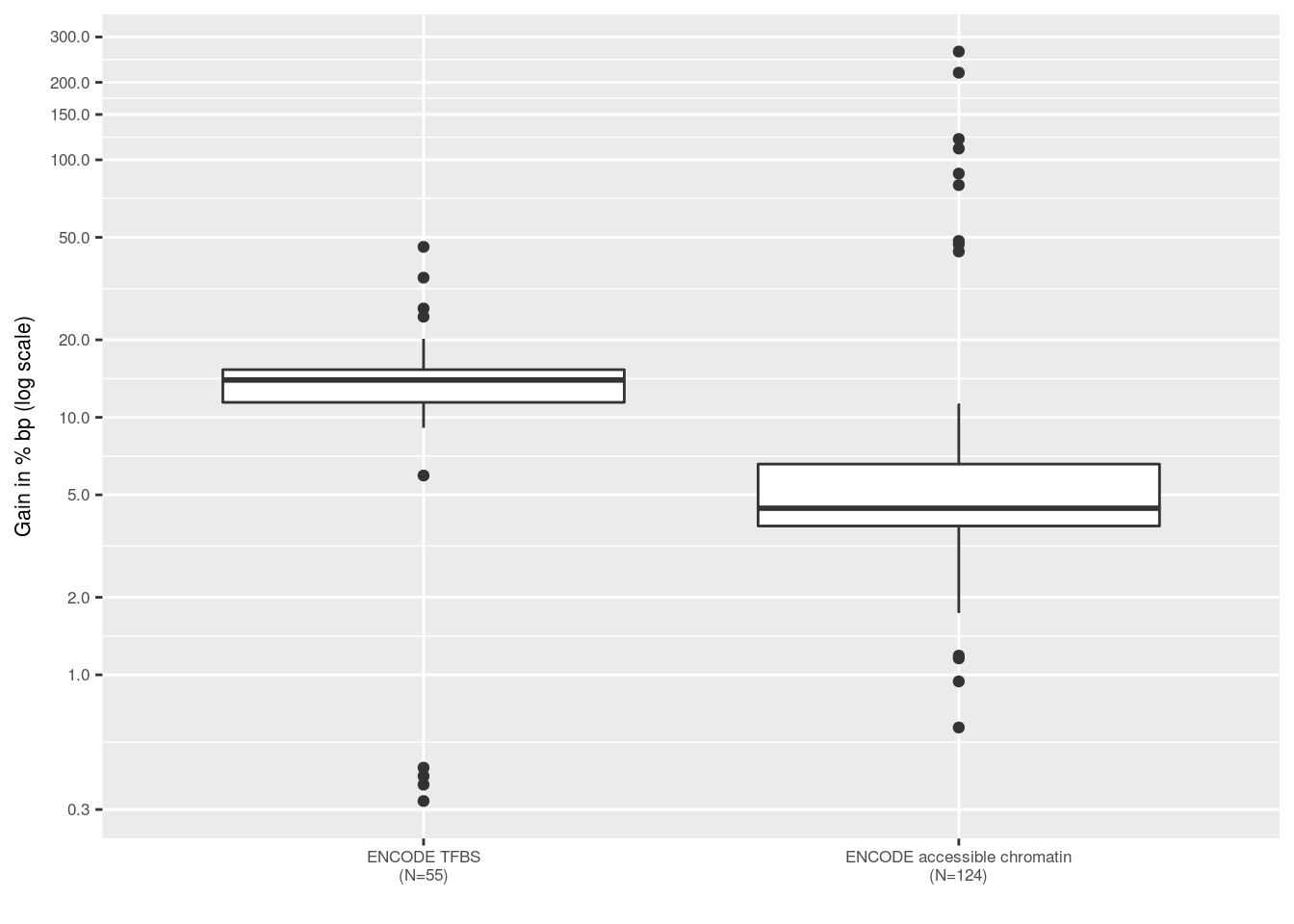

Supplement: Supplementary Figure 1 — Illustration of track collapsing and liftover. Original tracks for the same tissue (biological replicates) and in the same genome build were merged. Next, coordinates were lifted over to the other genome build (i.e., hg19 to hg38, and vice versa), and merging on the same tissues was performed again. [file Data_Sheet_1.zip › Supplementary Material/Image 3.tif]

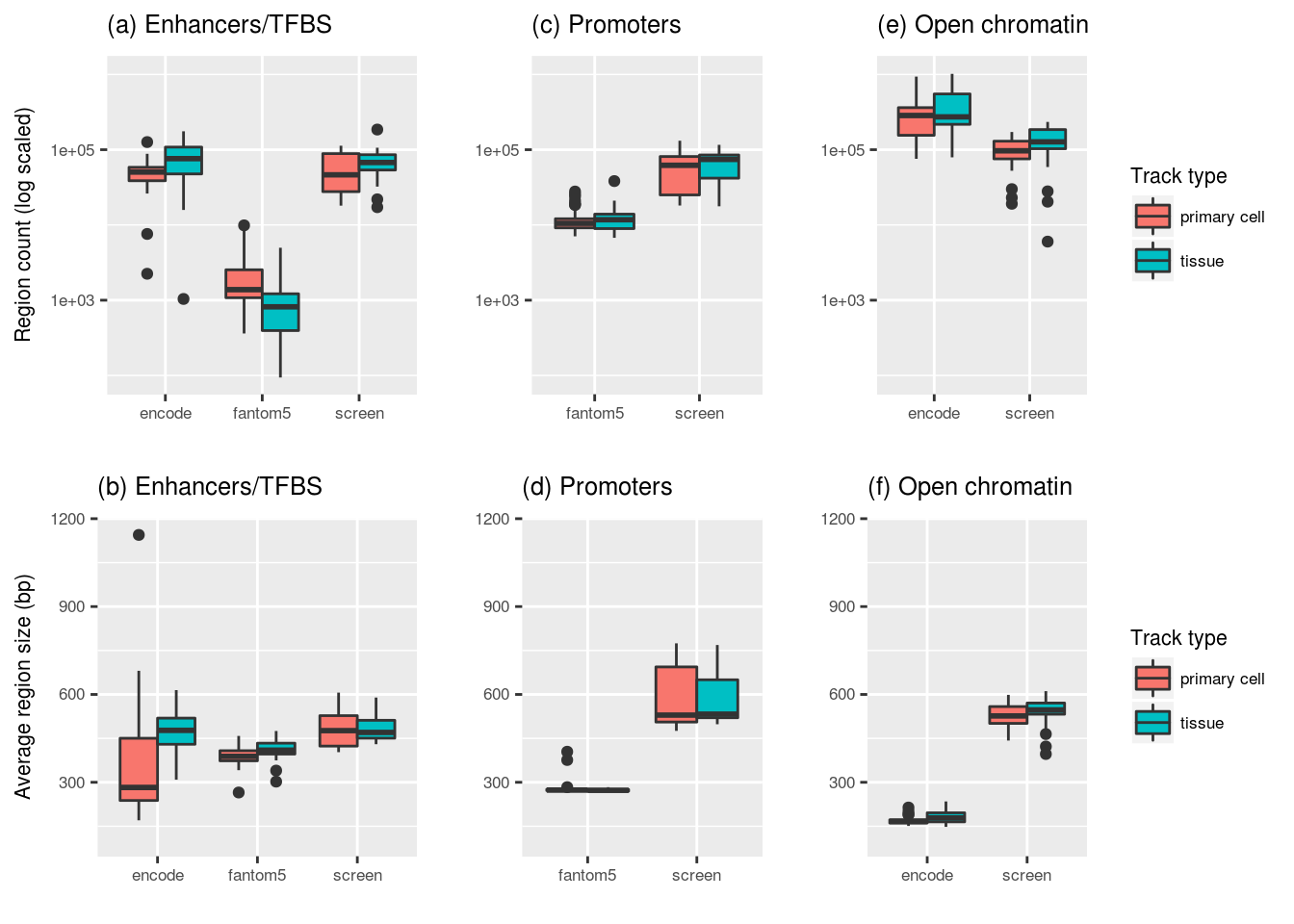

Supplement: Supplementary Figure 1 — Illustration of track collapsing and liftover. Original tracks for the same tissue (biological replicates) and in the same genome build were merged. Next, coordinates were lifted over to the other genome build (i.e., hg19 to hg38, and vice versa), and merging on the same tissues was performed again. [file Data_Sheet_1.zip › Supplementary Material/Image 4.tif]
