# Supplementary material for: Remus: A Web Application for Prioritization of Regulatory Regions and Variants in Monogenic Diseases
Source: Front Genet. 2021 Mar 5;12:638960. doi: 10.3389/fgene.2021.638960 (PMC7978111; doi:10.3389/fgene.2021.638960)
Supplement: Supplementary Figure 1 — Illustration of track collapsing and liftover. Original tracks for the same tissue (biological replicates) and in the same genome build were merged. Next, coordinates were lifted over to the other genome build (i.e., hg19 to hg38, and vice versa), and merging on the same tissues was performed again. [file Data Sheet 4.pdf › Supplementary Material/Data Sheet 4.pdf]

Distal regulators

chr	pos	gene	tissues	num_features	features_size	specificity	regulator_overlapping	regulator_closest	regulator_closest_distance	tissues_WIDE	num_features_WIDE	features_size_WIDE	specifcity	regulator_overlapping_WIDE	regulator_closest_WIDE	regulator_closest_distance_WIDE	num_features_ALL	features_size_ALL	regulator_overlapping_ALL	regulator_closest_ALL	regulator_closest_distance_ALL
chr11	17498513	ABCC8	pancreas (CHR_E, CHR_S, ENH_F, ENH_S, PR_F, PR_S)	71	31105	94.9150908	None	chr11:17526110-17526630	27598	pancreas (CHR_E, CHR_S, ENH_F, ENH_S	510	134031	78.0891988387	chr11:17497325-17498557	chr11:17497325-17498557	0	1286	611712	chr11:17496948-17499370	chr1:209987780-209990814	0
chr19	39137810	ACTN4	glomerular endothelial cell (CHR_E, CHR_S, PR_F)	69	39158	94.3855635	None	chr19:39137874-39138532	65	left renal pelvis (CHR_E, CHR_S);right ren	487	211662	69.6521050911	None	chr19:39137874-39138532	65	1040	697452	chr19:39137503-39151112	chr7:156061249-156061649	0
chr11	67250359	AIP	brain (CHR_E, CHR_S, ENH_F, PR_F, PR_S)	227	79443	87.3820888	chr11:67250103-67251115	chr11:67250103-67251115	0	astrocyte of the hippocampus (CHR_E);bra	619	193043	69.3390300268	chr11:67250103-67251115	chr11:67250103-67251115	0	1044	629605	chr11:67249821-67253212	chr8:11329995-11335497	0
chrX	55057617	ALAS2	reticulocyte (ENH_F, PR_F)	2	409	99.9180909	None	chrX:55057403-55057616	1	erythroblast (ENH_E);stromal cell of bone	38	17665	96.4622877673	None	chrX:55054339-55054930	2687	1358	499334	None	chr10:23508765-23508915	461
chr9	104198194	ALDOB	liver (CHR_E, ENH_E, ENH_F, ENH_S, PR_F, PR_S)	125	86675	79.4465325	chr9:104197797-104198580	chr9:104197797-104198580	0	hepatocyte (CHR_E, ENH_F, PR_F);right lo	137	93084	77.9267497421	chr9:104197778-104198580	chr9:104197778-104198580	0	1156	421705	chr9:104196495-104198580	chr10:23508765-23508915	403
chr8	11331747	BLK	pancreas (CHR_E, CHR_S, ENH_F, ENH_S, PR_F, PR_S)	120	54219	92.4130394	chr8:11331735-11332050	chr8:11331735-11332050	0	pancreas (CHR_E, CHR_S, ENH_F, ENH_S	743	196897	72.4478544262	chr8:11331324-11332406	chr8:11331324-11332406	0	1061	714634	chr8:11329995-11335497	chr10:23508765-23508915	401
chrX	135730217	CD40LG	naive thymus-derived CD4-positive, alpha-beta T cell (CHR_E, CHR_S, PR_F)	100	33192	93.7622155	chrX:135730097-135730424	chrX:135730097-135730424	0	CD4-positive, CD25-positive, alpha-beta re	214	80901	84.7962459031	chrX:135730097-135730424	chrX:135730097-135730424	0	1503	532112	chrX:135729962-135730864	chr10:23508765-23508915	329
chr7	117119337	CFTR	lung (CHR_E, CHR_S, ENH_F, ENH_S, PR_F, PR_S)	212	75727	85.1185669	chr7:117118531-117120609	chr7:117118531-117120609	0	lung (CHR_E, CHR_S, ENH_F, ENH_S, PR	332	118465	76.7199416746	chr7:117118251-117120609	chr7:117118251-117120609	0	1269	508869	chr7:117117934-117120646	chr10:23508765-23508915	320
chr3	48632780	COL7A1	skin fibroblast (CHR_S, ENH_F, PR_F)	80	49134	92.5370913	chr3:48632646-48632855	chr3:48632646-48632855	0	fibroblast of skin of back (CHR_E);fibrobl	422	147550	77.5887942452	chr3:48632646-48633622	chr3:48632646-48633622	0	1293	658376	chr3:48631523-48639515	chr11:5271374-5272287	92
chrX	146993366	FMR1	astrocyte (CHR_E, CHR_S, ENH_E, ENH_F, ENH_S, PR_F, PR_S)	909	175693	63.4299761	chrX:146993140-146993563	chrX:146993140-146993563	0	astrocyte of the hippocampus (CHR_E);bra	1010	208450	56.6116949643	chrX:146993140-146994427	chrX:146993140-146994427	0	1676	480429	chrX:146991935-146996054	chr11:5276008-5276656	0
chr19	49468350	FTL	erythroblast (ENH_E)	35	23652	96.5037694	chr19:49467797-49468757	chr19:49467797-49468757	0	granulocyte (ENH_F, PR_F);peripheral bloo	64	31999	95.2699186992	chr19:49467797-49468821	chr19:49467797-49468821	0	1082	676500	chr19:49463885-49474865	chr11:31685540-31686265	0
chr16	209709	HBA2	erythroblast (ENH_E)	29	19285	96.0463328	None	chr16:215748-216452	6040	erythroblast (ENH_E);stromal cell of bone	90	51113	89.5211931731	None	chr16:215748-216452	6040	645	487775	chr16:209142-210247	chr11:67249821-67253212	0
chr11	5271283	HBG1	blood (ENH_F, PR_F)	1	107	99.9711337	None	chr11:5297176-5297283	25894	granulocyte (ENH_F, PR_F);peripheral bloo	33	21084	94.3119830363	None	chr11:5268273-5269072	2211	1258	370674	None	chr12:114704302-114704713	0
chr11	5271201	HBG1	blood (ENH_F, PR_F)	1	107	99.9711337	None	chr11:5297176-5297283	25976	granulocyte (ENH_F, PR_F);peripheral bloo	33	21084	94.3119830363	None	chr11:5268273-5269072	2129	1258	370674	None	chr16:209142-210247	0
chr11	5271204	HBG1	blood (ENH_F, PR_F)	1	107	99.9711337	None	chr11:5297176-5297283	25973	granulocyte (ENH_F, PR_F);peripheral bloo	33	21084	94.3119830363	None	chr11:5268273-5269072	2132	1258	370674	None	chrX:105251550-105252509	0
chr11	5276125	HBG2	reticulocyte (ENH_F, PR_F)	5	1065	99.7134987	chr11:5276008-5276213	chr11:5276008-5276213	0	granulocyte (ENH_F, PR_F);reticulocyte (E	35	21452	94.2290827115	chr11:5276008-5276213	chr11:5276008-5276213	0	1265	371726	chr11:5276008-5276656	chr1:171620893-171622713	0
chr11	5276186	HBG2	reticulocyte (ENH_F, PR_F)	5	1065	99.7134987	chr11:5276008-5276213	chr11:5276008-5276213	0	granulocyte (ENH_F, PR_F);reticulocyte (E	35	21452	94.2290827115	chr11:5276008-5276213	chr11:5276008-5276213	0	1265	371726	chr11:5276008-5276656	chr3:48631523-48639515	0
chr11	5276213	HBG2	reticulocyte (ENH_F, PR_F)	5	1065	99.7134987	chr11:5276008-5276213	chr11:5276008-5276213	0	granulocyte (ENH_F, PR_F);reticulocyte (E	35	21452	94.2290827115	chr11:5276008-5276213	chr11:5276008-5276213	0	1265	371726	chr11:5276008-5276656	chr7:117117934-117120646	0
chrX	153237261	HCFC1	astrocyte (CHR_E, CHR_S, ENH_E, ENH_F, ENH_S, PR_F, PR_S)	359	77024	88.6654737	chrX:153235486-153239031	chrX:153235486-153239031	0	astrocyte of the hippocampus (CHR_E);bra	528	164976	75.722829158	chrX:153235486-153239582	chrX:153235486-153239582	0	991	679552	chrX:153231593-15324067(	chr9:104196495-104198580	0
chr20	42984264	HNF4A	pancreas (CHR_E, CHR_S, ENH_F, ENH_S, PR_F, PR_S)	86	44222	93.8989243	chr20:42983201-42985298	chr20:42983201-42985298	0	pancreas (CHR_E, CHR_S, ENH_F, ENH_S	611	151384	79.1143492963	chr20:42983201-42985298	chr20:42983201-42985298	0	1289	724823	chr20:42982955-42987085	chr11:2182191-2182675	0
chr20	42984276	HNF4A	pancreas (CHR_E, CHR_S, ENH_F, ENH_S, PR_F, PR_S)	86	44222	93.8989243	chr20:42983201-42985298	chr20:42983201-42985298	0	pancreas (CHR_E, CHR_S, ENH_F, ENH_S	611	151384	79.1143492963	chr20:42983201-42985298	chr20:42983201-42985298	0	1289	724823	chr20:42982955-42987085	chr11:5270623-5271096	105
chr20	42984299	HNF4A	pancreas (CHR_E, CHR_S, ENH_F, ENH_S, PR_F, PR_S)	86	44222	93.8989243	chr20:42983201-42985298	chr20:42983201-42985298	0	pancreas (CHR_E, CHR_S, ENH_F, ENH_S	611	151384	79.1143492963	chr20:42983201-42985298	chr20:42983201-42985298	0	1289	724823	chr20:42982955-42987085	chr11:5270623-5271096	108
chr20	42984309	HNF4A	pancreas (CHR_E, CHR_S, ENH_F, ENH_S, PR_F, PR_S)	86	44222	93.8989243	chr20:42983201-42985298	chr20:42983201-42985298	0	pancreas (CHR_E, CHR_S, ENH_F, ENH_S	611	151384	79.1143492963	chr20:42983201-42985298	chr20:42983201-42985298	0	1289	724823	chr20:42982955-42987085	chr11:5276008-5276656	0
chr11	2182543	INS	pancreas (CHR_E, CHR_S, ENH_F, ENH_S, PR_F, PR_S)	129	67156	91.624481	None	chr11:2178120-2178439	4104	pancreas (CHR_E, CHR_S, ENH_F, ENH_S	775	228706	71.4763916275	chr11:2182191-2182675	chr11:2182191-2182675	0	756	801813	chr11:2182191-2182675	chr11:5276008-5276656	0
chr1	209989478	IRF6	kidney (CHR_E, CHR_S, ENH_F, PR_F, PR_S)	242	86217	85.5697728	chr1:209989455-209989630	chr1:209989455-209989630	0	left renal pelvis (CHR_E, CHR_S);right ren	464	158252	73.5132013892	chr1:209989455-209989630	chr1:209989455-209989630	0	1286	597475	chr1:209987780-209990814	chr11:17496948-17499370	0
chr1	171621877	MYOC	eye (CHR_E, CHR_S, ENH_F, PR_F)	157	45526	90.2790352	chr1:171621815-171622035	chr1:171621815-171622035	0	retina (CHR_E, CHR_S, PR_F);retina embry	186	57190	87.788473036	chr1:171621815-171622035	chr1:171621815-171622035	0	1259	468328	chr1:171620893-171622713	chr17:42078653-42080742	0
chr17	42078968	NAGS	liver (CHR_E, ENH_E, ENH_F, ENH_S, PR_F, PR_S)	228	100469	85.3471749	chr17:42078653-42079613	chr17:42078653-42079613	0	small intestine (CHR_E, CHR_S, ENH_F, E	450	189822	72.3155544342	chr17:42078653-42079613	chr17:42078653-42079613	0	1143	685663	chr17:42078653-42080742	chr19:39137503-39151112	0
chrX	38211584	OTC	liver (CHR_E, ENH_E, ENH_F, ENH_S, PR_F, PR_S)	100	51540	90.7696606	chrX:38211503-38212292	chrX:38211503-38212292	0	hepatocyte (CHR_E, ENH_F, PR_F);right lo	149	60889	89.095340774	chrX:38211503-38212292	chrX:38211503-38212292	0	1524	558376	chrX:38211503-38212302	chr19:49463885-49474865	0
chr11	31685945	PAX6	retina (CHR_E, CHR_S, PR_F)	234	103857	79.9383026	chr11:31685540-31686265	chr11:31685540-31686265	0	retina (CHR_E, CHR_S, PR_F);eye (CHR_E	352	139786	72.9980219746	chr11:31685540-31686265	chr11:31685540-31686265	0	1316	517688	chr11:31685540-31686265	chr20:42982955-42987085	0
chr10	23508305	PTF1A	pancreas (CHR_E, CHR_S, ENH_F, ENH_S, PR_F, PR_S)	99	31651	93.2946064	None	chr10:23502854-23502866	5439	pancreas (CHR_E, CHR_S, ENH_F, ENH_S	276	82662	82.4877177595	None	chr10:23502854-23502866	5439	1233	472023	None	chr20:42982955-42987085	0
chr10	23508363	PTF1A	pancreas (CHR_E, CHR_S, ENH_F, ENH_S, PR_F, PR_S)	99	31651	93.2946064	None	chr10:23502854-23502866	5497	pancreas (CHR_E, CHR_S, ENH_F, ENH_S	276	82662	82.4877177595	None	chr10:23502854-23502866	5497	1233	472023	None	chr20:42982955-42987085	0
chr10	23508365	PTF1A	pancreas (CHR_E, CHR_S, ENH_F, ENH_S, PR_F, PR_S)	99	31651	93.2946064	None	chr10:23502854-23502866	5499	pancreas (CHR_E, CHR_S, ENH_F, ENH_S	276	82662	82.4877177595	None	chr10:23502854-23502866	5499	1233	472023	None	chr20:42982955-42987085	0
chr10	23508437	PTF1A	pancreas (CHR_E, CHR_S, ENH_F, ENH_S, PR_F, PR_S)	99	31651	93.2946064	None	chr10:23502854-23502866	5571	pancreas (CHR_E, CHR_S, ENH_F, ENH_S	276	82662	82.4877177595	None	chr10:23502854-23502866	5571	1233	472023	None	chrX:38211503-38212302	0
chr10	23508446	PTF1A	pancreas (CHR_E, CHR_S, ENH_F, ENH_S, PR_F, PR_S)	99	31651	93.2946064	None	chr10:23502854-23502866	5580	pancreas (CHR_E, CHR_S, ENH_F, ENH_S	276	82662	82.4877177595	None	chr10:23502854-23502866	5580	1233	472023	None	chrX:55057235-55057616	1
chrX	105251968	SERPINA7	liver (CHR_E, ENH_E, ENH_F, ENH_S, PR_F, PR_S)	33	14866	97.2302596	chrX:105251614-105252493	chrX:105251614-105252493	0	hepatocyte (CHR_E, ENH_F, PR_F);right lo	46	17775	96.6882728528	chrX:105251614-105252493	chrX:105251614-105252493	0	1637	536729	chrX:105251550-10525250(	chrX:135729962-135730864	0
chr7	156061506	SHH	brain (CHR_E, CHR_S, ENH_F, PR_F, PR_S)	342	118549	83.8245367	chr7:156061389-156061599	chr7:156061389-156061599	0	astrocyte of the hippocampus (CHR_E);bra	400	140001	80.8975104176	chr7:156061389-156061599	chr7:156061389-156061599	0	1168	732894	chr7:156061249-156061649	chrX:146991935-146996054	0
chr12	114704515	TBX5	heart (CHR_E, CHR_S, ENH_F, PR_F, PR_S)	317	110840	79.5236345	None	chr12:114702925-114703075	1440	heart right ventricle (CHR_E, ENH_S, PR_	332	119772	77.8735541938	None	chr12:114702915-114703075	1440	1382	541307	chr12:114704302-11470471	chrX:153231593-153240670	0

Intragenic

chr	pos	gene	tissues	num_features	features_size	specificity	regulator_overlapping	regulator_closest	regulator_closest_distance	tissues_WIDE	num_features_WIDE	features_size_WIDE	specificity	regulator_overlapping_WIDE	regulator_closest_WIDE	regulator_closest_distance_WIDE	num_features_ALL	features_size_ALL	regulator_overlapping_ALL	regulator_closest_ALL	regulator_closest_distance_ALL
chrX	55054635	ALAS2	reticulocyte (ENH_F, PR_F)	2	409	99.9180909	None	chrX:55057403-55057616	2769	erythroblast (ENH_E);reticulocyte (ENH_F,	40	18074	96.3803786644	chrX:55054339-55054930	chrX:55054339-55054930	0	1358	499334	chrX:55054339-55054930	chrX:55054339-55054930	0
chr1	21890663	ALPL	osteoblast (CHR_E, ENH_F, ENH_S, PR_F, PR_S)	796	170267	75.6837504	None	chr1:21890408-21890565	98	right lobe of liver (CHR_E, CHR_S, ENH_E	874	296548	57.6492497347	chr1:21890408-21890800	chr1:21890408-21890800	0	1106	700219	chr1:21888788-21892766	chr1:21888788-21892766	0
chr8	41655260	ANK1	erythroblast (ENH_E)	26	17430	97.8906579	chr8:41654850-41655526	chr8:41654850-41655526	0	erythroblast (ENH_E)	26	17430	97.8906579017	chr8:41654850-41655526	chr8:41654850-41655526	0	1587	826324	chr8:41653034-41657665	chr8:41653034-41657665	0
chr5	112073008	APC	colonic mucosa (ENH_S, PR_S)	20	7482	98.6511142	chr5:112072865-112074555	chr5:112072865-112074555	0	transverse colon (CHR_E, CHR_S, ENH_E)	149	55853	89.9305906108	chr5:112072865-112074555	chr5:112072865-112074555	0	1284	554680	chr5:112072783-112074582	chr5:112072783-112074582	0
chr2	219524871	BCS1L	skin fibroblast (CHR_S, ENH_F, PR_F)	88	40576	93.7812367	chr2:219523741-219524926	chr2:219523741-219524926	0	skin fibroblast (CHR_S, ENH_F, PR_F);ske	303	125545	80.7587087361	chr2:219523741-219524926	chr2:219523741-219524926	0	1205	652477	chr2:219523139-219525593	chr2:219523139-219525593	0
chrX	100641044	BTK	lymphocyte of B lineage (ENH_F, PR_F)	2	489	99.8953326	None	chrX:100641155-100641388	112	common myeloid progenitor, CD34-positive	92	34328	92.6523028977	chrX:100640812-100641481	chrX:100640812-100641481	0	1381	467194	chrX:100640802-100641793	chrX:100640802-100641793	0
chr13	60738072	DIAPH3	peripheral blood mononuclear cell (ENH_S, PR_S)	11	4443	99.055577	chr13:60737772-60738435	chr13:60737772-60738435	0	peripheral blood mononuclear cell (ENH_S,	11	4443	99.0555770482	chr13:60737772-60738435	chr13:60737772-60738435	0	1494	470446	chr13:60735915-60740519	chr13:60735915-60740519	0
chr13	113760124	F7	liver (CHR_E, ENH_E, ENH_F, ENH_S, PR_F, PR_S)	254	99710	87.5431791	chr13:113759817-113760385	chr13:113759817-113760385	0	right lobe of liver (CHR_E, CHR_S, ENH_	515	156425	80.4577453791	chr13:113759817-113760680	chr13:113759817-113760680	0	1062	800445	chr13:113759485-11376068	chr13:113759485-113760680	0
chr8	11560787	GATA4	heart (CHR_E, CHR_S, ENH_F, PR_F, PR_S)	331	148637	77.6459831	chr8:11560264-11561199	chr8:11560264-11561199	0	cardiac muscle cell (CHR_E, ENH_E, ENH_	642	231962	65.1144568619	chr8:11560264-11561199	chr8:11560264-11561199	0	1001	664923	chr8:11557483-11568038	chr8:11557483-11568038	0
chr8	11560864	GATA4	heart (CHR_E, CHR_S, ENH_F, PR_F, PR_S)	331	148637	77.6459831	chr8:11560264-11561199	chr8:11560264-11561199	0	cardiac muscle cell (CHR_E, ENH_E, ENH_	642	231962	65.1144568619	chr8:11560264-11561199	chr8:11560264-11561199	0	1001	664923	chr8:11557483-11568038	chr8:11557483-11568038	0
chr8	11561283	GATA4	heart (CHR_E, CHR_S, ENH_F, PR_F, PR_S)	331	148637	77.6459831	chr8:11561243-11561760	chr8:11561243-11561760	0	cardiac muscle cell (CHR_E, ENH_E, ENH_	642	231962	65.1144568619	chr8:11561243-11562285	chr8:11561243-11562285	0	1001	664923	chr8:11557483-11568038	chr8:11557483-11568038	0
chr8	11561369	GATA4	heart (CHR_E, CHR_S, ENH_F, PR_F, PR_S)	331	148637	77.6459831	chr8:11561243-11561760	chr8:11561243-11561760	0	cardiac muscle cell (CHR_E, ENH_E, ENH_	642	231962	65.1144568619	chr8:11561243-11562285	chr8:11561243-11562285	0	1001	664923	chr8:11557483-11568038	chr8:11557483-11568038	0
chr8	11561399	GATA4	heart (CHR_E, CHR_S, ENH_F, PR_F, PR_S)	331	148637	77.6459831	chr8:11561243-11561760	chr8:11561243-11561760	0	cardiac muscle cell (CHR_E, ENH_E, ENH_	642	231962	65.1144568619	chr8:11561243-11562285	chr8:11561243-11562285	0	1001	664923	chr8:11557483-11568038	chr8:11557483-11568038	0
chrX	70443099	GJB1	liver (CHR_E, ENH_E, ENH_F, ENH_S, PR_F, PR_S)	97	47047	89.5328762	chrX:70442401-70443516	chrX:70442401-70443516	0	right lobe of liver (CHR_E, CHR_S, ENH_	109	51240	88.6000080094	chrX:70442401-70443516	chrX:70442401-70443516	0	1321	449474	chrX:70442395-70443543	chrX:70442395-70443543	0
chrX	70443185	GJB1	liver (CHR_E, ENH_E, ENH_F, ENH_S, PR_F, PR_S)	97	47047	89.5328762	chrX:70442401-70443516	chrX:70442401-70443516	0	right lobe of liver (CHR_E, CHR_S, ENH_	109	51240	88.6000080094	chrX:70442401-70443516	chrX:70442401-70443516	0	1321	449474	chrX:70442395-70443543	chrX:70442395-70443543	0
chr1	228337561	GJC2	brain (CHR_E, CHR_S, ENH_F, PR_F, PR_S)	185	67411	90.4298076	chr1:228337423-228337662	chr1:228337423-228337662	0	neuron (ENH_F, PR_F);dendritic cell (ENH	185	67411	90.4298075626	chr1:228337423-228337662	chr1:228337423-228337662	0	946	704385	chr1:228326635-228340306	chr1:228326635-228340306	0
chr12	121416444	HNF1A	hepatocyte (CHR_E, ENH_F, PR_F)	58	9540	98.4531996	None	chr12:121416140-121416360	84	right lobe of liver (CHR_E, CHR_S, ENH_	567	221315	64.1163375527	chr12:121415882-121416924	chr12:121415882-121416924	0	1328	616757	chr12:121415442-12141989	chr12:121415442-121419892	0
chr12	121416446	HNF1A	hepatocyte (CHR_E, ENH_F, PR_F)	58	9540	98.4531996	None	chr12:121416140-121416360	86	right lobe of liver (CHR_E, CHR_S, ENH_	567	221315	64.1163375527	chr12:121415882-121416924	chr12:121415882-121416924	0	1328	616757	chr12:121415442-12141989	chr12:121415442-121419892	0
chr12	121416448	HNF1A	hepatocyte (CHR_E, ENH_F, PR_F)	58	9540	98.4531996	None	chr12:121416140-121416360	88	right lobe of liver (CHR_E, CHR_S, ENH_	567	221315	64.1163375527	chr12:121415882-121416924	chr12:121415882-121416924	0	1328	616757	chr12:121415442-12141989	chr12:121415442-121419892	0
chr11	17409772	KCNJ11	pancreas (CHR_E, CHR_S, ENH_F, ENH_S, PR_F, PR_S)	76	34342	94.5652363	None	chr11:17410321-17411422	550	neuron (ENH_F, PR_F);pancreas (CHR_E,	701	197079	68.8114322791	None	chr11:17409382-17409532	240	1293	631895	chr11:17403352-17411966	chr11:17403352-17411966	0
chr19	11200073	LDLR	liver (CHR_E, ENH_E, ENH_F, ENH_S, PR_F, PR_S)	301	155778	77.0129427	chr19:11199078-11202067	chr19:11199078-11202067	0	right lobe of liver (CHR_E, CHR_S, ENH_	753	292152	56.8891964756	chr19:11199078-11202067	chr19:11199078-11202067	0	983	677677	chr19:11198397-11206744	chr19:11198397-11206744	0
chr19	11200087	LDLR	liver (CHR_E, ENH_E, ENH_F, ENH_S, PR_F, PR_S)	301	155778	77.0129427	chr19:11199078-11202067	chr19:11199078-11202067	0	right lobe of liver (CHR_E, CHR_S, ENH_	753	292152	56.8891964756	chr19:11199078-11202067	chr19:11199078-11202067	0	983	677677	chr19:11198397-11206744	chr19:11198397-11206744	0
chr19	11200089	LDLR	liver (CHR_E, ENH_E, ENH_F, ENH_S, PR_F, PR_S)	301	155778	77.0129427	chr19:11199078-11202067	chr19:11199078-11202067	0	right lobe of liver (CHR_E, CHR_S, ENH_	753	292152	56.8891964756	chr19:11199078-11202067	chr19:11199078-11202067	0	983	677677	chr19:11198397-11206744	chr19:11198397-11206744	0
chr1	8021919	PARK7	brain (CHR_E, CHR_S, ENH_F, PR_F, PR_S)	307	117217	82.5771133	chr1:8021145-8022347	chr1:8021145-8022347	0	right lobe of liver (CHR_E, CHR_S, ENH_	615	287463	57.2721083986	chr1:8021145-8022741	chr1:8021145-8022741	0	1274	672776	chr1:8020254-8023005	chr1:8020254-8023005	0
chr3	12421189	PPARG	adipose tissue (ENH_F, PR_F)	4	1203	99.8007084	None	chr3:12392709-12393047	28142	adipose tissue (ENH_F, PR_F);subcutaneou	382	145212	75.9438603931	None	chr3:12421469-12421877	281	1323	603638	None	chr3:12421214-12421395	26
chr10	89623365	PTEN	breast epithelium (CHR_E, ENH_E, PR_S)	85	27281	94.5661873	chr10:89621301-89623874	chr10:89621301-89623874	0	sigmoid colon (CHR_S, ENH_E, ENH_S, PF	406	148203	70.481018205	chr10:89621247-89623874	chr10:89621247-89623874	0	1200	502060	chr10:89619737-89629882	chr10:89619737-89629882	0
chr10	89623373	PTEN	breast epithelium (CHR_E, ENH_E, PR_S)	85	27281	94.5661873	chr10:89621301-89623874	chr10:89621301-89623874	0	sigmoid colon (CHR_S, ENH_E, ENH_S, PF	406	148203	70.481018205	chr10:89621247-89623874	chr10:89621247-89623874	0	1200	502060	chr10:89619737-89629882	chr10:89619737-89629882	0
chr10	89623462	PTEN	breast epithelium (CHR_E, ENH_E, PR_S)	85	27281	94.5661873	chr10:89621301-89623874	chr10:89621301-89623874	0	sigmoid colon (CHR_S, ENH_E, ENH_S, PF	406	148203	70.481018205	chr10:89621247-89623874	chr10:89621247-89623874	0	1200	502060	chr10:89619737-89629882	chr10:89619737-89629882	0
chr9	35657917	RMRP	liver (CHR_E, ENH_E, ENH_F, ENH_S, PR_F, PR_S)	182	107196	82.8425182	chr9:35657224-35659234	chr9:35657224-35659234	0	hepatocyte (CHR_E, ENH_F, PR_F);liver (	235	140733	77.4746829669	chr9:35657224-35659234	chr9:35657224-35659234	0	1194	624777	chr9:35655512-35659967	chr9:35655512-35659967	0
chr9	35657945	RMRP	liver (CHR_E, ENH_E, ENH_F, ENH_S, PR_F, PR_S)	182	107196	82.8425182	chr9:35657224-35659234	chr9:35657224-35659234	0	hepatocyte (CHR_E, ENH_F, PR_F);liver (	235	140733	77.4746829669	chr9:35657224-35659234	chr9:35657224-35659234	0	1194	624777	chr9:35655512-35659967	chr9:35655512-35659967	0
chrY	2655719	SRY	testis (CHR_E, CHR_S, ENH_E, ENH_F, PR_F)	10	6889	95.4841924	chrY:2654199-2656065	chrY:2654199-2656065	0	testis (CHR_E, CHR_S, ENH_E, ENH_F, PF	10	6889	95.484192379	chrY:2654199-2656065	chrY:2654199-2656065	0	577	152553	chrY:2654164-2656716	chrY:2654164-2656716	0
chr18	77748580	TXNL4A	kidney embryonic (CHR_S, PR_S)	104	56184	90.3990813	chr18:77747747-77749352	chr18:77747747-77749352	0	mesenchymal cell (ENH_F, PR_F);kidney e	106	57119	90.239305256	chr18:77747747-77749352	chr18:77747747-77749352	0	774	585194	chr18:77747292-77749805	chr18:77747292-77749805	0
chr10	127505271	UROS	erythroblast (ENH_E)	5	3218	99.4943376	chr10:127505037-127505723	chr10:127505037-127505723	0	erythroblast (ENH_E)	5	3218	99.4943376184	chr10:127505037-127505723	chr10:127505037-127505723	0	1271	636393	chr10:127504904-12750572	chr10:127504904-127505723	0
chr10	127505287	UROS	erythroblast (ENH_E)	5	3218	99.4943376	chr10:127505037-127505723	chr10:127505037-127505723	0	erythroblast (ENH_E)	5	3218	99.4943376184	chr10:127505037-127505723	chr10:127505037-127505723	0	1271	636393	chr10:127504904-12750572	chr10:127504904-127505723	0
chr10	127505291	UROS	erythroblast (ENH_E)	5	3218	99.4943376	chr10:127505037-127505723	chr10:127505037-127505723	0	erythroblast (ENH_E)	5	3218	99.4943376184	chr10:127505037-127505723	chr10:127505037-127505723	0	1271	636393	chr10:127504904-12750572	chr10:127504904-127505723	0

Core promoters

chr	pos	gene	tissues	num_features	features_size	specificity	regulator_overlapping	regulator_closest	regulator_closest_distance	tissues_WIDE	num_features_WIDE	features_size_WIDE	specificity	regulator_overlapping_WIDE	regulator_closest_WIDE	regulator_closest_distance_WIDE	num_features_ALL	features_size_ALL	regulator_overlapping_ALL	regulator_closest_ALL	regulator_closest_distance_ALL
chr8	41655164	ANK1	erythroblast (ENH_E)	26	17430	97.8906579	chr8:41654850-41655526	chr8:41654850-41655526	0	erythroblast (ENH_E)	26	17430	97.8906579017	chr8:41654850-41655526	chr8:41654850-41655526	0	1587	826324	chr8:41653034-41657665	chr8:41653034-41657665	0
chr11	116708365	APOA1	liver (CHR_E, ENH_E, ENH_F, ENH_S, PR_F, PR_S)	181	102565	84.3568407	chr11:116707492-116708880	chr11:116707492-116708880	0	liver (CHR_E, ENH_E, ENH_F, ENH_S, PR_	445	184353	71.8825783111	chr11:116702128-116708880	chr11:116702128-116708880	0	1174	655654	chr11:116702128-11670888	chr11:116702128-116708885	0
chr19	45449218	APOC2	liver (CHR_E, ENH_E, ENH_F, ENH_S, PR_F, PR_S)	279	157330	76.7028619	chr19:45448639-45449775	chr19:45448639-45449775	0	liver (CHR_E, ENH_E, ENH_F, ENH_S, PR_	435	193399	71.3618304831	chr19:45448639-45449775	chr19:45448639-45449775	0	1080	675319	chr19:45447849-45449775	chr19:45447849-45449775	0
chr13	52585683	ATP7B	liver (CHR_E, ENH_E, ENH_F, ENH_S, PR_F, PR_S)	119	79222	85.1850605	chr13:52585084-52586829	chr13:52585084-52586829	0	liver (CHR_E, ENH_E, ENH_F, ENH_S, PR_	471	180270	66.2885418069	chr13:52585084-52587121	chr13:52585084-52587121	0	1204	534744	chr13:52584571-52587330	chr13:52584571-52587330	0
chr16	56995796	CETP	liver (CHR_E, ENH_E, ENH_F, ENH_S, PR_F, PR_S)	202	125822	82.1813418	chr16:56994920-56996322	chr16:56994920-56996322	0	liver (CHR_E, ENH_E, ENH_F, ENH_S, PR_	272	144599	79.522180917	chr16:56994920-56996322	chr16:56994920-56996322	0	1229	706125	chr16:56994314-56996517	chr16:56994314-56996517	0
chr7	117119923	CFTR	lung (CHR_E, CHR_S, ENH_F, ENH_S, PR_F, PR_S)	212	75727	85.1185669	chr7:117118531-117120609	chr7:117118531-117120609	0	colonic mucosa (ENH_S, PR_S);lung (CHR	332	118465	76.7199416746	chr7:117118251-117120609	chr7:117118251-117120609	0	1269	508869	chr7:117117934-117120646	chr7:117117934-117120646	0
chr7	117119984	CFTR	lung (CHR_E, CHR_S, ENH_F, ENH_S, PR_F, PR_S)	212	75727	85.1185669	chr7:117118531-117120609	chr7:117118531-117120609	0	colonic mucosa (ENH_S, PR_S);lung (CHR	332	118465	76.7199416746	chr7:117118251-117120609	chr7:117118251-117120609	0	1269	508869	chr7:117117934-117120646	chr7:117117934-117120646	0
chr17	4806453	CHRNE	skeletal muscle tissue (ENH_F, ENH_S, PR_F, PR_S)	113	48245	92.5346575	chr17:4806293-4807188	chr17:4806293-4807188	0	skeletal muscle cell (CHR_E, ENH_F, PR_	786	240893	62.7246604658	chr17:4806293-4807188	chr17:4806293-4807188	0	1062	646253	chr17:4791898-4807685	chr17:4791898-4807685	0
chr17	4806454	CHRNE	skeletal muscle tissue (ENH_F, ENH_S, PR_F, PR_S)	113	48245	92.5346575	chr17:4806293-4807188	chr17:4806293-4807188	0	skeletal muscle cell (CHR_E, ENH_F, PR_	786	240893	62.7246604658	chr17:4806293-4807188	chr17:4806293-4807188	0	1062	646253	chr17:4791898-4807685	chr17:4791898-4807685	0
chr17	3539712	CTNS	kidney (CHR_E, CHR_S, ENH_F, PR_F, PR_S)	245	83109	85.7853391	chr17:3539374-3540116	chr17:3539374-3540116	0	thyroid gland (CHR_E, CHR_S, ENH_E, EN	383	125829	78.4786657796	chr17:3538798-3540575	chr17:3538798-3540575	0	1213	584671	chr17:3537834-3540788	chr17:3537834-3540788	0
chr17	3539720	CTNS	kidney (CHR_E, CHR_S, ENH_F, PR_F, PR_S)	245	83109	85.7853391	chr17:3539374-3540116	chr17:3539374-3540116	0	thyroid gland (CHR_E, CHR_S, ENH_E, EN	383	125829	78.4786657796	chr17:3538798-3540575	chr17:3538798-3540575	0	1213	584671	chr17:3537834-3540788	chr17:3537834-3540788	0
chr13	113760062	F7	liver (CHR_E, ENH_E, ENH_F, ENH_S, PR_F, PR_S)	254	99710	87.5431791	chr13:113759817-113760385	chr13:113759817-113760385	0	liver (CHR_E, ENH_E, ENH_F, ENH_S, PR_	515	156425	80.4577453791	chr13:113759817-113760680	chr13:113759817-113760680	0	1062	800445	chr13:113759485-11376068	chr13:113759485-113760680	0
chrX	154251045	F8	liver (CHR_E, ENH_E, ENH_F, ENH_S, PR_F, PR_S)	107	66727	88.1192367	chrX:154250725-154251241	chrX:154250725-154251241	0	liver (CHR_E, ENH_E, ENH_F, ENH_S, PR_	210	92776	83.4812041187	chrX:154250725-154251241	chrX:154250725-154251241	0	1431	561639	chrX:154250567-15425158(	chrX:154250567-154251580	0
chrX	154251048	F8	liver (CHR_E, ENH_E, ENH_F, ENH_S, PR_F, PR_S)	107	66727	88.1192367	chrX:154250725-154251241	chrX:154250725-154251241	0	liver (CHR_E, ENH_E, ENH_F, ENH_S, PR_	210	92776	83.4812041187	chrX:154250725-154251241	chrX:154250725-154251241	0	1431	561639	chrX:154250567-15425158(	chrX:154250567-154251580	0
chrX	154251082	F8	liver (CHR_E, ENH_E, ENH_F, ENH_S, PR_F, PR_S)	107	66727	88.1192367	chrX:154250725-154251241	chrX:154250725-154251241	0	liver (CHR_E, ENH_E, ENH_F, ENH_S, PR_	210	92776	83.4812041187	chrX:154250725-154251241	chrX:154250725-154251241	0	1431	561639	chrX:154250567-15425158(	chrX:154250567-154251580	0
chrX	154251084	F8	liver (CHR_E, ENH_E, ENH_F, ENH_S, PR_F, PR_S)	107	66727	88.1192367	chrX:154250725-154251241	chrX:154250725-154251241	0	liver (CHR_E, ENH_E, ENH_F, ENH_S, PR_	210	92776	83.4812041187	chrX:154250725-154251241	chrX:154250725-154251241	0	1431	561639	chrX:154250567-15425158(	chrX:154250567-154251580	0
chrX	138612869	F9	liver (CHR_E, ENH_E, ENH_F, ENH_S, PR_F, PR_S)	64	36326	92.7304528	chrX:138612605-138613590	chrX:138612605-138613590	0	liver (CHR_E, ENH_E, ENH_F, ENH_S, PR_	73	38640	92.2673758908	chrX:138612605-138613590	chrX:138612605-138613590	0	1630	499701	chrX:138612484-13861360	chrX:138612484-138613607	0
chrX	138612871	F9	liver (CHR_E, ENH_E, ENH_F, ENH_S, PR_F, PR_S)	64	36326	92.7304528	chrX:138612605-138613590	chrX:138612605-138613590	0	liver (CHR_E, ENH_E, ENH_F, ENH_S, PR_	73	38640	92.2673758908	chrX:138612605-138613590	chrX:138612605-138613590	0	1630	499701	chrX:138612484-13861360	chrX:138612484-138613607	0
chrX	138612872	F9	liver (CHR_E, ENH_E, ENH_F, ENH_S, PR_F, PR_S)	64	36326	92.7304528	chrX:138612605-138613590	chrX:138612605-138613590	0	liver (CHR_E, ENH_E, ENH_F, ENH_S, PR_	73	38640	92.2673758908	chrX:138612605-138613590	chrX:138612605-138613590	0	1630	499701	chrX:138612484-13861360	chrX:138612484-138613607	0
chrX	138612874	F9	liver (CHR_E, ENH_E, ENH_F, ENH_S, PR_F, PR_S)	64	36326	92.7304528	chrX:138612605-138613590	chrX:138612605-138613590	0	liver (CHR_E, ENH_E, ENH_F, ENH_S, PR_	73	38640	92.2673758908	chrX:138612605-138613590	chrX:138612605-138613590	0	1630	499701	chrX:138612484-13861360	chrX:138612484-138613607	0
chrX	138612875	F9	liver (CHR_E, ENH_E, ENH_F, ENH_S, PR_F, PR_S)	64	36326	92.7304528	chrX:138612605-138613590	chrX:138612605-138613590	0	liver (CHR_E, ENH_E, ENH_F, ENH_S, PR_	73	38640	92.2673758908	chrX:138612605-138613590	chrX:138612605-138613590	0	1630	499701	chrX:138612484-13861360	chrX:138612484-138613607	0
chrX	138612876	F9	liver (CHR_E, ENH_E, ENH_F, ENH_S, PR_F, PR_S)	64	36326	92.7304528	chrX:138612605-138613590	chrX:138612605-138613590	0	liver (CHR_E, ENH_E, ENH_F, ENH_S, PR_	73	38640	92.2673758908	chrX:138612605-138613590	chrX:138612605-138613590	0	1630	499701	chrX:138612484-13861360	chrX:138612484-138613607	0
chrX	146993405	FMR1	astrocyte (CHR_E, CHR_S, ENH_E, ENH_F, ENH_S, PR_F, PR_S)	909	175693	63.4299761	chrX:146993140-146993563	chrX:146993140-146993563	0	midbrain (CHR_S);astrocyte (CHR_E, CHR	1010	208450	56.6116949643	chrX:146993140-146994427	chrX:146993140-146994427	0	1676	480429	chrX:146991935-14699605	chrX:146991935-146996054	0
chrX	146993444	FMR1	astrocyte (CHR_E, CHR_S, ENH_E, ENH_F, ENH_S, PR_F, PR_S)	909	175693	63.4299761	chrX:146993140-146993563	chrX:146993140-146993563	0	midbrain (CHR_S);astrocyte (CHR_E, CHR	1010	208450	56.6116949643	chrX:146993140-146994427	chrX:146993140-146994427	0	1676	480429	chrX:146991935-14699605	chrX:146991935-146996054	0
chr7	31003560	GHRHR	brain (CHR_E, CHR_S, ENH_F, PR_F, PR_S)	279	105611	82.6910344	chr7:31003360-31003641	chr7:31003360-31003641	0	brain (CHR_E, CHR_S, ENH_F, PR_F, PR_	279	105611	82.6910343652	chr7:31003360-31003641	chr7:31003360-31003641	0	1265	610152	chr7:31002190-31006342	chr7:31002190-31006342	0
chrX	70443029	GJB1	liver (CHR_E, ENH_E, ENH_F, ENH_S, PR_F, PR_S)	97	47047	89.5328762	chrX:70442401-70443516	chrX:70442401-70443516	0	liver (CHR_E, ENH_E, ENH_F, ENH_S, PR_	109	51240	88.6000080094	chrX:70442401-70443516	chrX:70442401-70443516	0	1321	449474	chrX:70442395-70443543	chrX:70442395-70443543	0
chrX	70443031	GJB1	liver (CHR_E, ENH_E, ENH_F, ENH_S, PR_F, PR_S)	97	47047	89.5328762	chrX:70442401-70443516	chrX:70442401-70443516	0	liver (CHR_E, ENH_E, ENH_F, ENH_S, PR_	109	51240	88.6000080094	chrX:70442401-70443516	chrX:70442401-70443516	0	1321	449474	chrX:70442395-70443543	chrX:70442395-70443543	0
chr13	20767158	GJB2	mammary epithelial cell (CHR_E, CHR_S, ENH_E, ENH_F, ENH_S, PR_F, PR_	138	35716	94.0039016	chr13:20765373-20767284	chr13:20765373-20767284	0	mammary epithelial cell (CHR_E, CHR_S,	138	35716	94.0039015939	chr13:20765373-20767284	chr13:20765373-20767284	0	1403	595654	chr13:20761990-20768975	chr13:20761990-20768975	0
chr11	5248326	HBB	erythroblast (ENH_E)	30	20374	94.5459899	chr11:5248003-5248806	chr11:5248003-5248806	0	erythroblast (ENH_E);stromal cell of bone	64	34904	90.6563871935	chr11:5248003-5248806	chr11:5248003-5248806	0	1268	373560	chr11:5247589-5248806	chr11:5247589-5248806	0
chr11	5248329	HBB	erythroblast (ENH_E)	30	20374	94.5459899	chr11:5248003-5248806	chr11:5248003-5248806	0	erythroblast (ENH_E);stromal cell of bone	64	34904	90.6563871935	chr11:5248003-5248806	chr11:5248003-5248806	0	1268	373560	chr11:5247589-5248806	chr11:5247589-5248806	0
chr11	5248330	HBB	erythroblast (ENH_E)	30	20374	94.5459899	chr11:5248003-5248806	chr11:5248003-5248806	0	erythroblast (ENH_E);stromal cell of bone	64	34904	90.6563871935	chr11:5248003-5248806	chr11:5248003-5248806	0	1268	373560	chr11:5247589-5248806	chr11:5247589-5248806	0
chr11	5248331	HBB	erythroblast (ENH_E)	30	20374	94.5459899	chr11:5248003-5248806	chr11:5248003-5248806	0	erythroblast (ENH_E);stromal cell of bone	64	34904	90.6563871935	chr11:5248003-5248806	chr11:5248003-5248806	0	1268	373560	chr11:5247589-5248806	chr11:5247589-5248806	0
chr11	5248332	HBB	erythroblast (ENH_E)	30	20374	94.5459899	chr11:5248003-5248806	chr11:5248003-5248806	0	erythroblast (ENH_E);stromal cell of bone	64	34904	90.6563871935	chr11:5248003-5248806	chr11:5248003-5248806	0	1268	373560	chr11:5247589-5248806	chr11:5247589-5248806	0
chr11	5248333	HBB	erythroblast (ENH_E)	30	20374	94.5459899	chr11:5248003-5248806	chr11:5248003-5248806	0	erythroblast (ENH_E);stromal cell of bone	64	34904	90.6563871935	chr11:5248003-5248806	chr11:5248003-5248806	0	1268	373560	chr11:5247589-5248806	chr11:5247589-5248806	0
chr11	5248372	HBB	erythroblast (ENH_E)	30	20374	94.5459899	chr11:5248003-5248806	chr11:5248003-5248806	0	erythroblast (ENH_E);stromal cell of bone	64	34904	90.6563871935	chr11:5248003-5248806	chr11:5248003-5248806	0	1268	373560	chr11:5247589-5248806	chr11:5247589-5248806	0
chr11	5248374	HBB	erythroblast (ENH_E)	30	20374	94.5459899	chr11:5248003-5248806	chr11:5248003-5248806	0	erythroblast (ENH_E);stromal cell of bone	64	34904	90.6563871935	chr11:5248003-5248806	chr11:5248003-5248806	0	1268	373560	chr11:5247589-5248806	chr11:5247589-5248806	0
chr11	5248388	HBB	erythroblast (ENH_E)	30	20374	94.5459899	chr11:5248003-5248806	chr11:5248003-5248806	0	erythroblast (ENH_E);stromal cell of bone	64	34904	90.6563871935	chr11:5248003-5248806	chr11:5248003-5248806	0	1268	373560	chr11:5247589-5248806	chr11:5247589-5248806	0
chr11	5248389	HBB	erythroblast (ENH_E)	30	20374	94.5459899	chr11:5248003-5248806	chr11:5248003-5248806	0	erythroblast (ENH_E);stromal cell of bone	64	34904	90.6563871935	chr11:5248003-5248806	chr11:5248003-5248806	0	1268	373560	chr11:5247589-5248806	chr11:5247589-5248806	0
chr11	5248391	HBB	erythroblast (ENH_E)	30	20374	94.5459899	chr11:5248003-5248806	chr11:5248003-5248806	0	erythroblast (ENH_E);stromal cell of bone	64	34904	90.6563871935	chr11:5248003-5248806	chr11:5248003-5248806	0	1268	373560	chr11:5247589-5248806	chr11:5247589-5248806	0
chr10	71075518	HK1	erythroblast (ENH_E)	19	13285	98.0088787	chr10:71075199-71075891	chr10:71075199-71075891	0	erythroblast (ENH_E);brain (CHR_E, CHR_	216	88342	86.7595307039	chr10:71075199-71075891	chr10:71075199-71075891	0	1414	667212	chr10:71074631-71076699	chr10:71074631-71076699	0
chr12	121416289	HNF1A	hepatocyte (CHR_E, ENH_F, PR_F)	58	9540	98.4531996	chr12:121416140-121416360	chr12:121416140-121416360	0	endocrine pancreas (ENH_S, PR_S);liver	567	221315	64.1163375527	chr12:121415882-121416924	chr12:121415882-121416924	0	1328	616757	chr12:121415442-12141989	chr12:121415442-121419892	0
chr12	121416354	HNF1A	hepatocyte (CHR_E, ENH_F, PR_F)	58	9540	98.4531996	chr12:121416140-121416360	chr12:121416140-121416360	0	endocrine pancreas (ENH_S, PR_S);liver	567	221315	64.1163375527	chr12:121415882-121416924	chr12:121415882-121416924	0	1328	616757	chr12:121415442-12141989	chr12:121415442-121419892	0
chr11	2182532	INS	pancreas (CHR_E, CHR_S, ENH_F, ENH_S, PR_F, PR_S)	129	67156	91.624481	None	chr11:2178120-2178439	4093	body of pancreas (CHR_E, CHR_S, ENH_E	775	228706	71.4763916275	chr11:2182191-2182675	chr11:2182191-2182675	0	756	801813	chr11:2182191-2182675	chr11:2182191-2182675	0
chr11	2182533	INS	pancreas (CHR_E, CHR_S, ENH_F, ENH_S, PR_F, PR_S)	129	67156	91.624481	None	chr11:2178120-2178439	4094	body of pancreas (CHR_E, CHR_S, ENH_E	775	228706	71.4763916275	chr11:2182191-2182675	chr11:2182191-2182675	0	756	801813	chr11:2182191-2182675	chr11:2182191-2182675	0
chr2	47630106	MSH2	sigmoid colon (CHR_S, ENH_E, ENH_S, PR_S)	109	60280	90.9663787	chr2:47629602-47630514	chr2:47629602-47630514	0	colonic mucosa (ENH_S, PR_S);uterus (CH	270	141749	78.7573525555	chr2:47629602-47631417	chr2:47629602-47631417	0	1346	667285	chr2:47628558-47631927	chr2:47628558-47631927	0
chr1	160001799	PIGM	granulocyte (ENH_F, PR_F)	2	691	99.8922469	chr1:160001625-160001888	chr1:160001625-160001888	0	granulocyte (ENH_F, PR_F);skin fibroblast	75	39574	93.8289143137	chr1:160001472-160002103	chr1:160001472-160002103	0	1046	641281	chr1:160000007-16000301	chr1:160000007-160003011	0
chr1	155271258	PKLR	erythroblast (ENH_E)	33	22728	96.57028	chr1:155271134-155271552	chr1:155271134-155271552	0	liver (CHR_E, ENH_E, ENH_F, ENH_S, PR_	319	155234	76.5747467096	chr1:155270459-155271785	chr1:155270459-155271785	0	1263	662678	chr1:155270435-15527179	chr1:155270435-155271795	0
chr1	155271269	PKLR	erythroblast (ENH_E)	33	22728	96.57028	chr1:155271134-155271552	chr1:155271134-155271552	0	liver (CHR_E, ENH_E, ENH_F, ENH_S, PR_	319	155234	76.5747467096	chr1:155270459-155271785	chr1:155270459-155271785	0	1263	662678	chr1:155270435-15527179	chr1:155270435-155271795	0
chr6	118869382	PLN	heart (CHR_E, CHR_S, ENH_F, PR_F, PR_S)	238	97055	79.3337308	chr6:118869250-118869598	chr6:118869250-118869598	0	heart left ventricle (CHR_E, CHR_S, ENH_	386	138283	70.5549049252	chr6:118869250-118869620	chr6:118869250-118869620	0	1225	469630	chr6:118868683-11887088	chr6:118868683-118870885	0
chr14	73603081	PSEN1	heart (CHR_E, CHR_S, ENH_F, PR_F, PR_S)	256	100715	83.5342068	chr14:73602751-73604090	chr14:73602751-73604090	0	heart left ventricle (CHR_E, CHR_S, ENH_	462	166019	72.8577220753	chr14:73602598-73604688	chr14:73602598-73604688	0	1404	611662	chr14:73602250-73604809	chr14:73602250-73604809	0
chr13	48877851	RB1	retina (CHR_E, CHR_S, PR_F)	98	42666	91.5624648	chr13:48877088-48879187	chr13:48877088-48879187	0	retina (CHR_E, CHR_S, PR_F);retina embry	131	48267	90.4548232144	chr13:48877088-48879187	chr13:48877088-48879187	0	1156	505669	chr13:48876128-48880341	chr13:48876128-48880341	0
chr13	48877856	RB1	retina (CHR_E, CHR_S, PR_F)	98	42666	91.5624648	chr13:48877088-48879187	chr13:48877088-48879187	0	retina (CHR_E, CHR_S, PR_F);retina embry	131	48267	90.4548232144	chr13:48877088-48879187	chr13:48877088-48879187	0	1156	505669	chr13:48876128-48880341	chr13:48876128-48880341	0
chr13	48877860	RB1	retina (CHR_E, CHR_S, PR_F)	98	42666	91.5624648	chr13:48877088-48879187	chr13:48877088-48879187	0	retina (CHR_E, CHR_S, PR_F);retina embry	131	48267	90.4548232144	chr13:48877088-48879187	chr13:48877088-48879187	0	1156	505669	chr13:48876128-48880341	chr13:48876128-48880341	0
chr9	35658032	RMRP	liver (CHR_E, ENH_E, ENH_F, ENH_S, PR_F, PR_S)	182	107196	82.8425182	chr9:35657224-35659234	chr9:35657224-35659234	0	kidney embryonic (CHR_S, PR_S);liver (C	235	140733	77.4746829669	chr9:35657224-35659234	chr9:35657224-35659234	0	1194	624777	chr9:35655512-35659967	chr9:35655512-35659967	0
chr1	173886568	SERPINC1	liver (CHR_E, ENH_E, ENH_F, ENH_S, PR_F, PR_S)	83	53476	86.7772437	chr1:173886107-173886858	chr1:173886107-173886858	0	liver (CHR_E, ENH_E, ENH_F, ENH_S, PR_	123	62427	84.5639724645	chr1:173886107-173887749	chr1:173886107-173887749	0	1145	404424	chr1:173885439-17388774	chr1:173885439-173887749	0
chr3	169482906	TERC	stromal cell of bone marrow (CHR_E, CHR_S)	50	28949	93.7889278	chr3:169482001-169483429	chr3:169482001-169483429	0	stromal cell of bone marrow (CHR_E, CHR	50	28949	93.7889278182	chr3:169482001-169483429	chr3:169482001-169483429	0	1151	466087	chr3:169480373-16948348	chr3:169480373-169483483	0
chr3	169482947	TERC	stromal cell of bone marrow (CHR_E, CHR_S)	50	28949	93.7889278	chr3:169482001-169483429	chr3:169482001-169483429	0	stromal cell of bone marrow (CHR_E, CHR	50	28949	93.7889278182	chr3:169482001-169483429	chr3:169482001-169483429	0	1151	466087	chr3:169480373-16948348	chr3:169480373-169483483	0
chr11	2193085	TH	brain (CHR_E, CHR_S, ENH_F, PR_F, PR_S)	226	85589	89.3493959	chr11:2192970-2193300	chr11:2192970-2193300	0	substantia nigra (ENH_S, PR_S);brain (C	254	98357	87.760559577	chr11:2192970-2193300	chr11:2192970-2193300	0	748	803607	chr11:2192522-2193545	chr11:2192522-2193545	0
chr11	2193086	TH	brain (CHR_E, CHR_S, ENH_F, PR_F, PR_S)	226	85589	89.3493959	chr11:2192970-2193300	chr11:2192970-2193300	0	substantia nigra (ENH_S, PR_S);brain (C	254	98357	87.760559577	chr11:2192970-2193300	chr11:2192970-2193300	0	748	803607	chr11:2192522-2193545	chr11:2192522-2193545	0
chr11	2193087	TH	brain (CHR_E, CHR_S, ENH_F, PR_F, PR_S)	226	85589	89.3493959	chr11:2192970-2193300	chr11:2192970-2193300	0	substantia nigra (ENH_S, PR_S);brain (C	254	98357	87.760559577	chr11:2192970-2193300	chr11:2192970-2193300	0	748	803607	chr11:2192522-2193545	chr11:2192522-2193545	0
chr2	234668851	UGT1A1	liver (CHR_E, ENH_E, ENH_F, ENH_S, PR_F, PR_S)	225	106848	82.8481442	chr2:234668556-234669718	chr2:234668556-234669718	0	liver (CHR_E, ENH_E, ENH_F, ENH_S, PR_	232	108531	82.5779793981	chr2:234668556-234669718	chr2:234668556-234669718	0	1237	622953	chr2:234668075-23466971	chr2:234668075-234669718	0

Overall median				98	44222	92.7304528					254	98357	82.4877177595				1265	561639			
